# Transcriptome analysis reveals a *de novo* DNA element that may interact with chromatin-associated proteins in *Plasmodium berghei* during erythrocytic development

**DOI:** 10.1038/s41598-025-03586-4

**Published:** 2025-05-28

**Authors:** Adaobi Okafor, Yagoub Adam, Benedikt Brors, Ezekiel Adebiyi

**Affiliations:** 1 Applied Bioinformatics, German Cancer Research Center (DKFZ), Im Neuenheimer Feld 280, 69120 Heidelberg, Germany; 2 Faculty of Biosciences, Heidelberg University, Im Neuenheimer Feld 234, 69120 Heidelberg, Germany; 3 Covenant University Bioinformatics Research (CUBRe), Covenant University, Ota, 112233 Ogun State Nigeria; 4 African Center of Excellence in Bioinformatics & Data Intensive Science, Makerere University, 10218 Kampala, Uganda; 5 Institute of Infectious Diseases (IDI), Makerere University, 10218 Kampala, Uganda

**Keywords:** *Plasmodium berghei*, RNA-Seq, ApiAP2 proteins, Regulatory motifs, *Plasmodium falciparum*, *Plasmodium vivax*, Computational biology and bioinformatics, Pathogenesis

## Abstract

The life cycle of *Plasmodium* parasites involves intricate, multistage processes that are tightly regulated by stage-specific transcription factors. These factors bind to regulatory regions within gene promoters, enabling the precise expression of genes required for each developmental stage. Despite the importance of these transcriptional mechanisms, our understanding remains limited, particularly in the rodent model organism *P. berghei.* To address this, we conducted a genome-wide analysis of RNA-Seq data from different developmental stages of *P. berghei* by initially integrating data from human malaria parasites *P. falciparum* and *P. vivax*. We identified unique transcriptional signatures across *Plasmodium* species. Our analysis of *P. berghei* revealed stage-specific gene sets clustered by expression profiles and predicted regulatory motifs involved in their control. We interpreted these motifs using known binding sites for eukaryotic transcription factors including ApiAP2 proteins. Additionally, we expanded the annotation of the AGGTAA motif which resembles a de novo motif linked to erythrocytic development in *P. falciparum*, and identified its potential interacting proteins including members of the PfMORC and GCN5 complexes. This study enhances our understanding of gene regulation in P. berghei and provides new insights into the transcriptional dynamics underlying *Plasmodium* development.

## Introduction

Malaria, an acute fever illness, is caused by plasmodial infection^1^. The intricate life cycle of *Plasmodium* parasites is marked by multiple developmental stages and environmental transitions and includes sexual reproduction in female *Anopheles* mosquito vectors and asexual reproduction in vertebrate host tissues particularly liver cells (hepatocytes) and red blood cells (erythrocytes)^2^. Temporal regulation of transcription by stage-specific transcription factor binding at particular regulatory elements upstream of genes facilitates the progression of the stage transitions^3^. In *Plasmodium* species, the ApiAP2 protein family, which contains Apicomplexan AP2 DNA-binding domains, was first identified in *Plasmodium falciparum*. This protein family serves as a key regulator of stage-specific transcription and is crucial for coordinating the parasite’s life cycle progression^4,5^. Protein binding microarray experiments also revealed the DNA binding preferences for 20 of the 27 *P. falciparum* ApiAP2 factors during the intraerythrocytic development cycle (IDC)^6^. However, the binding patterns of these factors across the entire life cycle stages remain unknown. Research is currently being conducted to explore the complex regulatory networks and cis-acting sequences that are crucial for organizing heterochromatin and modulating gene expression within it. Several ChIP-Seq studies have confirmed these binding prefernces and identified new ones as well. A study by Shang et al.^7^ has identified eight heterochromatin-associated factors, known as PfAP2-HFs. These factors are primarily found in heterochromatic regions although their coverage can vary significantly. Notably, the ApiAP2 factors can also target euchromatic gene locations based on specific DNA motifs they recognize^7^. Furthermore, Shang et al. suggested that these factors may have independent functions within heterochromatin indicating a complex interplay in the regulation of gene expression across different chromatin environments^7^.

An important and often used rodent-infecting model organism in biomedical research is *Plasmodium berghei*. *P. falciparum* has been the subject of the majority of studies on malaria putting *P. berghei* somewhat behind. The entire genomes of *Plasmodium* parasites are now accessible for functional genomics research due to extensive genome sequencing efforts^8,9^. Furthermore, much effort has been made in the last 20 years to utilize RNA-Seq to analyse the bulk transcriptomes of human malaria parasites with a primary focus on the blood stages and sporadic exploration of the mosquito stages (Supplementary Table S1). Despite the power of scRNA-seq in uncovering cellular heterogeneity and rare cell populations^10–15^ (Supplementary Table S1), bulk RNA-seq data was chosen for this study as it averages out cell-to-cell variability, reduces technical variability and has a lower incidence of drop-out events providing a more consistent measure of gene expression. Moreover, low capture rate is inherent in single-cell approaches^12^.

Moral and ethical issues hamper performing in-depth studies of the whole *Plasmodium* life cycle in humans^16^. *P. berghei* is an option that provides experimental robustness and has a well-established and standardised genetic modification methodology for in vivo research^16,17^. A few studies have recently investigated the bulk transcriptome of *P. berghei* focusing especially on individual analyses of the blood and mosquito stages (Supplementary Table S1). Notably, the two most recent RNA-Seq studies throughout liver stages were made possible by the *P. berghei* model resulting in data that now cover most of the life cycle^16,18,19^. Both studies examined *P. berghei* gene transcription using extensive available data but their primary areas of interest were the liver stages. Moreover, only one^18^ examined transcriptional regulation and they restricted the scope of their research to the liver stages.

A variety of innovative approaches have been explored to facilitate the in silico discovery of transcription regulatory elements within Plasmodium species. One notable example is the work by Young et al.^20^ who developed an advanced computational tool known as the Gene Enrichment Motif Searching (GEMS) algorithm. This algorithm is specifically designed to analyse cis-regulatory elements found in the P. falciparum genome which is crucial for understanding gene regulation in this parasite^20^. The GEMS algorithm relies on a hypergeometric scoring function, a statistical method that determines the significance of identified motifs within a given dataset^20^. Additionally, it uses sophisticated position-weight matrix optimization techniques to accurately detect meaningful cis-regulatory motifs that play essential roles in regulating gene expression. This work represents a significant advancement in our ability to map and understand the complex regulatory landscape of P. falciparum^20^.

A further advancement was made by Elemento et al.^21^ who developed a detailed and robust framework for analysing sequence motifs. Their approach focuses on assessing the mutual information between sequence data and gene expression measurements providing valuable insights into how genetic sequences relate to functional outcomes in cells. This framework distinguishes itself by making minimal assumptions about the underlying motif models and the biological mechanisms that drive gene expression^21^. This flexibility makes their method an adaptable tool for motif discovery across various data types and genomic contexts. Notably, it demonstrates exceptional sensitivity in detecting relevant motifs while maintaining a low rate of false positives thereby enhancing the reliability of the findings^21^.

The aim of our study is to conduct an integrated analysis that examines gene expression and regulatory mechanisms across the various stages of the *P. berghei* life cycle, within its unique host environments. Such a holistic approach is currently lacking in the literature. By synthesizing data from multiple studies, we seek to identify consistent expression patterns across multiple stages and uncover cross-stage co-regulation networks that are critical for the parasite’s survival and development. This research will contribute to a more cohesive understanding of gene regulation in *Plasmodium* offering new insights that individual studies have not fully captured.

## Methods

### Dataset

Bulk RNA-Seq data derived from wild type samples comprising most of the life cycle stages of *P. berghei*, *P. falciparum* and *P. vivax*, namely, salivary gland sporozoite; liver stages; ring, trophozoite, schizont (or mixed asexual blood stages, where unavailable); gametocyte and ookinete, generated from two independent studies (Supplementary Table S2) were downloaded as Sequence Read Archive (SRA) files from the public archives at the National Center for Biotechnology Information (NCBI) using sra-toolkit version 2.11.0^22^. The SRA files were converted into FASTQ files using ‘fastq-dump’ within the sra-toolkit. Reference genome FASTA files and associated gene annotation GTF files for each genome [accession numbers: ASM276v2 (*P. falciparum*), *PB*ANKA01 (*P. berghei*) and GCA 900,093,555 (*P. vivax*)] were also downloaded directly from the Ensembl Protists genome browser (release 55)^23^.

### Data preprocessing

All the raw read sequences were checked for quality with FASTQC version 0.11.9^24^. Sequencing adapters, low-quality reads and short reads were trimmed off using Trimmomatic version 0.39^25^ with the following parameters: ILLUMINACLIP: TruSeq2 (SE) or TruSeq3 (PE) or Nextera (PE) LEADING: 3 TRAILING: 3 SLIDINGWINDOW: 4:20 MINLEN: 20. The read sequences with a minimum length of 20 nucleotides and whose bases had a minimum Phred quality score of 20 were retained. Trimmed read sequences were rechecked with FastQC and aligned to their respective reference genomes and gene annotation files (Ensembl Protists release 51) using STAR version 2.7.8a^26^ with the following parameters: -sjdbOverhang (“read length” – 1)—limitBAMsortRAM 50,000,000,000. The strand specificities of the read sequences were confirmed prior to read quantification using the tool “how are we stranded here” version 1.0.1^27^. To quantify the aligned reads, the reads that only uniquely mapped to exonic regions in the alignment files were counted using ‘featureCounts’ from the Subread package version 2.0.1^28^ with the following parameters: -s Stranded (Reverse) or Unstranded -p (for PE reads) -t exon -g gene id.

### Differential gene expression analysis

Differential gene expression analysis was performed with DESeq2 version 1.22.2^29^. The read counts for *P. berghei* were integrated into a single matrix with those of *P. falciparum* and *P. vivax* which were used to assess the quality of the *P. berghei* datasets prior to the analysis. The *P. falciparum* and *P. vivax* gene ids were transformed by orthology into *P. berghei* gene ids using the BioMart tool in the Ensembl Protists genome browser (release 51) which included only the genes with 1:1:1 orthologues across the three species. A Spearman correlation test with hierarchical clustering was performed on the 500 most variable genes to evaluate the correlation of the *P. berghei* datasets with the *P. falciparum* and *P. vivax* datasets. Batch effects in the datasets were corrected using ‘removeBatchEffect’, implemented in limma^30^ prior to dimensionality reduction by principal component analysis (PCA) on the 500 most variable genes. The raw counts for the entire *P. berghei* dataset were analysed for differential expression using DESeq2 version 1.22.2^29^ which included the parasite stages in the design formula as the variable of interest. Each stage served as the factor level of interest and was compared with all other stages as controls in each pairwise comparison. The results of each pairwise comparison were generated including the log2-fold changes, p values and FDR-adjusted p values that control for multiple testing^31^ and the differentially expressed genes (DEGs) for each comparison were then filtered out (padj < 0.01 and log2 FC > 2 or < −2). To determine the total number of DEGs for each stage, the union of all pairwise comparisons involving that stage and direction of regulation was performed (Fig. 2A). Furthermore, for each parasite stage, the upregulated genes that were consistent in the results of the analysis of the datasets from the corresponding two independent studies were filtered out for use in downstream analysis and visualized with a Venn diagram^32^ (Fig. 2B). Normalized read counts for use in subsequent clustering analysis were also extracted with DESeq2 version 1.22.2^24^. Plots were obtained using the ggplot2 R package version 3.4.2^33^, and all heatmaps relating to the gene expression analysis were obtained using the ComplexHeatmap R package version 2.16.0^34^.

### Functional annotation

Gene Ontology (GO) terms related to biological processes for the upregulated genes (release 43) and genes in the coexpression clusters were retrieved from PlasmoDB (release 51)^35^. Redundant gene ontology terms were removed with the REVIGO webtool version 1.8.1 using the following parameters: Similarity set to 0.7 (medium), GO term sizes set to *P. falciparum* and semantic similarity measures set to SimRel^36^.

### Co-expression clustering analysis

For the identification of groups of genes with similar expression profiles, PAM analysis with hierarchical clustering was performed on scaled, normalized counts using the ComplexHeatmap R package version 2.16.0. The optimal number of clusters of similarly expressed genes in the dataset was determined using the factoextra R package version 1.0.7^37^ employing the elbow and silhouette methods.De novo motif discovery to identify putative regulatory sequences underlying the observed coexpression patterns, the promoter regions of the genes in each coexpression cluster were searched for overrepresentation of sequence motifs. First, the 5’UTR sequences 1000 bp upstream of the start codons of the genes were extracted from PlasmoDB (release 51). Motif analysis was performed with the ‘oligo analysis’ tool implemented in RSAT version 2020–02–03^38^ with default parameters, background model (expected frequency of eacholigonucleotide)estimatedfrom*Plasmodium berghei*.*PB*ANKA01.34 genome, number of pattern assemblies set to 50 and matrix clustering turned off and with STREME from the MEME Suite version 5.5.4^39^ with default universal and STREME parameters and MEME expected motif site distribution set to ‘any number of repetitions’.

For each co-expression cluster, the resulting motif collections from both approaches were combined into a single collection. To ensure uniformity, the MEME motif collections in.meme format were converted to.transfac formats compatible with RSAT using the ‘convert matrix’toolfromRSAT^38^ with defaultparameters,selectingthe *Plasmodium berghei*.*PB*ANKA01.34 genome from the list of provided organisms. Each transfac collection was clustered using the ‘matrix-clustering’ tool from RSAT^38^ with default parameters to group similar motifs together and determine a root motif representing the motifs in each collection, thus reducing the redundancy of the motifs and simplifying the interpretation of motif discovery results.

### Biological interpretation of the discovered motifs

The similarity of the discovered motifs to known motifs was determined by comparison to motifs in the JASPAR database^40^ and transcription factor-binding sites discovered in *P. falciparum* through protein binding microarrays (*PB*M)^6^ and assay for transposase-accessible chromatin using sequencing (ATAC-Seq)^41^. The TOMTOM tool from the MEME Suite^39^ was used with the following parameters: Euclidean distance as the comparison function, e-value less than 1 as the significance threshold and complete scoring turned off to ensure that only the aligned motif columns were considered in computing the comparison function. For compatibility, the.transfac motif collections from RSAT^38^ were converted to.meme format using the command ‘transfac2 meme’ from the MEME Suite^39^ with default parameters.

### Promoter architecture

To visualize the promoter architectures of *P. berghei* genes with suspected related functions in *P. falciparum*, the 5’UTR sequences 1000 bp upstream of the start codon of their orthologues were queried for the individual genomic locations of the regulatory motifs of interest within them using ‘matrix-scan’ in RSAT^38^ with default parameters. The scan or “feature list” was visualized using the ‘feature map’ tool within RSAT^38^ with default parameters.

### Gene regulatory network construction

To identify functional relationships that may exist between motifs and their target genes, the target genes harbouring the motifs were identified using ‘matrix-scan’. The gene regulatory network of the interactions of the motifs with their target genes was conducted using Cytoscape version 3.10.0^42^ and the numbers of unique and shared target genes among the motifs were visualized with a Venn diagram.

### Motif activity inference

Motif activity scores were calculated using an adaptation of the motif activity response analysis (MARA) model^43^. Read counts were used as a proxy for promoter expression levels^44^, and the number of sites (genomic locations) of the motifs was predicted using the ‘matrix-scan’ tool. The temporal motif activity profiles were scaled and visualized as a line plot.

### Motif enrichment

Transformation of *P. berghei* genes into *P. falciparum*, *P. vivax*, *P. knowlesi* and *P. ovale* orthologs and extraction of the 5’UTR sequences 1000 bp upstream of their start codons were done in PlasmoDB version 68. Motif enrichment analysis was performed on each gene set using the SEA tool from the MEME Suite^45^.

### Cross-correlation analysis

Cross-correlation analysis was performed for the blood stages (ring, trophozoite and schizont) on ring stage-delayed transcription factor expression data^9^ and leading motif activity profile data after scaling to identify transcription factors expressed in the ring stage that could subsequently bind to motifs in the trophozoite and schizont stages. Pearson correlation with hierarchical clustering (Euclidean distance, “complete” method) was applied to the time series data and visualized with a heatmap using the Heatmaply package version 1.4.2^46^. Further details of data analysis are available in the Supplementary Methods.

### Analysis of motifs in orthologous protein-coding genes

We conducted a comprehensive analysis of functionally annotated sequence motifs in orthologous protein-coding genes. To achieve this, we utilized the BioMart tool from the Ensembl Protists genome browser (release 60) to download the orthologous protein-coding genes of Plasmodium berghei which are also orthologous to both Plasmodium falciparum and Plasmodium vivax.

Before performing the differential gene expression analysis, we filtered out 1,827 differentially expressed genes (DEGs) from the initial dataset, which included all genes (both non-coding and duplicated) in P. berghei. We carried out the differential gene expression analysis using the DESeq2 R package to extract the differentially expressed orthologous protein-coding genes across the developmental stages.

To identify robust gene clusters, we utilized the bootstrap method from the fpc R package^47^. This technique employs statistical resampling to generate stable gene clusters. We set the parameter B which indicates the number of resampling runs, to 100 in line with the recommendations outlined in the documentation. Additionally, we assessed various cluster numbers (k) ranging from 3 to 10 to evaluate the stability and suitability of the clustering. The fpc R package is well-suited for selecting the most robust clusters that accurately reflect the underlying data structure across a range of k values.

Following this initial clustering phase, we implemented K-means clustering methods to further subdivide each cluster into refined subclusters which formed the basis for subsequent motif discovery analysis. Using the MEME Suite and Matrix-Scan tools, we performed motif discovery for genes within all identified subclusters. The outputs generated from the MEME Suite were meticulously annotated with the help of the JASPAR2022^40^ and TFBSTools^48^ R packages.

### Analysis of motifs in experimentally validated chip-seq protein binding signals in orthologous protein-coding genes

In addition to exploring motifs using bulk RNA-seq data, we conducted a thorough analysis of ChIP-seq data to identify key regulatory sequences, such as AGGTAA and GCACTA, and to assess their alignment with experimentally validated binding sites of various DNA-binding proteins. The ChIP-seq data, obtained from Plasmodium falciparum strain 3D7 were sourced from Chahine et al. (2024)^49^. Our analysis focused on sequences derived from ChIP-seq signals located within 1,000 base pairs upstream of conserved protein-coding genes. These conserved protein-coding genes in Plasmodium falciparum 3D7, that are also found in Plasmodium berghei, were retrieved using the BioMart tool from the Ensembl Protists genome browser.

As in the analysis of orthologous protein genes, motif discovery for sequences in the filtered ChIP-seq signals was performed using the MEME Suite and Matrix-Scan tools. The outputs generated from the MEME Suite were carefully annotated with the assistance of the JASPAR2022 and TFBSTools R packages.

## Results

### *Plasmodium* parasites have a unique transcriptional signature

**Fig. 1:**
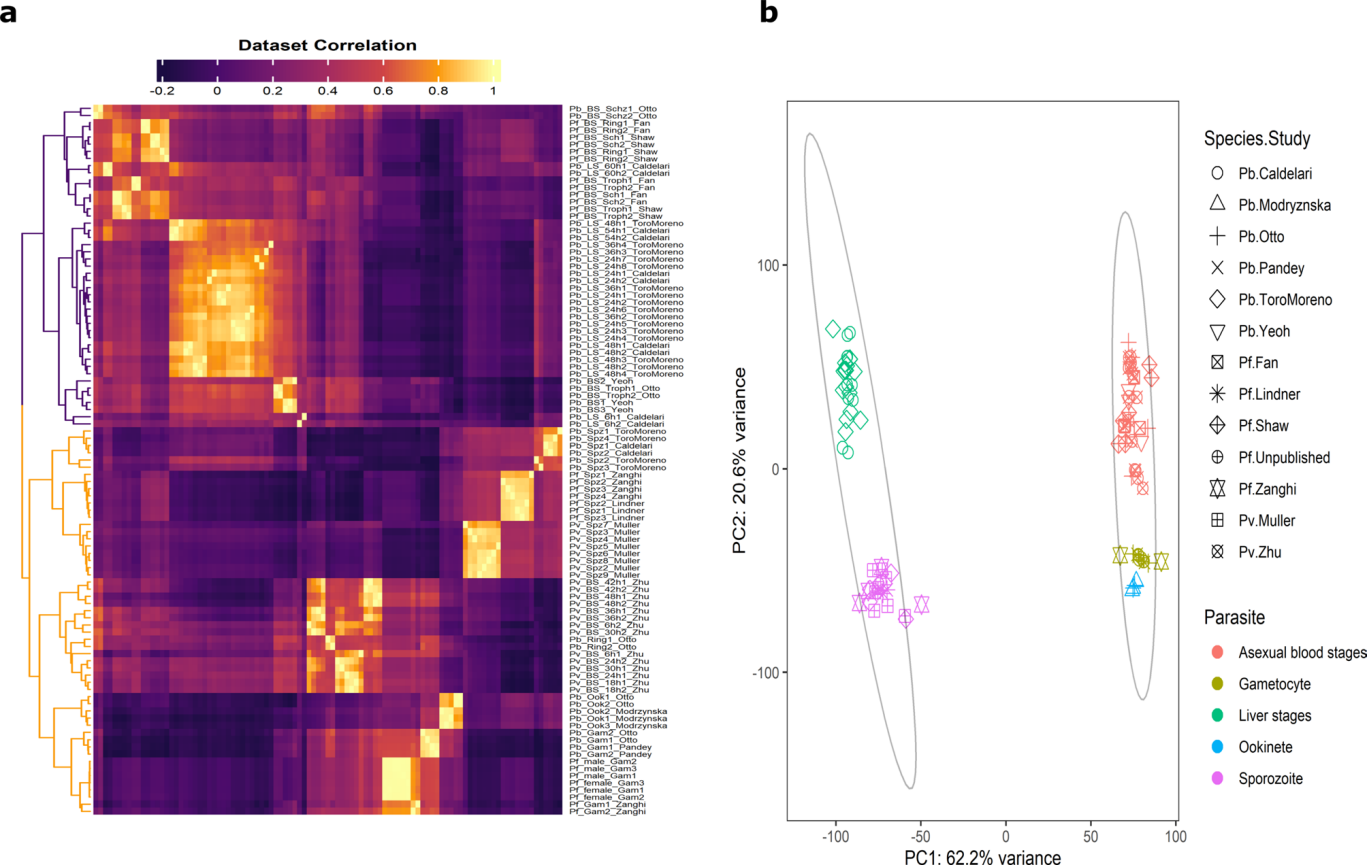
Overview of the *Plasmodium* transcriptome in different species based on the 500 most variable genes. a. Heatmap of sample-to-sample Spearman correlation of RNA-Seq expression values and b. PCA plot showing the clustering of samples based on their similarity. The rows (**A**) and data points (**B**) depict all samples and their biological replicates from different *Plasmodium* spp. generated in different studies.

The correlation of the highest variable genes in *P. berghei*, *P. falciparum* and *P. vivax* was used to assess the overall similarity of the data from the *P. berghei* samples to those of the other species, revealing two large patterns of clustering in the datasets. For all the species, the expression profiles of the asexual blood stages and liver stages clustered together in the first cluster, whereas gametocytes, ookinetes and sporozoites made up the majority of the second cluster (Fig. 1A).

In general, the two clusters of stages can be described as primarily consisting of (i) intracellular, metabolically active stages and (ii) extracellular, sexual, and motile stages (Fig. 1;^16,18^). This is in contrast with the canonical description of the *Plasmodium* life cycle, which describes the life cycle in terms of the host environment. With the exception of gametocyte formation from asexual progenitors in host red blood cells, the life cycle is essentially the same in this"host model."Transcriptomics, however, has made it possible to add more details to the life cycle description. The gametocytes in this“transcriptomic model”resemble the motile, extracellular stages of the mosquito vector more closely than the metabolically active, internal asexual stages found in the vertebrate host. The observed transcriptomic differences between parasite species may reflect adaptations to different host environments or evolutionary divergence. However, transcriptomic differences between stages of the different species are less pronounced suggesting that the core regulatory mechanisms controlling gene expression are conserved across species, while species-specific adaptations mainly involve fine-tuning of gene expression by epigenetic mechanisms^50^.

Regardless of the species or study from which the data were derived, a general grouping of all biological replicates by parasite stages was found using PC analysis of the most variable genes (Fig. 1B). Within the first two PCs, 82.8% of the total variance explained by the parasite stages was observed. The parasite stages were divided into two sizable groups, much like in the correlation analysis, to show two distinct underlying patterns. The sporozoites and liver stages composed the first cluster along PC1 and the asexual blood stages, gametocytes and ookinetes composed the second cluster.

### RNA-Seq data analysis of P. berghei

**Fig. 2:**
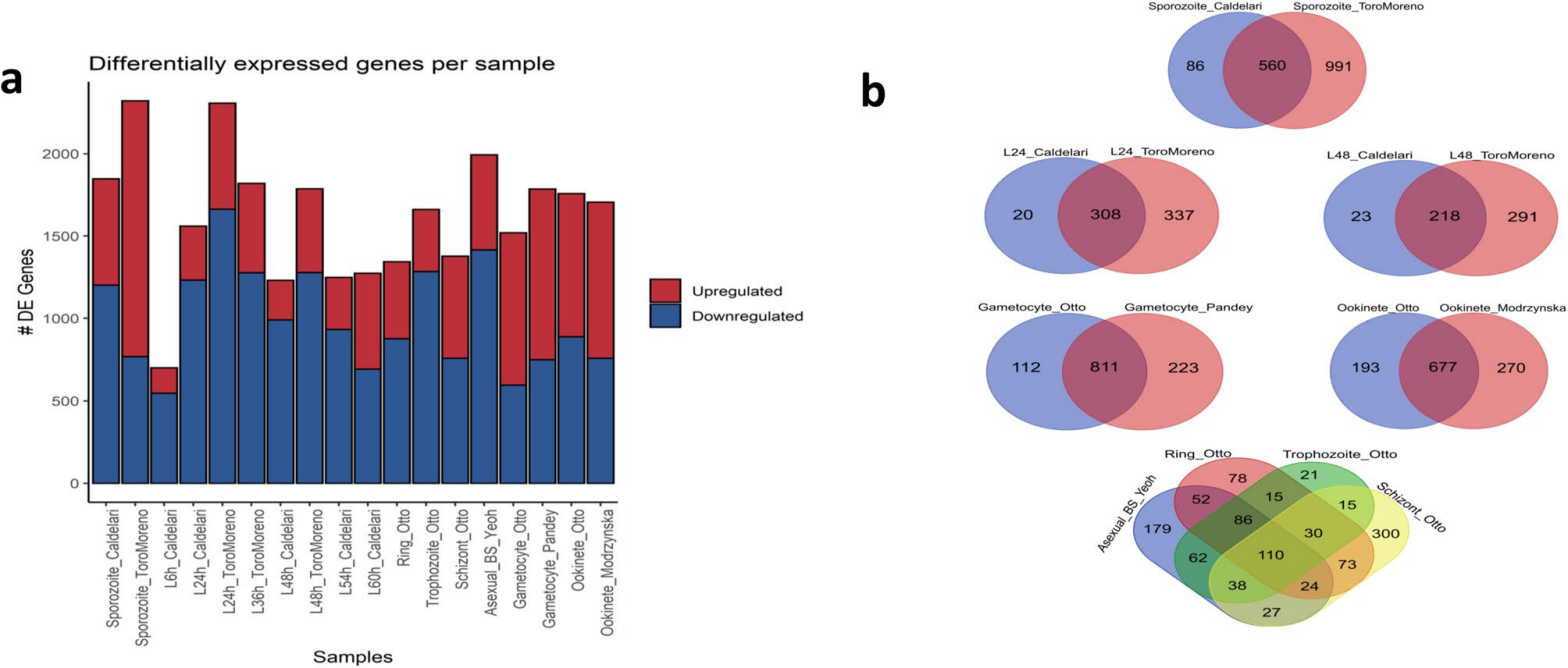
Identification of DEGs in the *P. berghei* samples. (**a**). Total DEGs from the comparison of each stage vs all other stages summarized for each *P. berghei* sample from each study (padj < 0.01, abs(log2 FC) > 2). (**b**) Intersection of upregulated genes identified in each *P. berghei* sample from two independent studies.

Differential gene expression was investigated throughout the life cycle stages of *P. berghei* to better understand gene transcription during its development. A total of 3355 DEGs were detected at an adjusted p value (FDR) of less than 0.01 and a minimum log2 FC of 2. With the exception of the liver 6 h post infection (hpi) stage (L6 h)^16^ which contained 701 DEGs (Fig. 2A). most likely due to fewer transcripts from the parasite at this stage^16^, over 1000 statistically significant DEGs (padj < 0.01, abs(log2 FC) > 2) were found in each stage. With 1231 genes identified, the liver stage at 48 hpi (L48 h)^16^ had the second-lowest number of DEGs discovered. The highest number of DEGs found was 2321 genes during the sporozoite stage^18^ while the median number of DEGs found (apart from L6 h) was 1706 genes during the ookinete stage^51^. As anticipated, there were variations in the number of DEGs produced for identical samples from two separate studies, underscoring the technical distinctions in RNA extraction, library preparation techniques, RNA sequencing depth, and sequencing platforms (Fig. 2A).

Filtering the upregulated genes identified for each stage in the results of their two corresponding independent studies (Fig. 2B) revealed 1832 genes for use in downstream analysis.

**Fig. 3:**
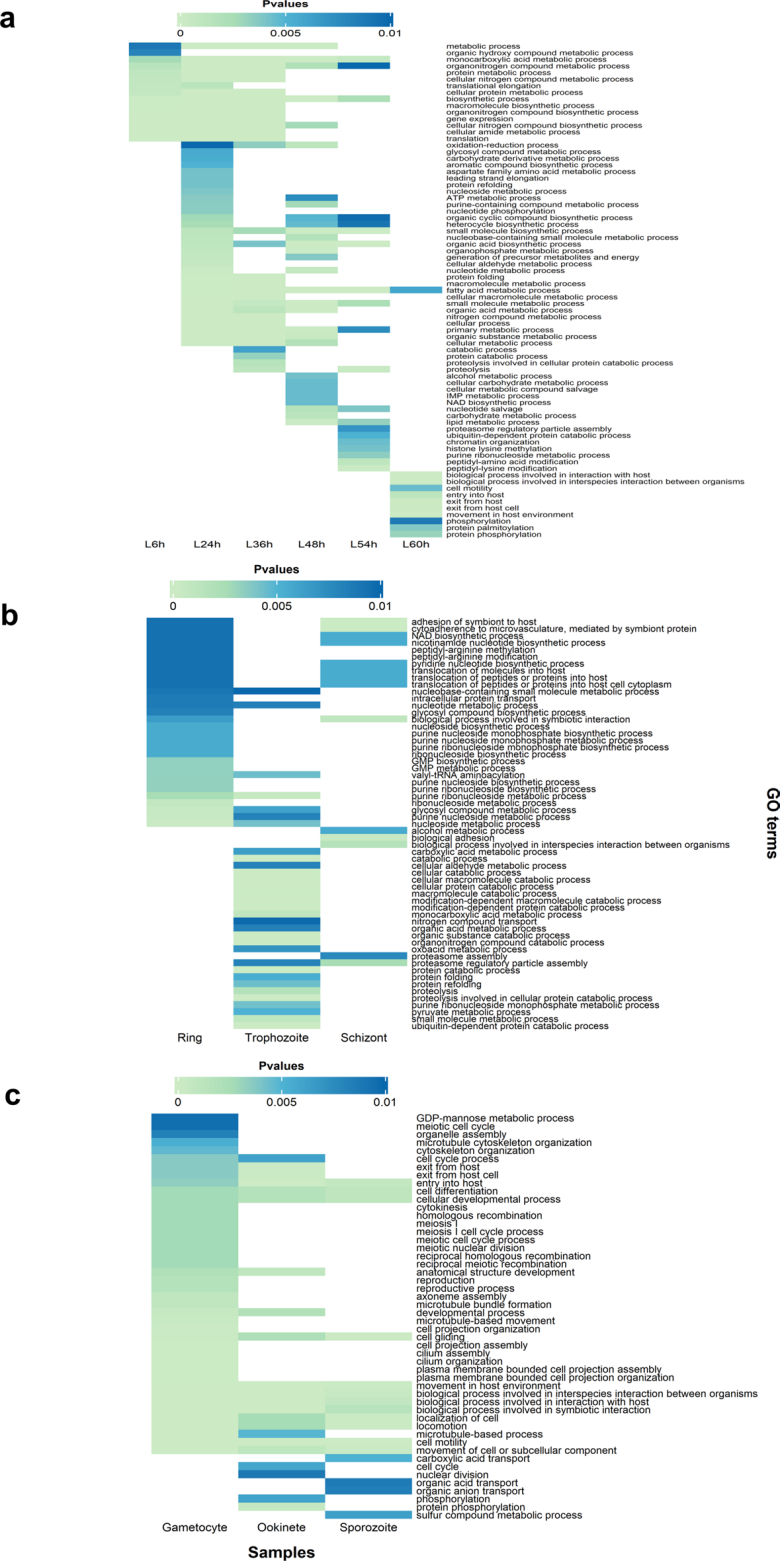
Heatmap of enriched Gene Ontology terms for biological processes associated with *P. berghei* DEGs in (**a**) liver stages, (**b**) asexual blood stages, and (**c**) sexual and transmission stages (p value < 0.01).

Gene ontology terms that describe the related biological processes of the intersection of upregulated genes at each developmental stage were identified. Several GO terms were found to be shared across comparable phases, suggesting similar functional themes. Among the biological processes enriched in the liver stages were metabolic processes (such as fatty acid metabolism), “translation”, “gene expression”, “oxidation‒reduction process” and “interaction with host” (Fig. 3A). Genes encoding *PK2, pdhA, and ACC*, among other enzymes involved in the type II fatty acid synthesis (FASII) pathway, and genes encoding Fab proteins (*FabB/FabF, FabZ, FabG*, and *FabI*) were notably upregulated (Supplementary Table S3). Additionally, there was an upregulation of genes encoding enzymes involved in lipid precursor synthesis (*G3PDH, G3PAT*), lipoic acid synthesis (*lipA, libB*) and acyl-CoA synthetase (*ACS*), which are involved in fatty acid transport. Furthermore, the liver-specific genes *LISP1* and *LISP2* which are known for their functions in the maturation of late liver stages and export to host liver cells are upregulated^18^.

The ring and trophozoite blood stages which show no significant differences in gene expression compared with the early and mid-liver stages respectively, were similarly associated with GO terms related to asexual growth. In addition to metabolic processes, some notable terms enriched in the blood stages were “translocation of peptides or proteins into the host cell cytoplasm” and “cytoadherence to microvasculature” (Fig. 3B). The genes that were found to be upregulated included those that encode proteins involved in invasion such as SERA proteins (*SERA3, SERA4*), Rhoptry genes (*RhopH2, RhopH3*) and merozoite surface proteins (MSPs), such as *MSP2* and *MSP7*. Throughout the stages, there was a constant upregulation of genes from the lysine-rich small, exported protein/early transcribed membrane protein families (*SEP/ETRAMP*) and multigene families of exported proteins including PIR, fam-a, and fam-b proteins (Supplementary Table S3). Furthermore, the biological processes related to symbiotic or interspecies interactions with the host were enriched in the schizonts (Fig. 3B).

Genes involved in sexual reproduction and the egress of parasites from host cells were upregulated during the gametocyte stage. These genes were implicated in various activities, including “meiotic cell cycle”, “homologous recombination”, “cytokinesis”, “axoneme assembly”, “cytoskeleton organization”, “locomotion”, and “exit from host cell” (Fig. 3C). The upregulated genes included those encoding nuclear fusion proteins (*NEK2* and *NEK4*); surface protein-coding genes such as *P230, P48/45, CCp, P25* and *P28*; the female-specific gene *G377*; the shared gene *MDV1*; and male-specific genes such as guanyl cyclase (*GC*), phospholipase C, *CDPK4*, *MAPK2*, and *HAP2* (Supplementary Table S3).

The upregulated genes in the mosquito stages were enriched in biological processes, such as “cell differentiation”, “cellular developmental process”, “entry into the host”, “cell gliding”, “movement in the host environment”, and biological processes involving symbiotic or interspecies interactions (Fig. 3C). In particular, there was an upregulation of genes encoding invasion proteins, including TRAP proteins, TRAP-related proteins, *CSP*, and genes encoding cell traversal proteins, including *PL*, *SPECT1*, and *CelTOS* (Supplementary Table S3).

### Coexpression clustering analysis reveals functionally enriched clusters

Notably, co-expression clustering analysis also split the parasite stages into two clusters, one containing gametocytes, ookinetes, and sporozoites and the other containing liver stages and asexual blood stages, similarly to the correlation analysis. A variety of clustering techniques and algorithms were used to find the optimal number of clusters within the DEGs. Three clusters received the highest“vote”according to the majority rule for determining the optimal number of clusters (Supplementary Figure S1). For this reason, clustering was performed with k = 3. The GO enrichment tool from PlasmoDB (version 51) was used to extract the biological processes connected with each cluster to better understand them and then these terms were summarized using REVIGO (Supplementary Table S8).

**Fig. 4:**
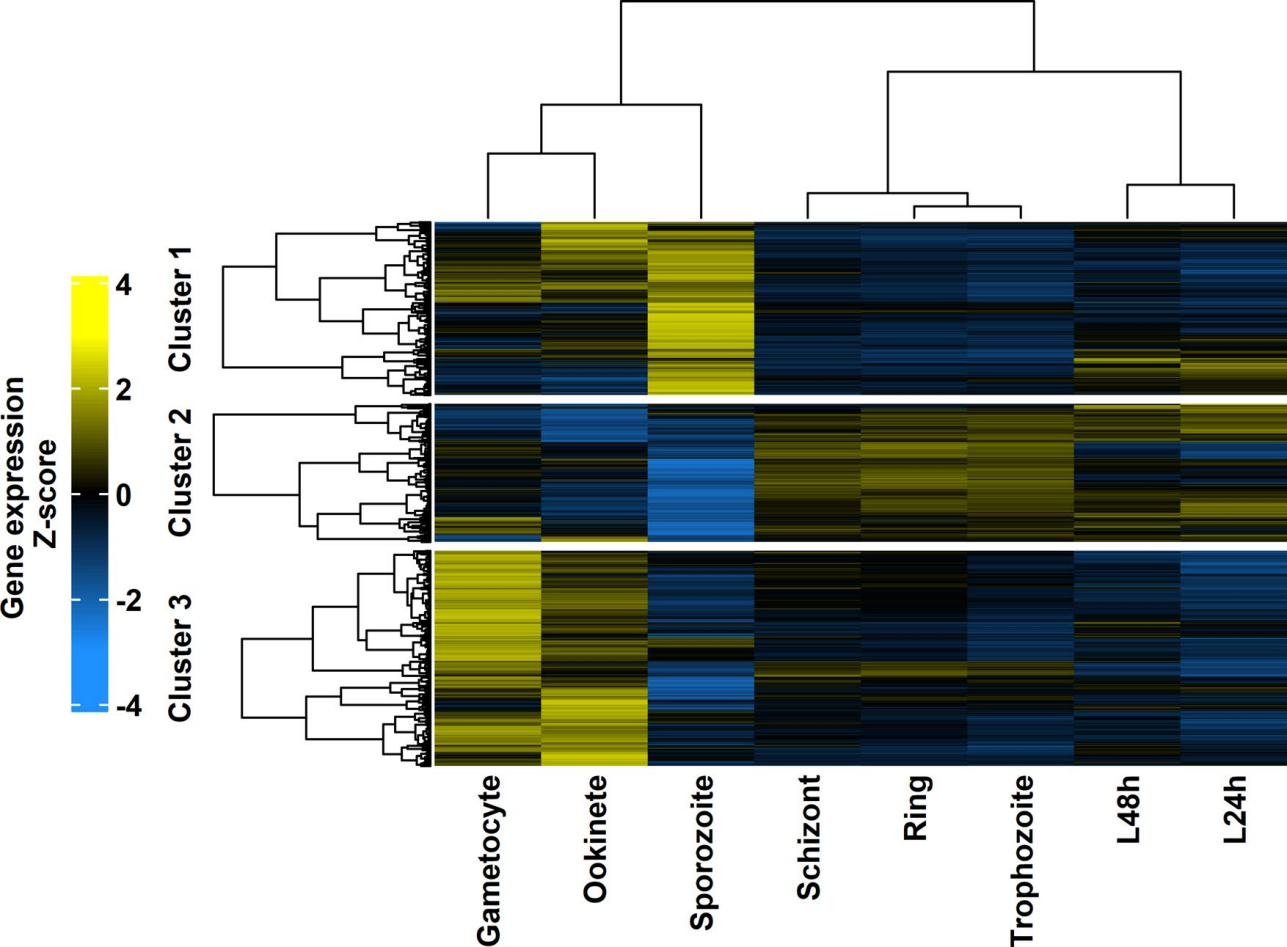
Heatmap of RNA-Seq-based average gene expression values across replicates, scaled by z-transformation. A total of 1832 genes (rows) are shown across the life cycle stages (columns). Clusters from PAM with k = 3 are shown as colour bars on the right-hand side. The dendrograms on the left-hand side show the within-cluster and cluster-to-cluster relationships of the PAM clusters, while the dendrograms on the top of the heatmap show the relationships among the stages.

Cluster 1 included 629 genes (Supplementary Table S4) that were typically downregulated in the liver and asexual blood stages and mostly upregulated in the sporozoite stage and partially upregulated in the gametocyte and ookinete stages (Fig. 4). Biological processes associated with migration in the host environment and interspecies interactions, which are compatible with being ready to infect a new host, were strongly enriched in this cluster (Supplementary Table S5). A total of 536 genes (Supplementary Table S4) from Cluster 2 were downregulated in the sporozoite and ookinete stages, upregulated in the liver and asexual blood stages and partially upregulated in the gametocytes (Fig. 4). The metabolism of various biochemical components, gene expression, proteolysis and biological adhesion to the host were among the biological activities that were substantially enriched (Supplementary Table S5). A total of 667 genes (Supplementary Table S4) whose expression was downregulated in the sporozoite, liver, and asexual blood stages and upregulated in the gametocyte and ookinete stages were found in Cluster 3 (Fig. 4). Many biological processes related to cell motility, microtubule-based activities and host interactions were enriched similarly to Cluster 1 as the parasite transitions from a non-motile to a motile state and it is ready to move from the vertebrate host to a mosquito vector (Supplementary Table S5).

### De novo motif discovery

**Fig. 5:**
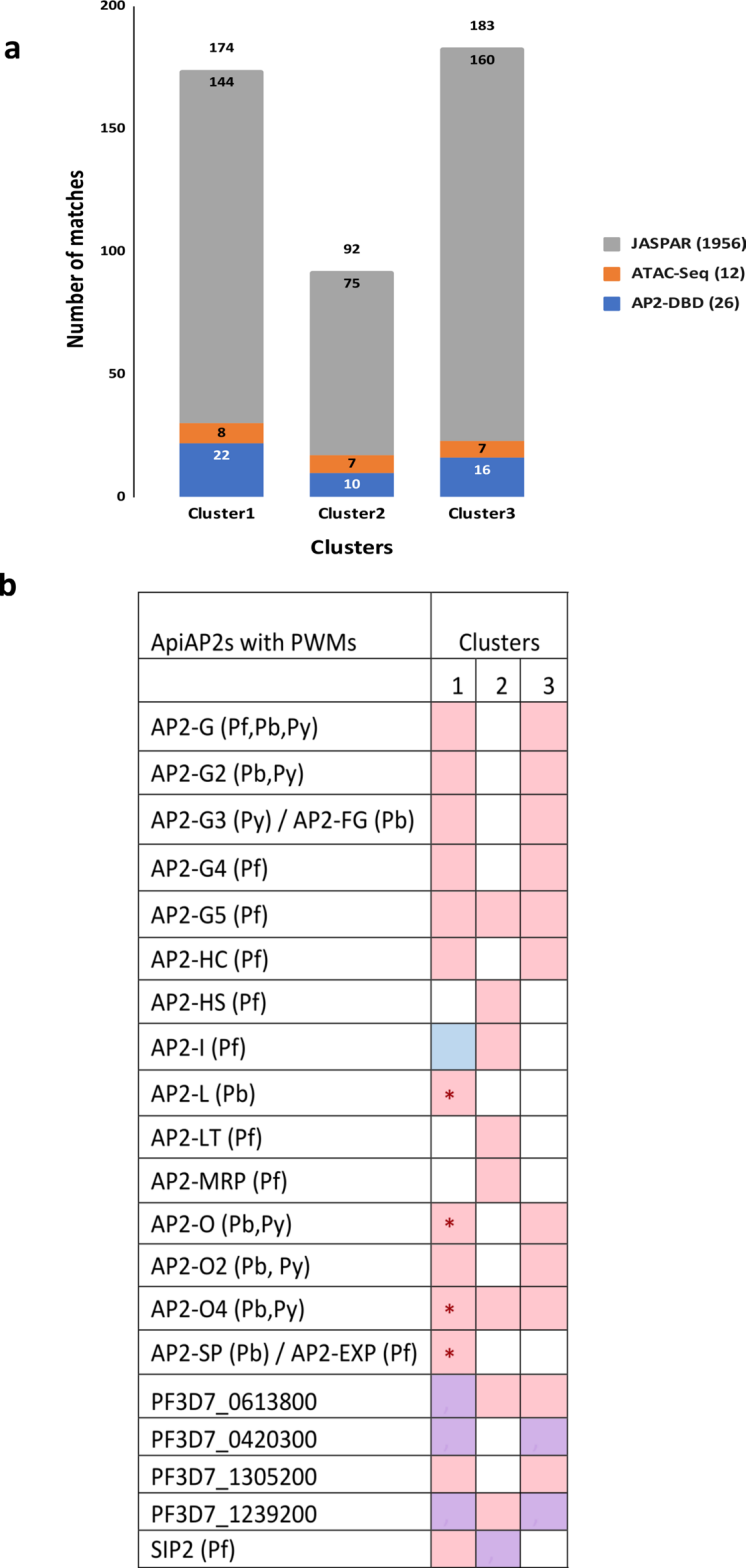
Similarities of the discovered root motifs to known motifs. (**a**) Comparison of the discovered motifs from each PAM cluster to known eukaryotic motifs, including those from *Plasmodium* genomes. Similarities were computed based on Euclidean distances. The total number of motifs in each database of known motifs is shown in brackets. Each stack of the bars corresponds to the number of known motifs in the respective collection that had hits to the discovered motifs in the cluster. The total number of matching motifs found for each cluster is represented by the number on top of the stacked bar. (**b**) Comparison of the discovered motifs from each PAM cluster to known motifs of *P. falciparum* AP2 domains with position weight matrices. Clusters = PAM coexpression clusters; Colours = similarity of the discovered motif to known AP2 binding sites detected: pink = motif similar to the binding site of the first AP2 domain, blue = motif similar to the binding site of the second AP2 domain, purple = motif similar to the binding site of both AP2 domains; * motif similarity detected in the same stage AP2 TF is expressed.

Specific regulatory sequence motifs are associated with the dynamics of transcription. To identify the resident putative regulatory sequences upstream of the genes showing the observed co-expression patterns, we searched the promoter regions defined as the 5’ UTR 1000 bp upstream of the start codon of the genes in each coexpression cluster for over-representation of short sequences using two de novo motif discovery tools from RSAT^38^ and MEME^39^. It is also typical to employ several de novo motif discovery approaches concurrently, enabling one to take advantage of their complementary qualities. Each tool identified a collection of motifs in the coexpression clusters, which were then clustered to remove the redundancy from both tools and identify the representative root motif for each motif cluster. Redundant motifs are frequently found when high-throughput datasets, such as those from ChIP-Seq or RNA-Seq, are analysed (Fig. 5A). Furthermore, the majority of motifs will be independently found by multiple tools, although some motifs may be identified solely by one tool. This means that redundant motifs with slight variations in length and/or nucleotide frequencies at specific positions may arise, and this redundancy can be reduced by employing motif clustering^52^ (Supplementary Data S1). Similarities between the discovered root motifs and those in JASPAR, a sizable collection of recognised, nonredundant motifs from plants, animals, nematodes, fungi, and insects, were detected. JASPAR contains binding motifs for a number of transcription factor (TF) types, including nuclear receptors, zinc fingers, bZips, helix turn helices, forkheads, POU/Homeodomains, and Myb proteins. Notably, the majority of the root motifs in all of the clusters matched the known DNA binding patterns of the ApiAP2 TFs which are important regulators of *Plasmodium* gene transcription and were initially found in *P. falciparum* via PBM experiments^6^ as well as motif predicted by in silico studies (Supplementary Table S6)^20,21^. In addition, the majority of the motifs (Fig. 5A) exhibited similarities to de novo motifs predicted by an ATAC-Seq analysis^41^. Unrelated TFs also exhibit similar patterns^52^, which explains why a particular motif could resemble one or more binding sites for TFs in the ApiAP2, ATAC-Seq, and JASPAR motif collections (Fig. 5A, Supplementary Data S2).

AP2 binding domains are often named based on the direct transcriptional activator roles they play at the relevant developmental stage (reviewed in^53^. Consequently, parallels to well-known AP2 binding domains with the underlying expression profiles of the genes in the corresponding coexpression clusters were observed (Fig. 5B). Many studies have been conducted on the transcriptional regulation of gene expression, especially in *P. berghei* and *P. falciparum,* during the IDC and mosquito phases (Table S1). While the majority of the AP2 binding domains have well-established biological roles in regulating the transitions between distinct developmental stages, the functions of other AP2 binding domains remain unknown. The majority of AP2 factors are essential for pathogenesis, optimal morphology and maturation, host cell invasion, sexual commitment, and sex differentiation processes^53^. The majority of the *P. berghei* root motifs in each main cluster showed redundancy in their resemblance to the motifs predicted in *P. falciparum* by ATAC-Seq, the binding sites of the *P. falciparum* AP2 TFs, and the known eukaryotic motifs in JASPAR. Similar motifs for the well-known transcriptional activator of sporozoites, AP2-SP, as well as other gametocyte- and ookinete-relevant AP2 binding domains, such as AP2-G, AP2-G2, AP2-FG, AP2-G4, AP2-G5, AP2-O, AP2-O2, and AP2-O4, were identified in Cluster 1, which contains genes highly expressed in sporozoites and with lower expression in gametocytes and ookinetes (Supplementary Data S2). A motif resembling the binding site of *Pf*AP2-I, the TF that orchestrates red blood cell invasion, was found among the identified motifs in Cluster 2, which is primarily composed of genes related to the asexual stage with a small number of genes related to gametocytes and ookinetes (Supplementary Data S2). Other motifs included AP2-O4 and *Pf*AP2-G5. Similar patterns for important AP2 TFs, such as AP2-G, AP2-G2, AP2-FG, AP2-G4, *Pf*AP2-G5, AP2-O, AP2-O2, and AP2-O4, were found in Cluster 3 which primarily contains genes related to gametocytes and ookinetes (Supplementary Data S2). *Pf*SIP2 (Clusters 1 and 2), *Pf*AP2-HS (Cluster 2), *Pf*AP2-HC (Clusters 1 and 3) (Supplementary Data S2) and uncharacterized AP2 binding domains (Fig. 5B) are additional motifs for AP2 factors that demonstrate similarities to the identified motifs. The discovery of de novo motifs in coexpression clusters that resemble uncharacterized AP2 binding domains suggested that these clusters may be under their regulation (Fig. 5B). These uncharacterized AP2 binding domains are resistant to disruption in the blood stages^51^, which makes it more difficult for functional genomics research to identify their associated phenotypes. To distinguish between biologically significant and off-target binding similarities, the similarities between the motifs in the clusters and the uncharacterized AP2 binding domains were evaluated in light of their documented interactions with other proteins in the literature. There are currently few studies indicating potential functions for these AP2 binding domains. Notably, a study that examined the trophozoite and schizont stages employing blue-native PAGE combined with quantitative mass spectrometry and machine learning revealed that in *P. falciparum*, the *Pf*MORC complex is associated with ApiAP2 proteins, comprising three uncharacterized ApiAP2 proteins (PF3D7_0420300/PBANKA_0521700, PF3D7_1239200/PBANKA_1453700, and PF3D7_0613800/PBANKA_0112100); three characterised ApiAP2 proteins (*Pf*AP2-I/PF3D7_1007700/PBANKA_1205900, *Pf*AP2-MRP/PF3D7_1107800/PBANKA_0939100, AP2-O5/PF3D7_1449500/PBANKA_1313200); and machinery for chromatin remodelling^54^. Using targeted immunoprecipitation with LC‒MS/MS proteomic quantification during asexual blood stage development in *P. falciparum*, the associations of the ApiAP2 proteins with the *Pf* MORC complex were also validated in another experiment^55^. The study identified six ApiAP2 proteins previously mentioned, as well as *Pf*AP2-G5/PF3D7_1139300/PBANKA_0909600. Additionally, using a yeast two-hybrid assay of *P. falciparum*-infected erythrocytes, PF3D7_0420300 and PF3D7_1007700 were also previously linked to histone acetylation since they form a complex with the histone acetyltransferase GCN5, as well as the characterised *Pf*AP2-LT/PF3D7_0802100/PBANKA_1228100^56^. Because there are insufficient ChIP-Seq investigations that explicitly target ApiAP2 proteins, it is currently unknown which of the multiple-domain AP2 binding domains are involved in these interactions. For instance, each of the three AP2 domains of PF3D7_1007700 binds a different sequence, making it challenging to consistently correlate the other two AP2 domains with these protein complexes, as only the third AP2 domain of PF3D7_1007700 has been characterised. Together, these findings support the notion that the motifs resembling the binding sites of the unidentified ApiAP2 proteins in Cluster 2 may have functional roles.

### Further analysis of selected motifs

**Fig. 6:**
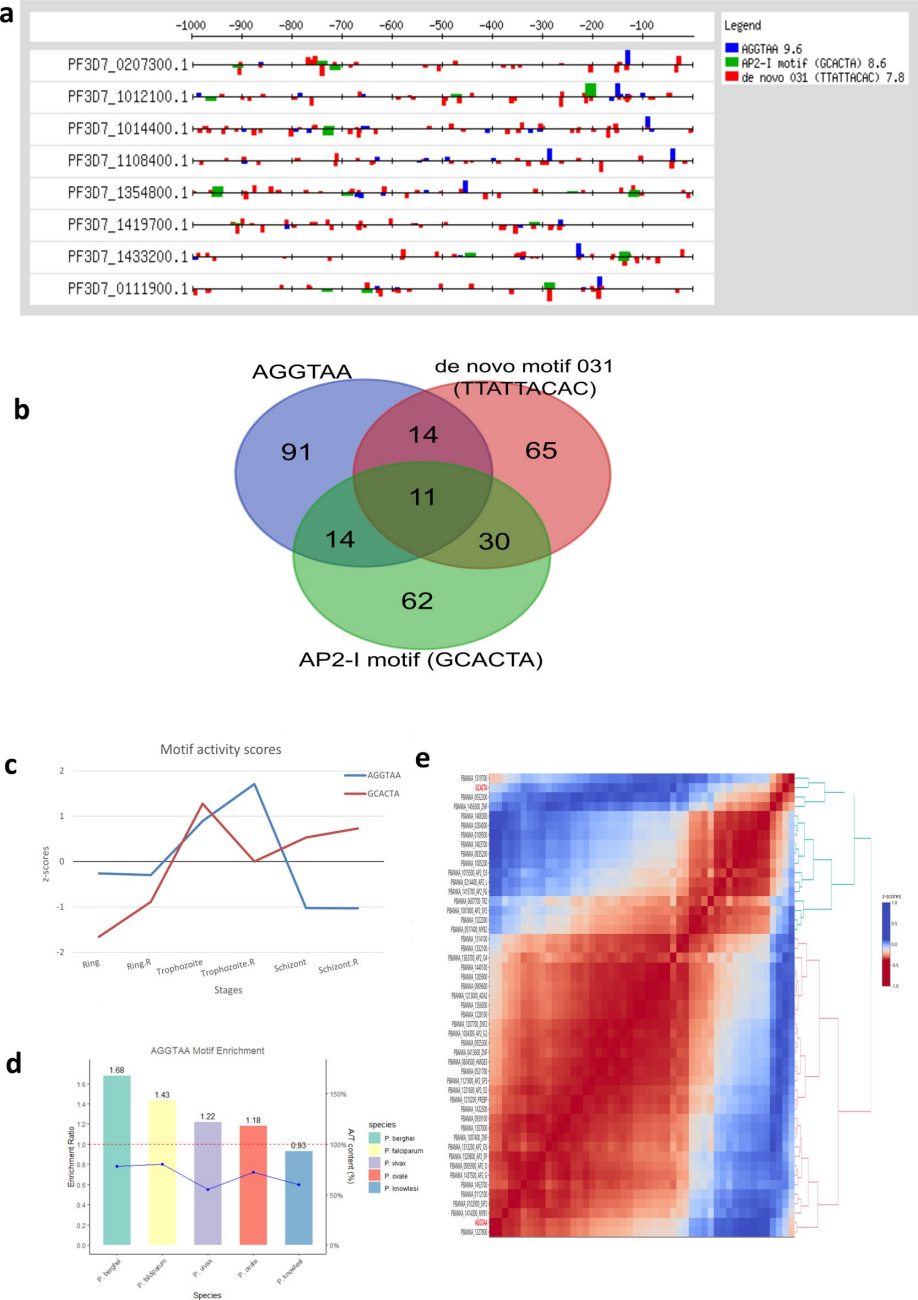
A de novo motif likely coregulated with the *Pf*AP2-I-related motif and prediction of its interactors. (**a**) Promoter architecture of some of the common target genes of the AGGTAA motif and the de novo motif 031 in *P. falciparum*. The promoter region of each gene represents its entire 5’UTR upstream of the start codon, and the bars represent the genomic locations of each instance of a motif. The AGGTAA motif is represented in blue, while the de novo motif 031 is represented in red. Overlapping instances of both motifs in opposite directions can be seen as expected for complementary sequences. (**b**) Relationships among the AGGTAA, de novo motif 031 and *Pf*AP2-I motifs in *P. berghei* showing the intersection of target genes harbouring the motifs. (**c**) Activities of the AGGTAA and GCACTA motifs in *P. berghei* as inferred by MARA^38^. The motif activities are scaled by z-transformation. The activity of the AGGTAA motif is represented by the blue line, while that of the GCACTA motif is represented by the red line. The motif activities are shown during the ring, trophozoite and schizont stages of asexual blood stage development. R represents biological replicates. (**d**) Enrichment ratios of the AGGTAA motif in *P. berghei* genes and their orthologs, please note that label P. ovale is referring to P. ovale curtisi GH01. (**e**) Heatmap of the cross-correlation of inferred motif activity scores for the AGGTAA and GCACTA motifs and the expression values of known transcription factors and coactivators in *P. berghei*, scaled by z-transformation. The rows represent the motifs and regulatory proteins. The AGGTAA and GCACTA motifs are highlighted in red.

The fifth and seventh root motifs within Cluster 2 which included *P. berghei* motifs with putative regulatory roles during asexual development did not resemble any of the known ApiAP2 motifs or the de novo motifs predicted by ATAC-Seq in a different study to be enriched in accessible chromatin regions of the *P. falciparum* genome during the IDC (Supplementary Data S1 and S2). This finding was made through motif comparison analysis using the TOMTOM tool^39^. Nevertheless, additional investigations revealed that the fifth motif, [A/T][A/T]AGGTAA[A/T][A/T] or simply AGGTAA might be related to de novo motif 031, one of the ATAC-Seq-predicted motifs. TTATTACAC is the binding sequence for de novo motif 031. The ATAC peaks localised to a variety of locations in the *P. falciparum* genome including promoters, exons, and intergenic regions; hence, only the ATAC-Seq peak regions that mapped exactly to 5’ UTRs were desired. The regions where the 5’ UTRs and the ATAC peaks overlapped were subsequently extracted. Using the RSAT"matrix-scan"tool^38^ the individual genomic coordinates for each occurrence of the AGGTAA motif and the de novo motif 031 within the accessible chromatin 5’UTRs were extracted, revealing that both motifs shared some genomic locations (Fig. 6A). The predicted motifs in the same cluster as the de novo motif 031 (TTATTACAC)^41^ included the motif for *Pf*AP2-I (PF3D7_1007700/PBANKA_1205900), a well-characterized ApiAP2 TF whose third AP2 domain contains the binding sequence GTGCACTA. During asexual development, *Pf*AP2-I orchestrates the invasion of red blood cells^57^. In our analysis, the motif for *Pf*AP2-I was validated by the motif comparison tool TOMTOM^39^ to be comparable to the [A/T][A/T]GCACTA[A/T][A/T] motif, or GCACTA for short. This motif appears in the same cluster as the [A/T][A/T]AGGTAA[A/T][A/T] motif which has a reverse complement TTTTACCTTT. This finding implies that the TFs that bind both motifs probably coregulate the target genes that harbour them. Overall, the connections between *Pf*AP2-I, AGGTAA, and de novo motif 031 were investigated through the construction of a gene regulatory network (Supplementary Table S7). Using the"matrix-scan"tool, 287 target genes from Cluster 2 that harboured the three motifs were extracted. Fifty-eight genes (≈20%) had binding sites for two of the motifs, whereas 218 genes (≈76%) had binding sites for only one of the motifs. Among all the genes, only 11 (approximately 4%) had binding sites for all three motifs (Fig. 6B). According to the motif activity inference analysis, the GCACTA motif is active throughout the schizont and trophozoite stages and inactive during the ring stage, whereas the AGGTAA motif is active during the trophozoite stage (Fig. 6C). Notably, the AGGTAA motif is enriched across *Plasmodium* species, except in *P. knowlesi*, with enrichment scores ranging from 1.18 to 1.68, indicating a consistent pattern of enrichment though with some variation in degree. In particular, higher enrichment was observed in the more A/T rich species like *P. berghei* and *P. falciparum* in comparison with the species with more diverged G/C content (*P. vivax* and *P. ovale* (Fig. 6D, Supplementary Data S3). Thirty of the forty-seven TFs that were examined during the cross-correlation analysis were found to correlate well with the AGGTAA motif, and the rest were found to correlate with the GCACTA motif. Remarkably, the well-characterized *Pf*ApiAP2-I (PF3D7_1007700/PBANKA_1205900) was among the potential interactors of the AGGTAA motif revealed by cross-correlation (Fig. 6E).

### Discovery of motifs in orthologous protein-coding genes

The total number of differentially expressed genes (DEGs) after excluding the initial cohort of 1,827 DEGs was 521 orthologous protein-coding genes which proceeded to the coexpression clustering step. The clustering output clarifies the DEGs’ organization into specific clusters and their respective subclusters. The sub-clustering analysis revealed the following clusters:

- Cluster 1: 138 orthologous genes, subdivided into Cluster 1_sub1 (n = 19), Cluster 1_sub2 (n = 27), Cluster 1_sub3 (n = 37), and Cluster 1_sub4 (n = 55).
- Cluster 2: 298 orthologous genes, subdivided into Cluster 2_sub1 (n = 95), Cluster 2_sub2 (n = 37), Cluster 2_sub3 (n = 58), Cluster 2_sub4 (n = 41), and Cluster 2_sub5 (n = 67).
- Cluster 3: 85 orthologous genes, subdivided into: Cluster 3_sub1 (n = 19), Cluster 3_sub2 (n = 16), Cluster 3_sub3 (n = 19), Cluster 3_sub4 (n = 15), Cluster 3_sub5 (n = 5), and Cluster 3_sub6 (n = 11).

Regarding motif discovery based on the MEME Suite, we identified a total of 66 motif targets. Notably, 52 of these targets align with our findings from a previous analysis where we included all differentially expressed genes (DEGs) including non-dominant and duplicated genes. Additionally, 14 motif target IDs emerged as unique to the orthologous protein-coding genes, highlighting new aspects of the supplementary analysis of these genes.

We thoroughly examined the functional classes associated with motif target IDs beyond merely comparing them. Using the JASPAR2022 and TFBSTools R packages, we found that the motif target IDs linked to orthologous protein-coding genes can be categorized into 11 distinct functional classes. Among these classes, seven classes demonstrated robustness consistently showing motif target IDs shared with the primary analysis that included all differentially expressed genes (DEGs). Additionally, we identified two specific motif classes—Basic Leucine Zipper (bZIP) factors and GCM domain factors—that were exclusively reported concerning the motif target IDs of orthologous protein-coding genes. Conversely, two other classes, TATA-binding proteins and C4 zinc finger-type factors were found only in the overlap of motif target IDs identified in the primary analysis of all DEGs and the supplementary analysis focused solely on orthologous genes. Our comprehensive analysis of motifs using the Matrix-Scan tool revealed a total of 37,485 occurrences of the AGGTAA motif. These instances were distributed across several distinct clusters with each demonstrating varying frequencies: Cluster1_sub1 (n = 894), Cluster1_sub2 (n = 924), Cluster1_sub3 (n = 2378), Cluster1_sub4 (n = 3858), Cluster2_sub1 (n = 9200), Cluster2_sub2 (n = 3137), Cluster2_sub3 (n = 5176), Cluster2_sub4 (n = 3620), Cluster2_sub5 (n = 5170), Cluster3_sub1 (n = 616), Cluster3_sub2 (n = 1038), Cluster3_sub3 (n = 658), Cluster3_sub4 (n = 198), Cluster3_sub5 (n = 108) and Cluster3_sub6 (n = 510). The marked diversity in motif frequency across these clusters highlights the nuanced distribution patterns observed in the analyzed data. In addition, we recorded 14,233 occurrences of the GCACTA motif which displayed a different distribution across the clusters: Cluster1_sub1 (n = 447), Cluster1_sub2 (n = 792), Cluster1_sub3 (n = 820), Cluster1_sub4 (n = 1975), Cluster2_sub1 (n = 2385), Cluster2_sub2 (n = 404), Cluster2_sub3 (n = 1973), Cluster2_sub4 (n = 1220), Cluster2_sub5 (n = 2388), Cluster3_sub1 (n = 616), Cluster3_sub2 (n = 519), Cluster3_sub3 (n = 94), Cluster3_sub4 (n = 396), Cluster3_sub5 (n = 0) and Cluster3_sub6 (n = 204). The diverse spread of occurrences for the GCACTA motif also underscores the presence of clusters with both rich and sparse motif distributions. For a deeper exploration of the detailed results surrounding the discovery of motifs in orthologous protein-coding genes, please refer to Supplementary Data S5: Supplementary File S5.zip.

### Identification of motifs in ChIP-seq signals related to orthologous protein-coding genes.

To conduct motif discovery analysis using ChIP-seq signals, we extracted a total of 367 signals which were distributed as follows: 44 signals associated with orthologous genes at the Ring stage, 109 signals linked to orthologous genes at the Schizont stage, and 214 signals associated with orthologous genes at the Trophozoite stage. In motif discovery using the MEME Suite, the distribution of sequence motifs is as follows: 40 motif targets were identified in the Ring stage, 88 in the Trophozoite stage, and 42 in the Schizont stage. 15 target IDs overlap across all the stages. Additionally, three motif target IDs overlap between the Ring and Trophozoite stages and 10 target IDs overlap between the Trophozoite and Schizont stages. However, no overlapping target IDs were found exclusively between the Schizont and Ring stages.

When analyzing the overlap of target IDs found in orthologous protein-coding genes, we discovered that 39 out of the 52 identified target IDs overlap with at least one developmental stage. The overlaps are summarized as follows: 4 motif target IDs overlap across all the analyses, 26 motif target IDs overlap with orthologous protein-coding genes and ChIP-Seq signals in the Schizont stage, 36 motif target IDs overlap in the Trophozoite stage and 15 motif IDs overlap in the Ring stage. In contrast, no overlap exists between the 14 motif target IDs exclusively identified in the supplementary analysis and the ChIP-Seq signals.

Regarding the motif classes based on JASPAR2022 and TFBSTools annotations, the motifs identified in the chip-seq signals belong to 18 distinct motif target classes. They include C2H2 zinc finger factors, RING-type zinc finger, TATA-binding proteins, A.T hook factors, Heat shock factors, Basic leucine zipper factors (bZIP), Tryptophan cluster factors, Basic helix-loop-helix factors (bHLH), Other C4 zinc finger-type factors, Fork head/winged helix factors, NDT80 domain factors, APSES-type DNA-binding domain, MADS-box factors, Homeo domain factors, Heteromeric CCAAT-binding factors, AP2/EREBP, GCM domain factors and High-mobility group (HMG) domain factors. Two of these 18 distinct motif target classes are identified exclusively in the ring stage:"A.T. hook factors“and”heat shock factors."Two motif target classes are recognized solely in the schizont stage: the APSES-type DNA-binding domain and NDT80 domain factors. Additionally, four motif target classes are identified exclusively in the trophozoite stage: AP2/EREBP factors, GCM domain factors, heteromeric CCAAT-binding factors and high-mobility group (HMG) domain factors. Regarding overlaps with motif classes identified based on orthologous protein-coding genes using bulk RNA-seq, this method identified one motif class exclusively in orthologous protein-coding genes which is the Rel homology region (RHR) factors. Meanwhile, eight motif classes are solely identified in the ChIP-seq signals of orthologous protein-coding genes which are RING-type zinc finger factors, A.T. hook factors, heat shock factors, basic helix-loop-helix factors (bHLH), forkhead/winged helix factors, NDT80 domain factors, APSES-type DNA-binding domain factors and heteromeric CCAAT-binding factors.

In the analysis of motifs using the Matrix-Scan tool linked to Chip-seq data for orthologous protein-coding genes, a total of 11,281 hits for the GCACTA motif were identified and distributed as follows: 478 hits in the ring stage, 5,184 hits in the schizont stage and 5,619 hits in the trophozoite stage. Additionally, 46,513 hits for the AGGTAA motif were identified and distributed as follows: 2,754 hits in the ring stage, 18,747 hits in the schizont stage and 25,012 hits in the trophozoite stage. For a deeper exploration of the detailed results surrounding the discovery of motifs in orthologous protein-coding genes using chip-seq data, please refer to Supplementary Data S6: Supplementary File S6.zip.

## Discussion

The activity of the AGGTAA motif in the trophozoite stage corresponded better with the expression of the uncharacterized *P. berghei* orthologue for *Pf*AP2- I, PBANKA_1205900, in the ring stage than with the activity of its related binding sequence, GCACTA, in the trophozoite and schizont stages (Fig. 6C and 6E). This could be explained by the possibility that *Pf*AP2-I binds to its target gene promoters, including its promoter at the trophozoite stage, and remains associated in schizonts. It has been demonstrated by live microscopy and nuclear fractionation assays that *Pf*AP2-I-GFP localises exclusively to the nucleus of the trophozoite and schizont stages^57^. On the other hand, RNA-Seq revealed that PBANKA_1205900 is expressed as early as the ring stage and peaks in the schizont stage^9^. This suggests that PBANKA_1205900 may interact with other proteins, such as the TF for the AGGTAA motif, after it is expressed in the early ring stage, before binding to its targets in the later stages. The association between trophozoite-stage AGGTAA motif activity and early expression of the uncharacterized PBANKA_0521700 was another significant but expected finding. It has been demonstrated that PBANKA_0521700 is expressed preferentially in the ring stage^9,51^. Interestingly, cross-correlation analysis also revealed a strong association between AGGTAA motif activity and the expression of members of the *Pf*MORC and GCN5 complexes, suggesting a potential interaction between chromatin-associated proteins and the TF that recognises the AGGTAA motif (Fig. 6E). Notably, DNA pull-down experiments in *P. falciparum* have demonstrated that the related motif, de novo motif 031, interacts with the second AP2 domain of the uncharacterized ApiAP2, PF3D7_0420300/PBANKA_0521700^41^ which is also a member of both protein complexes.

Despite having similar life cycles and biological characteristics, *Plasmodium* species differ greatly in their genetic makeup. These genetic and regulatory differences can be linked to evolutionary adaptations within the species. The variations in the genomes and regulatory components of *P. berghei* and *P. falciparum* are caused by various factors which include host specificity, transmission dynamics, and distinctive features of each species’ life cycle. These variations could manifest as different patterns of gene expression, species-specific TF involvement, or motifs. As such, interpretations of species-specific mechanisms should be made cautiously. In comparison to other eukaryotes, *Plasmodium* parasites lack a lot of TFs. To compensate for this, *Plasmodium* parasites have also developed some characteristics^58^. Research points to a potentially limited role for the ApiAP2 TFs in controlling erythrocytic transcription especially during the asexual phases. This suggests that while the interaction of AP2 binding domains with specific promoters can lead to either repression or activation of gene expression, it is dependent on the presence of many chromatin-associated proteins (apart from the few AP2 binding domains with additional chromatin-related functions) and a favourable epigenetic environment both of which aid in the transcription process^59^. Consequently, studies involving a more thorough examination of the interactions between all the mechanisms would contribute to our knowledge of holistic gene regulation in *Plasmodium*.

In addition to providing insightful information about transcriptional regulation throughout the life cycle of *P. berghei*, this study revealed patterns of gene transcription at different stages of development. Based on the different expression profiles of the DEGs across the *P. berghei* life cycle, unique clusters were identified. Additionally, a thorough examination of the promoter regions of the genes in each cluster with similar expression profiles was performed to identify shared DNA binding motifs that would aid in our continued comprehension of transcriptional regulation in *P. berghei.* A limitation of our study is the absence of filtering of divergent multicopy genes and non-protein-coding genes prior to unsupervised clustering which could influence the clustering outcomes and subsequent motif discovery. While our results provide valuable insights into *Plasmodium* life cycle progression, future analyses should include such filtering to further enhance the robustness and interpretability of the findings. The de novo motifs were interpreted in light of the purported or reported roles of the well-known AP2 domains at various life cycle stages. Furthermore, one motif, AGGTAA, was identified to be similar to a newly identified motif in *P. falciparum* whose function is not well understood. Extending the annotation of this de novo motif suggested its possible association with chromatin-associated proteins during erythrocytic development. Methodologies employed in motif discovery can significantly impact the results obtained. Hence, our finding of the AGGTAA motif may be attributed to our methodological limitations or biases inherent in our approaches and its biological relevance should therefore be considered in light of these limitations. Moreover, not all binding specificities for all ApiAP2 TFs have been resolved. Therefore, it is unclear whether the AGGTAA motif discovered in this study is, in fact, a genuine binding site that has not yet been identified in vivo. Hence, to demonstrate its functional significance for gene regulation throughout the parasite’s life cycle, experimental validation is valuable. GFP and luciferase reporter assays have previously been used to successfully demonstrate the stage-specific activity of a promoter harbouring ApiAP2 TFs, allowing both exact quantitative analysis and qualitative analysis^60^. For example, site- directed mutagenesis was used to truncate one of the four predicted binding sites for *Pf*AP2-G5 by introducing a mutation inside its sequence in the *PbLISP2* promoter. The mutation resulted in a significant increase in luciferase activity, suggesting that the orthologue of *Pf*AP2-G5 acts as a repressor and showing that one copy of a binding site was sufficient to impact transcription. It is interesting to note that the mutation did not elevate luciferase levels in other parasite stages maintaining the *PbLISP2* transcription profile specifically to the liver stage^60^.

The transcriptome specific to each stage, obtained from differential gene expression analysis, is a highly significant resource for the application of systems biology techniques to predict metabolic pathways important to different stages of the *P. berghei* life cycle. The data from the co-expression clustering may be helpful in creating multistage vaccination plans that depend on predetermined expression patterns. The underlying regulatory elements governing these expression patterns can be used in the creation of attenuated parasites via targeted mutagenesis employing genetic engineering techniques such as CRISPR-Cas9 or gene knockout which disrupts or modifies them.

It is important to clarify that our discussion centered on MORC and GCN5 in relation to the AGGTAA sequence motifs. This exploration was prompted by the compelling association demonstrated in Fig. 6-E which highlights a significant connection between AGGTAA sequence motifs and the various Ap2 genes. These genes play a crucial role in transcriptional regulation as they interact with key epigenetic factors like GCN5 and MORC, thereby influencing gene expression and fundamental cellular functions^61^. We conducted a thorough analysis of motif discovery, covering both protein-coding and non-coding genes. It provided an extensive overview of regulatory sequence motifs across various life stages. To enhance the reliability of the predicted sequence motifs, we carried out a supplementary analysis focused exclusively on protein-coding genes. This comprehensive follow-up revealed discrepancies in two critical areas: the number of differentially expressed genes and the sequence content of the identified motifs. These discrepancies are not surprising, considering that the identification of differentially expressed genes depends on library size which changes after the removal of non-coding genes like rRNAs and tRNAs. This filtering, while keeping other analytical parameters constant, naturally affects the results of the differential gene expression analysis. In our initial analysis, we identified a total of 1,832 differentially expressed genes while the follow-up revealed an increased count of 2,087 genes.

When evaluating the robustness of motif discovery through unsupervised clustering, it is crucial to recognize the consequences of filtering out non-coding genes. This includes genes encoding rRNAs and tRNAs, which significantly affect the overall number of relevant genes in the gene expression profile. Consequently, we cannot confidently assert that the unsupervised clusters and enriched motifs in gene promoters retain their stability after this gene filtering as numerous statistically significant non-coding genes may have been excluded in the process. For instance, our analysis of sequence motifs, especially in the context of protein-coding genes, has revealed noteworthy findings. A prominent example is the TATA box motif, an essential DNA sequence intricately located within the promoter regions of various protein-coding genes. This motif is critical for initiating transcription, serving as an important binding site for transcription factors and RNA polymerase, thus influencing gene expression and the overall regulation of cellular activity. Significantly, the inclusion of non-coding genes revealed the AGGTAA motif, a key regulatory sequence. Meanwhile, the TATA box motif, a hallmark promoter for numerous protein-coding genes, was confirmed within the protein-coding gene set. In summary, our latest analysis explores the potential benefits of filtering noncoding genes to enhance the accuracy/correctness of our results. Interestingly, following this, our findings have revealed the presence of TATA box motifs associated with protein-coding genes. The TATA box functions as a crucial promoter element, playing an essential role in the initiation of transcription across various biological systems in both eukaryotic and prokaryotic organisms^62^. This discovery underscores the intricate connections between gene regulation and functionality in numerous life forms. Conversely, the inclusion of noncoding genes has also reported intriguing regulatory sequence motifs, such as AGGTAA.

In our investigation into the robustness of identifying the key functional motifs AGGTAA and GCACTA, we analyzed differentially expressed genes (DEGs) associated with orthologous protein-coding genes. This analysis used bulk RNA sequencing data in combination with chromatin immunoprecipitation sequencing (ChIP-seq) to evaluate protein binding signals. The findings clearly demonstrated the presence of these motifs upstream of the orthologous protein genes, indicating their critical role in the regulatory mechanisms that govern gene expression. This evidence underscores the importance of these motifs in modulating the activity of protein-coding genes, thereby providing valuable insights into the complex processes of gene regulation.

The analysis of overlapping motif classes between orthologous protein-coding differentially expressed genes (DEGs) identified through bulk RNA-sequencing (RNA-seq) and protein-binding signals detected via chromatin immunoprecipitation sequencing (ChIP-seq) reveals significant insights into the regulatory landscape associated with these genes. The majority of identified motifs are closely linked to critical DNA regulatory functions particularly those involving transcription factors and various DNA-binding domains. Specifically, four distinct classes of transcription factor motifs have emerged from this analysis: (i) C2H2 Zinc Finger Factors which are characterized by their ability to bind DNA through zinc ions. These factors play essential roles in gene regulation and often mediating responses to environmental signals. (ii) GCM Domain Factors are crucial for processes such as cellular differentiation and development particularly in the context of neuronal and placental development. (iii) MADS-Box Factors are known for their role in flower development and morphological changes. These transcription factors comprise a significant group involved in regulating plant developmental pathways. (iv) TATA-Binding Proteins are essential for the initiation of transcription in eukaryotic cells. These proteins bind specifically to the TATA box element within promoters, thereby facilitating the recruitment of the transcription machinery. In addition to these transcription factor motifs, three classes of DNA-binding domain motifs were also identified enhancing our understanding of regulatory interactions: (i) Basic Leucine Zipper Factors (bZIP). This group is notable for their dimerization capabilities and their role in mediating cellular responses to stress, hormones, and other signaling molecules. (ii) Forkhead/Winged Helix Factors. These factors are recognized for their involvement in cell growth regulation, development, and metabolism. They often function in both developmental and pathological contexts. (iii) Tryptophan Cluster Factors are characterized by their unique tryptophan residues. They are less understood but are believed to contribute to important regulatory networks. Furthermore, two classes of general DNA regulatory domains have been identified and they play foundational roles in genomic architecture and function: (i) C4 Zinc Finger-Type Factors. are commonly involved in DNA recognition and transcriptional regulation. These proteins are often associated with cell growth and differentiation processes. (ii) High-Mobility Group (HMG) Domain Factors. These non-histone proteins are involved in DNA bending and architectural changes, facilitating complex regulatory interactions and contributing to chromatin remodeling. Together, these findings underscore the intricate interplay between transcriptional regulation and the structural dynamics of DNA, highlighting the importance of comprehensive analyses in understanding gene expression mechanisms at a molecular level.

Finally, it is crucial to highlight that the findings related to our identified motif classes necessitate further experimental analysis for comprehensive validation. This is particularly pertinent given the potential discrepancies observed with prior research concerning the classification of these motifs across different Plasmodium species. For example, several studies have indicated that the TATA box-binding protein class may not be conserved among various protists which include several Plasmodium species^63–67^. Due to this lack of consensus, we propose renaming it the TATA box-like binding protein until additional investigations can resolve the inconsistencies. Such clarification is essential not only for accurate nomenclature but also for deepening our understanding of the functional roles of these motif classes in Plasmodium biology including their implications in gene regulation and developmental processes. This ongoing research is vital for unraveling the complexities of Plasmodium’s genetic framework and could significantly enhance our knowledge of its biology and pathogenicity.

## Conclusion

This study conducts a comprehensive analysis of de novo sequence motifs in *Plasmodium berghei* throughout its erythrocytic development phase, a crucial stage in the parasite’s life cycle that occurs within the red blood cells. Our findings identify specific motifs that are differentially expressed during this period thereby offering insights into the regulatory mechanisms that govern gene expression in the parasite.

Looking ahead, we plan to integrate additional datasets by integrating transcriptomic and epigenomic information to elucidate the precise temporal dynamics of motif activity. By employing more detailed time-series expression data obtained from ChIP-seq experiments, we aim to enhance our understanding of how these motifs interact with various transcription factors throughout the developmental stages of *P. berghei*. This approach will provide a more thorough understanding of the molecular processes involved and may contribute to the identification of potential therapeutic targets for malaria treatment.

## Supplementary Information

Supplementary Information 1.

Supplementary Information 2.

Supplementary Information 3.

Supplementary Information 4.

Supplementary Information 5.

Supplementary Information 6.

Supplementary Information 7.

Supplementary Information 8.

Supplementary Information 9.

Supplementary Information 10.

Supplementary Information 11.

Supplementary Information 12.

Supplementary Information 13.

Supplementary Information 14.

Supplementary Information 15.

## Data Availability

Data supporting the results reported in the article are included within the article and its supplementary materials. The datasets analysed during the current study are available in the Gene Expression Omnibus repository, under the accession numbers SRP027529, SRP250329, SRP099925, SRP073801, SRP142460, SRP048710, SRP211863, SRP069075, SRP046739, and in the Sequence Read Archives under the accession numbers ERP105548, ERP004740, SRP197607, SRP090611, SRP100893.

## Abbreviations

Abs: Absolute Value
AP2: Apetala2 DNA-Binding Domain
ATAC: Seq Assay for Transposase-Accessible Chromatin with Sequencing
bp: Base Pairs
DEGs: Differentially Expressed Genes
DNA: Deoxyribonucleic Acid
FC: Fold Change
GO: Gene Ontology
GTF: Gene Transfer Format
hpi: Hours Post-Infection
IDC: Intraerythrocytic Developmental Cycle
LC–MS/MS: Liquid Chromatograpy with Tandem Mass Spectroscopy
log: Logarithm
MEME: Multiple EM (Expectation Maximization) for Motif Elicitation
padj: Adjusted p-Value
PAM: Partitioning Around Medoids
Pb: *Plasmodium berghei*
PBM: Protein Binding Microarray
PC: Principal Component
PCA: Principal Component Analysis
PE: Paired End
Pf: *Plasmodium falciparum*
Pv: *Plasmodium vivax*
PVM: Parasitophorous Vacuolar Membrane
REVIGO: Reduce + Visualize Gene Ontology
RNA: Ribonucleic Acid
RNA: Seq RNA Sequencing
RSAT: Regulatory Sequence Analysis Tool
SE: Single End
spp: Species
STREME: Sensitive, Thorough, Rapid Enriched Motif Elicitation
TF: Transcription Factor
TSS: Transcription Start Site
XSTREME: Motif Discovery and Enrichment Analysis
5’UTR: Five Prime Untranslated Region
