# Supplementary material for: Transcriptome analysis reveals a de novo DNA element that may interact with chromatin-associated proteins in Plasmodium berghei during erythrocytic development
Source: Sci Rep. 2025 May 28;15:18621. doi: 10.1038/s41598-025-03586-4 (PMC12120095; doi:10.1038/s41598-025-03586-4)
Supplement: Supplementary file 1 — Supplementary Information 1. [file 41598_2025_3586_MOESM1_ESM.zip › Supplementary File S1/Clustering/matrix-clustering_SUMMARY_cluster2.html]

matrix-clustering coexpression\_cluster2


# RSAT - matrix-clustering result

### Analysis: coexpression\_cluster2 (09/09/2023 18:16)

### Command

```
matrix-clustering  -v 1 -max_matrices 300 -matrix coexpression_cluster2 $RSAT/public_html/tmp/www-data/2023/09/09/matrix-clustering_2023-09-09.181618_SMplQh/matrix-clustering_query_matrices.transfac transfac -hclust_method average -calc sum -title coexpression_cluster2 -metric_build_tree Ncor -lth w 5 -lth cor 0.6 -lth Ncor 0.4 -quick -label_in_tree name -return json,heatmap -o $RSAT/public_html/tmp/www-data/2023/09/09/matrix-clustering_2023-09-09.181618_SMplQh/matrix-clustering
```


## Results Summary

|  |  |  |  |  |  |  |  |  |  |  |
| --- | --- | --- | --- | --- | --- | --- | --- | --- | --- | --- |
| Nb Input motifs | Nb Input collections | Nb Clusters Found | Download   Root motifs | Complete results [zip] | Parameters used  |  |  |  | | --- | --- | --- | | Linkage method | Similarity metric | Thresholds to partition the tree | | | |
| 32 | 1 | 9 | **Download** | Download | |  |  |  | | --- | --- | --- | | average | Ncor | **Ncor** = 0.4 **cor** = 0.6 | |

|  |  |
| --- | --- |
|  | **Logo Forest (dynamic browsing)**  **Logo Forest (rapid overview - low image quality)**  cluster\_1  cluster\_2  cluster\_3  cluster\_4  cluster\_5  cluster\_6  cluster\_7  cluster\_8  cluster\_9 |
|  | |  |  |  |  |  |  |  |  |  |  | | --- | --- | --- | --- | --- | --- | --- | --- | --- | --- | | **Clusters Summary**  Clusters Summary Table  | Root Motif | Root Motif (Reverse) | Cluster ID | # Motifs | Motif\_Name::Collection | Number of Motifs by Collection | Root Motif (transfac Format) | | --- | --- | --- | --- | --- | --- | --- | |  | | --- | |  | | cluster\_1 | 8 | cluster2\_motif4\_r::coexpression\_cluster2:: cluster2\_motif8\_r::coexpression\_cluster2:: cluster2\_motif17\_r::coexpression\_cluster2:: cluster2\_motif5\_r::coexpression\_cluster2:: cluster2\_motif10\_r::coexpression\_cluster2:: cluster2\_motif2\_r::coexpression\_cluster2:: cluster2\_motif18\_r::coexpression\_cluster2:: cluster2\_motif1\_r::coexpression\_cluster2:: | coexpression\_cluster2::8\_motifs | Show matrix |
| cluster\_2 | 3 | cluster2\_motif24\_m::coexpression\_cluster2:: cluster2\_motif20\_m::coexpression\_cluster2:: cluster2\_motif26\_m::coexpression\_cluster2:: | coexpression\_cluster2::3\_motifs | Show matrix | | |
| cluster\_3 | 9 | cluster2\_motif12\_r::coexpression\_cluster2:: cluster2\_motif16\_r::coexpression\_cluster2:: cluster2\_motif3\_r::coexpression\_cluster2:: cluster2\_motif6\_r::coexpression\_cluster2:: cluster2\_motif19\_r::coexpression\_cluster2:: cluster2\_motif9\_r::coexpression\_cluster2:: cluster2\_motif27\_m::coexpression\_cluster2:: cluster2\_motif14\_r::coexpression\_cluster2:: cluster2\_motif7\_r::coexpression\_cluster2:: | coexpression\_cluster2::9\_motifs | Show matrix | | |
| cluster\_4 | 7 | cluster2\_motif30\_m::coexpression\_cluster2:: cluster2\_motif29\_m::coexpression\_cluster2:: cluster2\_motif21\_m::coexpression\_cluster2:: cluster2\_motif22\_m::coexpression\_cluster2:: cluster2\_motif25\_m::coexpression\_cluster2:: cluster2\_motif23\_m::coexpression\_cluster2:: cluster2\_motif28\_m::coexpression\_cluster2:: | coexpression\_cluster2::7\_motifs | Show matrix | | |
| cluster\_5 | 1 | cluster2\_motif15\_r::coexpression\_cluster2 | coexpression\_cluster2 : 1 motif | Show matrix | | |
| cluster\_6 | 1 | cluster2\_motif32\_m::coexpression\_cluster2 | coexpression\_cluster2 : 1 motif | Show matrix | | |
| cluster\_7 | 1 | cluster2\_motif11\_r::coexpression\_cluster2 | coexpression\_cluster2 : 1 motif | Show matrix | | |
| cluster\_8 | 1 | cluster2\_motif33\_m::coexpression\_cluster2 | coexpression\_cluster2 : 1 motif | Show matrix | | |
| cluster\_9 | 1 | cluster2\_motif31\_m::coexpression\_cluster2 | coexpression\_cluster2 : 1 motif | Show matrix | | |

- Note: by default the table displays the first 50 clusters. Click on the *Show X entries* button to display more (or less) clusters.
- Click on the column names to change the order of the data.
- Use the *Search* window to find your motif (s) of interest.

| **Individual Cluster View**  Individual Cluster View **Show All**  **Hide All**  **cluster\_1**  **cluster\_2**  **cluster\_3**  **cluster\_4**  **cluster\_5**  **cluster\_6**  **cluster\_7**  **cluster\_8**  **cluster\_9**  **Display Logo Trees**   **cluster\_1**    **cluster\_2**    **cluster\_3**    **cluster\_4**    **cluster\_5**    **cluster\_6**    **cluster\_7**    **cluster\_8**    **cluster\_9**  **Display Branch-Motifs** **cluster\_1**   | Branch Motifs | | | | | | --- | --- | --- | --- | --- | | Node | Consensus | Logo | Logo (Reverse) | Collections | Matrix (Transfac format) | IC | Nb sites | | Node 1 | **---------wwwATATTATATAATATAww----** |  |  | coexpression\_cluster2::2\_motifs | PSSM | 11.23 | 16744 | | --- | --- | --- | --- | --- | --- | --- | --- | | Node 2 | **wwwtwTATAATATTATTATATtwwwwwwww---** |  |  | coexpression\_cluster2::2\_motifs | PSSM | 15.14 | 7399 | | Node 3 | **-------wwwwwwTATAATATAATww-------** |  |  | coexpression\_cluster2::2\_motifs | PSSM | 10.36 | 16758 | | Node 4 | **------wwTwwwwTATTATATAATATAww----** |  |  | coexpression\_cluster2::3\_motifs | PSSM | 12.04 | 22358 | | Node 5 | **------wwTwwwwTATTATATAATwwAwwTAww** |  |  | coexpression\_cluster2::4\_motifs | PSSM | 13.32 | 30226 | | Node 6 | **------wwwwwwwTATwATATAATwwAwwTAww** |  |  | coexpression\_cluster2::6\_motifs | PSSM | 12.86 | 46984 | | Node 7 | **wwwtwTwwwwwwwTATwATATwATwwwwwwAww** |  |  | coexpression\_cluster2::8\_motifs | PSSM | 15.12 | 54383 |  **cluster\_2**   | Branch Motifs | | | | | | --- | --- | --- | --- | --- | | Node | Consensus | Logo | Logo (Reverse) | Collections | Matrix (Transfac format) | IC | Nb sites | | Node 1 | **ATAtATAyATAyATA** |  |  | coexpression\_cluster2::2\_motifs | PSSM | 13.56 | 1235 | | --- | --- | --- | --- | --- | --- | --- | --- | | Node 2 | **ATATATAyATAyATA** |  |  | coexpression\_cluster2::3\_motifs | PSSM | 13.08 | 1378 |  **cluster\_3**   | Branch Motifs | | | | | | --- | --- | --- | --- | --- | | Node | Consensus | Logo | Logo (Reverse) | Collections | Matrix (Transfac format) | IC | Nb sites | | Node 1 | **wwTTTTAdAAwAww** |  |  | coexpression\_cluster2::2\_motifs | PSSM | 8.65 | 13623 | | --- | --- | --- | --- | --- | --- | --- | --- | | Node 2 | **wwTTTTAkAwwww-** |  |  | coexpression\_cluster2::2\_motifs | PSSM | 8.37 | 10832 | | Node 3 | **wwTATtmTATww--** |  |  | coexpression\_cluster2::2\_motifs | PSSM | 8.02 | 6913 | | Node 4 | **wwTTTTAdAwwaww** |  |  | coexpression\_cluster2::4\_motifs | PSSM | 8.19 | 24455 | | Node 5 | **-wwATAmTAAww--** |  |  | coexpression\_cluster2::2\_motifs | PSSM | 7.41 | 8501 | | Node 6 | **wwTATtyTATww--** |  |  | coexpression\_cluster2::3\_motifs | PSSM | 7.96 | 7327 | | Node 7 | **wwTWTTAkAwwwww** |  |  | coexpression\_cluster2::7\_motifs | PSSM | 7.35 | 31782 | | Node 8 | **wwTwTwakAwwwww** |  |  | coexpression\_cluster2::9\_motifs | PSSM | 6.73 | 40283 |  **cluster\_4**   | Branch Motifs | | | | | | --- | --- | --- | --- | --- | | Node | Consensus | Logo | Logo (Reverse) | Collections | Matrix (Transfac format) | IC | Nb sites | | Node 1 | **-rAAAAAArAGrrAAA-** |  |  | coexpression\_cluster2::2\_motifs | PSSM | 11.91 | 700 | | --- | --- | --- | --- | --- | --- | --- | --- | | Node 2 | **-rAAAAAaraGraAAAA** |  |  | coexpression\_cluster2::3\_motifs | PSSM | 11.80 | 933 | | Node 3 | **-AAATwAAwAAAwAAA-** |  |  | coexpression\_cluster2::2\_motifs | PSSM | 12.45 | 850 | | Node 4 | **AAAATwAAwAAwwAAA-** |  |  | coexpression\_cluster2::3\_motifs | PSSM | 13.09 | 1048 | | Node 5 | **AAAAwwAAwarawAAAA** |  |  | coexpression\_cluster2::6\_motifs | PSSM | 11.79 | 1980 | | Node 6 | **AAAAwwAAwarawAAAA** |  |  | coexpression\_cluster2::7\_motifs | PSSM | 11.27 | 2026 |  **cluster\_5**   | Branch Motifs | | | | | | --- | --- | --- | --- | --- | | Node | Consensus | Logo | Logo (Reverse) | Collections | Matrix (Transfac format) | IC | Nb sites | | Singleton | **wwAGGTAAww** |  |  | coexpression\_cluster2 : 1 motif | PSSM | 9.25 | 270 | | --- | --- | --- | --- | --- | --- | --- | --- |  **cluster\_6**   | Branch Motifs | | | | | | --- | --- | --- | --- | --- | | Node | Consensus | Logo | Logo (Reverse) | Collections | Matrix (Transfac format) | IC | Nb sites | | Singleton | **CaStGCtaattGCYG** |  |  | coexpression\_cluster2 : 1 motif | PSSM | 15.09 | 18 | | --- | --- | --- | --- | --- | --- | --- | --- |  **cluster\_7**   | Branch Motifs | | | | | | --- | --- | --- | --- | --- | | Node | Consensus | Logo | Logo (Reverse) | Collections | Matrix (Transfac format) | IC | Nb sites | | Singleton | **wwGCACTAww** |  |  | coexpression\_cluster2 : 1 motif | PSSM | 9.06 | 123 | | --- | --- | --- | --- | --- | --- | --- | --- |  **cluster\_8**   | Branch Motifs | | | | | | --- | --- | --- | --- | --- | | Node | Consensus | Logo | Logo (Reverse) | Collections | Matrix (Transfac format) | IC | Nb sites | | Singleton | **tSGRaaaGrtGtGCa** |  |  | coexpression\_cluster2 : 1 motif | PSSM | 16.85 | 14 | | --- | --- | --- | --- | --- | --- | --- | --- |  **cluster\_9**   | Branch Motifs | | | | | | --- | --- | --- | --- | --- | | Node | Consensus | Logo | Logo (Reverse) | Collections | Matrix (Transfac format) | IC | Nb sites | | Singleton | **KyktKGGGGSG** |  |  | coexpression\_cluster2 : 1 motif | PSSM | 9.09 | 71 | | --- | --- | --- | --- | --- | --- | --- | --- |   - **Each cluster has a different color.** - **Click on the upper buttons to display separately the information of each cluster.** - **Click on the circles at each tree to display its corresponding merged motifs.**   Definitions  - **Logo tree.**  This results shows the hierarchical tree with its logo alignment of one cluster. The logos are shown in Forward (left logo) and Reverse (right logo) orientation. - **Branch-motifs.**  On the trees are displayed the branch number that is the number in which the motifs were incorporated in the tree. The Branch-Motifs table shows the logo in both orientations and the link to the file in TRANSFAC format with the branch-motif. |
|  | |  |  |  |  |  |  |  |  |  |  |  |  |  |  |  |  |  |  |  |  |  |  |  |  |  |  |  |  |  |  |  |  |  |  |  |  |  |  |  |  |  |  |  |  |  |  |  |  |  |  |  |  |  |  |  |  |  |  |  |  |  |  |  |  |  |  |  |  |  |  |  |  |  |  |  |  |  |  |  |  |  |  |  |  |  |  |  |  |  |  |  |  |  |  |  |  |  |  |  |  |  |  |  |  |  |  |  |  |  |  |  |  |  |  |  |  |  |  |  |  |  |  |  |  |  |  |  |  |  |  |  |  |  |  |  |  |  |  |  |  |  |  |  |  |  |  |  |  |  |  |  |  |  |  |  |  |  |  |  |  |  |  |  |  |  |  |  |  |  |  |  |  |  |  |  |  |  |  |  |  |  |  |  |  |  |  |  |  |  |  |  |  |  |  |  |  |  |  |  |  |  |  |  |  |  |  |  |  |  |  |  |  |  |  |  |  |  |  |  |  |  |  |  |  |  |  |  |  |  |  |  |  |  |  |  |  |  |  |  |  |  |  |  |  |  |  |  |  |  |  |  |  |  |  |  |  |  |  |  |  |  |  |  |  |  |  |  |  |  |  |  |  |  |  |  |  |  |  |  |  |  |  |  |  |  |  |  |  |  |  |  |  |  |  |  |  |  |  |  |  |  |  |  |  |  |  |  |  |  |  |  |  |  |  |  |  |  |  |  |  |  |  |  |  |  |  |  |  |  |  |  |  |  |  |  |  |  |  |  |  |  |  |  |  |  |  |  |  |  |  |  |  |  |  |  |  |  |  |  |  |  |  |  |  | | --- | --- | --- | --- | --- | --- | --- | --- | --- | --- | --- | --- | --- | --- | --- | --- | --- | --- | --- | --- | --- | --- | --- | --- | --- | --- | --- | --- | --- | --- | --- | --- | --- | --- | --- | --- | --- | --- | --- | --- | --- | --- | --- | --- | --- | --- | --- | --- | --- | --- | --- | --- | --- | --- | --- | --- | --- | --- | --- | --- | --- | --- | --- | --- | --- | --- | --- | --- | --- | --- | --- | --- | --- | --- | --- | --- | --- | --- | --- | --- | --- | --- | --- | --- | --- | --- | --- | --- | --- | --- | --- | --- | --- | --- | --- | --- | --- | --- | --- | --- | --- | --- | --- | --- | --- | --- | --- | --- | --- | --- | --- | --- | --- | --- | --- | --- | --- | --- | --- | --- | --- | --- | --- | --- | --- | --- | --- | --- | --- | --- | --- | --- | --- | --- | --- | --- | --- | --- | --- | --- | --- | --- | --- | --- | --- | --- | --- | --- | --- | --- | --- | --- | --- | --- | --- | --- | --- | --- | --- | --- | --- | --- | --- | --- | --- | --- | --- | --- | --- | --- | --- | --- | --- | --- | --- | --- | --- | --- | --- | --- | --- | --- | --- | --- | --- | --- | --- | --- | --- | --- | --- | --- | --- | --- | --- | --- | --- | --- | --- | --- | --- | --- | --- | --- | --- | --- | --- | --- | --- | --- | --- | --- | --- | --- | --- | --- | --- | --- | --- | --- | --- | --- | --- | --- | --- | --- | --- | --- | --- | --- | --- | --- | --- | --- | --- | --- | --- | --- | --- | --- | --- | --- | --- | --- | --- | --- | --- | --- | --- | --- | --- | --- | --- | --- | --- | --- | --- | --- | --- | --- | --- | --- | --- | --- | --- | --- | --- | --- | --- | --- | --- | --- | --- | --- | --- | --- | --- | --- | --- | --- | --- | --- | --- | --- | --- | --- | --- | --- | --- | --- | --- | --- | --- | --- | --- | --- | --- | --- | --- | --- | --- | --- | --- | --- | --- | --- | --- | --- | --- | --- | --- | --- | --- | --- | --- | --- | --- | --- | --- | --- | --- | --- | --- | --- | --- | --- | --- | --- | --- | --- | --- | --- | --- | --- | --- | --- | --- | --- | --- | --- | --- | --- | --- | --- | --- | --- | --- | --- | --- | --- | --- | --- | --- | --- | --- | --- | --- | --- | --- | --- | --- | --- | --- | --- | | **Individual Motif View**  Individual Motif View  | Motif id | Motif name | Cluster | Collection | Width | IC | Number of sites | Consensus | Consensus (Rev) | Logo | Logo (Rev) | | --- | --- | --- | --- | --- | --- | --- | --- | --- | --- | --- | | coexpression\_cluster2\_m19\_cluster2\_motif20\_m | cluster2\_motif20\_m | cluster\_2 | coexpression\_cluster2 | 15 | 13.12 | 942 | **ATATATAYATRYATA** | **TATryATrTATaTAT** |  |  | | coexpression\_cluster2\_m4\_cluster2\_motif4\_r | cluster2\_motif4\_r | cluster\_1 | coexpression\_cluster2 | 33 | 15.31 | 4244 | **wwwtwTATAATATTATTATATtwwwwwwww+++** | **+++WWWWWWWWAATATAATAATATTATAWAWWW** |  |  | | coexpression\_cluster2\_m22\_cluster2\_motif23\_m | cluster2\_motif23\_m | cluster\_4 | coexpression\_cluster2 | 17 | 13.03 | 481 | **+AAATAAATAAATAAA+** | **+TTTATTTATTTATTT+** |  |  | | coexpression\_cluster2\_m21\_cluster2\_motif22\_m | cluster2\_motif22\_m | cluster\_4 | coexpression\_cluster2 | 17 | 11.52 | 399 | **+++AAAAAraGrrAAA+** | **+TTTYYCTYTTTTT+++** |  |  | | coexpression\_cluster2\_m2\_cluster2\_motif2\_r | cluster2\_motif2\_r | cluster\_1 | coexpression\_cluster2 | 33 | 12.97 | 5614 | **++++++WWTATATTWTTATATAATATAWW++++** | **++++wwtATATTATATAAwAATATAww++++++** |  |  | | coexpression\_cluster2\_m1\_cluster2\_motif1\_r | cluster2\_motif1\_r | cluster\_1 | coexpression\_cluster2 | 33 | 11.53 | 7590 | **+++++++++WWTATATTATATAATATAWW++++** | **++++wwTATATTATATAATATAww+++++++++** |  |  | | coexpression\_cluster2\_m17\_cluster2\_motif18\_r | cluster2\_motif18\_r | cluster\_1 | coexpression\_cluster2 | 33 | 11.07 | 9154 | **+++++++++WWWATATTATATAATATAWW++++** | **++++wwtaTATTATATAATAtwww+++++++++** |  |  | | coexpression\_cluster2\_m15\_cluster2\_motif16\_r | cluster2\_motif16\_r | cluster\_3 | coexpression\_cluster2 | 14 | 9.43 | 893 | **++wwTAAGAAww++** | **++WWTTCTTAWW++** |  |  | | coexpression\_cluster2\_m8\_cluster2\_motif8\_r | cluster2\_motif8\_r | cluster\_1 | coexpression\_cluster2 | 33 | 15.48 | 3155 | **WWWTATATAATATTATTATATWAWWAWWWW+++** | **+++wwwwtwwtwATATAATAATATTATAtAwww** |  |  | | coexpression\_cluster2\_m20\_cluster2\_motif21\_m | cluster2\_motif21\_m | cluster\_4 | coexpression\_cluster2 | 17 | 12.22 | 301 | **+rAAAAAwrArarAAA+** | **+TTTYTYTYWTTTTTY+** |  |  | | coexpression\_cluster2\_m7\_cluster2\_motif7\_r | cluster2\_motif7\_r | cluster\_3 | coexpression\_cluster2 | 14 | 9.25 | 4484 | **WWTATTHTATWW++** | **++wwATAdAATAww** |  |  | | coexpression\_cluster2\_m14\_cluster2\_motif15\_r | cluster2\_motif15\_r | cluster\_5 | coexpression\_cluster2 | 10 | 9.25 | 270 | **wwAGGTAAww** | **WWTTACCTWW** |  |  | | coexpression\_cluster2\_m30\_cluster2\_motif31\_m | cluster2\_motif31\_m | cluster\_9 | coexpression\_cluster2 | 11 | 9.09 | 71 | **KTtTkGrGGkG** | **CMCCYCMAAAM** |  |  | | coexpression\_cluster2\_m13\_cluster2\_motif14\_r | cluster2\_motif14\_r | cluster\_3 | coexpression\_cluster2 | 14 | 7.33 | 2429 | **WWTATCCTAWW+++** | **+++wwTAGgATAww** |  |  | | coexpression\_cluster2\_m5\_cluster2\_motif5\_r | cluster2\_motif5\_r | cluster\_1 | coexpression\_cluster2 | 33 | 10.55 | 8507 | **+++++++wwAwAwTATAATATAwTww+++++++** | **+++++++WWAWTATATTATAWTWTWW+++++++** |  |  | | coexpression\_cluster2\_m6\_cluster2\_motif6\_r | cluster2\_motif6\_r | cluster\_3 | coexpression\_cluster2 | 14 | 8.95 | 7557 | **wwTTTTAdAAAAww** | **WWTTTTHTAAAAWW** |  |  | | coexpression\_cluster2\_m9\_cluster2\_motif9\_r | cluster2\_motif9\_r | cluster\_3 | coexpression\_cluster2 | 14 | 9.28 | 3289 | **WWTTTTAKATWWW+** | **+wwwATmTAAAAww** |  |  | | coexpression\_cluster2\_m32\_cluster2\_motif33\_m | cluster2\_motif33\_m | cluster\_8 | coexpression\_cluster2 | 15 | 16.85 | 14 | **TGGrAAAGATGTGCA** | **TGCACATCTTTYCCA** |  |  | | coexpression\_cluster2\_m26\_cluster2\_motif27\_m | cluster2\_motif27\_m | cluster\_3 | coexpression\_cluster2 | 14 | 12.35 | 414 | **TATATTTTTTT+++** | **+++AAAAAAATATA** |  |  | | coexpression\_cluster2\_m27\_cluster2\_motif28\_m | cluster2\_motif28\_m | cluster\_4 | coexpression\_cluster2 | 17 | 14.00 | 369 | **+AAATTAAAAAAAAA++** | **++TTTTTTTTTAATTT+** |  |  | | coexpression\_cluster2\_m3\_cluster2\_motif3\_r | cluster2\_motif3\_r | cluster\_3 | coexpression\_cluster2 | 14 | 9.07 | 6066 | **wwTTTTAdAATAww** | **WWTATTHTAAAAWW** |  |  | | coexpression\_cluster2\_m31\_cluster2\_motif32\_m | cluster2\_motif32\_m | cluster\_6 | coexpression\_cluster2 | 15 | 15.09 | 18 | **yAsTGCTAATTGmyG** | **CRKCAATTAGCASTR** |  |  | | coexpression\_cluster2\_m12\_cluster2\_motif12\_r | cluster2\_motif12\_r | cluster\_3 | coexpression\_cluster2 | 14 | 7.70 | 7608 | **+wwATAmTAAww++** | **++WWTTAKTATWW+** |  |  | | coexpression\_cluster2\_m25\_cluster2\_motif26\_m | cluster2\_motif26\_m | cluster\_2 | coexpression\_cluster2 | 15 | 15.81 | 293 | **ATATATATATAYAT+** | **+ATRTATATATATAT** |  |  | | coexpression\_cluster2\_m16\_cluster2\_motif17\_r | cluster2\_motif17\_r | cluster\_1 | coexpression\_cluster2 | 33 | 9.99 | 8251 | **+++++++++wwwwTATAATATAATww+++++++** | **+++++++WWATTATATTATAWWWW+++++++++** |  |  | | coexpression\_cluster2\_m23\_cluster2\_motif24\_m | cluster2\_motif24\_m | cluster\_2 | coexpression\_cluster2 | 15 | 12.48 | 143 | **ATATATGCATATAT+** | **+ATATATGCATATAT** |  |  | | coexpression\_cluster2\_m29\_cluster2\_motif30\_m | cluster2\_motif30\_m | cluster\_4 | coexpression\_cluster2 | 17 | 14.13 | 46 | **++RTTWGTKTRYTTKTT** | **AAMAArYAmACwAAy++** |  |  | | coexpression\_cluster2\_m24\_cluster2\_motif25\_m | cluster2\_motif25\_m | cluster\_4 | coexpression\_cluster2 | 17 | 15.59 | 199 | **TTTATTATTATTWTT++** | **++AAwAATAATAATAAA** |  |  | | coexpression\_cluster2\_m28\_cluster2\_motif29\_m | cluster2\_motif29\_m | cluster\_4 | coexpression\_cluster2 | 17 | 11.79 | 233 | **++TTTTTYCMYYTTTTT** | **AAAAArrkGrAAAAA++** |  |  | | coexpression\_cluster2\_m10\_cluster2\_motif10\_r | cluster2\_motif10\_r | cluster\_1 | coexpression\_cluster2 | 33 | 12.66 | 7868 | **+++++++++wwTATATTATATAwwwwATATAww** | **WWTATATWWWWTATATAATATAWW+++++++++** |  |  | | coexpression\_cluster2\_m11\_cluster2\_motif11\_r | cluster2\_motif11\_r | cluster\_7 | coexpression\_cluster2 | 10 | 9.06 | 123 | **wwGCACTAww** | **WWTAGTGCWW** |  |  | | coexpression\_cluster2\_m18\_cluster2\_motif19\_r | cluster2\_motif19\_r | cluster\_3 | coexpression\_cluster2 | 14 | 7.96 | 7543 | **WWTTTTAKAWWW++** | **++wwwTmTAAAAww** |  |  |  - Click on the column names to change the order of the data. - Write the name of one cluster, collection or a pattern in the *Search* window. | | **Heatmap View**  Heatmap View Distance table | PDF | |

**Additional Files**

## Exported files

| File | Description |
| --- | --- |
| **Pairwise comparison** | This table shows the pairwise comparison between all the input motifs using different metrics. This is the *compare-matrices* result. |
| **Matrix description** | This table shows information of each input motif. |
| **Cluster-wise Motif Files [zip]** | One TRANSFAC file is generated for each cluster including all the input motifs grouped in the corresponding cluster. |
| **Root motifs** | A file with the root matrix of each cluster. |
| **Motif collections per cluster** | A tab separated file containing the counts of motif of each collection on each cluster. |
| **Clusters** | A tab separated file containing the clusters and their correspondng motifs. |
| **Central motifs IDs** | A tab separated file containing the central motif, that one who is the most similar to all the others motif in the same cluster. |
| **Central motifs (in TRANSFAC format)** | A transfac file containing the central motif, that one who is the most similar to all the others motif within the same cluster. |
| **Input motifs** | The input motifs analyzed with matrix-clustering. |

- **References**
  1. Castro-Mondragon JA et al.  RSAT matrix-clustering: dynamic exploration and redundancy reduction of transcription factor binding motif collections. doi: 10.1093/nar/gkx314; Nucleic Acids Research (2017)
  2. **Last RSAT release:** Nguyen NTT et al. RSAT 2018: regulatory sequence analysis tools 20th anniversary. Nucleic Acids Research (2018).
- **Additional Information**
  - Jacques van Helden course
- **Contact**
  - Jaime Castro-Mondragon - **j.a.c.mondragon@ncmm.uio.no**
  - Morgane Thomas-Chollier - **mthomas@biologie.ens.fr**
  - Jacques van Helden - **Jacques.van-Helden@univ-amu.fr**
