# Supplementary material for: Transcriptome analysis reveals a de novo DNA element that may interact with chromatin-associated proteins in Plasmodium berghei during erythrocytic development
Source: Sci Rep. 2025 May 28;15:18621. doi: 10.1038/s41598-025-03586-4 (PMC12120095; doi:10.1038/s41598-025-03586-4)
Supplement: Supplementary file 1 — Supplementary Information 1. [file 41598_2025_3586_MOESM1_ESM.zip › Supplementary File S1/Clustering/matrix-clustering_SUMMARY_cluster1.html]

matrix-clustering coexpression\_cluster1


# RSAT - matrix-clustering result

### Analysis: coexpression\_cluster1 (07/09/2023 11:03)

### Command

```
matrix-clustering  -v 1 -max_matrices 300 -matrix coexpression_cluster1 $RSAT/public_html/tmp/www-data/2023/09/07/matrix-clustering_2023-09-07.110304_kSNkrp/matrix-clustering_query_matrices.transfac transfac -hclust_method average -calc sum -title coexpression_cluster1 -metric_build_tree Ncor -lth w 5 -lth cor 0.6 -lth Ncor 0.4 -quick -label_in_tree name -return json,heatmap -o $RSAT/public_html/tmp/www-data/2023/09/07/matrix-clustering_2023-09-07.110304_kSNkrp/matrix-clustering
```


## Results Summary

|  |  |  |  |  |  |  |  |  |  |  |
| --- | --- | --- | --- | --- | --- | --- | --- | --- | --- | --- |
| Nb Input motifs | Nb Input collections | Nb Clusters Found | Download   Root motifs | Complete results [zip] | Parameters used  |  |  |  | | --- | --- | --- | | Linkage method | Similarity metric | Thresholds to partition the tree | | | |
| 44 | 1 | 13 | **Download** | Download | |  |  |  | | --- | --- | --- | | average | Ncor | **Ncor** = 0.4 **cor** = 0.6 | |

|  |  |
| --- | --- |
|  | **Logo Forest (dynamic browsing)**  **Logo Forest (rapid overview - low image quality)**  cluster\_1  cluster\_2  cluster\_3  cluster\_4  cluster\_5  cluster\_6  cluster\_7  cluster\_8  cluster\_9  cluster\_10  cluster\_11  cluster\_12  cluster\_13 |
|  | |  |  |  |  |  |  |  |  |  |  | | --- | --- | --- | --- | --- | --- | --- | --- | --- | --- | | **Clusters Summary**  Clusters Summary Table  | Root Motif | Root Motif (Reverse) | Cluster ID | # Motifs | Motif\_Name::Collection | Number of Motifs by Collection | Root Motif (transfac Format) | | --- | --- | --- | --- | --- | --- | --- | |  | | --- | |  | | cluster\_1 | 17 | cluster1\_motif33\_m::coexpression\_cluster1:: cluster1\_motif11\_r::coexpression\_cluster1:: cluster1\_motif2\_r::coexpression\_cluster1:: cluster1\_motif12\_r::coexpression\_cluster1:: cluster1\_motif1\_r::coexpression\_cluster1:: cluster1\_motif15\_r::coexpression\_cluster1:: cluster1\_motif7\_r::coexpression\_cluster1:: cluster1\_motif8\_r::coexpression\_cluster1:: cluster1\_motif3\_r::coexpression\_cluster1:: cluster1\_motif25\_r::coexpression\_cluster1:: cluster1\_motif9\_r::coexpression\_cluster1:: cluster1\_motif21\_r::coexpression\_cluster1:: cluster1\_motif40\_m::coexpression\_cluster1:: cluster1\_motif41\_m::coexpression\_cluster1:: cluster1\_motif28\_m::coexpression\_cluster1:: cluster1\_motif32\_m::coexpression\_cluster1:: cluster1\_motif34\_m::coexpression\_cluster1:: | coexpression\_cluster1::17\_motifs | Show matrix |
| cluster\_2 | 4 | cluster1\_motif20\_r::coexpression\_cluster1:: cluster1\_motif24\_r::coexpression\_cluster1:: cluster1\_motif23\_r::coexpression\_cluster1:: cluster1\_motif5\_r::coexpression\_cluster1:: | coexpression\_cluster1::4\_motifs | Show matrix | | |
| cluster\_3 | 6 | cluster1\_motif16\_r::coexpression\_cluster1:: cluster1\_motif17\_r::coexpression\_cluster1:: cluster1\_motif14\_r::coexpression\_cluster1:: cluster1\_motif19\_r::coexpression\_cluster1:: cluster1\_motif22\_r::coexpression\_cluster1:: cluster1\_motif6\_r::coexpression\_cluster1:: | coexpression\_cluster1::6\_motifs | Show matrix | | |
| cluster\_4 | 7 | cluster1\_motif43\_m::coexpression\_cluster1:: cluster1\_motif26\_m::coexpression\_cluster1:: cluster1\_motif27\_m::coexpression\_cluster1:: cluster1\_motif10\_r::coexpression\_cluster1:: cluster1\_motif31\_m::coexpression\_cluster1:: cluster1\_motif30\_m::coexpression\_cluster1:: cluster1\_motif4\_r::coexpression\_cluster1:: | coexpression\_cluster1::7\_motifs | Show matrix | | |
| cluster\_5 | 2 | cluster1\_motif29\_m::coexpression\_cluster1:: cluster1\_motif38\_m::coexpression\_cluster1:: | coexpression\_cluster1::2\_motifs | Show matrix | | |
| cluster\_6 | 1 | cluster1\_motif18\_r::coexpression\_cluster1 | coexpression\_cluster1 : 1 motif | Show matrix | | |
| cluster\_7 | 1 | cluster1\_motif37\_m::coexpression\_cluster1 | coexpression\_cluster1 : 1 motif | Show matrix | | |
| cluster\_8 | 1 | cluster1\_motif13\_r::coexpression\_cluster1 | coexpression\_cluster1 : 1 motif | Show matrix | | |
| cluster\_9 | 1 | cluster1\_motif36\_m::coexpression\_cluster1 | coexpression\_cluster1 : 1 motif | Show matrix | | |
| cluster\_10 | 1 | cluster1\_motif39\_m::coexpression\_cluster1 | coexpression\_cluster1 : 1 motif | Show matrix | | |
| cluster\_11 | 1 | cluster1\_motif35\_m::coexpression\_cluster1 | coexpression\_cluster1 : 1 motif | Show matrix | | |
| cluster\_12 | 1 | cluster1\_motif44\_m::coexpression\_cluster1 | coexpression\_cluster1 : 1 motif | Show matrix | | |
| cluster\_13 | 1 | cluster1\_motif42\_m::coexpression\_cluster1 | coexpression\_cluster1 : 1 motif | Show matrix | | |

- Note: by default the table displays the first 50 clusters. Click on the *Show X entries* button to display more (or less) clusters.
- Click on the column names to change the order of the data.
- Use the *Search* window to find your motif (s) of interest.

| **Individual Cluster View**  Individual Cluster View **Show All**  **Hide All**  **cluster\_1**  **cluster\_2**  **cluster\_3**  **cluster\_4**  **cluster\_5**  **cluster\_6**  **cluster\_7**  **cluster\_8**  **cluster\_9**  **cluster\_10**  **cluster\_11**  **cluster\_12**  **cluster\_13**  **Display Logo Trees**   **cluster\_1**    **cluster\_2**    **cluster\_3**    **cluster\_4**    **cluster\_5**    **cluster\_6**    **cluster\_7**    **cluster\_8**    **cluster\_9**    **cluster\_10**    **cluster\_11**    **cluster\_12**    **cluster\_13**  **Display Branch-Motifs** **cluster\_1**   | Branch Motifs | | | | | | --- | --- | --- | --- | --- | | Node | Consensus | Logo | Logo (Reverse) | Collections | Matrix (Transfac format) | IC | Nb sites | | Node 1 | **wwwAAgACAAwwww-** |  |  | coexpression\_cluster1::2\_motifs | PSSM | 8.53 | 3830 | | --- | --- | --- | --- | --- | --- | --- | --- | | Node 2 | **wwAAAgACAwww---** |  |  | coexpression\_cluster1::2\_motifs | PSSM | 7.80 | 5667 | | Node 3 | **wwwTAgACAAwwww-** |  |  | coexpression\_cluster1::2\_motifs | PSSM | 8.52 | 3731 | | Node 4 | **wwwAAgACAAwwww-** |  |  | coexpression\_cluster1::3\_motifs | PSSM | 8.55 | 6444 | | Node 5 | **wwAAAgACAwwww--** |  |  | coexpression\_cluster1::3\_motifs | PSSM | 8.09 | 7402 | | Node 6 | **wwaAAgAcAAwwwww** |  |  | coexpression\_cluster1::4\_motifs | PSSM | 8.67 | 8885 | | Node 7 | **rAAAAAAwAAAAAAM** |  |  | coexpression\_cluster1::2\_motifs | PSSM | 13.23 | 1137 | | Node 8 | **wwaWAgACAAwwwww** |  |  | coexpression\_cluster1::6\_motifs | PSSM | 8.20 | 12616 | | Node 9 | **wwAAAgACAwwwaww** |  |  | coexpression\_cluster1::4\_motifs | PSSM | 8.65 | 8769 | | Node 10 | **rAAAAAAarAAAAAM** |  |  | coexpression\_cluster1::3\_motifs | PSSM | 12.32 | 1412 | | Node 11 | **wwATrAAcAAAwwwA** |  |  | coexpression\_cluster1::2\_motifs | PSSM | 10.11 | 4689 | | Node 12 | **wwAAAgACAawwwww** |  |  | coexpression\_cluster1::10\_motifs | PSSM | 7.90 | 21385 | | Node 13 | **rAAAAAAAAAwAAAM** |  |  | coexpression\_cluster1::4\_motifs | PSSM | 12.20 | 1961 | | Node 14 | **waAwaAAmAAAwawA** |  |  | coexpression\_cluster1::6\_motifs | PSSM | 9.10 | 6648 | | Node 15 | **wwAWArAcAAwwwwa** |  |  | coexpression\_cluster1::16\_motifs | PSSM | 7.51 | 28033 | | Node 16 | **wwAWArAcAAwwwwa** |  |  | coexpression\_cluster1::17\_motifs | PSSM | 7.51 | 28333 |  **cluster\_2**   | Branch Motifs | | | | | | --- | --- | --- | --- | --- | | Node | Consensus | Logo | Logo (Reverse) | Collections | Matrix (Transfac format) | IC | Nb sites | | Node 1 | **wwaTGTTGTAww** |  |  | coexpression\_cluster1::2\_motifs | PSSM | 7.92 | 2634 | | --- | --- | --- | --- | --- | --- | --- | --- | | Node 2 | **wwwTGTTkTwww** |  |  | coexpression\_cluster1::3\_motifs | PSSM | 7.86 | 4889 | | Node 3 | **wwwTGTTkTwww** |  |  | coexpression\_cluster1::4\_motifs | PSSM | 7.67 | 5090 |  **cluster\_3**   | Branch Motifs | | | | | | --- | --- | --- | --- | --- | | Node | Consensus | Logo | Logo (Reverse) | Collections | Matrix (Transfac format) | IC | Nb sites | | Node 1 | **wwwwaAAmAAAAATwTtTAAAtww** |  |  | coexpression\_cluster1::2\_motifs | PSSM | 12.57 | 6001 | | --- | --- | --- | --- | --- | --- | --- | --- | | Node 2 | **--------wwwaATTTTTAAAwww** |  |  | coexpression\_cluster1::2\_motifs | PSSM | 9.22 | 14538 | | Node 3 | **wwAAaaAmAAAAAwwtwtAwAwww** |  |  | coexpression\_cluster1::3\_motifs | PSSM | 11.95 | 13464 | | Node 4 | **wwAwawwaAAAAATwTwtAAAwww** |  |  | coexpression\_cluster1::4\_motifs | PSSM | 11.64 | 21224 | | Node 5 | **wwAwawwaAawaATTTTTAAAwww** |  |  | coexpression\_cluster1::6\_motifs | PSSM | 11.34 | 35762 |  **cluster\_4**   | Branch Motifs | | | | | | --- | --- | --- | --- | --- | | Node | Consensus | Logo | Logo (Reverse) | Collections | Matrix (Transfac format) | IC | Nb sites | | Node 1 | **TATATRTATrTATry** |  |  | coexpression\_cluster1::2\_motifs | PSSM | 13.28 | 1269 | | --- | --- | --- | --- | --- | --- | --- | --- | | Node 2 | **---atACACACATww** |  |  | coexpression\_cluster1::2\_motifs | PSSM | 8.69 | 1868 | | Node 3 | **TATATATATryATry** |  |  | coexpression\_cluster1::3\_motifs | PSSM | 13.08 | 1472 | | Node 4 | **--waTrCATGCAyA-** |  |  | coexpression\_cluster1::2\_motifs | PSSM | 7.80 | 1602 | | Node 5 | **--watrCAyrCATaw** |  |  | coexpression\_cluster1::4\_motifs | PSSM | 7.45 | 3470 | | Node 6 | **TATaTryAyrYATay** |  |  | coexpression\_cluster1::7\_motifs | PSSM | 9.83 | 4942 |  **cluster\_5**   | Branch Motifs | | | | | | --- | --- | --- | --- | --- | | Node | Consensus | Logo | Logo (Reverse) | Collections | Matrix (Transfac format) | IC | Nb sites | | Node 1 | **yygtrwyAGCTAGCTrwhtwtr** |  |  | coexpression\_cluster1::2\_motifs | PSSM | 9.46 | 283 | | --- | --- | --- | --- | --- | --- | --- | --- |  **cluster\_6**   | Branch Motifs | | | | | | --- | --- | --- | --- | --- | | Node | Consensus | Logo | Logo (Reverse) | Collections | Matrix (Transfac format) | IC | Nb sites | | Singleton | **wwATTACACww** |  |  | coexpression\_cluster1 : 1 motif | PSSM | 7.22 | 3137 | | --- | --- | --- | --- | --- | --- | --- | --- |  **cluster\_7**   | Branch Motifs | | | | | | --- | --- | --- | --- | --- | | Node | Consensus | Logo | Logo (Reverse) | Collections | Matrix (Transfac format) | IC | Nb sites | | Singleton | **taatmatartaarr** |  |  | coexpression\_cluster1 : 1 motif | PSSM | 15.20 | 85 | | --- | --- | --- | --- | --- | --- | --- | --- |  **cluster\_8**   | Branch Motifs | | | | | | --- | --- | --- | --- | --- | | Node | Consensus | Logo | Logo (Reverse) | Collections | Matrix (Transfac format) | IC | Nb sites | | Singleton | **wwTCTGAAww** |  |  | coexpression\_cluster1 : 1 motif | PSSM | 9.23 | 216 | | --- | --- | --- | --- | --- | --- | --- | --- |  **cluster\_9**   | Branch Motifs | | | | | | --- | --- | --- | --- | --- | | Node | Consensus | Logo | Logo (Reverse) | Collections | Matrix (Transfac format) | IC | Nb sites | | Singleton | **tGaaCaaaty** |  |  | coexpression\_cluster1 : 1 motif | PSSM | 11.11 | 227 | | --- | --- | --- | --- | --- | --- | --- | --- |  **cluster\_10**   | Branch Motifs | | | | | | --- | --- | --- | --- | --- | | Node | Consensus | Logo | Logo (Reverse) | Collections | Matrix (Transfac format) | IC | Nb sites | | Singleton | **mbSssssyGCaCaCrCvSSSSS** |  |  | coexpression\_cluster1 : 1 motif | PSSM | 10.85 | 148 | | --- | --- | --- | --- | --- | --- | --- | --- |  **cluster\_11**   | Branch Motifs | | | | | | --- | --- | --- | --- | --- | | Node | Consensus | Logo | Logo (Reverse) | Collections | Matrix (Transfac format) | IC | Nb sites | | Singleton | **GCatGC** |  |  | coexpression\_cluster1 : 1 motif | PSSM | 6.26 | 168 | | --- | --- | --- | --- | --- | --- | --- | --- |  **cluster\_12**   | Branch Motifs | | | | | | --- | --- | --- | --- | --- | | Node | Consensus | Logo | Logo (Reverse) | Collections | Matrix (Transfac format) | IC | Nb sites | | Singleton | **CatttCatGaCCCat** |  |  | coexpression\_cluster1 : 1 motif | PSSM | 20.79 | 6 | | --- | --- | --- | --- | --- | --- | --- | --- |  **cluster\_13**   | Branch Motifs | | | | | | --- | --- | --- | --- | --- | | Node | Consensus | Logo | Logo (Reverse) | Collections | Matrix (Transfac format) | IC | Nb sites | | Singleton | **MSSkMCCCCCMVtmy** |  |  | coexpression\_cluster1 : 1 motif | PSSM | 9.94 | 25 | | --- | --- | --- | --- | --- | --- | --- | --- |   - **Each cluster has a different color.** - **Click on the upper buttons to display separately the information of each cluster.** - **Click on the circles at each tree to display its corresponding merged motifs.**   Definitions  - **Logo tree.**  This results shows the hierarchical tree with its logo alignment of one cluster. The logos are shown in Forward (left logo) and Reverse (right logo) orientation. - **Branch-motifs.**  On the trees are displayed the branch number that is the number in which the motifs were incorporated in the tree. The Branch-Motifs table shows the logo in both orientations and the link to the file in TRANSFAC format with the branch-motif. |
|  | |  |  |  |  |  |  |  |  |  |  |  |  |  |  |  |  |  |  |  |  |  |  |  |  |  |  |  |  |  |  |  |  |  |  |  |  |  |  |  |  |  |  |  |  |  |  |  |  |  |  |  |  |  |  |  |  |  |  |  |  |  |  |  |  |  |  |  |  |  |  |  |  |  |  |  |  |  |  |  |  |  |  |  |  |  |  |  |  |  |  |  |  |  |  |  |  |  |  |  |  |  |  |  |  |  |  |  |  |  |  |  |  |  |  |  |  |  |  |  |  |  |  |  |  |  |  |  |  |  |  |  |  |  |  |  |  |  |  |  |  |  |  |  |  |  |  |  |  |  |  |  |  |  |  |  |  |  |  |  |  |  |  |  |  |  |  |  |  |  |  |  |  |  |  |  |  |  |  |  |  |  |  |  |  |  |  |  |  |  |  |  |  |  |  |  |  |  |  |  |  |  |  |  |  |  |  |  |  |  |  |  |  |  |  |  |  |  |  |  |  |  |  |  |  |  |  |  |  |  |  |  |  |  |  |  |  |  |  |  |  |  |  |  |  |  |  |  |  |  |  |  |  |  |  |  |  |  |  |  |  |  |  |  |  |  |  |  |  |  |  |  |  |  |  |  |  |  |  |  |  |  |  |  |  |  |  |  |  |  |  |  |  |  |  |  |  |  |  |  |  |  |  |  |  |  |  |  |  |  |  |  |  |  |  |  |  |  |  |  |  |  |  |  |  |  |  |  |  |  |  |  |  |  |  |  |  |  |  |  |  |  |  |  |  |  |  |  |  |  |  |  |  |  |  |  |  |  |  |  |  |  |  |  |  |  |  |  |  |  |  |  |  |  |  |  |  |  |  |  |  |  |  |  |  |  |  |  |  |  |  |  |  |  |  |  |  |  |  |  |  |  |  |  |  |  |  |  |  |  |  |  |  |  |  |  |  |  |  |  |  |  |  |  |  |  |  |  |  |  |  |  |  |  |  |  |  |  |  |  |  |  |  |  |  |  |  |  |  |  |  |  |  |  |  |  |  |  |  |  |  |  |  |  |  |  |  |  |  |  |  |  |  |  |  |  |  |  |  |  |  |  |  |  |  |  |  |  |  |  |  |  |  |  |  |  |  | | --- | --- | --- | --- | --- | --- | --- | --- | --- | --- | --- | --- | --- | --- | --- | --- | --- | --- | --- | --- | --- | --- | --- | --- | --- | --- | --- | --- | --- | --- | --- | --- | --- | --- | --- | --- | --- | --- | --- | --- | --- | --- | --- | --- | --- | --- | --- | --- | --- | --- | --- | --- | --- | --- | --- | --- | --- | --- | --- | --- | --- | --- | --- | --- | --- | --- | --- | --- | --- | --- | --- | --- | --- | --- | --- | --- | --- | --- | --- | --- | --- | --- | --- | --- | --- | --- | --- | --- | --- | --- | --- | --- | --- | --- | --- | --- | --- | --- | --- | --- | --- | --- | --- | --- | --- | --- | --- | --- | --- | --- | --- | --- | --- | --- | --- | --- | --- | --- | --- | --- | --- | --- | --- | --- | --- | --- | --- | --- | --- | --- | --- | --- | --- | --- | --- | --- | --- | --- | --- | --- | --- | --- | --- | --- | --- | --- | --- | --- | --- | --- | --- | --- | --- | --- | --- | --- | --- | --- | --- | --- | --- | --- | --- | --- | --- | --- | --- | --- | --- | --- | --- | --- | --- | --- | --- | --- | --- | --- | --- | --- | --- | --- | --- | --- | --- | --- | --- | --- | --- | --- | --- | --- | --- | --- | --- | --- | --- | --- | --- | --- | --- | --- | --- | --- | --- | --- | --- | --- | --- | --- | --- | --- | --- | --- | --- | --- | --- | --- | --- | --- | --- | --- | --- | --- | --- | --- | --- | --- | --- | --- | --- | --- | --- | --- | --- | --- | --- | --- | --- | --- | --- | --- | --- | --- | --- | --- | --- | --- | --- | --- | --- | --- | --- | --- | --- | --- | --- | --- | --- | --- | --- | --- | --- | --- | --- | --- | --- | --- | --- | --- | --- | --- | --- | --- | --- | --- | --- | --- | --- | --- | --- | --- | --- | --- | --- | --- | --- | --- | --- | --- | --- | --- | --- | --- | --- | --- | --- | --- | --- | --- | --- | --- | --- | --- | --- | --- | --- | --- | --- | --- | --- | --- | --- | --- | --- | --- | --- | --- | --- | --- | --- | --- | --- | --- | --- | --- | --- | --- | --- | --- | --- | --- | --- | --- | --- | --- | --- | --- | --- | --- | --- | --- | --- | --- | --- | --- | --- | --- | --- | --- | --- | --- | --- | --- | --- | --- | --- | --- | --- | --- | --- | --- | --- | --- | --- | --- | --- | --- | --- | --- | --- | --- | --- | --- | --- | --- | --- | --- | --- | --- | --- | --- | --- | --- | --- | --- | --- | --- | --- | --- | --- | --- | --- | --- | --- | --- | --- | --- | --- | --- | --- | --- | --- | --- | --- | --- | --- | --- | --- | --- | --- | --- | --- | --- | --- | --- | --- | --- | --- | --- | --- | --- | --- | --- | --- | --- | --- | --- | --- | --- | --- | --- | --- | --- | --- | --- | --- | --- | --- | --- | --- | --- | --- | --- | --- | --- | --- | --- | --- | --- | --- | --- | --- | --- | --- | --- | --- | --- | --- | --- | --- | --- | --- | --- | --- | --- | --- | --- | --- | --- | --- | --- | --- | --- | --- | --- | --- | --- | --- | --- | --- | --- | --- | --- | --- | --- | --- | --- | --- | --- | --- | --- | --- | --- | --- | --- | | **Individual Motif View**  Individual Motif View  | Motif id | Motif name | Cluster | Collection | Width | IC | Number of sites | Consensus | Consensus (Rev) | Logo | Logo (Rev) | | --- | --- | --- | --- | --- | --- | --- | --- | --- | --- | --- | | coexpression\_cluster1\_m38\_cluster1\_motif38\_m | cluster1\_motif38\_m | cluster\_5 | coexpression\_cluster1 | 22 | 9.77 | 201 | **yygtrwyAGCTAGCTvrckmtr** | **YAKMGYBAGCTAGCTRWYACRR** |  |  | | coexpression\_cluster1\_m8\_cluster1\_motif8\_r | cluster1\_motif8\_r | cluster\_1 | coexpression\_cluster1 | 15 | 9.39 | 1367 | **WWTDYATGTCTTTWW** | **wwAAAgACATrhAww** |  |  | | coexpression\_cluster1\_m18\_cluster1\_motif18\_r | cluster1\_motif18\_r | cluster\_6 | coexpression\_cluster1 | 11 | 7.22 | 3137 | **wwATTACACww** | **WWGTGTAATWW** |  |  | | coexpression\_cluster1\_m32\_cluster1\_motif32\_m | cluster1\_motif32\_m | cluster\_1 | coexpression\_cluster1 | 15 | 14.02 | 873 | **RAAAAAAWAARAAAA** | **TTTTYTTwTTTTTTy** |  |  | | coexpression\_cluster1\_m31\_cluster1\_motif31\_m | cluster1\_motif31\_m | cluster\_4 | coexpression\_cluster1 | 15 | 11.62 | 128 | **+++ATgCACACATrT** | **AYATGTGTGCAT+++** |  |  | | coexpression\_cluster1\_m15\_cluster1\_motif15\_r | cluster1\_motif15\_r | cluster\_1 | coexpression\_cluster1 | 15 | 8.59 | 1677 | **wwwAAgACAATwww+** | **+WWWATTGTCTTWWW** |  |  | | coexpression\_cluster1\_m28\_cluster1\_motif28\_m | cluster1\_motif28\_m | cluster\_1 | coexpression\_cluster1 | 15 | 11.68 | 276 | **+TYTTTCTCTTTTT+** | **+AAAAAGAGAAArA+** |  |  | | coexpression\_cluster1\_m14\_cluster1\_motif14\_r | cluster1\_motif14\_r | cluster\_3 | coexpression\_cluster1 | 24 | 11.36 | 7760 | **+++WWWTTTAAAAATTTTTTWWWW** | **wwwwAAAAAATTTTTAAAwww+++** |  |  | | coexpression\_cluster1\_m17\_cluster1\_motif17\_r | cluster1\_motif17\_r | cluster\_3 | coexpression\_cluster1 | 24 | 9.39 | 9276 | **++++++++WWWAATTTTTAAAWWW** | **wwwTTTAAAAATTwww++++++++** |  |  | | coexpression\_cluster1\_m36\_cluster1\_motif36\_m | cluster1\_motif36\_m | cluster\_9 | coexpression\_cluster1 | 10 | 11.11 | 227 | **TGAACAAATT** | **AATTTGTTCA** |  |  | | coexpression\_cluster1\_m26\_cluster1\_motif26\_m | cluster1\_motif26\_m | cluster\_4 | coexpression\_cluster1 | 15 | 13.08 | 1000 | **TATATATATryATry** | **RYATRYATATATATA** |  |  | | coexpression\_cluster1\_m5\_cluster1\_motif5\_r | cluster1\_motif5\_r | cluster\_2 | coexpression\_cluster1 | 12 | 8.10 | 836 | **WWGTGTTGTAWW** | **wwtACAACAcww** |  |  | | coexpression\_cluster1\_m21\_cluster1\_motif21\_r | cluster1\_motif21\_r | cluster\_1 | coexpression\_cluster1 | 15 | 9.29 | 4491 | **wwATrAAcAAAwww+** | **+WWWTTTGTTYATWW** |  |  | | coexpression\_cluster1\_m33\_cluster1\_motif33\_m | cluster1\_motif33\_m | cluster\_1 | coexpression\_cluster1 | 15 | 13.42 | 300 | **+TATATATTTTTT++** | **++AAAAAATATATA+** |  |  | | coexpression\_cluster1\_m29\_cluster1\_motif29\_m | cluster1\_motif29\_m | cluster\_5 | coexpression\_cluster1 | 22 | 11.34 | 82 | **+++++++AGCTAGCYAwwwwt+** | **+AWWWWTRGCTAGCT+++++++** |  |  | | coexpression\_cluster1\_m19\_cluster1\_motif19\_r | cluster1\_motif19\_r | cluster\_3 | coexpression\_cluster1 | 24 | 12.38 | 7463 | **WWWTWTWWWWWTTTTTKTYTTTWW** | **wwAAArAmAAAAAwwwwwAwAwww** |  |  | | coexpression\_cluster1\_m12\_cluster1\_motif12\_r | cluster1\_motif12\_r | cluster\_1 | coexpression\_cluster1 | 15 | 9.70 | 2441 | **WWWTKTTKTYTTTWW** | **wwAAArAmAAmAwww** |  |  | | coexpression\_cluster1\_m37\_cluster1\_motif37\_m | cluster1\_motif37\_m | cluster\_7 | coexpression\_cluster1 | 14 | 15.20 | 85 | **TAATAATAATAAwA** | **TWTTATTATTATTA** |  |  | | coexpression\_cluster1\_m43\_cluster1\_motif43\_m | cluster1\_motif43\_m | cluster\_4 | coexpression\_cluster1 | 15 | 12.92 | 203 | **++TATATATGCATGY** | **rCATGCATATATA++** |  |  | | coexpression\_cluster1\_m25\_cluster1\_motif25\_r | cluster1\_motif25\_r | cluster\_1 | coexpression\_cluster1 | 15 | 7.85 | 4724 | **wwAAAgACAwww+++** | **+++WWWTGTCTTTWW** |  |  | | coexpression\_cluster1\_m11\_cluster1\_motif11\_r | cluster1\_motif11\_r | cluster\_1 | coexpression\_cluster1 | 15 | 8.50 | 1748 | **WWWTAGACAAWWWW+** | **+wwwwTTGTcTAwww** |  |  | | coexpression\_cluster1\_m4\_cluster1\_motif4\_r | cluster1\_motif4\_r | cluster\_4 | coexpression\_cluster1 | 15 | 8.93 | 1058 | **++WATGCATGCATW+** | **+waTGCATGCAtw++** |  |  | | coexpression\_cluster1\_m39\_cluster1\_motif39\_m | cluster1\_motif39\_m | cluster\_10 | coexpression\_cluster1 | 22 | 10.85 | 148 | **wtswwttTGCACACACrhmvrg** | **CYBKDYGTGTGTGCAAAWWSAW** |  |  | | coexpression\_cluster1\_m23\_cluster1\_motif23\_r | cluster1\_motif23\_r | cluster\_2 | coexpression\_cluster1 | 12 | 8.21 | 1798 | **wwATGTTGTAww** | **WWTACAACATWW** |  |  | | coexpression\_cluster1\_m30\_cluster1\_motif30\_m | cluster1\_motif30\_m | cluster\_4 | coexpression\_cluster1 | 15 | 8.24 | 544 | **+++RYAYATRCAYA+** | **+TrTGyATrTry+++** |  |  | | coexpression\_cluster1\_m3\_cluster1\_motif3\_r | cluster1\_motif3\_r | cluster\_1 | coexpression\_cluster1 | 15 | 8.87 | 1735 | **WWWATGTCTTTWW++** | **++wwAAAgACATwww** |  |  | | coexpression\_cluster1\_m42\_cluster1\_motif42\_m | cluster1\_motif42\_m | cluster\_13 | coexpression\_cluster1 | 15 | 9.94 | 25 | **msvwmCCCCMmmTAt** | **ATAKKKGGGGKWBSK** |  |  | | coexpression\_cluster1\_m41\_cluster1\_motif41\_m | cluster1\_motif41\_m | cluster\_1 | coexpression\_cluster1 | 15 | 14.58 | 549 | **+TTAWTTTWTYTT++** | **++AARAwAAAwTAA+** |  |  | | coexpression\_cluster1\_m2\_cluster1\_motif2\_r | cluster1\_motif2\_r | cluster\_1 | coexpression\_cluster1 | 15 | 8.90 | 1983 | **wwATAgACAAwwww+** | **+WWWWTTGTCTATWW** |  |  | | coexpression\_cluster1\_m1\_cluster1\_motif1\_r | cluster1\_motif1\_r | cluster\_1 | coexpression\_cluster1 | 15 | 9.10 | 2614 | **WWWWTTGTCTTTWW+** | **+wwAAAgACAAwwww** |  |  | | coexpression\_cluster1\_m40\_cluster1\_motif40\_m | cluster1\_motif40\_m | cluster\_1 | coexpression\_cluster1 | 15 | 14.58 | 198 | **AAATAAATaAAATAA** | **TTATTTTATTTATTT** |  |  | | coexpression\_cluster1\_m35\_cluster1\_motif35\_m | cluster1\_motif35\_m | cluster\_11 | coexpression\_cluster1 | 6 | 6.26 | 168 | **GCATGC** | **GCATGC** |  |  | | coexpression\_cluster1\_m16\_cluster1\_motif16\_r | cluster1\_motif16\_r | cluster\_3 | coexpression\_cluster1 | 24 | 9.39 | 5262 | **+++++++++WWYATTTTTAAATWW** | **wwATTTAAAAATrww+++++++++** |  |  | | coexpression\_cluster1\_m10\_cluster1\_motif10\_r | cluster1\_motif10\_r | cluster\_4 | coexpression\_cluster1 | 15 | 8.75 | 1740 | **+++atACACACATww** | **WWATGTGTGTAT+++** |  |  | | coexpression\_cluster1\_m20\_cluster1\_motif20\_r | cluster1\_motif20\_r | cluster\_2 | coexpression\_cluster1 | 12 | 9.05 | 201 | **++WWTGCAACWW** | **wwGTTGCAww++** |  |  | | coexpression\_cluster1\_m13\_cluster1\_motif13\_r | cluster1\_motif13\_r | cluster\_8 | coexpression\_cluster1 | 10 | 9.23 | 216 | **wwTCTGAAww** | **WWTTCAGAWW** |  |  | | coexpression\_cluster1\_m6\_cluster1\_motif6\_r | cluster1\_motif6\_r | cluster\_3 | coexpression\_cluster1 | 24 | 13.05 | 3274 | **wwATrAAmAAAAATwTtTAAATww** | **WWATTTAAAWATTTTTKTTYATWW** |  |  | | coexpression\_cluster1\_m22\_cluster1\_motif22\_r | cluster1\_motif22\_r | cluster\_3 | coexpression\_cluster1 | 24 | 13.12 | 2727 | **wwTAmAAmAAAAATwTtTAAAtww** | **WWATTTAAAWATTTTTKTTKTAWW** |  |  | | coexpression\_cluster1\_m9\_cluster1\_motif9\_r | cluster1\_motif9\_r | cluster\_1 | coexpression\_cluster1 | 15 | 8.35 | 943 | **wwAAAGACAcww+++** | **+++WWGTGTCTTTWW** |  |  | | coexpression\_cluster1\_m34\_cluster1\_motif34\_m | cluster1\_motif34\_m | cluster\_1 | coexpression\_cluster1 | 15 | 14.58 | 264 | **+AAAAAAAAAAAAAC** | **GTTTTTTTTTTTTT+** |  |  | | coexpression\_cluster1\_m7\_cluster1\_motif7\_r | cluster1\_motif7\_r | cluster\_1 | coexpression\_cluster1 | 15 | 8.62 | 2153 | **WWWAAGACAAWWWW+** | **+wwwwTTGTcTTwww** |  |  | | coexpression\_cluster1\_m44\_cluster1\_motif44\_m | cluster1\_motif44\_m | cluster\_12 | coexpression\_cluster1 | 15 | 20.79 | 6 | **CATTTCATGACCCAT** | **ATGGGTCATGAAATG** |  |  | | coexpression\_cluster1\_m24\_cluster1\_motif24\_r | cluster1\_motif24\_r | cluster\_2 | coexpression\_cluster1 | 12 | 9.22 | 2255 | **+WWTGTTTTWW+** | **+wwAAAACAww+** |  |  | | coexpression\_cluster1\_m27\_cluster1\_motif27\_m | cluster1\_motif27\_m | cluster\_4 | coexpression\_cluster1 | 15 | 15.49 | 269 | **TATATrTAyATATA+** | **+TATATRTAYATATA** |  |  |  - Click on the column names to change the order of the data. - Write the name of one cluster, collection or a pattern in the *Search* window. | | **Heatmap View**  Heatmap View Distance table | PDF | |

**Additional Files**

## Exported files

| File | Description |
| --- | --- |
| **Pairwise comparison** | This table shows the pairwise comparison between all the input motifs using different metrics. This is the *compare-matrices* result. |
| **Matrix description** | This table shows information of each input motif. |
| **Cluster-wise Motif Files [zip]** | One TRANSFAC file is generated for each cluster including all the input motifs grouped in the corresponding cluster. |
| **Root motifs** | A file with the root matrix of each cluster. |
| **Motif collections per cluster** | A tab separated file containing the counts of motif of each collection on each cluster. |
| **Clusters** | A tab separated file containing the clusters and their correspondng motifs. |
| **Central motifs IDs** | A tab separated file containing the central motif, that one who is the most similar to all the others motif in the same cluster. |
| **Central motifs (in TRANSFAC format)** | A transfac file containing the central motif, that one who is the most similar to all the others motif within the same cluster. |
| **Input motifs** | The input motifs analyzed with matrix-clustering. |

- **References**
  1. Castro-Mondragon JA et al.  RSAT matrix-clustering: dynamic exploration and redundancy reduction of transcription factor binding motif collections. doi: 10.1093/nar/gkx314; Nucleic Acids Research (2017)
  2. **Last RSAT release:** Nguyen NTT et al. RSAT 2018: regulatory sequence analysis tools 20th anniversary. Nucleic Acids Research (2018).
- **Additional Information**
  - Jacques van Helden course
- **Contact**
  - Jaime Castro-Mondragon - **j.a.c.mondragon@ncmm.uio.no**
  - Morgane Thomas-Chollier - **mthomas@biologie.ens.fr**
  - Jacques van Helden - **Jacques.van-Helden@univ-amu.fr**
