# Supplementary material for: Transcriptome analysis reveals a de novo DNA element that may interact with chromatin-associated proteins in Plasmodium berghei during erythrocytic development
Source: Sci Rep. 2025 May 28;15:18621. doi: 10.1038/s41598-025-03586-4 (PMC12120095; doi:10.1038/s41598-025-03586-4)
Supplement: Supplementary file 1 — Supplementary Information 1. [file 41598_2025_3586_MOESM1_ESM.zip › Supplementary File S1/Clustering/matrix-clustering_SUMMARY_cluster3.html]

matrix-clustering coexpression\_cluster3


# RSAT - matrix-clustering result

### Analysis: coexpression\_cluster3 (08/09/2023 12:48)

### Command

```
matrix-clustering  -v 1 -max_matrices 300 -matrix coexpression_cluster3 $RSAT/public_html/tmp/www-data/2023/09/08/matrix-clustering_2023-09-08.124743_XczLfy/matrix-clustering_query_matrices.transfac transfac -hclust_method average -calc sum -title coexpression_cluster3 -metric_build_tree Ncor -lth w 5 -lth cor 0.6 -lth Ncor 0.4 -quick -label_in_tree name -return json,heatmap -o $RSAT/public_html/tmp/www-data/2023/09/08/matrix-clustering_2023-09-08.124743_XczLfy/matrix-clustering
```


## Results Summary

|  |  |  |  |  |  |  |  |  |  |  |
| --- | --- | --- | --- | --- | --- | --- | --- | --- | --- | --- |
| Nb Input motifs | Nb Input collections | Nb Clusters Found | Download   Root motifs | Complete results [zip] | Parameters used  |  |  |  | | --- | --- | --- | | Linkage method | Similarity metric | Thresholds to partition the tree | | | |
| 44 | 1 | 9 | **Download** | Download | |  |  |  | | --- | --- | --- | | average | Ncor | **Ncor** = 0.4 **cor** = 0.6 | |

|  |  |
| --- | --- |
|  | **Logo Forest (dynamic browsing)**  **Logo Forest (rapid overview - low image quality)**  cluster\_1  cluster\_2  cluster\_3  cluster\_4  cluster\_5  cluster\_6  cluster\_7  cluster\_8  cluster\_9 |
|  | |  |  |  |  |  |  |  |  |  |  | | --- | --- | --- | --- | --- | --- | --- | --- | --- | --- | | **Clusters Summary**  Clusters Summary Table  | Root Motif | Root Motif (Reverse) | Cluster ID | # Motifs | Motif\_Name::Collection | Number of Motifs by Collection | Root Motif (transfac Format) | | --- | --- | --- | --- | --- | --- | --- | |  | | --- | |  | | cluster\_1 | 18 | cluster3\_motif19\_r::coexpression\_cluster3:: cluster3\_motif36\_m::coexpression\_cluster3:: cluster3\_motif8\_r::coexpression\_cluster3:: cluster3\_motif3\_r::coexpression\_cluster3:: cluster3\_motif18\_r::coexpression\_cluster3:: cluster3\_motif5\_r::coexpression\_cluster3:: cluster3\_motif21\_r::coexpression\_cluster3:: cluster3\_motif15\_r::coexpression\_cluster3:: cluster3\_motif10\_r::coexpression\_cluster3:: cluster3\_motif4\_r::coexpression\_cluster3:: cluster3\_motif14\_r::coexpression\_cluster3:: cluster3\_motif13\_r::coexpression\_cluster3:: cluster3\_motif1\_r::coexpression\_cluster3:: cluster3\_motif7\_r::coexpression\_cluster3:: cluster3\_motif9\_r::coexpression\_cluster3:: cluster3\_motif16\_r::coexpression\_cluster3:: cluster3\_motif22\_m::coexpression\_cluster3:: cluster3\_motif23\_m::coexpression\_cluster3:: | coexpression\_cluster3::18\_motifs | Show matrix |
| cluster\_2 | 4 | cluster3\_motif35\_m::coexpression\_cluster3:: cluster3\_motif44\_m::coexpression\_cluster3:: cluster3\_motif40\_m::coexpression\_cluster3:: cluster3\_motif42\_m::coexpression\_cluster3:: | coexpression\_cluster3::4\_motifs | Show matrix | | |
| cluster\_3 | 2 | cluster3\_motif33\_m::coexpression\_cluster3:: cluster3\_motif34\_m::coexpression\_cluster3:: | coexpression\_cluster3::2\_motifs | Show matrix | | |
| cluster\_4 | 6 | cluster3\_motif41\_m::coexpression\_cluster3:: cluster3\_motif30\_m::coexpression\_cluster3:: cluster3\_motif28\_m::coexpression\_cluster3:: cluster3\_motif6\_r::coexpression\_cluster3:: cluster3\_motif11\_r::coexpression\_cluster3:: cluster3\_motif12\_r::coexpression\_cluster3:: | coexpression\_cluster3::6\_motifs | Show matrix | | |
| cluster\_5 | 7 | cluster3\_motif32\_m::coexpression\_cluster3:: cluster3\_motif31\_m::coexpression\_cluster3:: cluster3\_motif2\_r::coexpression\_cluster3:: cluster3\_motif25\_m::coexpression\_cluster3:: cluster3\_motif29\_m::coexpression\_cluster3:: cluster3\_motif37\_m::coexpression\_cluster3:: cluster3\_motif39\_m::coexpression\_cluster3:: | coexpression\_cluster3::7\_motifs | Show matrix | | |
| cluster\_6 | 3 | cluster3\_motif27\_m::coexpression\_cluster3:: cluster3\_motif24\_m::coexpression\_cluster3:: cluster3\_motif26\_m::coexpression\_cluster3:: | coexpression\_cluster3::3\_motifs | Show matrix | | |
| cluster\_7 | 2 | cluster3\_motif17\_r::coexpression\_cluster3:: cluster3\_motif43\_m::coexpression\_cluster3:: | coexpression\_cluster3::2\_motifs | Show matrix | | |
| cluster\_8 | 1 | cluster3\_motif20\_r::coexpression\_cluster3 | coexpression\_cluster3 : 1 motif | Show matrix | | |
| cluster\_9 | 1 | cluster3\_motif38\_m::coexpression\_cluster3 | coexpression\_cluster3 : 1 motif | Show matrix | | |

- Note: by default the table displays the first 50 clusters. Click on the *Show X entries* button to display more (or less) clusters.
- Click on the column names to change the order of the data.
- Use the *Search* window to find your motif (s) of interest.

| **Individual Cluster View**  Individual Cluster View **Show All**  **Hide All**  **cluster\_1**  **cluster\_2**  **cluster\_3**  **cluster\_4**  **cluster\_5**  **cluster\_6**  **cluster\_7**  **cluster\_8**  **cluster\_9**  **Display Logo Trees**   **cluster\_1**    **cluster\_2**    **cluster\_3**    **cluster\_4**    **cluster\_5**    **cluster\_6**    **cluster\_7**    **cluster\_8**    **cluster\_9**  **Display Branch-Motifs** **cluster\_1**   | Branch Motifs | | | | | | --- | --- | --- | --- | --- | | Node | Consensus | Logo | Logo (Reverse) | Collections | Matrix (Transfac format) | IC | Nb sites | | Node 1 | **-wwTATAkACAtaww------** |  |  | coexpression\_cluster3::2\_motifs | PSSM | 8.75 | 6081 | | --- | --- | --- | --- | --- | --- | --- | --- | | Node 2 | **--wwaTrCACACAcwww----** |  |  | coexpression\_cluster3::2\_motifs | PSSM | 9.69 | 1566 | | Node 3 | **-wwTATAgAcAAAwwww----** |  |  | coexpression\_cluster3::2\_motifs | PSSM | 9.21 | 7123 | | Node 4 | **-wwTATAgAcAAAwwww----** |  |  | coexpression\_cluster3::3\_motifs | PSSM | 8.97 | 9166 | | Node 5 | **-wwtATAkACAwaww------** |  |  | coexpression\_cluster3::3\_motifs | PSSM | 8.50 | 8249 | | Node 6 | **--wwwwArAmAAAAAww----** |  |  | coexpression\_cluster3::2\_motifs | PSSM | 9.23 | 7775 | | Node 7 | **-CATATryATATATATA----** |  |  | coexpression\_cluster3::2\_motifs | PSSM | 14.89 | 1170 | | Node 8 | **-wwTATAkACAwwww------** |  |  | coexpression\_cluster3::4\_motifs | PSSM | 8.23 | 12408 | | Node 9 | **-wwwwwAgAmAAAaaww----** |  |  | coexpression\_cluster3::5\_motifs | PSSM | 8.69 | 16941 | | Node 10 | **--wwaTrCACACAhwww----** |  |  | coexpression\_cluster3::3\_motifs | PSSM | 8.70 | 4377 | | Node 11 | **-wwwaTAcACACAhawww---** |  |  | coexpression\_cluster3::4\_motifs | PSSM | 8.92 | 6125 | | Node 12 | **-wwwATAgAcAAAwwww----** |  |  | coexpression\_cluster3::9\_motifs | PSSM | 8.22 | 29349 | | Node 13 | **aTwwATmTACAww--------** |  |  | coexpression\_cluster3::2\_motifs | PSSM | 8.95 | 4213 | | Node 14 | **-wwwATAgACAaAwwwww---** |  |  | coexpression\_cluster3::13\_motifs | PSSM | 8.03 | 35474 | | Node 15 | **-yATAyAyAyATrTrTrTAta** |  |  | coexpression\_cluster3::3\_motifs | PSSM | 13.67 | 1962 | | Node 16 | **-wwwATAgAcAaAwawwwAta** |  |  | coexpression\_cluster3::16\_motifs | PSSM | 9.26 | 37436 | | Node 17 | **awwwATAkACAwAwawwwAta** |  |  | coexpression\_cluster3::18\_motifs | PSSM | 9.44 | 41648 |  **cluster\_2**   | Branch Motifs | | | | | | --- | --- | --- | --- | --- | | Node | Consensus | Logo | Logo (Reverse) | Collections | Matrix (Transfac format) | IC | Nb sites | | Node 1 | **thwwrtgCACACACAytyrkaw-** |  |  | coexpression\_cluster3::2\_motifs | PSSM | 7.86 | 644 | | --- | --- | --- | --- | --- | --- | --- | --- | | Node 2 | **twwaawkCACACACaywwtgrwy** |  |  | coexpression\_cluster3::3\_motifs | PSSM | 7.84 | 1143 | | Node 3 | **wwwaawtyrCACACAywwwgrwy** |  |  | coexpression\_cluster3::4\_motifs | PSSM | 7.76 | 1481 |  **cluster\_3**   | Branch Motifs | | | | | | --- | --- | --- | --- | --- | | Node | Consensus | Logo | Logo (Reverse) | Collections | Matrix (Transfac format) | IC | Nb sites | | Node 1 | **WTATKAACAaktMAKA** |  |  | coexpression\_cluster3::2\_motifs | PSSM | 13.68 | 258 | | --- | --- | --- | --- | --- | --- | --- | --- |  **cluster\_4**   | Branch Motifs | | | | | | --- | --- | --- | --- | --- | | Node | Consensus | Logo | Logo (Reverse) | Collections | Matrix (Transfac format) | IC | Nb sites | | Node 1 | **-----wwAgCTAGCTAwwww--** |  |  | coexpression\_cluster3::2\_motifs | PSSM | 8.76 | 1495 | | --- | --- | --- | --- | --- | --- | --- | --- | | Node 2 | **----wwwAgCTAGCTAwwwww-** |  |  | coexpression\_cluster3::3\_motifs | PSSM | 9.04 | 1754 | | Node 3 | **----wwwAgcTAGCTAwwwww-** |  |  | coexpression\_cluster3::4\_motifs | PSSM | 9.05 | 1933 | | Node 4 | **----wwwAgCTAGCTAwwwww-** |  |  | coexpression\_cluster3::5\_motifs | PSSM | 9.11 | 2063 | | Node 5 | **yygtwwwAgCTAGCTAwwwwwr** |  |  | coexpression\_cluster3::6\_motifs | PSSM | 9.25 | 2196 |  **cluster\_5**   | Branch Motifs | | | | | | --- | --- | --- | --- | --- | | Node | Consensus | Logo | Logo (Reverse) | Collections | Matrix (Transfac format) | IC | Nb sites | | Node 1 | **AAAAAAArarAAwAA-** |  |  | coexpression\_cluster3::2\_motifs | PSSM | 12.02 | 1593 | | --- | --- | --- | --- | --- | --- | --- | --- | | Node 2 | **aaAAAArAmAAAaAaw** |  |  | coexpression\_cluster3::3\_motifs | PSSM | 10.39 | 10168 | | Node 3 | **aaAAAArAmAAAaAaw** |  |  | coexpression\_cluster3::4\_motifs | PSSM | 10.28 | 10539 | | Node 4 | **aaAAAArAmAAAaAaw** |  |  | coexpression\_cluster3::5\_motifs | PSSM | 10.21 | 10882 | | Node 5 | **-AAwTAAAAmAAAaw-** |  |  | coexpression\_cluster3::2\_motifs | PSSM | 11.03 | 1193 | | Node 6 | **aaAAAArAmAAAaAww** |  |  | coexpression\_cluster3::7\_motifs | PSSM | 9.80 | 12075 |  **cluster\_6**   | Branch Motifs | | | | | | --- | --- | --- | --- | --- | | Node | Consensus | Logo | Logo (Reverse) | Collections | Matrix (Transfac format) | IC | Nb sites | | Node 1 | **---TrCAYACAy** |  |  | coexpression\_cluster3::2\_motifs | PSSM | 8.99 | 1459 | | --- | --- | --- | --- | --- | --- | --- | --- | | Node 2 | **rTATrCAyACAy** |  |  | coexpression\_cluster3::3\_motifs | PSSM | 10.58 | 1762 |  **cluster\_7**   | Branch Motifs | | | | | | --- | --- | --- | --- | --- | | Node | Consensus | Logo | Logo (Reverse) | Collections | Matrix (Transfac format) | IC | Nb sites | | Node 1 | **yrtwtATTCTAwwc** |  |  | coexpression\_cluster3::2\_motifs | PSSM | 8.92 | 772 | | --- | --- | --- | --- | --- | --- | --- | --- |  **cluster\_8**   | Branch Motifs | | | | | | --- | --- | --- | --- | --- | | Node | Consensus | Logo | Logo (Reverse) | Collections | Matrix (Transfac format) | IC | Nb sites | | Singleton | **wtTCTTCAww** |  |  | coexpression\_cluster3 : 1 motif | PSSM | 9.20 | 326 | | --- | --- | --- | --- | --- | --- | --- | --- |  **cluster\_9**   | Branch Motifs | | | | | | --- | --- | --- | --- | --- | | Node | Consensus | Logo | Logo (Reverse) | Collections | Matrix (Transfac format) | IC | Nb sites | | Singleton | **aaytratrr** |  |  | coexpression\_cluster3 : 1 motif | PSSM | 9.47 | 692 | | --- | --- | --- | --- | --- | --- | --- | --- |   - **Each cluster has a different color.** - **Click on the upper buttons to display separately the information of each cluster.** - **Click on the circles at each tree to display its corresponding merged motifs.**   Definitions  - **Logo tree.**  This results shows the hierarchical tree with its logo alignment of one cluster. The logos are shown in Forward (left logo) and Reverse (right logo) orientation. - **Branch-motifs.**  On the trees are displayed the branch number that is the number in which the motifs were incorporated in the tree. The Branch-Motifs table shows the logo in both orientations and the link to the file in TRANSFAC format with the branch-motif. |
|  | |  |  |  |  |  |  |  |  |  |  |  |  |  |  |  |  |  |  |  |  |  |  |  |  |  |  |  |  |  |  |  |  |  |  |  |  |  |  |  |  |  |  |  |  |  |  |  |  |  |  |  |  |  |  |  |  |  |  |  |  |  |  |  |  |  |  |  |  |  |  |  |  |  |  |  |  |  |  |  |  |  |  |  |  |  |  |  |  |  |  |  |  |  |  |  |  |  |  |  |  |  |  |  |  |  |  |  |  |  |  |  |  |  |  |  |  |  |  |  |  |  |  |  |  |  |  |  |  |  |  |  |  |  |  |  |  |  |  |  |  |  |  |  |  |  |  |  |  |  |  |  |  |  |  |  |  |  |  |  |  |  |  |  |  |  |  |  |  |  |  |  |  |  |  |  |  |  |  |  |  |  |  |  |  |  |  |  |  |  |  |  |  |  |  |  |  |  |  |  |  |  |  |  |  |  |  |  |  |  |  |  |  |  |  |  |  |  |  |  |  |  |  |  |  |  |  |  |  |  |  |  |  |  |  |  |  |  |  |  |  |  |  |  |  |  |  |  |  |  |  |  |  |  |  |  |  |  |  |  |  |  |  |  |  |  |  |  |  |  |  |  |  |  |  |  |  |  |  |  |  |  |  |  |  |  |  |  |  |  |  |  |  |  |  |  |  |  |  |  |  |  |  |  |  |  |  |  |  |  |  |  |  |  |  |  |  |  |  |  |  |  |  |  |  |  |  |  |  |  |  |  |  |  |  |  |  |  |  |  |  |  |  |  |  |  |  |  |  |  |  |  |  |  |  |  |  |  |  |  |  |  |  |  |  |  |  |  |  |  |  |  |  |  |  |  |  |  |  |  |  |  |  |  |  |  |  |  |  |  |  |  |  |  |  |  |  |  |  |  |  |  |  |  |  |  |  |  |  |  |  |  |  |  |  |  |  |  |  |  |  |  |  |  |  |  |  |  |  |  |  |  |  |  |  |  |  |  |  |  |  |  |  |  |  |  |  |  |  |  |  |  |  |  |  |  |  |  |  |  |  |  |  |  |  |  |  |  |  |  |  |  |  |  |  |  |  |  |  |  |  |  |  |  |  |  |  |  |  |  |  |  |  |  |  |  |  | | --- | --- | --- | --- | --- | --- | --- | --- | --- | --- | --- | --- | --- | --- | --- | --- | --- | --- | --- | --- | --- | --- | --- | --- | --- | --- | --- | --- | --- | --- | --- | --- | --- | --- | --- | --- | --- | --- | --- | --- | --- | --- | --- | --- | --- | --- | --- | --- | --- | --- | --- | --- | --- | --- | --- | --- | --- | --- | --- | --- | --- | --- | --- | --- | --- | --- | --- | --- | --- | --- | --- | --- | --- | --- | --- | --- | --- | --- | --- | --- | --- | --- | --- | --- | --- | --- | --- | --- | --- | --- | --- | --- | --- | --- | --- | --- | --- | --- | --- | --- | --- | --- | --- | --- | --- | --- | --- | --- | --- | --- | --- | --- | --- | --- | --- | --- | --- | --- | --- | --- | --- | --- | --- | --- | --- | --- | --- | --- | --- | --- | --- | --- | --- | --- | --- | --- | --- | --- | --- | --- | --- | --- | --- | --- | --- | --- | --- | --- | --- | --- | --- | --- | --- | --- | --- | --- | --- | --- | --- | --- | --- | --- | --- | --- | --- | --- | --- | --- | --- | --- | --- | --- | --- | --- | --- | --- | --- | --- | --- | --- | --- | --- | --- | --- | --- | --- | --- | --- | --- | --- | --- | --- | --- | --- | --- | --- | --- | --- | --- | --- | --- | --- | --- | --- | --- | --- | --- | --- | --- | --- | --- | --- | --- | --- | --- | --- | --- | --- | --- | --- | --- | --- | --- | --- | --- | --- | --- | --- | --- | --- | --- | --- | --- | --- | --- | --- | --- | --- | --- | --- | --- | --- | --- | --- | --- | --- | --- | --- | --- | --- | --- | --- | --- | --- | --- | --- | --- | --- | --- | --- | --- | --- | --- | --- | --- | --- | --- | --- | --- | --- | --- | --- | --- | --- | --- | --- | --- | --- | --- | --- | --- | --- | --- | --- | --- | --- | --- | --- | --- | --- | --- | --- | --- | --- | --- | --- | --- | --- | --- | --- | --- | --- | --- | --- | --- | --- | --- | --- | --- | --- | --- | --- | --- | --- | --- | --- | --- | --- | --- | --- | --- | --- | --- | --- | --- | --- | --- | --- | --- | --- | --- | --- | --- | --- | --- | --- | --- | --- | --- | --- | --- | --- | --- | --- | --- | --- | --- | --- | --- | --- | --- | --- | --- | --- | --- | --- | --- | --- | --- | --- | --- | --- | --- | --- | --- | --- | --- | --- | --- | --- | --- | --- | --- | --- | --- | --- | --- | --- | --- | --- | --- | --- | --- | --- | --- | --- | --- | --- | --- | --- | --- | --- | --- | --- | --- | --- | --- | --- | --- | --- | --- | --- | --- | --- | --- | --- | --- | --- | --- | --- | --- | --- | --- | --- | --- | --- | --- | --- | --- | --- | --- | --- | --- | --- | --- | --- | --- | --- | --- | --- | --- | --- | --- | --- | --- | --- | --- | --- | --- | --- | --- | --- | --- | --- | --- | --- | --- | --- | --- | --- | --- | --- | --- | --- | --- | --- | --- | --- | --- | --- | --- | --- | --- | --- | --- | --- | --- | --- | --- | --- | --- | --- | --- | --- | --- | --- | --- | --- | --- | --- | --- | --- | --- | --- | --- | --- | --- | --- | --- | --- | --- | --- | --- | --- | --- | --- | | **Individual Motif View**  Individual Motif View  | Motif id | Motif name | Cluster | Collection | Width | IC | Number of sites | Consensus | Consensus (Rev) | Logo | Logo (Rev) | | --- | --- | --- | --- | --- | --- | --- | --- | --- | --- | --- | | coexpression\_cluster3\_m30\_cluster3\_motif30\_m | cluster3\_motif30\_m | cluster\_4 | coexpression\_cluster3 | 22 | 10.18 | 130 | **+++++TRGCTAGYTRT++++++** | **++++++ayARCTAGCyA+++++** |  |  | | coexpression\_cluster3\_m41\_cluster3\_motif41\_m | cluster3\_motif41\_m | cluster\_4 | coexpression\_cluster3 | 22 | 9.77 | 133 | **YAKMGYBAGCTAGCTRWYACRR** | **yygtrwyAGCTAGCTvrckmtr** |  |  | | coexpression\_cluster3\_m38\_cluster3\_motif38\_m | cluster3\_motif38\_m | cluster\_9 | coexpression\_cluster3 | 9 | 9.47 | 692 | **AATTAATAA** | **TTATTAATT** |  |  | | coexpression\_cluster3\_m10\_cluster3\_motif10\_r | cluster3\_motif10\_r | cluster\_1 | coexpression\_cluster3 | 21 | 8.93 | 2783 | **+wwTATAkACAwwww++++++** | **++++++WWWWTGTMTATAWW+** |  |  | | coexpression\_cluster3\_m17\_cluster3\_motif17\_r | cluster3\_motif17\_r | cluster\_7 | coexpression\_cluster3 | 14 | 9.22 | 684 | **+++wwATTCTAww+** | **+WWTAGAATWW+++** |  |  | | coexpression\_cluster3\_m26\_cluster3\_motif26\_m | cluster3\_motif26\_m | cluster\_6 | coexpression\_cluster3 | 12 | 8.68 | 1000 | **+++TrCAyACA+** | **+TGTRTGYA+++** |  |  | | coexpression\_cluster3\_m29\_cluster3\_motif29\_m | cluster3\_motif29\_m | cluster\_5 | coexpression\_cluster3 | 16 | 16.21 | 593 | **AAAAAAAAAAAATAA+** | **+TTATTTTTTTTTTTT** |  |  | | coexpression\_cluster3\_m24\_cluster3\_motif24\_m | cluster3\_motif24\_m | cluster\_6 | coexpression\_cluster3 | 12 | 9.68 | 459 | **+++TGCACACAY** | **rTGTGTGCA+++** |  |  | | coexpression\_cluster3\_m19\_cluster3\_motif19\_r | cluster3\_motif19\_r | cluster\_1 | coexpression\_cluster3 | 21 | 7.77 | 3909 | **++wwATmTACAww++++++++** | **++++++++WWTGTAKATWW++** |  |  | | coexpression\_cluster3\_m33\_cluster3\_motif33\_m | cluster3\_motif33\_m | cluster\_3 | coexpression\_cluster3 | 16 | 13.54 | 111 | **WTATKAACAaktCAK+** | **+MTGAMTTGTTMATAW** |  |  | | coexpression\_cluster3\_m43\_cluster3\_motif43\_m | cluster3\_motif43\_m | cluster\_7 | coexpression\_cluster3 | 14 | 9.18 | 88 | **YRTTTGTTCTAYGC** | **gcrTAGAACAAayr** |  |  | | coexpression\_cluster3\_m16\_cluster3\_motif16\_r | cluster3\_motif16\_r | cluster\_1 | coexpression\_cluster3 | 21 | 12.66 | 792 | **+TATAYAYAYATRTRTRTATA** | **taTAyAyAyATrTrTrTAta+** |  |  | | coexpression\_cluster3\_m32\_cluster3\_motif32\_m | cluster3\_motif32\_m | cluster\_5 | coexpression\_cluster3 | 16 | 10.98 | 344 | **++TTTTTTCTCTTTT+** | **+AAAAGAGAAaAAa++** |  |  | | coexpression\_cluster3\_m31\_cluster3\_motif31\_m | cluster3\_motif31\_m | cluster\_5 | coexpression\_cluster3 | 16 | 11.80 | 372 | **TTTTCCTTTTTTT+++** | **+++AAAAAAAGGAAAA** |  |  | | coexpression\_cluster3\_m23\_cluster3\_motif23\_m | cluster3\_motif23\_m | cluster\_1 | coexpression\_cluster3 | 21 | 14.46 | 687 | **++ATATryATATATATA++++** | **++++TATATATATRYATAT++** |  |  | | coexpression\_cluster3\_m4\_cluster3\_motif4\_r | cluster3\_motif4\_r | cluster\_1 | coexpression\_cluster3 | 21 | 8.85 | 3298 | **+wwtATAkACATAww++++++** | **++++++WWTATGTMTATAWW+** |  |  | | coexpression\_cluster3\_m18\_cluster3\_motif18\_r | cluster3\_motif18\_r | cluster\_1 | coexpression\_cluster3 | 21 | 9.78 | 628 | **++WWWTGCACACACWWW++++** | **++++wwwgTGTGTGcAwww++** |  |  | | coexpression\_cluster3\_m25\_cluster3\_motif25\_m | cluster3\_motif25\_m | cluster\_5 | coexpression\_cluster3 | 16 | 11.75 | 1000 | **aAAAAAArrrRAaAA+** | **+TTTTYYYYTTTTTTT** |  |  | | coexpression\_cluster3\_m36\_cluster3\_motif36\_m | cluster3\_motif36\_m | cluster\_1 | coexpression\_cluster3 | 21 | 8.90 | 304 | **aTGTAyrTACAt+++++++++** | **+++++++++ATGTAYRTACAT** |  |  | | coexpression\_cluster3\_m20\_cluster3\_motif20\_r | cluster3\_motif20\_r | cluster\_8 | coexpression\_cluster3 | 10 | 9.20 | 326 | **wtTCTTCAww** | **WWTGAAGAAW** |  |  | | coexpression\_cluster3\_m15\_cluster3\_motif15\_r | cluster3\_motif15\_r | cluster\_1 | coexpression\_cluster3 | 21 | 8.98 | 2168 | **+WWWATAGACAAWWW++++++** | **++++++wwwTTGTcTATwww+** |  |  | | coexpression\_cluster3\_m9\_cluster3\_motif9\_r | cluster3\_motif9\_r | cluster\_1 | coexpression\_cluster3 | 21 | 9.50 | 3429 | **++WWTTARAMAAAAAWW++++** | **++++wwTTTTTkTyTAaww++** |  |  | | coexpression\_cluster3\_m13\_cluster3\_motif13\_r | cluster3\_motif13\_r | cluster\_1 | coexpression\_cluster3 | 21 | 9.34 | 2336 | **+wwwrTAgACAAAawww++++** | **++++WWWTTTTGTCTAYWWW+** |  |  | | coexpression\_cluster3\_m34\_cluster3\_motif34\_m | cluster3\_motif34\_m | cluster\_3 | coexpression\_cluster3 | 16 | 12.86 | 147 | **+TATKAACAAKTMAKA** | **TMTkamtTGTTmATA+** |  |  | | coexpression\_cluster3\_m42\_cluster3\_motif42\_m | cluster3\_motif42\_m | cluster\_2 | coexpression\_cluster3 | 23 | 7.11 | 456 | **thdwgwryACACACayyyrgrw+** | **+WYCYRRRTGTGTGTRYWCWHDA** |  |  | | coexpression\_cluster3\_m27\_cluster3\_motif27\_m | cluster3\_motif27\_m | cluster\_6 | coexpression\_cluster3 | 12 | 8.94 | 303 | **RTATGCATAY++** | **++rTATGCATAy** |  |  | | coexpression\_cluster3\_m5\_cluster3\_motif5\_r | cluster3\_motif5\_r | cluster\_1 | coexpression\_cluster3 | 21 | 9.83 | 938 | **++wwATrCACACAcwww++++** | **++++WWWGTGTGTGYATWW++** |  |  | | coexpression\_cluster3\_m37\_cluster3\_motif37\_m | cluster3\_motif37\_m | cluster\_5 | coexpression\_cluster3 | 16 | 12.38 | 731 | **+AATKAAAAAAAAaw+** | **+WTTTTTTTTTMATT+** |  |  | | coexpression\_cluster3\_m1\_cluster3\_motif1\_r | cluster3\_motif1\_r | cluster\_1 | coexpression\_cluster3 | 21 | 9.41 | 4787 | **+wwTATAkAcAAAwwww++++** | **++++WWWWTTTGTMTATAWW+** |  |  | | coexpression\_cluster3\_m21\_cluster3\_motif21\_r | cluster3\_motif21\_r | cluster\_1 | coexpression\_cluster3 | 21 | 7.93 | 4159 | **+WWTATAKACAWW++++++++** | **++++++++wwtGTmTATAww+** |  |  | | coexpression\_cluster3\_m40\_cluster3\_motif40\_m | cluster3\_motif40\_m | cluster\_2 | coexpression\_cluster3 | 23 | 11.81 | 189 | **TrtwwtgCRCACACACrwrkmw+** | **+WKMYWYGTGTGTGYGCAWWAYA** |  |  | | coexpression\_cluster3\_m8\_cluster3\_motif8\_r | cluster3\_motif8\_r | cluster\_1 | coexpression\_cluster3 | 21 | 10.11 | 1748 | **+WWWTGTGTGTMTATAWW+++** | **+++wwTATAkACAcAcAwww+** |  |  | | coexpression\_cluster3\_m14\_cluster3\_motif14\_r | cluster3\_motif14\_r | cluster\_1 | coexpression\_cluster3 | 21 | 9.11 | 2043 | **+WWWATAGACAAWDWWW++++** | **++++wwwhwTTGTcTATwww+** |  |  | | coexpression\_cluster3\_m6\_cluster3\_motif6\_r | cluster3\_motif6\_r | cluster\_4 | coexpression\_cluster3 | 22 | 10.33 | 259 | **++++WWAAMTAGCTAGCYAWW+** | **+wwTrgCTAGCTAktTww++++** |  |  | | coexpression\_cluster3\_m28\_cluster3\_motif28\_m | cluster3\_motif28\_m | cluster\_4 | coexpression\_cluster3 | 22 | 10.13 | 179 | **+++++++AAHTAGCTADTT+++** | **+++AahTAGCTAdtT+++++++** |  |  | | coexpression\_cluster3\_m35\_cluster3\_motif35\_m | cluster3\_motif35\_m | cluster\_2 | coexpression\_cluster3 | 23 | 10.73 | 339 | **CYYKRYGTGTGTGCAAAWTSAW+** | **+wtsawttTGCACACACrymrrg** |  |  | | coexpression\_cluster3\_m12\_cluster3\_motif12\_r | cluster3\_motif12\_r | cluster\_4 | coexpression\_cluster3 | 22 | 9.03 | 682 | **+++++wwAGCTAGCTAAwww++** | **++WWWTTAGCTAGCTWW+++++** |  |  | | coexpression\_cluster3\_m44\_cluster3\_motif44\_m | cluster3\_motif44\_m | cluster\_2 | coexpression\_cluster3 | 23 | 9.69 | 500 | **MWWAMMTCACACACATWWTGRAY** | **rtycawwatGTGTGTgakktwwk** |  |  | | coexpression\_cluster3\_m11\_cluster3\_motif11\_r | cluster3\_motif11\_r | cluster\_4 | coexpression\_cluster3 | 22 | 8.78 | 813 | **+++++wwAgCTAGCTATww+++** | **+++WWATAGCTAGCTWW+++++** |  |  | | coexpression\_cluster3\_m3\_cluster3\_motif3\_r | cluster3\_motif3\_r | cluster\_1 | coexpression\_cluster3 | 21 | 8.32 | 2811 | **+++WWWTGTGTGTATW+++++** | **+++++watACACACAwww+++** |  |  | | coexpression\_cluster3\_m22\_cluster3\_motif22\_m | cluster3\_motif22\_m | cluster\_1 | coexpression\_cluster3 | 21 | 14.36 | 483 | **+CATAYRYATATATAT+++++** | **+++++ATATATATrYRTATG+** |  |  | | coexpression\_cluster3\_m7\_cluster3\_motif7\_r | cluster3\_motif7\_r | cluster\_1 | coexpression\_cluster3 | 21 | 9.79 | 4346 | **++WWWAARAMAAAAAWW++++** | **++++wwTTTTTkTyTTwww++** |  |  | | coexpression\_cluster3\_m2\_cluster3\_motif2\_r | cluster3\_motif2\_r | cluster\_5 | coexpression\_cluster3 | 16 | 10.55 | 8575 | **WWTTTTTKTYTTTTTW** | **waAAAArAmAAAaaww** |  |  | | coexpression\_cluster3\_m39\_cluster3\_motif39\_m | cluster3\_motif39\_m | cluster\_5 | coexpression\_cluster3 | 16 | 11.26 | 462 | **+AAATAAAACAA++++** | **++++TTGTTTTATTT+** |  |  |  - Click on the column names to change the order of the data. - Write the name of one cluster, collection or a pattern in the *Search* window. | | **Heatmap View**  Heatmap View Distance table | PDF | |

**Additional Files**

## Exported files

| File | Description |
| --- | --- |
| **Pairwise comparison** | This table shows the pairwise comparison between all the input motifs using different metrics. This is the *compare-matrices* result. |
| **Matrix description** | This table shows information of each input motif. |
| **Cluster-wise Motif Files [zip]** | One TRANSFAC file is generated for each cluster including all the input motifs grouped in the corresponding cluster. |
| **Root motifs** | A file with the root matrix of each cluster. |
| **Motif collections per cluster** | A tab separated file containing the counts of motif of each collection on each cluster. |
| **Clusters** | A tab separated file containing the clusters and their correspondng motifs. |
| **Central motifs IDs** | A tab separated file containing the central motif, that one who is the most similar to all the others motif in the same cluster. |
| **Central motifs (in TRANSFAC format)** | A transfac file containing the central motif, that one who is the most similar to all the others motif within the same cluster. |
| **Input motifs** | The input motifs analyzed with matrix-clustering. |

- **References**
  1. Castro-Mondragon JA et al.  RSAT matrix-clustering: dynamic exploration and redundancy reduction of transcription factor binding motif collections. doi: 10.1093/nar/gkx314; Nucleic Acids Research (2017)
  2. **Last RSAT release:** Nguyen NTT et al. RSAT 2018: regulatory sequence analysis tools 20th anniversary. Nucleic Acids Research (2018).
- **Additional Information**
  - Jacques van Helden course
- **Contact**
  - Jaime Castro-Mondragon - **j.a.c.mondragon@ncmm.uio.no**
  - Morgane Thomas-Chollier - **mthomas@biologie.ens.fr**
  - Jacques van Helden - **Jacques.van-Helden@univ-amu.fr**
