# Supplementary material for: Transcriptome analysis reveals a de novo DNA element that may interact with chromatin-associated proteins in Plasmodium berghei during erythrocytic development
Source: Sci Rep. 2025 May 28;15:18621. doi: 10.1038/s41598-025-03586-4 (PMC12120095; doi:10.1038/s41598-025-03586-4)
Supplement: Supplementary file 2 — Supplementary Information 2. [file 41598_2025_3586_MOESM2_ESM.zip › Supplementary File S2/Clustering/matrix-clustering_SUMMARY_cluster1_1.html]

matrix-clustering coexpression\_cluster1\_1


# RSAT - matrix-clustering result

### Analysis: coexpression\_cluster1\_1 (08/09/2023 14:47)

### Command

```
matrix-clustering  -v 1 -max_matrices 300 -matrix coexpression_cluster1_1 $RSAT/public_html/tmp/www-data/2023/09/08/matrix-clustering_2023-09-08.144739_G7IEX9/matrix-clustering_query_matrices.transfac transfac -hclust_method average -calc sum -title coexpression_cluster1_1 -metric_build_tree Ncor -lth w 5 -lth cor 0.6 -lth Ncor 0.4 -quick -label_in_tree name -return json,heatmap -o $RSAT/public_html/tmp/www-data/2023/09/08/matrix-clustering_2023-09-08.144739_G7IEX9/matrix-clustering
```


## Results Summary

|  |  |  |  |  |  |  |  |  |  |  |
| --- | --- | --- | --- | --- | --- | --- | --- | --- | --- | --- |
| Nb Input motifs | Nb Input collections | Nb Clusters Found | Download   Root motifs | Complete results [zip] | Parameters used  |  |  |  | | --- | --- | --- | | Linkage method | Similarity metric | Thresholds to partition the tree | | | |
| 23 | 1 | 12 | **Download** | Download | |  |  |  | | --- | --- | --- | | average | Ncor | **Ncor** = 0.4 **cor** = 0.6 | |

|  |  |
| --- | --- |
|  | **Logo Forest (dynamic browsing)**  **Logo Forest (rapid overview - low image quality)**  cluster\_1  cluster\_2  cluster\_3  cluster\_4  cluster\_5  cluster\_6  cluster\_7  cluster\_8  cluster\_9  cluster\_10  cluster\_11  cluster\_12 |
|  | |  |  |  |  |  |  |  |  |  |  | | --- | --- | --- | --- | --- | --- | --- | --- | --- | --- | | **Clusters Summary**  Clusters Summary Table  | Root Motif | Root Motif (Reverse) | Cluster ID | # Motifs | Motif\_Name::Collection | Number of Motifs by Collection | Root Motif (transfac Format) | | --- | --- | --- | --- | --- | --- | --- | |  | | --- | |  | | cluster\_1 | 4 | cluster1\_1\_motif16\_m::coexpression\_cluster1\_1:: cluster1\_1\_motif6\_r::coexpression\_cluster1\_1:: cluster1\_1\_motif12\_r::coexpression\_cluster1\_1:: cluster1\_1\_motif2\_r::coexpression\_cluster1\_1:: | coexpression\_cluster1\_1::4\_motifs | Show matrix |
| cluster\_2 | 4 | cluster1\_1\_motif1\_r::coexpression\_cluster1\_1:: cluster1\_1\_motif11\_r::coexpression\_cluster1\_1:: cluster1\_1\_motif3\_r::coexpression\_cluster1\_1:: cluster1\_1\_motif4\_r::coexpression\_cluster1\_1:: | coexpression\_cluster1\_1::4\_motifs | Show matrix | | |
| cluster\_3 | 4 | cluster1\_1\_motif5\_r::coexpression\_cluster1\_1:: cluster1\_1\_motif8\_r::coexpression\_cluster1\_1:: cluster1\_1\_motif13\_m::coexpression\_cluster1\_1:: cluster1\_1\_motif14\_m::coexpression\_cluster1\_1:: | coexpression\_cluster1\_1::4\_motifs | Show matrix | | |
| cluster\_4 | 3 | cluster1\_1\_motif10\_r::coexpression\_cluster1\_1:: cluster1\_1\_motif7\_r::coexpression\_cluster1\_1:: cluster1\_1\_motif9\_r::coexpression\_cluster1\_1:: | coexpression\_cluster1\_1::3\_motifs | Show matrix | | |
| cluster\_5 | 1 | cluster1\_1\_motif15\_m::coexpression\_cluster1\_1 | coexpression\_cluster1\_1 : 1 motif | Show matrix | | |
| cluster\_6 | 1 | cluster1\_1\_motif19\_m::coexpression\_cluster1\_1 | coexpression\_cluster1\_1 : 1 motif | Show matrix | | |
| cluster\_7 | 1 | cluster1\_1\_motif22\_m::coexpression\_cluster1\_1 | coexpression\_cluster1\_1 : 1 motif | Show matrix | | |
| cluster\_8 | 1 | cluster1\_1\_motif23\_m::coexpression\_cluster1\_1 | coexpression\_cluster1\_1 : 1 motif | Show matrix | | |
| cluster\_9 | 1 | cluster1\_1\_motif17\_m::coexpression\_cluster1\_1 | coexpression\_cluster1\_1 : 1 motif | Show matrix | | |
| cluster\_10 | 1 | cluster1\_1\_motif20\_m::coexpression\_cluster1\_1 | coexpression\_cluster1\_1 : 1 motif | Show matrix | | |
| cluster\_11 | 1 | cluster1\_1\_motif21\_m::coexpression\_cluster1\_1 | coexpression\_cluster1\_1 : 1 motif | Show matrix | | |
| cluster\_12 | 1 | cluster1\_1\_motif18\_m::coexpression\_cluster1\_1 | coexpression\_cluster1\_1 : 1 motif | Show matrix | | |

- Note: by default the table displays the first 50 clusters. Click on the *Show X entries* button to display more (or less) clusters.
- Click on the column names to change the order of the data.
- Use the *Search* window to find your motif (s) of interest.

| **Individual Cluster View**  Individual Cluster View **Show All**  **Hide All**  **cluster\_1**  **cluster\_2**  **cluster\_3**  **cluster\_4**  **cluster\_5**  **cluster\_6**  **cluster\_7**  **cluster\_8**  **cluster\_9**  **cluster\_10**  **cluster\_11**  **cluster\_12**  **Display Logo Trees**   **cluster\_1**    **cluster\_2**    **cluster\_3**    **cluster\_4**    **cluster\_5**    **cluster\_6**    **cluster\_7**    **cluster\_8**    **cluster\_9**    **cluster\_10**    **cluster\_11**    **cluster\_12**  **Display Branch-Motifs** **cluster\_1**   | Branch Motifs | | | | | | --- | --- | --- | --- | --- | | Node | Consensus | Logo | Logo (Reverse) | Collections | Matrix (Transfac format) | IC | Nb sites | | Node 1 | **wwTGCATGCAww--** |  |  | coexpression\_cluster1\_1::2\_motifs | PSSM | 8.21 | 673 | | --- | --- | --- | --- | --- | --- | --- | --- | | Node 2 | **wwTGCATGCAtwww** |  |  | coexpression\_cluster1\_1::3\_motifs | PSSM | 8.53 | 1058 | | Node 3 | **wwTGCATGCAtwww** |  |  | coexpression\_cluster1\_1::4\_motifs | PSSM | 8.64 | 1171 |  **cluster\_2**   | Branch Motifs | | | | | | --- | --- | --- | --- | --- | | Node | Consensus | Logo | Logo (Reverse) | Collections | Matrix (Transfac format) | IC | Nb sites | | Node 1 | **wwwwATTTAAATTATAATTTAwwww--** |  |  | coexpression\_cluster1\_1::2\_motifs | PSSM | 13.54 | 2263 | | --- | --- | --- | --- | --- | --- | --- | --- | | Node 2 | **wwwwATTTAAATTATAATTTAwwwwww** |  |  | coexpression\_cluster1\_1::3\_motifs | PSSM | 14.06 | 3341 | | Node 3 | **wwwwwTwTAAwTTAwAwTwTawwwwww** |  |  | coexpression\_cluster1\_1::4\_motifs | PSSM | 12.66 | 5673 |  **cluster\_3**   | Branch Motifs | | | | | | --- | --- | --- | --- | --- | | Node | Consensus | Logo | Logo (Reverse) | Collections | Matrix (Transfac format) | IC | Nb sites | | Node 1 | **wwACATmTwww---** |  |  | coexpression\_cluster1\_1::2\_motifs | PSSM | 7.06 | 1545 | | --- | --- | --- | --- | --- | --- | --- | --- | | Node 2 | **ATAyATATATATrT** |  |  | coexpression\_cluster1\_1::2\_motifs | PSSM | 14.39 | 134 | | Node 3 | **wwACATmTwtwTrT** |  |  | coexpression\_cluster1\_1::4\_motifs | PSSM | 9.55 | 1679 |  **cluster\_4**   | Branch Motifs | | | | | | --- | --- | --- | --- | --- | | Node | Consensus | Logo | Logo (Reverse) | Collections | Matrix (Transfac format) | IC | Nb sites | | Node 1 | **wwwTATTAmAww** |  |  | coexpression\_cluster1\_1::2\_motifs | PSSM | 7.86 | 3108 | | --- | --- | --- | --- | --- | --- | --- | --- | | Node 2 | **wwwtATwAaaww** |  |  | coexpression\_cluster1\_1::3\_motifs | PSSM | 6.52 | 5337 |  **cluster\_5**   | Branch Motifs | | | | | | --- | --- | --- | --- | --- | | Node | Consensus | Logo | Logo (Reverse) | Collections | Matrix (Transfac format) | IC | Nb sites | | Singleton | **aaaGaGaamaartSv** |  |  | coexpression\_cluster1\_1 : 1 motif | PSSM | 12.30 | 49 | | --- | --- | --- | --- | --- | --- | --- | --- |  **cluster\_6**   | Branch Motifs | | | | | | --- | --- | --- | --- | --- | | Node | Consensus | Logo | Logo (Reverse) | Collections | Matrix (Transfac format) | IC | Nb sites | | Singleton | **aaaattarCrCaaaa** |  |  | coexpression\_cluster1\_1 : 1 motif | PSSM | 15.03 | 42 | | --- | --- | --- | --- | --- | --- | --- | --- |  **cluster\_7**   | Branch Motifs | | | | | | --- | --- | --- | --- | --- | | Node | Consensus | Logo | Logo (Reverse) | Collections | Matrix (Transfac format) | IC | Nb sites | | Singleton | **CCtGSMtGaGSGGyK** |  |  | coexpression\_cluster1\_1 : 1 motif | PSSM | 13.58 | 13 | | --- | --- | --- | --- | --- | --- | --- | --- |  **cluster\_8**   | Branch Motifs | | | | | | --- | --- | --- | --- | --- | | Node | Consensus | Logo | Logo (Reverse) | Collections | Matrix (Transfac format) | IC | Nb sites | | Singleton | **SrYttCMyGaCSSat** |  |  | coexpression\_cluster1\_1 : 1 motif | PSSM | 13.95 | 14 | | --- | --- | --- | --- | --- | --- | --- | --- |  **cluster\_9**   | Branch Motifs | | | | | | --- | --- | --- | --- | --- | | Node | Consensus | Logo | Logo (Reverse) | Collections | Matrix (Transfac format) | IC | Nb sites | | Singleton | **tSrGttCraRtCy** |  |  | coexpression\_cluster1\_1 : 1 motif | PSSM | 14.30 | 11 | | --- | --- | --- | --- | --- | --- | --- | --- |  **cluster\_10**   | Branch Motifs | | | | | | --- | --- | --- | --- | --- | | Node | Consensus | Logo | Logo (Reverse) | Collections | Matrix (Transfac format) | IC | Nb sites | | Singleton | **SSSGGSaVmYSBSSG** |  |  | coexpression\_cluster1\_1 : 1 motif | PSSM | 10.45 | 19 | | --- | --- | --- | --- | --- | --- | --- | --- |  **cluster\_11**   | Branch Motifs | | | | | | --- | --- | --- | --- | --- | | Node | Consensus | Logo | Logo (Reverse) | Collections | Matrix (Transfac format) | IC | Nb sites | | Singleton | **CGSCCSKaMGGtwRS** |  |  | coexpression\_cluster1\_1 : 1 motif | PSSM | 13.67 | 14 | | --- | --- | --- | --- | --- | --- | --- | --- |  **cluster\_12**   | Branch Motifs | | | | | | --- | --- | --- | --- | --- | | Node | Consensus | Logo | Logo (Reverse) | Collections | Matrix (Transfac format) | IC | Nb sites | | Singleton | **GCCSCC** |  |  | coexpression\_cluster1\_1 : 1 motif | PSSM | 6.22 | 60 | | --- | --- | --- | --- | --- | --- | --- | --- |   - **Each cluster has a different color.** - **Click on the upper buttons to display separately the information of each cluster.** - **Click on the circles at each tree to display its corresponding merged motifs.**   Definitions  - **Logo tree.**  This results shows the hierarchical tree with its logo alignment of one cluster. The logos are shown in Forward (left logo) and Reverse (right logo) orientation. - **Branch-motifs.**  On the trees are displayed the branch number that is the number in which the motifs were incorporated in the tree. The Branch-Motifs table shows the logo in both orientations and the link to the file in TRANSFAC format with the branch-motif. |
|  | |  |  |  |  |  |  |  |  |  |  |  |  |  |  |  |  |  |  |  |  |  |  |  |  |  |  |  |  |  |  |  |  |  |  |  |  |  |  |  |  |  |  |  |  |  |  |  |  |  |  |  |  |  |  |  |  |  |  |  |  |  |  |  |  |  |  |  |  |  |  |  |  |  |  |  |  |  |  |  |  |  |  |  |  |  |  |  |  |  |  |  |  |  |  |  |  |  |  |  |  |  |  |  |  |  |  |  |  |  |  |  |  |  |  |  |  |  |  |  |  |  |  |  |  |  |  |  |  |  |  |  |  |  |  |  |  |  |  |  |  |  |  |  |  |  |  |  |  |  |  |  |  |  |  |  |  |  |  |  |  |  |  |  |  |  |  |  |  |  |  |  |  |  |  |  |  |  |  |  |  |  |  |  |  |  |  |  |  |  |  |  |  |  |  |  |  |  |  |  |  |  |  |  |  |  |  |  |  |  |  |  |  |  |  |  |  |  |  |  |  |  |  |  |  |  |  |  |  |  |  |  |  |  |  |  |  |  |  |  |  |  |  |  |  |  |  |  |  |  |  |  |  |  |  |  |  |  |  |  |  |  |  |  |  |  | | --- | --- | --- | --- | --- | --- | --- | --- | --- | --- | --- | --- | --- | --- | --- | --- | --- | --- | --- | --- | --- | --- | --- | --- | --- | --- | --- | --- | --- | --- | --- | --- | --- | --- | --- | --- | --- | --- | --- | --- | --- | --- | --- | --- | --- | --- | --- | --- | --- | --- | --- | --- | --- | --- | --- | --- | --- | --- | --- | --- | --- | --- | --- | --- | --- | --- | --- | --- | --- | --- | --- | --- | --- | --- | --- | --- | --- | --- | --- | --- | --- | --- | --- | --- | --- | --- | --- | --- | --- | --- | --- | --- | --- | --- | --- | --- | --- | --- | --- | --- | --- | --- | --- | --- | --- | --- | --- | --- | --- | --- | --- | --- | --- | --- | --- | --- | --- | --- | --- | --- | --- | --- | --- | --- | --- | --- | --- | --- | --- | --- | --- | --- | --- | --- | --- | --- | --- | --- | --- | --- | --- | --- | --- | --- | --- | --- | --- | --- | --- | --- | --- | --- | --- | --- | --- | --- | --- | --- | --- | --- | --- | --- | --- | --- | --- | --- | --- | --- | --- | --- | --- | --- | --- | --- | --- | --- | --- | --- | --- | --- | --- | --- | --- | --- | --- | --- | --- | --- | --- | --- | --- | --- | --- | --- | --- | --- | --- | --- | --- | --- | --- | --- | --- | --- | --- | --- | --- | --- | --- | --- | --- | --- | --- | --- | --- | --- | --- | --- | --- | --- | --- | --- | --- | --- | --- | --- | --- | --- | --- | --- | --- | --- | --- | --- | --- | --- | --- | --- | --- | --- | --- | --- | --- | --- | --- | --- | --- | --- | --- | --- | --- | --- | --- | --- | --- | --- | --- | --- | --- | --- | --- | --- | --- | --- | --- | | **Individual Motif View**  Individual Motif View  | Motif id | Motif name | Cluster | Collection | Width | IC | Number of sites | Consensus | Consensus (Rev) | Logo | Logo (Rev) | | --- | --- | --- | --- | --- | --- | --- | --- | --- | --- | --- | | coexpression\_cluster1\_1\_m21\_cluster1\_1\_motif21\_m | cluster1\_1\_motif21\_m | cluster\_11 | coexpression\_cluster1\_1 | 15 | 13.67 | 14 | **CGkyYSkAmGGTwrS** | **SYWACCKTMSRRMCG** |  |  | | coexpression\_cluster1\_1\_m7\_cluster1\_1\_motif7\_r | cluster1\_1\_motif7\_r | cluster\_4 | coexpression\_cluster1\_1 | 12 | 7.65 | 2194 | **+wwTATTAyAww** | **WWTRTAATAWW+** |  |  | | coexpression\_cluster1\_1\_m20\_cluster1\_1\_motif20\_m | cluster1\_1\_motif20\_m | cluster\_10 | coexpression\_cluster1\_1 | 15 | 10.45 | 19 | **sysGGswvAYCkscG** | **CGSMGRTBWSCCSRS** |  |  | | coexpression\_cluster1\_1\_m16\_cluster1\_1\_motif16\_m | cluster1\_1\_motif16\_m | cluster\_1 | coexpression\_cluster1\_1 | 14 | 8.56 | 113 | **+++ATGCATGY+++** | **+++rCATGCAT+++** |  |  | | coexpression\_cluster1\_1\_m3\_cluster1\_1\_motif3\_r | cluster1\_1\_motif3\_r | cluster\_2 | coexpression\_cluster1\_1 | 27 | 13.50 | 1169 | **+wwTATTTAAATTwwAATTTAwwww++** | **++WWWWTAAATTWWAATTTAAATAWW+** |  |  | | coexpression\_cluster1\_1\_m12\_cluster1\_1\_motif12\_r | cluster1\_1\_motif12\_r | cluster\_1 | coexpression\_cluster1\_1 | 14 | 7.86 | 409 | **wwtGCATGCAww++** | **++WWTGCATGCAWW** |  |  | | coexpression\_cluster1\_1\_m1\_cluster1\_1\_motif1\_r | cluster1\_1\_motif1\_r | cluster\_2 | coexpression\_cluster1\_1 | 27 | 11.66 | 2332 | **++WWWWATAATTTAAATTATWWWW+++** | **+++wwwwATAATTTAAATTATwwww++** |  |  | | coexpression\_cluster1\_1\_m5\_cluster1\_1\_motif5\_r | cluster1\_1\_motif5\_r | cluster\_3 | coexpression\_cluster1\_1 | 14 | 7.52 | 767 | **wwACATyTTww+++** | **+++WWAARATGTWW** |  |  | | coexpression\_cluster1\_1\_m19\_cluster1\_1\_motif19\_m | cluster1\_1\_motif19\_m | cluster\_6 | coexpression\_cluster1\_1 | 15 | 15.03 | 42 | **AAAATwAAmamAWAA** | **TTWTKTKTTWATTTT** |  |  | | coexpression\_cluster1\_1\_m13\_cluster1\_1\_motif13\_m | cluster1\_1\_motif13\_m | cluster\_3 | coexpression\_cluster1\_1 | 14 | 15.52 | 66 | **ATATATATATAT++** | **++ATATATATATAT** |  |  | | coexpression\_cluster1\_1\_m15\_cluster1\_1\_motif15\_m | cluster1\_1\_motif15\_m | cluster\_5 | coexpression\_cluster1\_1 | 15 | 12.30 | 49 | **AAArAGAAAAAATga** | **TCATTTTTTCTYTTT** |  |  | | coexpression\_cluster1\_1\_m11\_cluster1\_1\_motif11\_r | cluster1\_1\_motif11\_r | cluster\_2 | coexpression\_cluster1\_1 | 27 | 14.38 | 1078 | **WWWWATWTAAATTATAATTTAAWWWWW** | **wwwwwTTAAATTATAATTTAWATwwww** |  |  | | coexpression\_cluster1\_1\_m23\_cluster1\_1\_motif23\_m | cluster1\_1\_motif23\_m | cluster\_8 | coexpression\_cluster1\_1 | 15 | 13.95 | 14 | **cAYTTCMTGACcCAT** | **ATGGGTCAKGAARTG** |  |  | | coexpression\_cluster1\_1\_m2\_cluster1\_1\_motif2\_r | cluster1\_1\_motif2\_r | cluster\_1 | coexpression\_cluster1\_1 | 14 | 8.95 | 264 | **WWTGCATGCAWW++** | **++wwTGCATGCAww** |  |  | | coexpression\_cluster1\_1\_m8\_cluster1\_1\_motif8\_r | cluster1\_1\_motif8\_r | cluster\_3 | coexpression\_cluster1\_1 | 14 | 7.79 | 778 | **wwACATmTAww+++** | **+++WWTAKATGTWW** |  |  | | coexpression\_cluster1\_1\_m4\_cluster1\_1\_motif4\_r | cluster1\_1\_motif4\_r | cluster\_2 | coexpression\_cluster1\_1 | 27 | 13.58 | 1094 | **wwwwATTTAAATTATAATTTAwww+++** | **+++WWWTAAATTATAATTTAAATWWWW** |  |  | | coexpression\_cluster1\_1\_m18\_cluster1\_1\_motif18\_m | cluster1\_1\_motif18\_m | cluster\_12 | coexpression\_cluster1\_1 | 6 | 6.22 | 60 | **dCCCCC** | **GGGGGH** |  |  | | coexpression\_cluster1\_1\_m10\_cluster1\_1\_motif10\_r | cluster1\_1\_motif10\_r | cluster\_4 | coexpression\_cluster1\_1 | 12 | 7.57 | 2229 | **WWTTTATCAWW+** | **+wwTgATAAAww** |  |  | | coexpression\_cluster1\_1\_m9\_cluster1\_1\_motif9\_r | cluster1\_1\_motif9\_r | cluster\_4 | coexpression\_cluster1\_1 | 12 | 9.66 | 914 | **wwwTATTAAww+** | **+WWTTAATAWWW** |  |  | | coexpression\_cluster1\_1\_m6\_cluster1\_1\_motif6\_r | cluster1\_1\_motif6\_r | cluster\_1 | coexpression\_cluster1\_1 | 14 | 8.87 | 385 | **WWTGCATGCATTWW** | **wwAATGCATGCAww** |  |  | | coexpression\_cluster1\_1\_m22\_cluster1\_1\_motif22\_m | cluster1\_1\_motif22\_m | cluster\_7 | coexpression\_cluster1\_1 | 15 | 13.58 | 13 | **mmTGCmTGArcGRTK** | **MAYCGYTCAKGCAKK** |  |  | | coexpression\_cluster1\_1\_m14\_cluster1\_1\_motif14\_m | cluster1\_1\_motif14\_m | cluster\_3 | coexpression\_cluster1\_1 | 14 | 14.20 | 68 | **ATACATATATATrT** | **AYATATATATGTAT** |  |  | | coexpression\_cluster1\_1\_m17\_cluster1\_1\_motif17\_m | cluster1\_1\_motif17\_m | cluster\_9 | coexpression\_cluster1\_1 | 13 | 14.30 | 11 | **WsRGTTCRArTCY** | **RGAYTYGAACYSW** |  |  |  - Click on the column names to change the order of the data. - Write the name of one cluster, collection or a pattern in the *Search* window. | | **Heatmap View**  Heatmap View Distance table | PDF | |

**Additional Files**

## Exported files

| File | Description |
| --- | --- |
| **Pairwise comparison** | This table shows the pairwise comparison between all the input motifs using different metrics. This is the *compare-matrices* result. |
| **Matrix description** | This table shows information of each input motif. |
| **Cluster-wise Motif Files [zip]** | One TRANSFAC file is generated for each cluster including all the input motifs grouped in the corresponding cluster. |
| **Root motifs** | A file with the root matrix of each cluster. |
| **Motif collections per cluster** | A tab separated file containing the counts of motif of each collection on each cluster. |
| **Clusters** | A tab separated file containing the clusters and their correspondng motifs. |
| **Central motifs IDs** | A tab separated file containing the central motif, that one who is the most similar to all the others motif in the same cluster. |
| **Central motifs (in TRANSFAC format)** | A transfac file containing the central motif, that one who is the most similar to all the others motif within the same cluster. |
| **Input motifs** | The input motifs analyzed with matrix-clustering. |

- **References**
  1. Castro-Mondragon JA et al.  RSAT matrix-clustering: dynamic exploration and redundancy reduction of transcription factor binding motif collections. doi: 10.1093/nar/gkx314; Nucleic Acids Research (2017)
  2. **Last RSAT release:** Nguyen NTT et al. RSAT 2018: regulatory sequence analysis tools 20th anniversary. Nucleic Acids Research (2018).
- **Additional Information**
  - Jacques van Helden course
- **Contact**
  - Jaime Castro-Mondragon - **j.a.c.mondragon@ncmm.uio.no**
  - Morgane Thomas-Chollier - **mthomas@biologie.ens.fr**
  - Jacques van Helden - **Jacques.van-Helden@univ-amu.fr**
