# Supplementary material for: Transcriptome analysis reveals a de novo DNA element that may interact with chromatin-associated proteins in Plasmodium berghei during erythrocytic development
Source: Sci Rep. 2025 May 28;15:18621. doi: 10.1038/s41598-025-03586-4 (PMC12120095; doi:10.1038/s41598-025-03586-4)
Supplement: Supplementary file 2 — Supplementary Information 2. [file 41598_2025_3586_MOESM2_ESM.zip › Supplementary File S2/Clustering/matrix-clustering_SUMMARY_cluster1_12.html]

matrix-clustering coexpression\_cluster1\_12


# RSAT - matrix-clustering result

### Analysis: coexpression\_cluster1\_12 (08/09/2023 15:17)

### Command

```
matrix-clustering  -v 1 -max_matrices 300 -matrix coexpression_cluster1_12 $RSAT/public_html/tmp/www-data/2023/09/08/matrix-clustering_2023-09-08.151652_SAQQkK/matrix-clustering_query_matrices.transfac transfac -hclust_method average -calc sum -title coexpression_cluster1_12 -metric_build_tree Ncor -lth w 5 -lth cor 0.6 -lth Ncor 0.4 -quick -label_in_tree name -return json,heatmap -o $RSAT/public_html/tmp/www-data/2023/09/08/matrix-clustering_2023-09-08.151652_SAQQkK/matrix-clustering
```


## Results Summary

|  |  |  |  |  |  |  |  |  |  |  |
| --- | --- | --- | --- | --- | --- | --- | --- | --- | --- | --- |
| Nb Input motifs | Nb Input collections | Nb Clusters Found | Download   Root motifs | Complete results [zip] | Parameters used  |  |  |  | | --- | --- | --- | | Linkage method | Similarity metric | Thresholds to partition the tree | | | |
| 29 | 1 | 8 | **Download** | Download | |  |  |  | | --- | --- | --- | | average | Ncor | **Ncor** = 0.4 **cor** = 0.6 | |

|  |  |
| --- | --- |
|  | **Logo Forest (dynamic browsing)**  **Logo Forest (rapid overview - low image quality)**  cluster\_1  cluster\_2  cluster\_3  cluster\_4  cluster\_5  cluster\_6  cluster\_7  cluster\_8 |
|  | |  |  |  |  |  |  |  |  |  |  | | --- | --- | --- | --- | --- | --- | --- | --- | --- | --- | | **Clusters Summary**  Clusters Summary Table  | Root Motif | Root Motif (Reverse) | Cluster ID | # Motifs | Motif\_Name::Collection | Number of Motifs by Collection | Root Motif (transfac Format) | | --- | --- | --- | --- | --- | --- | --- | |  | | --- | |  | | cluster\_1 | 14 | cluster1\_12\_motif25\_m::coexpression\_cluster1\_12:: cluster1\_12\_motif19\_m::coexpression\_cluster1\_12:: cluster1\_12\_motif12\_r::coexpression\_cluster1\_12:: cluster1\_12\_motif3\_r::coexpression\_cluster1\_12:: cluster1\_12\_motif8\_r::coexpression\_cluster1\_12:: cluster1\_12\_motif15\_r::coexpression\_cluster1\_12:: cluster1\_12\_motif6\_r::coexpression\_cluster1\_12:: cluster1\_12\_motif4\_r::coexpression\_cluster1\_12:: cluster1\_12\_motif11\_r::coexpression\_cluster1\_12:: cluster1\_12\_motif1\_r::coexpression\_cluster1\_12:: cluster1\_12\_motif10\_r::coexpression\_cluster1\_12:: cluster1\_12\_motif2\_r::coexpression\_cluster1\_12:: cluster1\_12\_motif24\_m::coexpression\_cluster1\_12:: cluster1\_12\_motif7\_r::coexpression\_cluster1\_12:: | coexpression\_cluster1\_12::14\_motifs | Show matrix |
| cluster\_2 | 8 | cluster1\_12\_motif13\_r::coexpression\_cluster1\_12:: cluster1\_12\_motif16\_r::coexpression\_cluster1\_12:: cluster1\_12\_motif14\_r::coexpression\_cluster1\_12:: cluster1\_12\_motif9\_r::coexpression\_cluster1\_12:: cluster1\_12\_motif23\_m::coexpression\_cluster1\_12:: cluster1\_12\_motif5\_r::coexpression\_cluster1\_12:: cluster1\_12\_motif17\_m::coexpression\_cluster1\_12:: cluster1\_12\_motif18\_m::coexpression\_cluster1\_12:: | coexpression\_cluster1\_12::8\_motifs | Show matrix | | |
| cluster\_3 | 2 | cluster1\_12\_motif20\_m::coexpression\_cluster1\_12:: cluster1\_12\_motif27\_m::coexpression\_cluster1\_12:: | coexpression\_cluster1\_12::2\_motifs | Show matrix | | |
| cluster\_4 | 1 | cluster1\_12\_motif21\_m::coexpression\_cluster1\_12 | coexpression\_cluster1\_12 : 1 motif | Show matrix | | |
| cluster\_5 | 1 | cluster1\_12\_motif26\_m::coexpression\_cluster1\_12 | coexpression\_cluster1\_12 : 1 motif | Show matrix | | |
| cluster\_6 | 1 | cluster1\_12\_motif28\_m::coexpression\_cluster1\_12 | coexpression\_cluster1\_12 : 1 motif | Show matrix | | |
| cluster\_7 | 1 | cluster1\_12\_motif29\_m::coexpression\_cluster1\_12 | coexpression\_cluster1\_12 : 1 motif | Show matrix | | |
| cluster\_8 | 1 | cluster1\_12\_motif22\_m::coexpression\_cluster1\_12 | coexpression\_cluster1\_12 : 1 motif | Show matrix | | |

- Note: by default the table displays the first 50 clusters. Click on the *Show X entries* button to display more (or less) clusters.
- Click on the column names to change the order of the data.
- Use the *Search* window to find your motif (s) of interest.

| **Individual Cluster View**  Individual Cluster View **Show All**  **Hide All**  **cluster\_1**  **cluster\_2**  **cluster\_3**  **cluster\_4**  **cluster\_5**  **cluster\_6**  **cluster\_7**  **cluster\_8**  **Display Logo Trees**   **cluster\_1**    **cluster\_2**    **cluster\_3**    **cluster\_4**    **cluster\_5**    **cluster\_6**    **cluster\_7**    **cluster\_8**  **Display Branch-Motifs** **cluster\_1**   | Branch Motifs | | | | | | --- | --- | --- | --- | --- | | Node | Consensus | Logo | Logo (Reverse) | Collections | Matrix (Transfac format) | IC | Nb sites | | Node 1 | **wawAAArAmAAAAAAwww-** |  |  | coexpression\_cluster1\_12::2\_motifs | PSSM | 11.09 | 4358 | | --- | --- | --- | --- | --- | --- | --- | --- | | Node 2 | **wwAAAArAmAAAAAAwww-** |  |  | coexpression\_cluster1\_12::3\_motifs | PSSM | 10.99 | 5938 | | Node 3 | **wwwwTArAmAAAAAAwww-** |  |  | coexpression\_cluster1\_12::2\_motifs | PSSM | 10.23 | 2447 | | Node 4 | **----waAAAArAmATwww-** |  |  | coexpression\_cluster1\_12::2\_motifs | PSSM | 9.12 | 2560 | | Node 5 | **----waAAAArAmAAwwww** |  |  | coexpression\_cluster1\_12::2\_motifs | PSSM | 9.41 | 1984 | | Node 6 | **wwwAWArAmAAAAAAwww-** |  |  | coexpression\_cluster1\_12::5\_motifs | PSSM | 10.38 | 8385 | | Node 7 | **----waAAAArAmAAwwww** |  |  | coexpression\_cluster1\_12::3\_motifs | PSSM | 9.20 | 2691 | | Node 8 | **----waAAAArAmAwwwww** |  |  | coexpression\_cluster1\_12::5\_motifs | PSSM | 8.89 | 5251 | | Node 9 | **---wwAAAmAAAAAAwww-** |  |  | coexpression\_cluster1\_12::2\_motifs | PSSM | 9.72 | 4001 | | Node 10 | **wwwawAaAmAAAAAAwww-** |  |  | coexpression\_cluster1\_12::7\_motifs | PSSM | 10.22 | 12386 | | Node 11 | **wwwawAaAmAAAAAawwww** |  |  | coexpression\_cluster1\_12::12\_motifs | PSSM | 9.65 | 17637 | | Node 12 | **wwwawAaAmAAAAAawwww** |  |  | coexpression\_cluster1\_12::13\_motifs | PSSM | 9.65 | 17726 | | Node 13 | **wwwawAaAmAAAAAawwww** |  |  | coexpression\_cluster1\_12::14\_motifs | PSSM | 9.64 | 17836 |  **cluster\_2**   | Branch Motifs | | | | | | --- | --- | --- | --- | --- | | Node | Consensus | Logo | Logo (Reverse) | Collections | Matrix (Transfac format) | IC | Nb sites | | Node 1 | **awATRCACAcAtaw---** |  |  | coexpression\_cluster1\_12::2\_motifs | PSSM | 9.03 | 901 | | --- | --- | --- | --- | --- | --- | --- | --- | | Node 2 | **-tATrTATryATATATd** |  |  | coexpression\_cluster1\_12::2\_motifs | PSSM | 12.56 | 414 | | Node 3 | **awATGCACACATaw---** |  |  | coexpression\_cluster1\_12::3\_motifs | PSSM | 9.04 | 1125 | | Node 4 | **-taTATrTaTATATaTd** |  |  | coexpression\_cluster1\_12::3\_motifs | PSSM | 11.56 | 2273 | | Node 5 | **-taTATrTaTATATaTd** |  |  | coexpression\_cluster1\_12::4\_motifs | PSSM | 11.32 | 2372 | | Node 6 | **atATryryAyATAtaTd** |  |  | coexpression\_cluster1\_12::7\_motifs | PSSM | 10.00 | 3497 | | Node 7 | **ataTATrTrTATAtaTd** |  |  | coexpression\_cluster1\_12::8\_motifs | PSSM | 9.32 | 5019 |  **cluster\_3**   | Branch Motifs | | | | | | --- | --- | --- | --- | --- | | Node | Consensus | Logo | Logo (Reverse) | Collections | Matrix (Transfac format) | IC | Nb sites | | Node 1 | **wwywwtkyrCACACACrhvgrk** |  |  | coexpression\_cluster1\_12::2\_motifs | PSSM | 10.37 | 136 | | --- | --- | --- | --- | --- | --- | --- | --- |  **cluster\_4**   | Branch Motifs | | | | | | --- | --- | --- | --- | --- | | Node | Consensus | Logo | Logo (Reverse) | Collections | Matrix (Transfac format) | IC | Nb sites | | Singleton | **aGMtaGCYa** |  |  | coexpression\_cluster1\_12 : 1 motif | PSSM | 8.17 | 46 | | --- | --- | --- | --- | --- | --- | --- | --- |  **cluster\_5**   | Branch Motifs | | | | | | --- | --- | --- | --- | --- | | Node | Consensus | Logo | Logo (Reverse) | Collections | Matrix (Transfac format) | IC | Nb sites | | Singleton | **SSSbSsBaGCtaGCtSVCSsbS** |  |  | coexpression\_cluster1\_12 : 1 motif | PSSM | 9.86 | 59 | | --- | --- | --- | --- | --- | --- | --- | --- |  **cluster\_6**   | Branch Motifs | | | | | | --- | --- | --- | --- | --- | | Node | Consensus | Logo | Logo (Reverse) | Collections | Matrix (Transfac format) | IC | Nb sites | | Singleton | **GBGkGSGkGdG** |  |  | coexpression\_cluster1\_12 : 1 motif | PSSM | 7.73 | 34 | | --- | --- | --- | --- | --- | --- | --- | --- |  **cluster\_7**   | Branch Motifs | | | | | | --- | --- | --- | --- | --- | | Node | Consensus | Logo | Logo (Reverse) | Collections | Matrix (Transfac format) | IC | Nb sites | | Singleton | **SsssvsswyraGCCSrGvvssG** |  |  | coexpression\_cluster1\_12 : 1 motif | PSSM | 7.56 | 99 | | --- | --- | --- | --- | --- | --- | --- | --- |  **cluster\_8**   | Branch Motifs | | | | | | --- | --- | --- | --- | --- | | Node | Consensus | Logo | Logo (Reverse) | Collections | Matrix (Transfac format) | IC | Nb sites | | Singleton | **aaatCGGaa** |  |  | coexpression\_cluster1\_12 : 1 motif | PSSM | 8.61 | 110 | | --- | --- | --- | --- | --- | --- | --- | --- |   - **Each cluster has a different color.** - **Click on the upper buttons to display separately the information of each cluster.** - **Click on the circles at each tree to display its corresponding merged motifs.**   Definitions  - **Logo tree.**  This results shows the hierarchical tree with its logo alignment of one cluster. The logos are shown in Forward (left logo) and Reverse (right logo) orientation. - **Branch-motifs.**  On the trees are displayed the branch number that is the number in which the motifs were incorporated in the tree. The Branch-Motifs table shows the logo in both orientations and the link to the file in TRANSFAC format with the branch-motif. |
|  | |  |  |  |  |  |  |  |  |  |  |  |  |  |  |  |  |  |  |  |  |  |  |  |  |  |  |  |  |  |  |  |  |  |  |  |  |  |  |  |  |  |  |  |  |  |  |  |  |  |  |  |  |  |  |  |  |  |  |  |  |  |  |  |  |  |  |  |  |  |  |  |  |  |  |  |  |  |  |  |  |  |  |  |  |  |  |  |  |  |  |  |  |  |  |  |  |  |  |  |  |  |  |  |  |  |  |  |  |  |  |  |  |  |  |  |  |  |  |  |  |  |  |  |  |  |  |  |  |  |  |  |  |  |  |  |  |  |  |  |  |  |  |  |  |  |  |  |  |  |  |  |  |  |  |  |  |  |  |  |  |  |  |  |  |  |  |  |  |  |  |  |  |  |  |  |  |  |  |  |  |  |  |  |  |  |  |  |  |  |  |  |  |  |  |  |  |  |  |  |  |  |  |  |  |  |  |  |  |  |  |  |  |  |  |  |  |  |  |  |  |  |  |  |  |  |  |  |  |  |  |  |  |  |  |  |  |  |  |  |  |  |  |  |  |  |  |  |  |  |  |  |  |  |  |  |  |  |  |  |  |  |  |  |  |  |  |  |  |  |  |  |  |  |  |  |  |  |  |  |  |  |  |  |  |  |  |  |  |  |  |  |  |  |  |  |  |  |  |  |  |  |  |  |  |  |  |  |  |  |  |  |  |  |  |  |  |  |  |  |  |  |  |  |  |  |  |  |  |  |  |  | | --- | --- | --- | --- | --- | --- | --- | --- | --- | --- | --- | --- | --- | --- | --- | --- | --- | --- | --- | --- | --- | --- | --- | --- | --- | --- | --- | --- | --- | --- | --- | --- | --- | --- | --- | --- | --- | --- | --- | --- | --- | --- | --- | --- | --- | --- | --- | --- | --- | --- | --- | --- | --- | --- | --- | --- | --- | --- | --- | --- | --- | --- | --- | --- | --- | --- | --- | --- | --- | --- | --- | --- | --- | --- | --- | --- | --- | --- | --- | --- | --- | --- | --- | --- | --- | --- | --- | --- | --- | --- | --- | --- | --- | --- | --- | --- | --- | --- | --- | --- | --- | --- | --- | --- | --- | --- | --- | --- | --- | --- | --- | --- | --- | --- | --- | --- | --- | --- | --- | --- | --- | --- | --- | --- | --- | --- | --- | --- | --- | --- | --- | --- | --- | --- | --- | --- | --- | --- | --- | --- | --- | --- | --- | --- | --- | --- | --- | --- | --- | --- | --- | --- | --- | --- | --- | --- | --- | --- | --- | --- | --- | --- | --- | --- | --- | --- | --- | --- | --- | --- | --- | --- | --- | --- | --- | --- | --- | --- | --- | --- | --- | --- | --- | --- | --- | --- | --- | --- | --- | --- | --- | --- | --- | --- | --- | --- | --- | --- | --- | --- | --- | --- | --- | --- | --- | --- | --- | --- | --- | --- | --- | --- | --- | --- | --- | --- | --- | --- | --- | --- | --- | --- | --- | --- | --- | --- | --- | --- | --- | --- | --- | --- | --- | --- | --- | --- | --- | --- | --- | --- | --- | --- | --- | --- | --- | --- | --- | --- | --- | --- | --- | --- | --- | --- | --- | --- | --- | --- | --- | --- | --- | --- | --- | --- | --- | --- | --- | --- | --- | --- | --- | --- | --- | --- | --- | --- | --- | --- | --- | --- | --- | --- | --- | --- | --- | --- | --- | --- | --- | --- | --- | --- | --- | --- | --- | --- | --- | --- | --- | --- | --- | --- | --- | --- | --- | --- | --- | --- | --- | --- | --- | --- | --- | --- | --- | --- | --- | --- | --- | --- | --- | --- | --- | --- | --- | --- | --- | --- | --- | --- | --- | | **Individual Motif View**  Individual Motif View  | Motif id | Motif name | Cluster | Collection | Width | IC | Number of sites | Consensus | Consensus (Rev) | Logo | Logo (Rev) | | --- | --- | --- | --- | --- | --- | --- | --- | --- | --- | --- | | coexpression\_cluster1\_12\_m5\_cluster1\_12\_motif5\_r | cluster1\_12\_motif5\_r | cluster\_2 | coexpression\_cluster1\_12 | 17 | 10.62 | 1859 | **+waTATrTaTATAtw++** | **++WATATATAYATATW+** |  |  | | coexpression\_cluster1\_12\_m12\_cluster1\_12\_motif12\_r | cluster1\_12\_motif12\_r | cluster\_1 | coexpression\_cluster1\_12 | 19 | 9.19 | 957 | **++++WWAAAARACATTWW+** | **+wwAATgTyTTTTww++++** |  |  | | coexpression\_cluster1\_12\_m14\_cluster1\_12\_motif14\_r | cluster1\_12\_motif14\_r | cluster\_2 | coexpression\_cluster1\_12 | 17 | 9.44 | 320 | **awATrCACACAyaw+++** | **+++WTRTGTGTGYATWT** |  |  | | coexpression\_cluster1\_12\_m6\_cluster1\_12\_motif6\_r | cluster1\_12\_motif6\_r | cluster\_1 | coexpression\_cluster1\_12 | 19 | 9.59 | 1326 | **++++waAAAArAmAATww+** | **+WWATTKTYTTTTTW++++** |  |  | | coexpression\_cluster1\_12\_m28\_cluster1\_12\_motif28\_m | cluster1\_12\_motif28\_m | cluster\_6 | coexpression\_cluster1\_12 | 11 | 7.73 | 34 | **GkrkGsrtGwG** | **CWCAYSCMYMC** |  |  | | coexpression\_cluster1\_12\_m16\_cluster1\_12\_motif16\_r | cluster1\_12\_motif16\_r | cluster\_2 | coexpression\_cluster1\_12 | 17 | 9.59 | 224 | **+WTATGTGTGCAWW+++** | **+++wwTGCACACATaw+** |  |  | | coexpression\_cluster1\_12\_m24\_cluster1\_12\_motif24\_m | cluster1\_12\_motif24\_m | cluster\_1 | coexpression\_cluster1\_12 | 19 | 12.56 | 265 | **+++AAAAARAmrAAAAAA+** | **+TTTTTTYKTYTTTTT+++** |  |  | | coexpression\_cluster1\_12\_m7\_cluster1\_12\_motif7\_r | cluster1\_12\_motif7\_r | cluster\_1 | coexpression\_cluster1\_12 | 19 | 9.98 | 3736 | **+++wwAAAmAAAAAAwww+** | **+WWWTTTTTTKTTTWW+++** |  |  | | coexpression\_cluster1\_12\_m15\_cluster1\_12\_motif15\_r | cluster1\_12\_motif15\_r | cluster\_1 | coexpression\_cluster1\_12 | 19 | 9.53 | 658 | **++++wawAAAgACAAawww** | **WWWTTTGTCTTTWTW++++** |  |  | | coexpression\_cluster1\_12\_m27\_cluster1\_12\_motif27\_m | cluster1\_12\_motif27\_m | cluster\_3 | coexpression\_cluster1\_12 | 22 | 12.34 | 64 | **TrtwwtgCRCACACACrwrkmw** | **WKMYWYGTGTGTGYGCAWWAYA** |  |  | | coexpression\_cluster1\_12\_m13\_cluster1\_12\_motif13\_r | cluster1\_12\_motif13\_r | cluster\_2 | coexpression\_cluster1\_12 | 17 | 7.54 | 1522 | **++WWATGTGTAWW++++** | **++++wwTACACATww++** |  |  | | coexpression\_cluster1\_12\_m4\_cluster1\_12\_motif4\_r | cluster1\_12\_motif4\_r | cluster\_1 | coexpression\_cluster1\_12 | 19 | 11.20 | 1580 | **WWWTTTTTTKTYTTWTWW+** | **+wwAwAArAmAAAAAAwww** |  |  | | coexpression\_cluster1\_12\_m8\_cluster1\_12\_motif8\_r | cluster1\_12\_motif8\_r | cluster\_1 | coexpression\_cluster1\_12 | 19 | 8.89 | 707 | **++++WWWTGTCTTTTTW++** | **++waAAAAgACAwww++++** |  |  | | coexpression\_cluster1\_12\_m20\_cluster1\_12\_motif20\_m | cluster1\_12\_motif20\_m | cluster\_3 | coexpression\_cluster1\_12 | 22 | 11.05 | 72 | **wtswwttTGCACACACrhmvrg** | **CYBKDYGTGTGTGCAAAWWSAW** |  |  | | coexpression\_cluster1\_12\_m29\_cluster1\_12\_motif29\_m | cluster1\_12\_motif29\_m | cluster\_7 | coexpression\_cluster1\_12 | 22 | 7.56 | 99 | **rwawwwwwTAAGCCcArawwar** | **YTWWTYTGGGCTTAWWWWWTWY** |  |  | | coexpression\_cluster1\_12\_m9\_cluster1\_12\_motif9\_r | cluster1\_12\_motif9\_r | cluster\_2 | coexpression\_cluster1\_12 | 17 | 9.21 | 581 | **awATGCAcAyATww+++** | **+++WWATRTGTGCATWT** |  |  | | coexpression\_cluster1\_12\_m11\_cluster1\_12\_motif11\_r | cluster1\_12\_motif11\_r | cluster\_1 | coexpression\_cluster1\_12 | 19 | 11.10 | 1932 | **WWWAAARAMAAAAAAWWW+** | **+wwwTTTTTTkTyTTTwww** |  |  | | coexpression\_cluster1\_12\_m23\_cluster1\_12\_motif23\_m | cluster1\_12\_motif23\_m | cluster\_2 | coexpression\_cluster1\_12 | 17 | 13.63 | 99 | **++ATrCAYRYATATAT+** | **+ATATATRYRTGYAT++** |  |  | | coexpression\_cluster1\_12\_m10\_cluster1\_12\_motif10\_r | cluster1\_12\_motif10\_r | cluster\_1 | coexpression\_cluster1\_12 | 19 | 10.13 | 1149 | **+WWWTARAMAAAAAAWWW+** | **+wwwTTTTTTkTyTAwww+** |  |  | | coexpression\_cluster1\_12\_m19\_cluster1\_12\_motif19\_m | cluster1\_12\_motif19\_m | cluster\_1 | coexpression\_cluster1\_12 | 19 | 14.06 | 89 | **+++++TTATTAWTTTTTT+** | **+AAAaAAwTAATAA+++++** |  |  | | coexpression\_cluster1\_12\_m3\_cluster1\_12\_motif3\_r | cluster1\_12\_motif3\_r | cluster\_1 | coexpression\_cluster1\_12 | 19 | 9.72 | 1603 | **++++waAAAArAhATAww+** | **+WWTATDTYTTTTTW++++** |  |  | | coexpression\_cluster1\_12\_m2\_cluster1\_12\_motif2\_r | cluster1\_12\_motif2\_r | cluster\_1 | coexpression\_cluster1\_12 | 19 | 10.39 | 1298 | **wwwATAdAmAAAAAAwww+** | **+WWWTTTTTTKTHTATWWW** |  |  | | coexpression\_cluster1\_12\_m25\_cluster1\_12\_motif25\_m | cluster1\_12\_motif25\_m | cluster\_1 | coexpression\_cluster1\_12 | 19 | 12.81 | 110 | **+++CWTTTTTTTTAATT++** | **++AATTAAAAaAAAwg+++** |  |  | | coexpression\_cluster1\_12\_m26\_cluster1\_12\_motif26\_m | cluster1\_12\_motif26\_m | cluster\_5 | coexpression\_cluster1\_12 | 22 | 9.86 | 59 | **yygtrwyAGCTAGCTvrckmtg** | **CAKMGYBAGCTAGCTRWYACRR** |  |  | | coexpression\_cluster1\_12\_m21\_cluster1\_12\_motif21\_m | cluster1\_12\_motif21\_m | cluster\_4 | coexpression\_cluster1\_12 | 9 | 8.17 | 46 | **AGmTAGcyA** | **TRGCTAKCT** |  |  | | coexpression\_cluster1\_12\_m22\_cluster1\_12\_motif22\_m | cluster1\_12\_motif22\_m | cluster\_8 | coexpression\_cluster1\_12 | 9 | 8.61 | 110 | **AAATCGGAA** | **TTCCGATTT** |  |  | | coexpression\_cluster1\_12\_m1\_cluster1\_12\_motif1\_r | cluster1\_12\_motif1\_r | cluster\_1 | coexpression\_cluster1\_12 | 19 | 11.26 | 2426 | **waAAAArAmAAAAAAwww+** | **+WWWTTTTTTKTYTTTTTW** |  |  | | coexpression\_cluster1\_12\_m18\_cluster1\_12\_motif18\_m | cluster1\_12\_motif18\_m | cluster\_2 | coexpression\_cluster1\_12 | 17 | 13.36 | 79 | **+HATATATRYATATATD** | **hATATATryATATATd+** |  |  | | coexpression\_cluster1\_12\_m17\_cluster1\_12\_motif17\_m | cluster1\_12\_motif17\_m | cluster\_2 | coexpression\_cluster1\_12 | 17 | 12.84 | 335 | **+TATRTATRYATATAT+** | **+ATATATRyATAyATA+** |  |  |  - Click on the column names to change the order of the data. - Write the name of one cluster, collection or a pattern in the *Search* window. | | **Heatmap View**  Heatmap View Distance table | PDF | |

**Additional Files**

## Exported files

| File | Description |
| --- | --- |
| **Pairwise comparison** | This table shows the pairwise comparison between all the input motifs using different metrics. This is the *compare-matrices* result. |
| **Matrix description** | This table shows information of each input motif. |
| **Cluster-wise Motif Files [zip]** | One TRANSFAC file is generated for each cluster including all the input motifs grouped in the corresponding cluster. |
| **Root motifs** | A file with the root matrix of each cluster. |
| **Motif collections per cluster** | A tab separated file containing the counts of motif of each collection on each cluster. |
| **Clusters** | A tab separated file containing the clusters and their correspondng motifs. |
| **Central motifs IDs** | A tab separated file containing the central motif, that one who is the most similar to all the others motif in the same cluster. |
| **Central motifs (in TRANSFAC format)** | A transfac file containing the central motif, that one who is the most similar to all the others motif within the same cluster. |
| **Input motifs** | The input motifs analyzed with matrix-clustering. |

- **References**
  1. Castro-Mondragon JA et al.  RSAT matrix-clustering: dynamic exploration and redundancy reduction of transcription factor binding motif collections. doi: 10.1093/nar/gkx314; Nucleic Acids Research (2017)
  2. **Last RSAT release:** Nguyen NTT et al. RSAT 2018: regulatory sequence analysis tools 20th anniversary. Nucleic Acids Research (2018).
- **Additional Information**
  - Jacques van Helden course
- **Contact**
  - Jaime Castro-Mondragon - **j.a.c.mondragon@ncmm.uio.no**
  - Morgane Thomas-Chollier - **mthomas@biologie.ens.fr**
  - Jacques van Helden - **Jacques.van-Helden@univ-amu.fr**
