# Supplementary material for: Transcriptome analysis reveals a de novo DNA element that may interact with chromatin-associated proteins in Plasmodium berghei during erythrocytic development
Source: Sci Rep. 2025 May 28;15:18621. doi: 10.1038/s41598-025-03586-4 (PMC12120095; doi:10.1038/s41598-025-03586-4)
Supplement: Supplementary file 2 — Supplementary Information 2. [file 41598_2025_3586_MOESM2_ESM.zip › Supplementary File S2/Clustering/matrix-clustering_SUMMARY_cluster1_3.html]

matrix-clustering coexpression\_cluster1\_3


# RSAT - matrix-clustering result

### Analysis: coexpression\_cluster1\_3 (08/09/2023 14:59)

### Command

```
matrix-clustering  -v 1 -max_matrices 300 -matrix coexpression_cluster1_3 $RSAT/public_html/tmp/www-data/2023/09/08/matrix-clustering_2023-09-08.145929_1pV1Ah/matrix-clustering_query_matrices.transfac transfac -hclust_method average -calc sum -title coexpression_cluster1_3 -metric_build_tree Ncor -lth w 5 -lth cor 0.6 -lth Ncor 0.4 -quick -label_in_tree name -return json,heatmap -o $RSAT/public_html/tmp/www-data/2023/09/08/matrix-clustering_2023-09-08.145929_1pV1Ah/matrix-clustering
```


## Results Summary

|  |  |  |  |  |  |  |  |  |  |  |
| --- | --- | --- | --- | --- | --- | --- | --- | --- | --- | --- |
| Nb Input motifs | Nb Input collections | Nb Clusters Found | Download   Root motifs | Complete results [zip] | Parameters used  |  |  |  | | --- | --- | --- | | Linkage method | Similarity metric | Thresholds to partition the tree | | | |
| 30 | 1 | 10 | **Download** | Download | |  |  |  | | --- | --- | --- | | average | Ncor | **Ncor** = 0.4 **cor** = 0.6 | |

|  |  |
| --- | --- |
|  | **Logo Forest (dynamic browsing)**  **Logo Forest (rapid overview - low image quality)**  cluster\_1  cluster\_2  cluster\_3  cluster\_4  cluster\_5  cluster\_6  cluster\_7  cluster\_8  cluster\_9  cluster\_10 |
|  | |  |  |  |  |  |  |  |  |  |  | | --- | --- | --- | --- | --- | --- | --- | --- | --- | --- | | **Clusters Summary**  Clusters Summary Table  | Root Motif | Root Motif (Reverse) | Cluster ID | # Motifs | Motif\_Name::Collection | Number of Motifs by Collection | Root Motif (transfac Format) | | --- | --- | --- | --- | --- | --- | --- | |  | | --- | |  | | cluster\_1 | 11 | cluster1\_3\_motif10\_r::coexpression\_cluster1\_3:: cluster1\_3\_motif4\_r::coexpression\_cluster1\_3:: cluster1\_3\_motif23\_m::coexpression\_cluster1\_3:: cluster1\_3\_motif1\_r::coexpression\_cluster1\_3:: cluster1\_3\_motif12\_r::coexpression\_cluster1\_3:: cluster1\_3\_motif17\_r::coexpression\_cluster1\_3:: cluster1\_3\_motif15\_r::coexpression\_cluster1\_3:: cluster1\_3\_motif6\_r::coexpression\_cluster1\_3:: cluster1\_3\_motif26\_m::coexpression\_cluster1\_3:: cluster1\_3\_motif21\_m::coexpression\_cluster1\_3:: cluster1\_3\_motif24\_m::coexpression\_cluster1\_3:: | coexpression\_cluster1\_3::11\_motifs | Show matrix |
| cluster\_2 | 7 | cluster1\_3\_motif27\_m::coexpression\_cluster1\_3:: cluster1\_3\_motif20\_m::coexpression\_cluster1\_3:: cluster1\_3\_motif25\_m::coexpression\_cluster1\_3:: cluster1\_3\_motif16\_r::coexpression\_cluster1\_3:: cluster1\_3\_motif7\_r::coexpression\_cluster1\_3:: cluster1\_3\_motif2\_r::coexpression\_cluster1\_3:: cluster1\_3\_motif3\_r::coexpression\_cluster1\_3:: | coexpression\_cluster1\_3::7\_motifs | Show matrix | | |
| cluster\_3 | 5 | cluster1\_3\_motif13\_r::coexpression\_cluster1\_3:: cluster1\_3\_motif9\_r::coexpression\_cluster1\_3:: cluster1\_3\_motif14\_r::coexpression\_cluster1\_3:: cluster1\_3\_motif22\_m::coexpression\_cluster1\_3:: cluster1\_3\_motif5\_r::coexpression\_cluster1\_3:: | coexpression\_cluster1\_3::5\_motifs | Show matrix | | |
| cluster\_4 | 1 | cluster1\_3\_motif19\_r::coexpression\_cluster1\_3 | coexpression\_cluster1\_3 : 1 motif | Show matrix | | |
| cluster\_5 | 1 | cluster1\_3\_motif8\_r::coexpression\_cluster1\_3 | coexpression\_cluster1\_3 : 1 motif | Show matrix | | |
| cluster\_6 | 1 | cluster1\_3\_motif28\_m::coexpression\_cluster1\_3 | coexpression\_cluster1\_3 : 1 motif | Show matrix | | |
| cluster\_7 | 1 | cluster1\_3\_motif11\_r::coexpression\_cluster1\_3 | coexpression\_cluster1\_3 : 1 motif | Show matrix | | |
| cluster\_8 | 1 | cluster1\_3\_motif18\_r::coexpression\_cluster1\_3 | coexpression\_cluster1\_3 : 1 motif | Show matrix | | |
| cluster\_9 | 1 | cluster1\_3\_motif30\_m::coexpression\_cluster1\_3 | coexpression\_cluster1\_3 : 1 motif | Show matrix | | |
| cluster\_10 | 1 | cluster1\_3\_motif29\_m::coexpression\_cluster1\_3 | coexpression\_cluster1\_3 : 1 motif | Show matrix | | |

- Note: by default the table displays the first 50 clusters. Click on the *Show X entries* button to display more (or less) clusters.
- Click on the column names to change the order of the data.
- Use the *Search* window to find your motif (s) of interest.

| **Individual Cluster View**  Individual Cluster View **Show All**  **Hide All**  **cluster\_1**  **cluster\_2**  **cluster\_3**  **cluster\_4**  **cluster\_5**  **cluster\_6**  **cluster\_7**  **cluster\_8**  **cluster\_9**  **cluster\_10**  **Display Logo Trees**   **cluster\_1**    **cluster\_2**    **cluster\_3**    **cluster\_4**    **cluster\_5**    **cluster\_6**    **cluster\_7**    **cluster\_8**    **cluster\_9**    **cluster\_10**  **Display Branch-Motifs** **cluster\_1**   | Branch Motifs | | | | | | --- | --- | --- | --- | --- | | Node | Consensus | Logo | Logo (Reverse) | Collections | Matrix (Transfac format) | IC | Nb sites | | Node 1 | **wwaAAAAArAmAAAww--** |  |  | coexpression\_cluster1\_3::2\_motifs | PSSM | 10.16 | 4023 | | --- | --- | --- | --- | --- | --- | --- | --- | | Node 2 | **wwwAAAAArAcAwwwww-** |  |  | coexpression\_cluster1\_3::2\_motifs | PSSM | 9.55 | 2079 | | Node 3 | **-AAAAAAtAAAAAAAa--** |  |  | coexpression\_cluster1\_3::2\_motifs | PSSM | 12.98 | 809 | | Node 4 | **wwaAAAAArAmAwawww-** |  |  | coexpression\_cluster1\_3::4\_motifs | PSSM | 9.77 | 6102 | | Node 5 | **wwaAAAAArAmAwawwww** |  |  | coexpression\_cluster1\_3::5\_motifs | PSSM | 9.95 | 6780 | | Node 6 | **-AAAAAAtRAAAAAAA--** |  |  | coexpression\_cluster1\_3::3\_motifs | PSSM | 12.59 | 931 | | Node 7 | **wwaAAAAArAmAaaawww** |  |  | coexpression\_cluster1\_3::8\_motifs | PSSM | 9.83 | 7711 | | Node 8 | **---wwwwwTACAACAwww** |  |  | coexpression\_cluster1\_3::2\_motifs | PSSM | 8.65 | 725 | | Node 9 | **wwaAAAAArAmAaaawww** |  |  | coexpression\_cluster1\_3::9\_motifs | PSSM | 9.82 | 7830 | | Node 10 | **wwaAAAAArAmAAaawww** |  |  | coexpression\_cluster1\_3::11\_motifs | PSSM | 9.42 | 8555 |  **cluster\_2**   | Branch Motifs | | | | | | --- | --- | --- | --- | --- | | Node | Consensus | Logo | Logo (Reverse) | Collections | Matrix (Transfac format) | IC | Nb sites | | Node 1 | **---ATATATATrYATATA** |  |  | coexpression\_cluster1\_3::2\_motifs | PSSM | 13.23 | 707 | | --- | --- | --- | --- | --- | --- | --- | --- | | Node 2 | **watAyATGCATryAwaww** |  |  | coexpression\_cluster1\_3::2\_motifs | PSSM | 10.53 | 857 | | Node 3 | **--wwtwTrCATGCAwAww** |  |  | coexpression\_cluster1\_3::2\_motifs | PSSM | 9.14 | 1205 | | Node 4 | **wawAyaTrCATGyAwAww** |  |  | coexpression\_cluster1\_3::4\_motifs | PSSM | 9.40 | 2062 | | Node 5 | **wawAtATryATryAwAta** |  |  | coexpression\_cluster1\_3::6\_motifs | PSSM | 9.67 | 2769 | | Node 6 | **wawAtATryATryAwAta** |  |  | coexpression\_cluster1\_3::7\_motifs | PSSM | 9.74 | 2872 |  **cluster\_3**   | Branch Motifs | | | | | | --- | --- | --- | --- | --- | | Node | Consensus | Logo | Logo (Reverse) | Collections | Matrix (Transfac format) | IC | Nb sites | | Node 1 | **AwwAACAAAww---** |  |  | coexpression\_cluster1\_3::2\_motifs | PSSM | 10.34 | 948 | | --- | --- | --- | --- | --- | --- | --- | --- | | Node 2 | **-wwAAmAAAATTww** |  |  | coexpression\_cluster1\_3::2\_motifs | PSSM | 9.53 | 3037 | | Node 3 | **AwwAACwmAAww--** |  |  | coexpression\_cluster1\_3::3\_motifs | PSSM | 8.38 | 1687 | | Node 4 | **AwwAAmAAAATTww** |  |  | coexpression\_cluster1\_3::5\_motifs | PSSM | 9.42 | 4724 |  **cluster\_4**   | Branch Motifs | | | | | | --- | --- | --- | --- | --- | | Node | Consensus | Logo | Logo (Reverse) | Collections | Matrix (Transfac format) | IC | Nb sites | | Singleton | **aaAAGAGAaa** |  |  | coexpression\_cluster1\_3 : 1 motif | PSSM | 9.68 | 122 | | --- | --- | --- | --- | --- | --- | --- | --- |  **cluster\_5**   | Branch Motifs | | | | | | --- | --- | --- | --- | --- | | Node | Consensus | Logo | Logo (Reverse) | Collections | Matrix (Transfac format) | IC | Nb sites | | Singleton | **wwAAAACAww** |  |  | coexpression\_cluster1\_3 : 1 motif | PSSM | 9.17 | 821 | | --- | --- | --- | --- | --- | --- | --- | --- |  **cluster\_6**   | Branch Motifs | | | | | | --- | --- | --- | --- | --- | | Node | Consensus | Logo | Logo (Reverse) | Collections | Matrix (Transfac format) | IC | Nb sites | | Singleton | **bVYkYSSmtkyCCC** |  |  | coexpression\_cluster1\_3 : 1 motif | PSSM | 7.59 | 66 | | --- | --- | --- | --- | --- | --- | --- | --- |  **cluster\_7**   | Branch Motifs | | | | | | --- | --- | --- | --- | --- | | Node | Consensus | Logo | Logo (Reverse) | Collections | Matrix (Transfac format) | IC | Nb sites | | Singleton | **wwCAAATGww** |  |  | coexpression\_cluster1\_3 : 1 motif | PSSM | 8.75 | 205 | | --- | --- | --- | --- | --- | --- | --- | --- |  **cluster\_8**   | Branch Motifs | | | | | | --- | --- | --- | --- | --- | | Node | Consensus | Logo | Logo (Reverse) | Collections | Matrix (Transfac format) | IC | Nb sites | | Singleton | **wwAACGTCww** |  |  | coexpression\_cluster1\_3 : 1 motif | PSSM | 9.04 | 31 | | --- | --- | --- | --- | --- | --- | --- | --- |  **cluster\_9**   | Branch Motifs | | | | | | --- | --- | --- | --- | --- | | Node | Consensus | Logo | Logo (Reverse) | Collections | Matrix (Transfac format) | IC | Nb sites | | Singleton | **aaaCmttGbaaGttG** |  |  | coexpression\_cluster1\_3 : 1 motif | PSSM | 16.37 | 11 | | --- | --- | --- | --- | --- | --- | --- | --- |  **cluster\_10**   | Branch Motifs | | | | | | --- | --- | --- | --- | --- | | Node | Consensus | Logo | Logo (Reverse) | Collections | Matrix (Transfac format) | IC | Nb sites | | Singleton | **tGKtmGGGRCayCam** |  |  | coexpression\_cluster1\_3 : 1 motif | PSSM | 16.04 | 10 | | --- | --- | --- | --- | --- | --- | --- | --- |   - **Each cluster has a different color.** - **Click on the upper buttons to display separately the information of each cluster.** - **Click on the circles at each tree to display its corresponding merged motifs.**   Definitions  - **Logo tree.**  This results shows the hierarchical tree with its logo alignment of one cluster. The logos are shown in Forward (left logo) and Reverse (right logo) orientation. - **Branch-motifs.**  On the trees are displayed the branch number that is the number in which the motifs were incorporated in the tree. The Branch-Motifs table shows the logo in both orientations and the link to the file in TRANSFAC format with the branch-motif. |
|  | |  |  |  |  |  |  |  |  |  |  |  |  |  |  |  |  |  |  |  |  |  |  |  |  |  |  |  |  |  |  |  |  |  |  |  |  |  |  |  |  |  |  |  |  |  |  |  |  |  |  |  |  |  |  |  |  |  |  |  |  |  |  |  |  |  |  |  |  |  |  |  |  |  |  |  |  |  |  |  |  |  |  |  |  |  |  |  |  |  |  |  |  |  |  |  |  |  |  |  |  |  |  |  |  |  |  |  |  |  |  |  |  |  |  |  |  |  |  |  |  |  |  |  |  |  |  |  |  |  |  |  |  |  |  |  |  |  |  |  |  |  |  |  |  |  |  |  |  |  |  |  |  |  |  |  |  |  |  |  |  |  |  |  |  |  |  |  |  |  |  |  |  |  |  |  |  |  |  |  |  |  |  |  |  |  |  |  |  |  |  |  |  |  |  |  |  |  |  |  |  |  |  |  |  |  |  |  |  |  |  |  |  |  |  |  |  |  |  |  |  |  |  |  |  |  |  |  |  |  |  |  |  |  |  |  |  |  |  |  |  |  |  |  |  |  |  |  |  |  |  |  |  |  |  |  |  |  |  |  |  |  |  |  |  |  |  |  |  |  |  |  |  |  |  |  |  |  |  |  |  |  |  |  |  |  |  |  |  |  |  |  |  |  |  |  |  |  |  |  |  |  |  |  |  |  |  |  |  |  |  |  |  |  |  |  |  |  |  |  |  |  |  |  |  |  |  |  |  |  |  |  |  |  |  |  |  |  |  |  |  |  |  | | --- | --- | --- | --- | --- | --- | --- | --- | --- | --- | --- | --- | --- | --- | --- | --- | --- | --- | --- | --- | --- | --- | --- | --- | --- | --- | --- | --- | --- | --- | --- | --- | --- | --- | --- | --- | --- | --- | --- | --- | --- | --- | --- | --- | --- | --- | --- | --- | --- | --- | --- | --- | --- | --- | --- | --- | --- | --- | --- | --- | --- | --- | --- | --- | --- | --- | --- | --- | --- | --- | --- | --- | --- | --- | --- | --- | --- | --- | --- | --- | --- | --- | --- | --- | --- | --- | --- | --- | --- | --- | --- | --- | --- | --- | --- | --- | --- | --- | --- | --- | --- | --- | --- | --- | --- | --- | --- | --- | --- | --- | --- | --- | --- | --- | --- | --- | --- | --- | --- | --- | --- | --- | --- | --- | --- | --- | --- | --- | --- | --- | --- | --- | --- | --- | --- | --- | --- | --- | --- | --- | --- | --- | --- | --- | --- | --- | --- | --- | --- | --- | --- | --- | --- | --- | --- | --- | --- | --- | --- | --- | --- | --- | --- | --- | --- | --- | --- | --- | --- | --- | --- | --- | --- | --- | --- | --- | --- | --- | --- | --- | --- | --- | --- | --- | --- | --- | --- | --- | --- | --- | --- | --- | --- | --- | --- | --- | --- | --- | --- | --- | --- | --- | --- | --- | --- | --- | --- | --- | --- | --- | --- | --- | --- | --- | --- | --- | --- | --- | --- | --- | --- | --- | --- | --- | --- | --- | --- | --- | --- | --- | --- | --- | --- | --- | --- | --- | --- | --- | --- | --- | --- | --- | --- | --- | --- | --- | --- | --- | --- | --- | --- | --- | --- | --- | --- | --- | --- | --- | --- | --- | --- | --- | --- | --- | --- | --- | --- | --- | --- | --- | --- | --- | --- | --- | --- | --- | --- | --- | --- | --- | --- | --- | --- | --- | --- | --- | --- | --- | --- | --- | --- | --- | --- | --- | --- | --- | --- | --- | --- | --- | --- | --- | --- | --- | --- | --- | --- | --- | --- | --- | --- | --- | --- | --- | --- | --- | --- | --- | --- | --- | --- | --- | --- | --- | --- | --- | --- | --- | --- | --- | --- | --- | --- | --- | --- | --- | --- | --- | --- | --- | --- | --- | | **Individual Motif View**  Individual Motif View  | Motif id | Motif name | Cluster | Collection | Width | IC | Number of sites | Consensus | Consensus (Rev) | Logo | Logo (Rev) | | --- | --- | --- | --- | --- | --- | --- | --- | --- | --- | --- | | coexpression\_cluster1\_3\_m28\_cluster1\_3\_motif28\_m | cluster1\_3\_motif28\_m | cluster\_6 | coexpression\_cluster1\_3 | 14 | 7.59 | 66 | **tmywyscaTtTyCC** | **GGRAAATGSRWRKA** |  |  | | coexpression\_cluster1\_3\_m15\_cluster1\_3\_motif15\_r | cluster1\_3\_motif15\_r | cluster\_1 | coexpression\_cluster1\_3 | 18 | 9.36 | 1342 | **wwwAAAAArAcAtwww++** | **++WWWATGTYTTTTTWWW** |  |  | | coexpression\_cluster1\_3\_m5\_cluster1\_3\_motif5\_r | cluster1\_3\_motif5\_r | cluster\_3 | coexpression\_cluster1\_3 | 14 | 9.26 | 849 | **+waAACAAAww+++** | **+++WWTTTGTTTW+** |  |  | | coexpression\_cluster1\_3\_m21\_cluster1\_3\_motif21\_m | cluster1\_3\_motif21\_m | cluster\_1 | coexpression\_cluster1\_3 | 18 | 14.60 | 335 | **+AAAAAATAAaWAAA+++** | **+++TTTWTTTATTTTTT+** |  |  | | coexpression\_cluster1\_3\_m8\_cluster1\_3\_motif8\_r | cluster1\_3\_motif8\_r | cluster\_5 | coexpression\_cluster1\_3 | 10 | 9.17 | 821 | **wwAAAACAww** | **WWTGTTTTWW** |  |  | | coexpression\_cluster1\_3\_m24\_cluster1\_3\_motif24\_m | cluster1\_3\_motif24\_m | cluster\_1 | coexpression\_cluster1\_3 | 18 | 12.81 | 474 | **+AAAAAAdAAAAAAAa++** | **++TTTTTTTTHTTTTTT+** |  |  | | coexpression\_cluster1\_3\_m19\_cluster1\_3\_motif19\_r | cluster1\_3\_motif19\_r | cluster\_4 | coexpression\_cluster1\_3 | 10 | 9.68 | 122 | **aaAAGAGAaa** | **TTTCTCTTTT** |  |  | | coexpression\_cluster1\_3\_m7\_cluster1\_3\_motif7\_r | cluster1\_3\_motif7\_r | cluster\_2 | coexpression\_cluster1\_3 | 18 | 10.56 | 530 | **WATAYATGCATRTATW++** | **++watAyATGCATrTatw** |  |  | | coexpression\_cluster1\_3\_m6\_cluster1\_3\_motif6\_r | cluster1\_3\_motif6\_r | cluster\_1 | coexpression\_cluster1\_3 | 18 | 9.93 | 737 | **wwaaAAAAgAcAwmaww+** | **+WWTKWTGTCTTTTTTWW** |  |  | | coexpression\_cluster1\_3\_m23\_cluster1\_3\_motif23\_m | cluster1\_3\_motif23\_m | cluster\_1 | coexpression\_cluster1\_3 | 18 | 14.39 | 120 | **TTTTTTTTTTTAATT+++** | **+++AATTAAAAAAAAAAA** |  |  | | coexpression\_cluster1\_3\_m13\_cluster1\_3\_motif13\_r | cluster1\_3\_motif13\_r | cluster\_3 | coexpression\_cluster1\_3 | 14 | 9.45 | 1236 | **+wwAAcAAAATTww** | **WWAATTTTGTTWW+** |  |  | | coexpression\_cluster1\_3\_m17\_cluster1\_3\_motif17\_r | cluster1\_3\_motif17\_r | cluster\_1 | coexpression\_cluster1\_3 | 18 | 10.78 | 2921 | **wwaAAAAArAmAAAww++** | **++WWTTTKTYTTTTTTWW** |  |  | | coexpression\_cluster1\_3\_m12\_cluster1\_3\_motif12\_r | cluster1\_3\_motif12\_r | cluster\_1 | coexpression\_cluster1\_3 | 18 | 9.42 | 1102 | **wwwAAAAArAcAmAww++** | **++WWTKTGTYTTTTTWWW** |  |  | | coexpression\_cluster1\_3\_m25\_cluster1\_3\_motif25\_m | cluster1\_3\_motif25\_m | cluster\_2 | coexpression\_cluster1\_3 | 18 | 15.44 | 182 | **+++ATATRTATAYATAT+** | **+ATATRTATAYATAT+++** |  |  | | coexpression\_cluster1\_3\_m20\_cluster1\_3\_motif20\_m | cluster1\_3\_motif20\_m | cluster\_2 | coexpression\_cluster1\_3 | 18 | 12.68 | 525 | **+++ATATAYATRYATRYA** | **TRYATRyATRTATAT+++** |  |  | | coexpression\_cluster1\_3\_m29\_cluster1\_3\_motif29\_m | cluster1\_3\_motif29\_m | cluster\_10 | coexpression\_cluster1\_3 | 15 | 16.04 | 10 | **TGKTAGGGRCATCAA** | **TTGATGYCCCTAMCA** |  |  | | coexpression\_cluster1\_3\_m27\_cluster1\_3\_motif27\_m | cluster1\_3\_motif27\_m | cluster\_2 | coexpression\_cluster1\_3 | 18 | 11.25 | 103 | **++++++TATGYATGTA++** | **++TACATRCATA++++++** |  |  | | coexpression\_cluster1\_3\_m9\_cluster1\_3\_motif9\_r | cluster1\_3\_motif9\_r | cluster\_3 | coexpression\_cluster1\_3 | 14 | 9.09 | 1801 | **++++wwAAAATTww** | **WWAATTTTWW++++** |  |  | | coexpression\_cluster1\_3\_m16\_cluster1\_3\_motif16\_r | cluster1\_3\_motif16\_r | cluster\_2 | coexpression\_cluster1\_3 | 18 | 10.89 | 327 | **wawAyATGCATrcAwAww** | **WWTWTGYATGCATRTWTW** |  |  | | coexpression\_cluster1\_3\_m2\_cluster1\_3\_motif2\_r | cluster1\_3\_motif2\_r | cluster\_2 | coexpression\_cluster1\_3 | 18 | 9.20 | 655 | **++++waTACATGCAwAww** | **WWTWTGCATGTATW++++** |  |  | | coexpression\_cluster1\_3\_m22\_cluster1\_3\_motif22\_m | cluster1\_3\_motif22\_m | cluster\_3 | coexpression\_cluster1\_3 | 14 | 12.14 | 99 | **ATTAACAAAAT+++** | **+++ATTTTGTTAAT** |  |  | | coexpression\_cluster1\_3\_m26\_cluster1\_3\_motif26\_m | cluster1\_3\_motif26\_m | cluster\_1 | coexpression\_cluster1\_3 | 18 | 11.65 | 122 | **+++TYTTTTTCATTTT++** | **++AAAAtGAAAAarA+++** |  |  | | coexpression\_cluster1\_3\_m3\_cluster1\_3\_motif3\_r | cluster1\_3\_motif3\_r | cluster\_2 | coexpression\_cluster1\_3 | 18 | 9.36 | 550 | **++wwTwTGCATGCAwAww** | **WWTWTGCATGCAWAWW++** |  |  | | coexpression\_cluster1\_3\_m10\_cluster1\_3\_motif10\_r | cluster1\_3\_motif10\_r | cluster\_1 | coexpression\_cluster1\_3 | 18 | 9.71 | 204 | **+++wwAwAgACAAcAdww** | **WWHTGTTGTCTWTWW+++** |  |  | | coexpression\_cluster1\_3\_m30\_cluster1\_3\_motif30\_m | cluster1\_3\_motif30\_m | cluster\_9 | coexpression\_cluster1\_3 | 15 | 16.37 | 11 | **AAACATTGyAAGTTG** | **CAACTTRCAATGTTT** |  |  | | coexpression\_cluster1\_3\_m11\_cluster1\_3\_motif11\_r | cluster1\_3\_motif11\_r | cluster\_7 | coexpression\_cluster1\_3 | 10 | 8.75 | 205 | **wwCAAATGww** | **WWCATTTGWW** |  |  | | coexpression\_cluster1\_3\_m14\_cluster1\_3\_motif14\_r | cluster1\_3\_motif14\_r | cluster\_3 | coexpression\_cluster1\_3 | 14 | 7.24 | 739 | **+WWTTGAGTTWW++** | **++wwAAcTCAAww+** |  |  | | coexpression\_cluster1\_3\_m4\_cluster1\_3\_motif4\_r | cluster1\_3\_motif4\_r | cluster\_1 | coexpression\_cluster1\_3 | 18 | 8.86 | 521 | **+++++wwTTACAACAwww** | **WWWTGTTGTAAWW+++++** |  |  | | coexpression\_cluster1\_3\_m1\_cluster1\_3\_motif1\_r | cluster1\_3\_motif1\_r | cluster\_1 | coexpression\_cluster1\_3 | 18 | 10.86 | 678 | **WWYTKTTKTYTTTTTWWW** | **wwwAAAAArAmAAmArww** |  |  | | coexpression\_cluster1\_3\_m18\_cluster1\_3\_motif18\_r | cluster1\_3\_motif18\_r | cluster\_8 | coexpression\_cluster1\_3 | 10 | 9.04 | 31 | **wwAACGTCww** | **WWGACGTTWW** |  |  |  - Click on the column names to change the order of the data. - Write the name of one cluster, collection or a pattern in the *Search* window. | | **Heatmap View**  Heatmap View Distance table | PDF | |

**Additional Files**

## Exported files

| File | Description |
| --- | --- |
| **Pairwise comparison** | This table shows the pairwise comparison between all the input motifs using different metrics. This is the *compare-matrices* result. |
| **Matrix description** | This table shows information of each input motif. |
| **Cluster-wise Motif Files [zip]** | One TRANSFAC file is generated for each cluster including all the input motifs grouped in the corresponding cluster. |
| **Root motifs** | A file with the root matrix of each cluster. |
| **Motif collections per cluster** | A tab separated file containing the counts of motif of each collection on each cluster. |
| **Clusters** | A tab separated file containing the clusters and their correspondng motifs. |
| **Central motifs IDs** | A tab separated file containing the central motif, that one who is the most similar to all the others motif in the same cluster. |
| **Central motifs (in TRANSFAC format)** | A transfac file containing the central motif, that one who is the most similar to all the others motif within the same cluster. |
| **Input motifs** | The input motifs analyzed with matrix-clustering. |

- **References**
  1. Castro-Mondragon JA et al.  RSAT matrix-clustering: dynamic exploration and redundancy reduction of transcription factor binding motif collections. doi: 10.1093/nar/gkx314; Nucleic Acids Research (2017)
  2. **Last RSAT release:** Nguyen NTT et al. RSAT 2018: regulatory sequence analysis tools 20th anniversary. Nucleic Acids Research (2018).
- **Additional Information**
  - Jacques van Helden course
- **Contact**
  - Jaime Castro-Mondragon - **j.a.c.mondragon@ncmm.uio.no**
  - Morgane Thomas-Chollier - **mthomas@biologie.ens.fr**
  - Jacques van Helden - **Jacques.van-Helden@univ-amu.fr**
