# Supplementary material for: Transcriptome analysis reveals a de novo DNA element that may interact with chromatin-associated proteins in Plasmodium berghei during erythrocytic development
Source: Sci Rep. 2025 May 28;15:18621. doi: 10.1038/s41598-025-03586-4 (PMC12120095; doi:10.1038/s41598-025-03586-4)
Supplement: Supplementary file 2 — Supplementary Information 2. [file 41598_2025_3586_MOESM2_ESM.zip › Supplementary File S2/Clustering/matrix-clustering_SUMMARY_cluster1_6.html]

matrix-clustering coexpression\_cluster1\_6


# RSAT - matrix-clustering result

### Analysis: coexpression\_cluster1\_6 (08/09/2023 15:09)

### Command

```
matrix-clustering  -v 1 -max_matrices 300 -matrix coexpression_cluster1_6 $RSAT/public_html/tmp/www-data/2023/09/08/matrix-clustering_2023-09-08.150848_ydhDNs/matrix-clustering_query_matrices.transfac transfac -hclust_method average -calc sum -title coexpression_cluster1_6 -metric_build_tree Ncor -lth w 5 -lth cor 0.6 -lth Ncor 0.4 -quick -label_in_tree name -return json,heatmap -o $RSAT/public_html/tmp/www-data/2023/09/08/matrix-clustering_2023-09-08.150848_ydhDNs/matrix-clustering
```


## Results Summary

|  |  |  |  |  |  |  |  |  |  |  |
| --- | --- | --- | --- | --- | --- | --- | --- | --- | --- | --- |
| Nb Input motifs | Nb Input collections | Nb Clusters Found | Download   Root motifs | Complete results [zip] | Parameters used  |  |  |  | | --- | --- | --- | | Linkage method | Similarity metric | Thresholds to partition the tree | | | |
| 32 | 1 | 14 | **Download** | Download | |  |  |  | | --- | --- | --- | | average | Ncor | **Ncor** = 0.4 **cor** = 0.6 | |

|  |  |
| --- | --- |
|  | **Logo Forest (dynamic browsing)**  **Logo Forest (rapid overview - low image quality)**  cluster\_1  cluster\_2  cluster\_3  cluster\_4  cluster\_5  cluster\_6  cluster\_7  cluster\_8  cluster\_9  cluster\_10  cluster\_11  cluster\_12  cluster\_13  cluster\_14 |
|  | |  |  |  |  |  |  |  |  |  |  | | --- | --- | --- | --- | --- | --- | --- | --- | --- | --- | | **Clusters Summary**  Clusters Summary Table  | Root Motif | Root Motif (Reverse) | Cluster ID | # Motifs | Motif\_Name::Collection | Number of Motifs by Collection | Root Motif (transfac Format) | | --- | --- | --- | --- | --- | --- | --- | |  | | --- | |  | | cluster\_1 | 7 | cluster1\_6\_motif6\_r::coexpression\_cluster1\_6:: cluster1\_6\_motif2\_r::coexpression\_cluster1\_6:: cluster1\_6\_motif13\_r::coexpression\_cluster1\_6:: cluster1\_6\_motif8\_r::coexpression\_cluster1\_6:: cluster1\_6\_motif25\_m::coexpression\_cluster1\_6:: cluster1\_6\_motif24\_m::coexpression\_cluster1\_6:: cluster1\_6\_motif26\_m::coexpression\_cluster1\_6:: | coexpression\_cluster1\_6::7\_motifs | Show matrix |
| cluster\_2 | 2 | cluster1\_6\_motif10\_r::coexpression\_cluster1\_6:: cluster1\_6\_motif3\_r::coexpression\_cluster1\_6:: | coexpression\_cluster1\_6::2\_motifs | Show matrix | | |
| cluster\_3 | 2 | cluster1\_6\_motif20\_m::coexpression\_cluster1\_6:: cluster1\_6\_motif21\_m::coexpression\_cluster1\_6:: | coexpression\_cluster1\_6::2\_motifs | Show matrix | | |
| cluster\_4 | 2 | cluster1\_6\_motif12\_r::coexpression\_cluster1\_6:: cluster1\_6\_motif18\_r::coexpression\_cluster1\_6:: | coexpression\_cluster1\_6::2\_motifs | Show matrix | | |
| cluster\_5 | 3 | cluster1\_6\_motif14\_r::coexpression\_cluster1\_6:: cluster1\_6\_motif15\_r::coexpression\_cluster1\_6:: cluster1\_6\_motif31\_m::coexpression\_cluster1\_6:: | coexpression\_cluster1\_6::3\_motifs | Show matrix | | |
| cluster\_6 | 5 | cluster1\_6\_motif22\_m::coexpression\_cluster1\_6:: cluster1\_6\_motif1\_r::coexpression\_cluster1\_6:: cluster1\_6\_motif7\_r::coexpression\_cluster1\_6:: cluster1\_6\_motif17\_r::coexpression\_cluster1\_6:: cluster1\_6\_motif27\_m::coexpression\_cluster1\_6:: | coexpression\_cluster1\_6::5\_motifs | Show matrix | | |
| cluster\_7 | 2 | cluster1\_6\_motif19\_r::coexpression\_cluster1\_6:: cluster1\_6\_motif4\_r::coexpression\_cluster1\_6:: | coexpression\_cluster1\_6::2\_motifs | Show matrix | | |
| cluster\_8 | 1 | cluster1\_6\_motif5\_r::coexpression\_cluster1\_6 | coexpression\_cluster1\_6 : 1 motif | Show matrix | | |
| cluster\_9 | 2 | cluster1\_6\_motif23\_m::coexpression\_cluster1\_6:: cluster1\_6\_motif28\_m::coexpression\_cluster1\_6:: | coexpression\_cluster1\_6::2\_motifs | Show matrix | | |
| cluster\_10 | 1 | cluster1\_6\_motif30\_m::coexpression\_cluster1\_6 | coexpression\_cluster1\_6 : 1 motif | Show matrix | | |
| cluster\_11 | 2 | cluster1\_6\_motif11\_r::coexpression\_cluster1\_6:: cluster1\_6\_motif16\_r::coexpression\_cluster1\_6:: | coexpression\_cluster1\_6::2\_motifs | Show matrix | | |
| cluster\_12 | 1 | cluster1\_6\_motif9\_r::coexpression\_cluster1\_6 | coexpression\_cluster1\_6 : 1 motif | Show matrix | | |
| cluster\_13 | 1 | cluster1\_6\_motif29\_m::coexpression\_cluster1\_6 | coexpression\_cluster1\_6 : 1 motif | Show matrix | | |
| cluster\_14 | 1 | cluster1\_6\_motif32\_m::coexpression\_cluster1\_6 | coexpression\_cluster1\_6 : 1 motif | Show matrix | | |

- Note: by default the table displays the first 50 clusters. Click on the *Show X entries* button to display more (or less) clusters.
- Click on the column names to change the order of the data.
- Use the *Search* window to find your motif (s) of interest.

| **Individual Cluster View**  Individual Cluster View **Show All**  **Hide All**  **cluster\_1**  **cluster\_2**  **cluster\_3**  **cluster\_4**  **cluster\_5**  **cluster\_6**  **cluster\_7**  **cluster\_8**  **cluster\_9**  **cluster\_10**  **cluster\_11**  **cluster\_12**  **cluster\_13**  **cluster\_14**  **Display Logo Trees**   **cluster\_1**    **cluster\_2**    **cluster\_3**    **cluster\_4**    **cluster\_5**    **cluster\_6**    **cluster\_7**    **cluster\_8**    **cluster\_9**    **cluster\_10**    **cluster\_11**    **cluster\_12**    **cluster\_13**    **cluster\_14**  **Display Branch-Motifs** **cluster\_1**   | Branch Motifs | | | | | | --- | --- | --- | --- | --- | | Node | Consensus | Logo | Logo (Reverse) | Collections | Matrix (Transfac format) | IC | Nb sites | | Node 1 | **-wwAAAAgACAwww--** |  |  | coexpression\_cluster1\_6::2\_motifs | PSSM | 8.46 | 802 | | --- | --- | --- | --- | --- | --- | --- | --- | | Node 2 | **-wwAAAAgACAwwww-** |  |  | coexpression\_cluster1\_6::3\_motifs | PSSM | 8.60 | 1379 | | Node 3 | **AAAAAAAAAAAAwAA-** |  |  | coexpression\_cluster1\_6::2\_motifs | PSSM | 12.87 | 416 | | Node 4 | **-wwAAAAgACAawww-** |  |  | coexpression\_cluster1\_6::4\_motifs | PSSM | 8.27 | 1663 | | Node 5 | **AAAAAAAaAAAAwAAA** |  |  | coexpression\_cluster1\_6::3\_motifs | PSSM | 13.43 | 524 | | Node 6 | **AwaAAAArAmAawwaA** |  |  | coexpression\_cluster1\_6::7\_motifs | PSSM | 9.92 | 2187 |  **cluster\_2**   | Branch Motifs | | | | | | --- | --- | --- | --- | --- | | Node | Consensus | Logo | Logo (Reverse) | Collections | Matrix (Transfac format) | IC | Nb sites | | Node 1 | **wwwwAAAAAAATTTTTTTwwww** |  |  | coexpression\_cluster1\_6::2\_motifs | PSSM | 12.62 | 1479 | | --- | --- | --- | --- | --- | --- | --- | --- |  **cluster\_3**   | Branch Motifs | | | | | | --- | --- | --- | --- | --- | | Node | Consensus | Logo | Logo (Reverse) | Collections | Matrix (Transfac format) | IC | Nb sites | | Node 1 | **TATATRyATATaTAT** |  |  | coexpression\_cluster1\_6::2\_motifs | PSSM | 12.64 | 349 | | --- | --- | --- | --- | --- | --- | --- | --- |  **cluster\_4**   | Branch Motifs | | | | | | --- | --- | --- | --- | --- | | Node | Consensus | Logo | Logo (Reverse) | Collections | Matrix (Transfac format) | IC | Nb sites | | Node 1 | **wwyAACCAwa** |  |  | coexpression\_cluster1\_6::2\_motifs | PSSM | 8.64 | 109 | | --- | --- | --- | --- | --- | --- | --- | --- |  **cluster\_5**   | Branch Motifs | | | | | | --- | --- | --- | --- | --- | | Node | Consensus | Logo | Logo (Reverse) | Collections | Matrix (Transfac format) | IC | Nb sites | | Node 1 | **whTAGCTAwwT** |  |  | coexpression\_cluster1\_6::2\_motifs | PSSM | 9.64 | 202 | | --- | --- | --- | --- | --- | --- | --- | --- | | Node 2 | **waTAGCyAaww** |  |  | coexpression\_cluster1\_6::3\_motifs | PSSM | 7.31 | 513 |  **cluster\_6**   | Branch Motifs | | | | | | --- | --- | --- | --- | --- | | Node | Consensus | Logo | Logo (Reverse) | Collections | Matrix (Transfac format) | IC | Nb sites | | Node 1 | **wwTTATGAAcAAaww---** |  |  | coexpression\_cluster1\_6::2\_motifs | PSSM | 9.19 | 717 | | --- | --- | --- | --- | --- | --- | --- | --- | | Node 2 | **wwTTATGAAcAAawwAkr** |  |  | coexpression\_cluster1\_6::3\_motifs | PSSM | 11.78 | 776 | | Node 3 | **---wwTkAACAaw-----** |  |  | coexpression\_cluster1\_6::2\_motifs | PSSM | 8.08 | 351 | | Node 4 | **wwTTATkAACAAwwwAkr** |  |  | coexpression\_cluster1\_6::5\_motifs | PSSM | 11.60 | 1127 |  **cluster\_7**   | Branch Motifs | | | | | | --- | --- | --- | --- | --- | | Node | Consensus | Logo | Logo (Reverse) | Collections | Matrix (Transfac format) | IC | Nb sites | | Node 1 | **wwTGTTGTAAww** |  |  | coexpression\_cluster1\_6::2\_motifs | PSSM | 8.17 | 335 | | --- | --- | --- | --- | --- | --- | --- | --- |  **cluster\_8**   | Branch Motifs | | | | | | --- | --- | --- | --- | --- | | Node | Consensus | Logo | Logo (Reverse) | Collections | Matrix (Transfac format) | IC | Nb sites | | Singleton | **wwACACACAaww** |  |  | coexpression\_cluster1\_6 : 1 motif | PSSM | 8.37 | 361 | | --- | --- | --- | --- | --- | --- | --- | --- |  **cluster\_9**   | Branch Motifs | | | | | | --- | --- | --- | --- | --- | | Node | Consensus | Logo | Logo (Reverse) | Collections | Matrix (Transfac format) | IC | Nb sites | | Node 1 | **yystrwTAGCTAkyTrwytatg** |  |  | coexpression\_cluster1\_6::2\_motifs | PSSM | 9.45 | 109 | | --- | --- | --- | --- | --- | --- | --- | --- |  **cluster\_10**   | Branch Motifs | | | | | | --- | --- | --- | --- | --- | | Node | Consensus | Logo | Logo (Reverse) | Collections | Matrix (Transfac format) | IC | Nb sites | | Singleton | **aaaaaSGrrra** |  |  | coexpression\_cluster1\_6 : 1 motif | PSSM | 10.37 | 79 | | --- | --- | --- | --- | --- | --- | --- | --- |  **cluster\_11**   | Branch Motifs | | | | | | --- | --- | --- | --- | --- | | Node | Consensus | Logo | Logo (Reverse) | Collections | Matrix (Transfac format) | IC | Nb sites | | Node 1 | **wwAATTCAGwwww** |  |  | coexpression\_cluster1\_6::2\_motifs | PSSM | 8.06 | 469 | | --- | --- | --- | --- | --- | --- | --- | --- |  **cluster\_12**   | Branch Motifs | | | | | | --- | --- | --- | --- | --- | | Node | Consensus | Logo | Logo (Reverse) | Collections | Matrix (Transfac format) | IC | Nb sites | | Singleton | **wwGAGTTAww** |  |  | coexpression\_cluster1\_6 : 1 motif | PSSM | 9.09 | 58 | | --- | --- | --- | --- | --- | --- | --- | --- |  **cluster\_13**   | Branch Motifs | | | | | | --- | --- | --- | --- | --- | | Node | Consensus | Logo | Logo (Reverse) | Collections | Matrix (Transfac format) | IC | Nb sites | | Singleton | **mbSssssyGCaCaCrCvSSSSS** |  |  | coexpression\_cluster1\_6 : 1 motif | PSSM | 11.24 | 51 | | --- | --- | --- | --- | --- | --- | --- | --- |  **cluster\_14**   | Branch Motifs | | | | | | --- | --- | --- | --- | --- | | Node | Consensus | Logo | Logo (Reverse) | Collections | Matrix (Transfac format) | IC | Nb sites | | Singleton | **CVCCVC** |  |  | coexpression\_cluster1\_6 : 1 motif | PSSM | 6.35 | 49 | | --- | --- | --- | --- | --- | --- | --- | --- |   - **Each cluster has a different color.** - **Click on the upper buttons to display separately the information of each cluster.** - **Click on the circles at each tree to display its corresponding merged motifs.**   Definitions  - **Logo tree.**  This results shows the hierarchical tree with its logo alignment of one cluster. The logos are shown in Forward (left logo) and Reverse (right logo) orientation. - **Branch-motifs.**  On the trees are displayed the branch number that is the number in which the motifs were incorporated in the tree. The Branch-Motifs table shows the logo in both orientations and the link to the file in TRANSFAC format with the branch-motif. |
|  | |  |  |  |  |  |  |  |  |  |  |  |  |  |  |  |  |  |  |  |  |  |  |  |  |  |  |  |  |  |  |  |  |  |  |  |  |  |  |  |  |  |  |  |  |  |  |  |  |  |  |  |  |  |  |  |  |  |  |  |  |  |  |  |  |  |  |  |  |  |  |  |  |  |  |  |  |  |  |  |  |  |  |  |  |  |  |  |  |  |  |  |  |  |  |  |  |  |  |  |  |  |  |  |  |  |  |  |  |  |  |  |  |  |  |  |  |  |  |  |  |  |  |  |  |  |  |  |  |  |  |  |  |  |  |  |  |  |  |  |  |  |  |  |  |  |  |  |  |  |  |  |  |  |  |  |  |  |  |  |  |  |  |  |  |  |  |  |  |  |  |  |  |  |  |  |  |  |  |  |  |  |  |  |  |  |  |  |  |  |  |  |  |  |  |  |  |  |  |  |  |  |  |  |  |  |  |  |  |  |  |  |  |  |  |  |  |  |  |  |  |  |  |  |  |  |  |  |  |  |  |  |  |  |  |  |  |  |  |  |  |  |  |  |  |  |  |  |  |  |  |  |  |  |  |  |  |  |  |  |  |  |  |  |  |  |  |  |  |  |  |  |  |  |  |  |  |  |  |  |  |  |  |  |  |  |  |  |  |  |  |  |  |  |  |  |  |  |  |  |  |  |  |  |  |  |  |  |  |  |  |  |  |  |  |  |  |  |  |  |  |  |  |  |  |  |  |  |  |  |  |  |  |  |  |  |  |  |  |  |  |  |  |  |  |  |  |  |  |  |  |  |  |  |  |  |  |  |  |  |  |  |  |  |  | | --- | --- | --- | --- | --- | --- | --- | --- | --- | --- | --- | --- | --- | --- | --- | --- | --- | --- | --- | --- | --- | --- | --- | --- | --- | --- | --- | --- | --- | --- | --- | --- | --- | --- | --- | --- | --- | --- | --- | --- | --- | --- | --- | --- | --- | --- | --- | --- | --- | --- | --- | --- | --- | --- | --- | --- | --- | --- | --- | --- | --- | --- | --- | --- | --- | --- | --- | --- | --- | --- | --- | --- | --- | --- | --- | --- | --- | --- | --- | --- | --- | --- | --- | --- | --- | --- | --- | --- | --- | --- | --- | --- | --- | --- | --- | --- | --- | --- | --- | --- | --- | --- | --- | --- | --- | --- | --- | --- | --- | --- | --- | --- | --- | --- | --- | --- | --- | --- | --- | --- | --- | --- | --- | --- | --- | --- | --- | --- | --- | --- | --- | --- | --- | --- | --- | --- | --- | --- | --- | --- | --- | --- | --- | --- | --- | --- | --- | --- | --- | --- | --- | --- | --- | --- | --- | --- | --- | --- | --- | --- | --- | --- | --- | --- | --- | --- | --- | --- | --- | --- | --- | --- | --- | --- | --- | --- | --- | --- | --- | --- | --- | --- | --- | --- | --- | --- | --- | --- | --- | --- | --- | --- | --- | --- | --- | --- | --- | --- | --- | --- | --- | --- | --- | --- | --- | --- | --- | --- | --- | --- | --- | --- | --- | --- | --- | --- | --- | --- | --- | --- | --- | --- | --- | --- | --- | --- | --- | --- | --- | --- | --- | --- | --- | --- | --- | --- | --- | --- | --- | --- | --- | --- | --- | --- | --- | --- | --- | --- | --- | --- | --- | --- | --- | --- | --- | --- | --- | --- | --- | --- | --- | --- | --- | --- | --- | --- | --- | --- | --- | --- | --- | --- | --- | --- | --- | --- | --- | --- | --- | --- | --- | --- | --- | --- | --- | --- | --- | --- | --- | --- | --- | --- | --- | --- | --- | --- | --- | --- | --- | --- | --- | --- | --- | --- | --- | --- | --- | --- | --- | --- | --- | --- | --- | --- | --- | --- | --- | --- | --- | --- | --- | --- | --- | --- | --- | --- | --- | --- | --- | --- | --- | --- | --- | --- | --- | --- | --- | --- | --- | --- | --- | --- | --- | --- | --- | --- | --- | --- | --- | --- | --- | --- | --- | --- | --- | --- | --- | --- | --- | --- | --- | --- | --- | --- | | **Individual Motif View**  Individual Motif View  | Motif id | Motif name | Cluster | Collection | Width | IC | Number of sites | Consensus | Consensus (Rev) | Logo | Logo (Rev) | | --- | --- | --- | --- | --- | --- | --- | --- | --- | --- | --- | | coexpression\_cluster1\_6\_m22\_cluster1\_6\_motif22\_m | cluster1\_6\_motif22\_m | cluster\_6 | coexpression\_cluster1\_6 | 18 | 12.48 | 59 | **+++YMTKRMWTGTTCATA** | **TATGAACAwkymAkr+++** |  |  | | coexpression\_cluster1\_6\_m31\_cluster1\_6\_motif31\_m | cluster1\_6\_motif31\_m | cluster\_5 | coexpression\_cluster1\_6 | 11 | 10.50 | 52 | **rcTAGCyAWTT** | **AAWTRGCTAGY** |  |  | | coexpression\_cluster1\_6\_m3\_cluster1\_6\_motif3\_r | cluster1\_6\_motif3\_r | cluster\_2 | coexpression\_cluster1\_6 | 22 | 12.78 | 570 | **wwwmAAAAAAATTTTTTTkwww** | **WWWMAAAAAAATTTTTTTKWWW** |  |  | | coexpression\_cluster1\_6\_m27\_cluster1\_6\_motif27\_m | cluster1\_6\_motif27\_m | cluster\_6 | coexpression\_cluster1\_6 | 18 | 7.75 | 134 | **+++++TGAACAA++++++** | **++++++TTGTTCA+++++** |  |  | | coexpression\_cluster1\_6\_m24\_cluster1\_6\_motif24\_m | cluster1\_6\_motif24\_m | cluster\_1 | coexpression\_cluster1\_6 | 16 | 15.78 | 107 | **+AAAAAAAAAAATAA+** | **+TTATTTTTTTTTTT+** |  |  | | coexpression\_cluster1\_6\_m18\_cluster1\_6\_motif18\_r | cluster1\_6\_motif18\_r | cluster\_4 | coexpression\_cluster1\_6 | 10 | 9.24 | 73 | **wwTAACCAww** | **WWTGGTTAWW** |  |  | | coexpression\_cluster1\_6\_m32\_cluster1\_6\_motif32\_m | cluster1\_6\_motif32\_m | cluster\_14 | coexpression\_cluster1\_6 | 6 | 6.35 | 49 | **CmCCmC** | **GKGGKG** |  |  | | coexpression\_cluster1\_6\_m12\_cluster1\_6\_motif12\_r | cluster1\_6\_motif12\_r | cluster\_4 | coexpression\_cluster1\_6 | 10 | 9.50 | 36 | **waCAACCAwa** | **TWTGGTTGTW** |  |  | | coexpression\_cluster1\_6\_m19\_cluster1\_6\_motif19\_r | cluster1\_6\_motif19\_r | cluster\_7 | coexpression\_cluster1\_6 | 12 | 8.86 | 52 | **+wwGTTGCAww+** | **+WWTGCAACWW+** |  |  | | coexpression\_cluster1\_6\_m11\_cluster1\_6\_motif11\_r | cluster1\_6\_motif11\_r | cluster\_11 | coexpression\_cluster1\_6 | 13 | 9.70 | 59 | **+++rtTCAGAAww** | **WWTTCTGAAY+++** |  |  | | coexpression\_cluster1\_6\_m9\_cluster1\_6\_motif9\_r | cluster1\_6\_motif9\_r | cluster\_12 | coexpression\_cluster1\_6 | 10 | 9.09 | 58 | **wwGAGTTAww** | **WWTAACTCWW** |  |  | | coexpression\_cluster1\_6\_m10\_cluster1\_6\_motif10\_r | cluster1\_6\_motif10\_r | cluster\_2 | coexpression\_cluster1\_6 | 22 | 12.48 | 909 | **WWWWAAAAAAATTTTTTTWWW+** | **+wwwAAAAAAATTTTTTTwwww** |  |  | | coexpression\_cluster1\_6\_m23\_cluster1\_6\_motif23\_m | cluster1\_6\_motif23\_m | cluster\_9 | coexpression\_cluster1\_6 | 22 | 11.68 | 51 | **+++++wTrGCTAkYTwwkha++** | **++TDMWWARMTAGCYAW+++++** |  |  | | coexpression\_cluster1\_6\_m2\_cluster1\_6\_motif2\_r | cluster1\_6\_motif2\_r | cluster\_1 | coexpression\_cluster1\_6 | 16 | 8.89 | 577 | **+WWWTTGTCTTTTWW+** | **+wwaAAAgACAAwww+** |  |  | | coexpression\_cluster1\_6\_m28\_cluster1\_6\_motif28\_m | cluster1\_6\_motif28\_m | cluster\_9 | coexpression\_cluster1\_6 | 22 | 9.85 | 58 | **yystrwyAGCTAGCTvrckmtg** | **CAKMGYBAGCTAGCTRWYASRR** |  |  | | coexpression\_cluster1\_6\_m17\_cluster1\_6\_motif17\_r | cluster1\_6\_motif17\_r | cluster\_6 | coexpression\_cluster1\_6 | 18 | 9.22 | 217 | **+++WWTTAACAWW+++++** | **+++++wwTGTTAAww+++** |  |  | | coexpression\_cluster1\_6\_m16\_cluster1\_6\_motif16\_r | cluster1\_6\_motif16\_r | cluster\_11 | coexpression\_cluster1\_6 | 13 | 7.16 | 410 | **wwAATTCAgww++** | **++WWCTGAATTWW** |  |  | | coexpression\_cluster1\_6\_m13\_cluster1\_6\_motif13\_r | cluster1\_6\_motif13\_r | cluster\_1 | coexpression\_cluster1\_6 | 16 | 8.57 | 631 | **+wwAAAAgACAwww++** | **++WWWTGTCTTTTWW+** |  |  | | coexpression\_cluster1\_6\_m6\_cluster1\_6\_motif6\_r | cluster1\_6\_motif6\_r | cluster\_1 | coexpression\_cluster1\_6 | 16 | 8.90 | 284 | **+WWWTTGTCTATTWW+** | **+wwAATAgACAAwww+** |  |  | | coexpression\_cluster1\_6\_m4\_cluster1\_6\_motif4\_r | cluster1\_6\_motif4\_r | cluster\_7 | coexpression\_cluster1\_6 | 12 | 8.77 | 283 | **wwTGTTGTAAww** | **WWTTACAACAWW** |  |  | | coexpression\_cluster1\_6\_m25\_cluster1\_6\_motif25\_m | cluster1\_6\_motif25\_m | cluster\_1 | coexpression\_cluster1\_6 | 16 | 13.97 | 108 | **+++TTTATTTTATTTT** | **AAAATAAAATAAA+++** |  |  | | coexpression\_cluster1\_6\_m8\_cluster1\_6\_motif8\_r | cluster1\_6\_motif8\_r | cluster\_1 | coexpression\_cluster1\_6 | 16 | 8.95 | 171 | **+wwwAAAgACAcaw++** | **++WTGTGTCTTTWWW+** |  |  | | coexpression\_cluster1\_6\_m26\_cluster1\_6\_motif26\_m | cluster1\_6\_motif26\_m | cluster\_1 | coexpression\_cluster1\_6 | 16 | 12.64 | 309 | **AAAAAaAAAaAAAAA+** | **+TTTTTTTTTTTTTTT** |  |  | | coexpression\_cluster1\_6\_m21\_cluster1\_6\_motif21\_m | cluster1\_6\_motif21\_m | cluster\_3 | coexpression\_cluster1\_6 | 15 | 13.90 | 99 | **TrTATATATATAyA+** | **+TRTATATATATAYA** |  |  | | coexpression\_cluster1\_6\_m1\_cluster1\_6\_motif1\_r | cluster1\_6\_motif1\_r | cluster\_6 | coexpression\_cluster1\_6 | 18 | 9.56 | 566 | **WWTTATGAAMAAAWW+++** | **+++wwtTTkTTcATAAww** |  |  | | coexpression\_cluster1\_6\_m14\_cluster1\_6\_motif14\_r | cluster1\_6\_motif14\_r | cluster\_5 | coexpression\_cluster1\_6 | 11 | 7.27 | 311 | **WWTTGGCTATW** | **waTAGCCAAww** |  |  | | coexpression\_cluster1\_6\_m15\_cluster1\_6\_motif15\_r | cluster1\_6\_motif15\_r | cluster\_5 | coexpression\_cluster1\_6 | 11 | 9.27 | 150 | **WWTAGCTAWW+** | **+wwTAGCTAww** |  |  | | coexpression\_cluster1\_6\_m20\_cluster1\_6\_motif20\_m | cluster1\_6\_motif20\_m | cluster\_3 | coexpression\_cluster1\_6 | 15 | 12.52 | 250 | **TATATryATATrTrT** | **AYAYATATRYATATA** |  |  | | coexpression\_cluster1\_6\_m5\_cluster1\_6\_motif5\_r | cluster1\_6\_motif5\_r | cluster\_8 | coexpression\_cluster1\_6 | 12 | 8.37 | 361 | **wwACACACAaww** | **WWTTGTGTGTWW** |  |  | | coexpression\_cluster1\_6\_m7\_cluster1\_6\_motif7\_r | cluster1\_6\_motif7\_r | cluster\_6 | coexpression\_cluster1\_6 | 18 | 9.12 | 151 | **++wwmTGAACAAwww+++** | **+++WWWTTGTTCAKWW++** |  |  | | coexpression\_cluster1\_6\_m29\_cluster1\_6\_motif29\_m | cluster1\_6\_motif29\_m | cluster\_13 | coexpression\_cluster1\_6 | 22 | 11.24 | 51 | **wtsawttTGCACACACrymrrg** | **CYYKRYGTGTGTGCAAAWTSAW** |  |  | | coexpression\_cluster1\_6\_m30\_cluster1\_6\_motif30\_m | cluster1\_6\_motif30\_m | cluster\_10 | coexpression\_cluster1\_6 | 11 | 10.37 | 79 | **AAAAACGAAaA** | **TTTTCGTTTTT** |  |  |  - Click on the column names to change the order of the data. - Write the name of one cluster, collection or a pattern in the *Search* window. | | **Heatmap View**  Heatmap View Distance table | PDF | |

**Additional Files**

## Exported files

| File | Description |
| --- | --- |
| **Pairwise comparison** | This table shows the pairwise comparison between all the input motifs using different metrics. This is the *compare-matrices* result. |
| **Matrix description** | This table shows information of each input motif. |
| **Cluster-wise Motif Files [zip]** | One TRANSFAC file is generated for each cluster including all the input motifs grouped in the corresponding cluster. |
| **Root motifs** | A file with the root matrix of each cluster. |
| **Motif collections per cluster** | A tab separated file containing the counts of motif of each collection on each cluster. |
| **Clusters** | A tab separated file containing the clusters and their correspondng motifs. |
| **Central motifs IDs** | A tab separated file containing the central motif, that one who is the most similar to all the others motif in the same cluster. |
| **Central motifs (in TRANSFAC format)** | A transfac file containing the central motif, that one who is the most similar to all the others motif within the same cluster. |
| **Input motifs** | The input motifs analyzed with matrix-clustering. |

- **References**
  1. Castro-Mondragon JA et al.  RSAT matrix-clustering: dynamic exploration and redundancy reduction of transcription factor binding motif collections. doi: 10.1093/nar/gkx314; Nucleic Acids Research (2017)
  2. **Last RSAT release:** Nguyen NTT et al. RSAT 2018: regulatory sequence analysis tools 20th anniversary. Nucleic Acids Research (2018).
- **Additional Information**
  - Jacques van Helden course
- **Contact**
  - Jaime Castro-Mondragon - **j.a.c.mondragon@ncmm.uio.no**
  - Morgane Thomas-Chollier - **mthomas@biologie.ens.fr**
  - Jacques van Helden - **Jacques.van-Helden@univ-amu.fr**
