# Supplementary material for: Transcriptome analysis reveals a de novo DNA element that may interact with chromatin-associated proteins in Plasmodium berghei during erythrocytic development
Source: Sci Rep. 2025 May 28;15:18621. doi: 10.1038/s41598-025-03586-4 (PMC12120095; doi:10.1038/s41598-025-03586-4)
Supplement: Supplementary file 2 — Supplementary Information 2. [file 41598_2025_3586_MOESM2_ESM.zip › Supplementary File S2/Clustering/matrix-clustering_SUMMARY_cluster2_10.html]

matrix-clustering coexpression\_cluster2\_10


# RSAT - matrix-clustering result

### Analysis: coexpression\_cluster2\_10 (08/09/2023 15:39)

### Command

```
matrix-clustering  -v 1 -max_matrices 300 -matrix coexpression_cluster2_10 $RSAT/public_html/tmp/www-data/2023/09/08/matrix-clustering_2023-09-08.153926_Sv8JQG/matrix-clustering_query_matrices.transfac transfac -hclust_method average -calc sum -title coexpression_cluster2_10 -metric_build_tree Ncor -lth w 5 -lth cor 0.6 -lth Ncor 0.4 -quick -label_in_tree name -return json,heatmap -o $RSAT/public_html/tmp/www-data/2023/09/08/matrix-clustering_2023-09-08.153926_Sv8JQG/matrix-clustering
```


## Results Summary

|  |  |  |  |  |  |  |  |  |  |  |
| --- | --- | --- | --- | --- | --- | --- | --- | --- | --- | --- |
| Nb Input motifs | Nb Input collections | Nb Clusters Found | Download   Root motifs | Complete results [zip] | Parameters used  |  |  |  | | --- | --- | --- | | Linkage method | Similarity metric | Thresholds to partition the tree | | | |
| 16 | 1 | 6 | **Download** | Download | |  |  |  | | --- | --- | --- | | average | Ncor | **Ncor** = 0.4 **cor** = 0.6 | |

|  |  |
| --- | --- |
|  | **Logo Forest (dynamic browsing)**  **Logo Forest (rapid overview - low image quality)**  cluster\_1  cluster\_2  cluster\_3  cluster\_4  cluster\_5  cluster\_6 |
|  | |  |  |  |  |  |  |  |  |  |  | | --- | --- | --- | --- | --- | --- | --- | --- | --- | --- | | **Clusters Summary**  Clusters Summary Table  | Root Motif | Root Motif (Reverse) | Cluster ID | # Motifs | Motif\_Name::Collection | Number of Motifs by Collection | Root Motif (transfac Format) | | --- | --- | --- | --- | --- | --- | --- | |  | | --- | |  | | cluster\_1 | 5 | cluster2\_10\_motif10\_m::coexpression\_cluster2\_10:: cluster2\_10\_motif9\_m::coexpression\_cluster2\_10:: cluster2\_10\_motif15\_m::coexpression\_cluster2\_10:: cluster2\_10\_motif4\_r::coexpression\_cluster2\_10:: cluster2\_10\_motif6\_r::coexpression\_cluster2\_10:: | coexpression\_cluster2\_10::5\_motifs | Show matrix |
| cluster\_2 | 3 | cluster2\_10\_motif2\_r::coexpression\_cluster2\_10:: cluster2\_10\_motif1\_r::coexpression\_cluster2\_10:: cluster2\_10\_motif3\_r::coexpression\_cluster2\_10:: | coexpression\_cluster2\_10::3\_motifs | Show matrix | | |
| cluster\_3 | 4 | cluster2\_10\_motif16\_m::coexpression\_cluster2\_10:: cluster2\_10\_motif14\_m::coexpression\_cluster2\_10:: cluster2\_10\_motif12\_m::coexpression\_cluster2\_10:: cluster2\_10\_motif8\_m::coexpression\_cluster2\_10:: | coexpression\_cluster2\_10::4\_motifs | Show matrix | | |
| cluster\_4 | 2 | cluster2\_10\_motif13\_m::coexpression\_cluster2\_10:: cluster2\_10\_motif5\_r::coexpression\_cluster2\_10:: | coexpression\_cluster2\_10::2\_motifs | Show matrix | | |
| cluster\_5 | 1 | cluster2\_10\_motif7\_r::coexpression\_cluster2\_10 | coexpression\_cluster2\_10 : 1 motif | Show matrix | | |
| cluster\_6 | 1 | cluster2\_10\_motif11\_m::coexpression\_cluster2\_10 | coexpression\_cluster2\_10 : 1 motif | Show matrix | | |

- Note: by default the table displays the first 50 clusters. Click on the *Show X entries* button to display more (or less) clusters.
- Click on the column names to change the order of the data.
- Use the *Search* window to find your motif (s) of interest.

| **Individual Cluster View**  Individual Cluster View **Show All**  **Hide All**  **cluster\_1**  **cluster\_2**  **cluster\_3**  **cluster\_4**  **cluster\_5**  **cluster\_6**  **Display Logo Trees**   **cluster\_1**    **cluster\_2**    **cluster\_3**    **cluster\_4**    **cluster\_5**    **cluster\_6**  **Display Branch-Motifs** **cluster\_1**   | Branch Motifs | | | | | | --- | --- | --- | --- | --- | | Node | Consensus | Logo | Logo (Reverse) | Collections | Matrix (Transfac format) | IC | Nb sites | | Node 1 | **TAtATryATAYATAy-** |  |  | coexpression\_cluster2\_10::2\_motifs | PSSM | 13.60 | 394 | | --- | --- | --- | --- | --- | --- | --- | --- | | Node 2 | **--wwATTAyATAww--** |  |  | coexpression\_cluster2\_10::2\_motifs | PSSM | 8.47 | 1443 | | Node 3 | **--wwATTAyATAwwCA** |  |  | coexpression\_cluster2\_10::3\_motifs | PSSM | 11.17 | 1491 | | Node 4 | **TAwwAtTAyATAtayA** |  |  | coexpression\_cluster2\_10::5\_motifs | PSSM | 11.23 | 1884 |  **cluster\_2**   | Branch Motifs | | | | | | --- | --- | --- | --- | --- | | Node | Consensus | Logo | Logo (Reverse) | Collections | Matrix (Transfac format) | IC | Nb sites | | Node 1 | **wwwwwwTATTATATAATATATwww** |  |  | coexpression\_cluster2\_10::2\_motifs | PSSM | 12.72 | 3184 | | --- | --- | --- | --- | --- | --- | --- | --- | | Node 2 | **wwwwwwwwwTATATAATATATwww** |  |  | coexpression\_cluster2\_10::3\_motifs | PSSM | 12.11 | 5521 |  **cluster\_3**   | Branch Motifs | | | | | | --- | --- | --- | --- | --- | | Node | Consensus | Logo | Logo (Reverse) | Collections | Matrix (Transfac format) | IC | Nb sites | | Node 1 | **TTTTwYywtTTTTTT-** |  |  | coexpression\_cluster2\_10::2\_motifs | PSSM | 11.80 | 397 | | --- | --- | --- | --- | --- | --- | --- | --- | | Node 2 | **TTTTwTTwTTTTTTT-** |  |  | coexpression\_cluster2\_10::3\_motifs | PSSM | 11.25 | 480 | | Node 3 | **TTTTwTywTTTTTTTG** |  |  | coexpression\_cluster2\_10::4\_motifs | PSSM | 12.25 | 497 |  **cluster\_4**   | Branch Motifs | | | | | | --- | --- | --- | --- | --- | | Node | Consensus | Logo | Logo (Reverse) | Collections | Matrix (Transfac format) | IC | Nb sites | | Node 1 | **wmCTTAAcAAww** |  |  | coexpression\_cluster2\_10::2\_motifs | PSSM | 8.50 | 418 | | --- | --- | --- | --- | --- | --- | --- | --- |  **cluster\_5**   | Branch Motifs | | | | | | --- | --- | --- | --- | --- | | Node | Consensus | Logo | Logo (Reverse) | Collections | Matrix (Transfac format) | IC | Nb sites | | Singleton | **wwAgAACTTAww** |  |  | coexpression\_cluster2\_10 : 1 motif | PSSM | 8.92 | 180 | | --- | --- | --- | --- | --- | --- | --- | --- |  **cluster\_6**   | Branch Motifs | | | | | | --- | --- | --- | --- | --- | | Node | Consensus | Logo | Logo (Reverse) | Collections | Matrix (Transfac format) | IC | Nb sites | | Singleton | **kGCataCa** |  |  | coexpression\_cluster2\_10 : 1 motif | PSSM | 8.90 | 87 | | --- | --- | --- | --- | --- | --- | --- | --- |   - **Each cluster has a different color.** - **Click on the upper buttons to display separately the information of each cluster.** - **Click on the circles at each tree to display its corresponding merged motifs.**   Definitions  - **Logo tree.**  This results shows the hierarchical tree with its logo alignment of one cluster. The logos are shown in Forward (left logo) and Reverse (right logo) orientation. - **Branch-motifs.**  On the trees are displayed the branch number that is the number in which the motifs were incorporated in the tree. The Branch-Motifs table shows the logo in both orientations and the link to the file in TRANSFAC format with the branch-motif. |
|  | |  |  |  |  |  |  |  |  |  |  |  |  |  |  |  |  |  |  |  |  |  |  |  |  |  |  |  |  |  |  |  |  |  |  |  |  |  |  |  |  |  |  |  |  |  |  |  |  |  |  |  |  |  |  |  |  |  |  |  |  |  |  |  |  |  |  |  |  |  |  |  |  |  |  |  |  |  |  |  |  |  |  |  |  |  |  |  |  |  |  |  |  |  |  |  |  |  |  |  |  |  |  |  |  |  |  |  |  |  |  |  |  |  |  |  |  |  |  |  |  |  |  |  |  |  |  |  |  |  |  |  |  |  |  |  |  |  |  |  |  |  |  |  |  |  |  |  |  |  |  |  |  |  |  |  |  |  |  |  |  |  |  |  |  |  |  |  |  |  |  |  |  |  |  |  |  |  |  |  |  |  |  |  |  |  |  |  |  | | --- | --- | --- | --- | --- | --- | --- | --- | --- | --- | --- | --- | --- | --- | --- | --- | --- | --- | --- | --- | --- | --- | --- | --- | --- | --- | --- | --- | --- | --- | --- | --- | --- | --- | --- | --- | --- | --- | --- | --- | --- | --- | --- | --- | --- | --- | --- | --- | --- | --- | --- | --- | --- | --- | --- | --- | --- | --- | --- | --- | --- | --- | --- | --- | --- | --- | --- | --- | --- | --- | --- | --- | --- | --- | --- | --- | --- | --- | --- | --- | --- | --- | --- | --- | --- | --- | --- | --- | --- | --- | --- | --- | --- | --- | --- | --- | --- | --- | --- | --- | --- | --- | --- | --- | --- | --- | --- | --- | --- | --- | --- | --- | --- | --- | --- | --- | --- | --- | --- | --- | --- | --- | --- | --- | --- | --- | --- | --- | --- | --- | --- | --- | --- | --- | --- | --- | --- | --- | --- | --- | --- | --- | --- | --- | --- | --- | --- | --- | --- | --- | --- | --- | --- | --- | --- | --- | --- | --- | --- | --- | --- | --- | --- | --- | --- | --- | --- | --- | --- | --- | --- | --- | --- | --- | --- | --- | --- | --- | --- | --- | --- | --- | --- | --- | --- | --- | --- | --- | | **Individual Motif View**  Individual Motif View  | Motif id | Motif name | Cluster | Collection | Width | IC | Number of sites | Consensus | Consensus (Rev) | Logo | Logo (Rev) | | --- | --- | --- | --- | --- | --- | --- | --- | --- | --- | --- | | coexpression\_cluster2\_10\_m1\_cluster2\_10\_motif1\_r | cluster2\_10\_motif1\_r | cluster\_2 | coexpression\_cluster2\_10 | 24 | 13.39 | 1367 | **wwwATATATTATATAATATATwww** | **WWWATATATTATATAATATATWWW** |  |  | | coexpression\_cluster2\_10\_m14\_cluster2\_10\_motif14\_m | cluster2\_10\_motif14\_m | cluster\_3 | coexpression\_cluster2\_10 | 16 | 14.70 | 83 | **KTTTTTTTTTTAAT++** | **++ATTAAAAAAAAAAm** |  |  | | coexpression\_cluster2\_10\_m11\_cluster2\_10\_motif11\_m | cluster2\_10\_motif11\_m | cluster\_6 | coexpression\_cluster2\_10 | 8 | 8.90 | 87 | **TGCATACA** | **TGTATGCA** |  |  | | coexpression\_cluster2\_10\_m13\_cluster2\_10\_motif13\_m | cluster2\_10\_motif13\_m | cluster\_4 | coexpression\_cluster2\_10 | 12 | 6.57 | 111 | **+CCTTAA+++++** | **+++++TTAAGG+** |  |  | | coexpression\_cluster2\_10\_m12\_cluster2\_10\_motif12\_m | cluster2\_10\_motif12\_m | cluster\_3 | coexpression\_cluster2\_10 | 16 | 12.73 | 229 | **TTTTTyytyTTTTTy+** | **+RAAAAARARRAAAAA** |  |  | | coexpression\_cluster2\_10\_m16\_cluster2\_10\_motif16\_m | cluster2\_10\_motif16\_m | cluster\_3 | coexpression\_cluster2\_10 | 16 | 16.59 | 17 | **+CYAAAAAAAGTACAC** | **GTGTACTTTTTTTrG+** |  |  | | coexpression\_cluster2\_10\_m10\_cluster2\_10\_motif10\_m | cluster2\_10\_motif10\_m | cluster\_1 | coexpression\_cluster2\_10 | 16 | 13.49 | 307 | **TAyATAyATAyATAy+** | **+RTATRTATRTATRTA** |  |  | | coexpression\_cluster2\_10\_m2\_cluster2\_10\_motif2\_r | cluster2\_10\_motif2\_r | cluster\_2 | coexpression\_cluster2\_10 | 24 | 11.20 | 2337 | **+++++WWWATATATTATATATWWW** | **wwwATATATAATATATwww+++++** |  |  | | coexpression\_cluster2\_10\_m6\_cluster2\_10\_motif6\_r | cluster2\_10\_motif6\_r | cluster\_1 | coexpression\_cluster2\_10 | 16 | 9.20 | 1158 | **++wwATTAyATAww++** | **++WWTATRTAATWW++** |  |  | | coexpression\_cluster2\_10\_m9\_cluster2\_10\_motif9\_m | cluster2\_10\_motif9\_m | cluster\_1 | coexpression\_cluster2\_10 | 16 | 16.48 | 87 | **TATATRTATATATAT+** | **+ATATATATAyATATA** |  |  | | coexpression\_cluster2\_10\_m8\_cluster2\_10\_motif8\_m | cluster2\_10\_motif8\_m | cluster\_3 | coexpression\_cluster2\_10 | 16 | 14.95 | 168 | **TWTTATTATTTTTTT+** | **+AAAAAAATAATAAwA** |  |  | | coexpression\_cluster2\_10\_m15\_cluster2\_10\_motif15\_m | cluster2\_10\_motif15\_m | cluster\_1 | coexpression\_cluster2\_10 | 16 | 12.81 | 48 | **++++ATWATATAWGCA** | **TGCwTATATwAT++++** |  |  | | coexpression\_cluster2\_10\_m5\_cluster2\_10\_motif5\_r | cluster2\_10\_motif5\_r | cluster\_4 | coexpression\_cluster2\_10 | 12 | 8.45 | 307 | **wwCTTAAcAAww** | **WWTTGTTAAGWW** |  |  | | coexpression\_cluster2\_10\_m3\_cluster2\_10\_motif3\_r | cluster2\_10\_motif3\_r | cluster\_2 | coexpression\_cluster2\_10 | 24 | 11.95 | 1817 | **+++WWWTATTATATAATATATWWW** | **wwwATATATTATATAATAwww+++** |  |  | | coexpression\_cluster2\_10\_m7\_cluster2\_10\_motif7\_r | cluster2\_10\_motif7\_r | cluster\_5 | coexpression\_cluster2\_10 | 12 | 8.92 | 180 | **wwAgAACTTAww** | **WWTAAGTTCTWW** |  |  | | coexpression\_cluster2\_10\_m4\_cluster2\_10\_motif4\_r | cluster2\_10\_motif4\_r | cluster\_1 | coexpression\_cluster2\_10 | 16 | 9.35 | 285 | **++wwACTATAww++++** | **++++WWTATAGTWW++** |  |  |  - Click on the column names to change the order of the data. - Write the name of one cluster, collection or a pattern in the *Search* window. | | **Heatmap View**  Heatmap View Distance table | PDF | |

**Additional Files**

## Exported files

| File | Description |
| --- | --- |
| **Pairwise comparison** | This table shows the pairwise comparison between all the input motifs using different metrics. This is the *compare-matrices* result. |
| **Matrix description** | This table shows information of each input motif. |
| **Cluster-wise Motif Files [zip]** | One TRANSFAC file is generated for each cluster including all the input motifs grouped in the corresponding cluster. |
| **Root motifs** | A file with the root matrix of each cluster. |
| **Motif collections per cluster** | A tab separated file containing the counts of motif of each collection on each cluster. |
| **Clusters** | A tab separated file containing the clusters and their correspondng motifs. |
| **Central motifs IDs** | A tab separated file containing the central motif, that one who is the most similar to all the others motif in the same cluster. |
| **Central motifs (in TRANSFAC format)** | A transfac file containing the central motif, that one who is the most similar to all the others motif within the same cluster. |
| **Input motifs** | The input motifs analyzed with matrix-clustering. |

- **References**
  1. Castro-Mondragon JA et al.  RSAT matrix-clustering: dynamic exploration and redundancy reduction of transcription factor binding motif collections. doi: 10.1093/nar/gkx314; Nucleic Acids Research (2017)
  2. **Last RSAT release:** Nguyen NTT et al. RSAT 2018: regulatory sequence analysis tools 20th anniversary. Nucleic Acids Research (2018).
- **Additional Information**
  - Jacques van Helden course
- **Contact**
  - Jaime Castro-Mondragon - **j.a.c.mondragon@ncmm.uio.no**
  - Morgane Thomas-Chollier - **mthomas@biologie.ens.fr**
  - Jacques van Helden - **Jacques.van-Helden@univ-amu.fr**
