# Supplementary material for: Transcriptome analysis reveals a de novo DNA element that may interact with chromatin-associated proteins in Plasmodium berghei during erythrocytic development
Source: Sci Rep. 2025 May 28;15:18621. doi: 10.1038/s41598-025-03586-4 (PMC12120095; doi:10.1038/s41598-025-03586-4)
Supplement: Supplementary file 2 — Supplementary Information 2. [file 41598_2025_3586_MOESM2_ESM.zip › Supplementary File S2/Clustering/matrix-clustering_SUMMARY_cluster2_11.html]

matrix-clustering coexpression\_cluster2\_11


# RSAT - matrix-clustering result

### Analysis: coexpression\_cluster2\_11 (08/09/2023 15:46)

### Command

```
matrix-clustering  -v 1 -max_matrices 300 -matrix coexpression_cluster2_11 $RSAT/public_html/tmp/www-data/2023/09/08/matrix-clustering_2023-09-08.154551_5ZxetT/matrix-clustering_query_matrices.transfac transfac -hclust_method average -calc sum -title coexpression_cluster2_11 -metric_build_tree Ncor -lth w 5 -lth cor 0.6 -lth Ncor 0.4 -quick -label_in_tree name -return json,heatmap -o $RSAT/public_html/tmp/www-data/2023/09/08/matrix-clustering_2023-09-08.154551_5ZxetT/matrix-clustering
```


## Results Summary

|  |  |  |  |  |  |  |  |  |  |  |
| --- | --- | --- | --- | --- | --- | --- | --- | --- | --- | --- |
| Nb Input motifs | Nb Input collections | Nb Clusters Found | Download   Root motifs | Complete results [zip] | Parameters used  |  |  |  | | --- | --- | --- | | Linkage method | Similarity metric | Thresholds to partition the tree | | | |
| 14 | 1 | 8 | **Download** | Download | |  |  |  | | --- | --- | --- | | average | Ncor | **Ncor** = 0.4 **cor** = 0.6 | |

|  |  |
| --- | --- |
|  | **Logo Forest (dynamic browsing)**  **Logo Forest (rapid overview - low image quality)**  cluster\_1  cluster\_2  cluster\_3  cluster\_4  cluster\_5  cluster\_6  cluster\_7  cluster\_8 |
|  | |  |  |  |  |  |  |  |  |  |  | | --- | --- | --- | --- | --- | --- | --- | --- | --- | --- | | **Clusters Summary**  Clusters Summary Table  | Root Motif | Root Motif (Reverse) | Cluster ID | # Motifs | Motif\_Name::Collection | Number of Motifs by Collection | Root Motif (transfac Format) | | --- | --- | --- | --- | --- | --- | --- | |  | | --- | |  | | cluster\_1 | 5 | cluster2\_11\_motif10\_m::coexpression\_cluster2\_11:: cluster2\_11\_motif1\_r::coexpression\_cluster2\_11:: cluster2\_11\_motif2\_r::coexpression\_cluster2\_11:: cluster2\_11\_motif5\_m::coexpression\_cluster2\_11:: cluster2\_11\_motif6\_m::coexpression\_cluster2\_11:: | coexpression\_cluster2\_11::5\_motifs | Show matrix |
| cluster\_2 | 2 | cluster2\_11\_motif11\_m::coexpression\_cluster2\_11:: cluster2\_11\_motif8\_m::coexpression\_cluster2\_11:: | coexpression\_cluster2\_11::2\_motifs | Show matrix | | |
| cluster\_3 | 2 | cluster2\_11\_motif7\_m::coexpression\_cluster2\_11:: cluster2\_11\_motif9\_m::coexpression\_cluster2\_11:: | coexpression\_cluster2\_11::2\_motifs | Show matrix | | |
| cluster\_4 | 1 | cluster2\_11\_motif4\_r::coexpression\_cluster2\_11 | coexpression\_cluster2\_11 : 1 motif | Show matrix | | |
| cluster\_5 | 1 | cluster2\_11\_motif3\_r::coexpression\_cluster2\_11 | coexpression\_cluster2\_11 : 1 motif | Show matrix | | |
| cluster\_6 | 1 | cluster2\_11\_motif12\_m::coexpression\_cluster2\_11 | coexpression\_cluster2\_11 : 1 motif | Show matrix | | |
| cluster\_7 | 1 | cluster2\_11\_motif13\_m::coexpression\_cluster2\_11 | coexpression\_cluster2\_11 : 1 motif | Show matrix | | |
| cluster\_8 | 1 | cluster2\_11\_motif14\_m::coexpression\_cluster2\_11 | coexpression\_cluster2\_11 : 1 motif | Show matrix | | |

- Note: by default the table displays the first 50 clusters. Click on the *Show X entries* button to display more (or less) clusters.
- Click on the column names to change the order of the data.
- Use the *Search* window to find your motif (s) of interest.

| **Individual Cluster View**  Individual Cluster View **Show All**  **Hide All**  **cluster\_1**  **cluster\_2**  **cluster\_3**  **cluster\_4**  **cluster\_5**  **cluster\_6**  **cluster\_7**  **cluster\_8**  **Display Logo Trees**   **cluster\_1**    **cluster\_2**    **cluster\_3**    **cluster\_4**    **cluster\_5**    **cluster\_6**    **cluster\_7**    **cluster\_8**  **Display Branch-Motifs** **cluster\_1**   | Branch Motifs | | | | | | --- | --- | --- | --- | --- | | Node | Consensus | Logo | Logo (Reverse) | Collections | Matrix (Transfac format) | IC | Nb sites | | Node 1 | **--wwAAAgTGAaaww-** |  |  | coexpression\_cluster2\_11::2\_motifs | PSSM | 8.01 | 1192 | | --- | --- | --- | --- | --- | --- | --- | --- | | Node 2 | **-AAAAAarRawAAwAA** |  |  | coexpression\_cluster2\_11::2\_motifs | PSSM | 10.89 | 400 | | Node 3 | **-AaaAAArtgAAawaA** |  |  | coexpression\_cluster2\_11::4\_motifs | PSSM | 8.87 | 1591 | | Node 4 | **GAaaAAArtgAAawaA** |  |  | coexpression\_cluster2\_11::5\_motifs | PSSM | 9.91 | 1645 |  **cluster\_2**   | Branch Motifs | | | | | | --- | --- | --- | --- | --- | | Node | Consensus | Logo | Logo (Reverse) | Collections | Matrix (Transfac format) | IC | Nb sites | | Node 1 | **TaYrCACACAhw** |  |  | coexpression\_cluster2\_11::2\_motifs | PSSM | 10.18 | 103 | | --- | --- | --- | --- | --- | --- | --- | --- |  **cluster\_3**   | Branch Motifs | | | | | | --- | --- | --- | --- | --- | | Node | Consensus | Logo | Logo (Reverse) | Collections | Matrix (Transfac format) | IC | Nb sites | | Node 1 | **rTRTATryATATATR** |  |  | coexpression\_cluster2\_11::2\_motifs | PSSM | 12.86 | 404 | | --- | --- | --- | --- | --- | --- | --- | --- |  **cluster\_4**   | Branch Motifs | | | | | | --- | --- | --- | --- | --- | | Node | Consensus | Logo | Logo (Reverse) | Collections | Matrix (Transfac format) | IC | Nb sites | | Singleton | **wrCGTGCAtw** |  |  | coexpression\_cluster2\_11 : 1 motif | PSSM | 9.78 | 26 | | --- | --- | --- | --- | --- | --- | --- | --- |  **cluster\_5**   | Branch Motifs | | | | | | --- | --- | --- | --- | --- | | Node | Consensus | Logo | Logo (Reverse) | Collections | Matrix (Transfac format) | IC | Nb sites | | Singleton | **wwATAATGww** |  |  | coexpression\_cluster2\_11 : 1 motif | PSSM | 9.10 | 296 | | --- | --- | --- | --- | --- | --- | --- | --- |  **cluster\_6**   | Branch Motifs | | | | | | --- | --- | --- | --- | --- | | Node | Consensus | Logo | Logo (Reverse) | Collections | Matrix (Transfac format) | IC | Nb sites | | Singleton | **sSsvSMrSSaGCataCGmSkVmS** |  |  | coexpression\_cluster2\_11 : 1 motif | PSSM | 10.03 | 16 | | --- | --- | --- | --- | --- | --- | --- | --- |  **cluster\_7**   | Branch Motifs | | | | | | --- | --- | --- | --- | --- | | Node | Consensus | Logo | Logo (Reverse) | Collections | Matrix (Transfac format) | IC | Nb sites | | Singleton | **ksbssbSCRCaCaCaCSsSsvb** |  |  | coexpression\_cluster2\_11 : 1 motif | PSSM | 12.41 | 28 | | --- | --- | --- | --- | --- | --- | --- | --- |  **cluster\_8**   | Branch Motifs | | | | | | --- | --- | --- | --- | --- | | Node | Consensus | Logo | Logo (Reverse) | Collections | Matrix (Transfac format) | IC | Nb sites | | Singleton | **CSCCSSCa** |  |  | coexpression\_cluster2\_11 : 1 motif | PSSM | 8.11 | 14 | | --- | --- | --- | --- | --- | --- | --- | --- |   - **Each cluster has a different color.** - **Click on the upper buttons to display separately the information of each cluster.** - **Click on the circles at each tree to display its corresponding merged motifs.**   Definitions  - **Logo tree.**  This results shows the hierarchical tree with its logo alignment of one cluster. The logos are shown in Forward (left logo) and Reverse (right logo) orientation. - **Branch-motifs.**  On the trees are displayed the branch number that is the number in which the motifs were incorporated in the tree. The Branch-Motifs table shows the logo in both orientations and the link to the file in TRANSFAC format with the branch-motif. |
|  | |  |  |  |  |  |  |  |  |  |  |  |  |  |  |  |  |  |  |  |  |  |  |  |  |  |  |  |  |  |  |  |  |  |  |  |  |  |  |  |  |  |  |  |  |  |  |  |  |  |  |  |  |  |  |  |  |  |  |  |  |  |  |  |  |  |  |  |  |  |  |  |  |  |  |  |  |  |  |  |  |  |  |  |  |  |  |  |  |  |  |  |  |  |  |  |  |  |  |  |  |  |  |  |  |  |  |  |  |  |  |  |  |  |  |  |  |  |  |  |  |  |  |  |  |  |  |  |  |  |  |  |  |  |  |  |  |  |  |  |  |  |  |  |  |  |  |  |  |  |  |  |  |  |  |  |  |  |  |  |  |  |  |  |  |  |  | | --- | --- | --- | --- | --- | --- | --- | --- | --- | --- | --- | --- | --- | --- | --- | --- | --- | --- | --- | --- | --- | --- | --- | --- | --- | --- | --- | --- | --- | --- | --- | --- | --- | --- | --- | --- | --- | --- | --- | --- | --- | --- | --- | --- | --- | --- | --- | --- | --- | --- | --- | --- | --- | --- | --- | --- | --- | --- | --- | --- | --- | --- | --- | --- | --- | --- | --- | --- | --- | --- | --- | --- | --- | --- | --- | --- | --- | --- | --- | --- | --- | --- | --- | --- | --- | --- | --- | --- | --- | --- | --- | --- | --- | --- | --- | --- | --- | --- | --- | --- | --- | --- | --- | --- | --- | --- | --- | --- | --- | --- | --- | --- | --- | --- | --- | --- | --- | --- | --- | --- | --- | --- | --- | --- | --- | --- | --- | --- | --- | --- | --- | --- | --- | --- | --- | --- | --- | --- | --- | --- | --- | --- | --- | --- | --- | --- | --- | --- | --- | --- | --- | --- | --- | --- | --- | --- | --- | --- | --- | --- | --- | --- | --- | --- | --- | --- | | **Individual Motif View**  Individual Motif View  | Motif id | Motif name | Cluster | Collection | Width | IC | Number of sites | Consensus | Consensus (Rev) | Logo | Logo (Rev) | | --- | --- | --- | --- | --- | --- | --- | --- | --- | --- | --- | | coexpression\_cluster2\_11\_m6\_cluster2\_11\_motif6\_m | cluster2\_11\_motif6\_m | cluster\_1 | coexpression\_cluster2\_11 | 16 | 11.60 | 288 | **+ARAAAwrrrAAAAaA** | **TTTTTTYYYWTTTYT+** |  |  | | coexpression\_cluster2\_11\_m3\_cluster2\_11\_motif3\_r | cluster2\_11\_motif3\_r | cluster\_5 | coexpression\_cluster2\_11 | 10 | 9.10 | 296 | **wwATAATGww** | **WWCATTATWW** |  |  | | coexpression\_cluster2\_11\_m4\_cluster2\_11\_motif4\_r | cluster2\_11\_motif4\_r | cluster\_4 | coexpression\_cluster2\_11 | 10 | 9.78 | 26 | **wrCGTGCATw** | **WATGCACGYW** |  |  | | coexpression\_cluster2\_11\_m13\_cluster2\_11\_motif13\_m | cluster2\_11\_motif13\_m | cluster\_7 | coexpression\_cluster2\_11 | 22 | 12.41 | 28 | **TvtddtsCRCACACACrhrkmw** | **WKMYDYGTGTGTGYGSAHHABA** |  |  | | coexpression\_cluster2\_11\_m11\_cluster2\_11\_motif11\_m | cluster2\_11\_motif11\_m | cluster\_2 | coexpression\_cluster2\_11 | 12 | 10.31 | 35 | **+DYACACACAYW** | **wrTGTGTGTRh+** |  |  | | coexpression\_cluster2\_11\_m7\_cluster2\_11\_motif7\_m | cluster2\_11\_motif7\_m | cluster\_3 | coexpression\_cluster2\_11 | 15 | 12.62 | 282 | **rTryATryATATATR** | **YATATATRYATRYAY** |  |  | | coexpression\_cluster2\_11\_m1\_cluster2\_11\_motif1\_r | cluster2\_11\_motif1\_r | cluster\_1 | coexpression\_cluster2\_11 | 16 | 7.38 | 618 | **++wwAAAgTGAww+++** | **+++WWTCACTTTWW++** |  |  | | coexpression\_cluster2\_11\_m9\_cluster2\_11\_motif9\_m | cluster2\_11\_motif9\_m | cluster\_3 | coexpression\_cluster2\_11 | 15 | 12.00 | 122 | **+TATATGCATAT+++** | **+++ATATGCATATA+** |  |  | | coexpression\_cluster2\_11\_m12\_cluster2\_11\_motif12\_m | cluster2\_11\_motif12\_m | cluster\_6 | coexpression\_cluster2\_11 | 23 | 10.03 | 16 | **wmbmsmahkAGMATACrwkTrhr** | **YDYAMWYGTATKCTMDTKSKVKW** |  |  | | coexpression\_cluster2\_11\_m10\_cluster2\_11\_motif10\_m | cluster2\_11\_motif10\_m | cluster\_1 | coexpression\_cluster2\_11 | 16 | 10.98 | 54 | **YTTTTTTTCTC+++++** | **+++++GAGAAAaAAAr** |  |  | | coexpression\_cluster2\_11\_m5\_cluster2\_11\_motif5\_m | cluster2\_11\_motif5\_m | cluster\_1 | coexpression\_cluster2\_11 | 16 | 15.84 | 112 | **+AAAAAAAAATAATAw** | **WTATTATTTTTTTTT+** |  |  | | coexpression\_cluster2\_11\_m2\_cluster2\_11\_motif2\_r | cluster2\_11\_motif2\_r | cluster\_1 | coexpression\_cluster2\_11 | 16 | 8.27 | 574 | **++wwAAAgTGAAAww+** | **+WWTTTCACTTTWW++** |  |  | | coexpression\_cluster2\_11\_m14\_cluster2\_11\_motif14\_m | cluster2\_11\_motif14\_m | cluster\_8 | coexpression\_cluster2\_11 | 8 | 8.11 | 14 | **CCCCyCCA** | **TGGRGGGG** |  |  | | coexpression\_cluster2\_11\_m8\_cluster2\_11\_motif8\_m | cluster2\_11\_motif8\_m | cluster\_2 | coexpression\_cluster2\_11 | 12 | 11.24 | 68 | **TATgCACACAww** | **WWTGTGTGCATA** |  |  |  - Click on the column names to change the order of the data. - Write the name of one cluster, collection or a pattern in the *Search* window. | | **Heatmap View**  Heatmap View Distance table | PDF | |

**Additional Files**

## Exported files

| File | Description |
| --- | --- |
| **Pairwise comparison** | This table shows the pairwise comparison between all the input motifs using different metrics. This is the *compare-matrices* result. |
| **Matrix description** | This table shows information of each input motif. |
| **Cluster-wise Motif Files [zip]** | One TRANSFAC file is generated for each cluster including all the input motifs grouped in the corresponding cluster. |
| **Root motifs** | A file with the root matrix of each cluster. |
| **Motif collections per cluster** | A tab separated file containing the counts of motif of each collection on each cluster. |
| **Clusters** | A tab separated file containing the clusters and their correspondng motifs. |
| **Central motifs IDs** | A tab separated file containing the central motif, that one who is the most similar to all the others motif in the same cluster. |
| **Central motifs (in TRANSFAC format)** | A transfac file containing the central motif, that one who is the most similar to all the others motif within the same cluster. |
| **Input motifs** | The input motifs analyzed with matrix-clustering. |

- **References**
  1. Castro-Mondragon JA et al.  RSAT matrix-clustering: dynamic exploration and redundancy reduction of transcription factor binding motif collections. doi: 10.1093/nar/gkx314; Nucleic Acids Research (2017)
  2. **Last RSAT release:** Nguyen NTT et al. RSAT 2018: regulatory sequence analysis tools 20th anniversary. Nucleic Acids Research (2018).
- **Additional Information**
  - Jacques van Helden course
- **Contact**
  - Jaime Castro-Mondragon - **j.a.c.mondragon@ncmm.uio.no**
  - Morgane Thomas-Chollier - **mthomas@biologie.ens.fr**
  - Jacques van Helden - **Jacques.van-Helden@univ-amu.fr**
