# Supplementary material for: Transcriptome analysis reveals a de novo DNA element that may interact with chromatin-associated proteins in Plasmodium berghei during erythrocytic development
Source: Sci Rep. 2025 May 28;15:18621. doi: 10.1038/s41598-025-03586-4 (PMC12120095; doi:10.1038/s41598-025-03586-4)
Supplement: Supplementary file 2 — Supplementary Information 2. [file 41598_2025_3586_MOESM2_ESM.zip › Supplementary File S2/Clustering/matrix-clustering_SUMMARY_cluster2_2.html]

matrix-clustering coexpression\_cluster2\_2


# RSAT - matrix-clustering result

### Analysis: coexpression\_cluster2\_2 (08/09/2023 15:27)

### Command

```
matrix-clustering  -v 1 -max_matrices 300 -matrix coexpression_cluster2_2 $RSAT/public_html/tmp/www-data/2023/09/08/matrix-clustering_2023-09-08.152703_KrMovj/matrix-clustering_query_matrices.transfac transfac -hclust_method average -calc sum -title coexpression_cluster2_2 -metric_build_tree Ncor -lth w 5 -lth cor 0.6 -lth Ncor 0.4 -quick -label_in_tree name -return json,heatmap -o $RSAT/public_html/tmp/www-data/2023/09/08/matrix-clustering_2023-09-08.152703_KrMovj/matrix-clustering
```


## Results Summary

|  |  |  |  |  |  |  |  |  |  |  |
| --- | --- | --- | --- | --- | --- | --- | --- | --- | --- | --- |
| Nb Input motifs | Nb Input collections | Nb Clusters Found | Download   Root motifs | Complete results [zip] | Parameters used  |  |  |  | | --- | --- | --- | | Linkage method | Similarity metric | Thresholds to partition the tree | | | |
| 38 | 1 | 17 | **Download** | Download | |  |  |  | | --- | --- | --- | | average | Ncor | **Ncor** = 0.4 **cor** = 0.6 | |

|  |  |
| --- | --- |
|  | **Logo Forest (dynamic browsing)**  **Logo Forest (rapid overview - low image quality)**  cluster\_1  cluster\_2  cluster\_3  cluster\_4  cluster\_5  cluster\_6  cluster\_7  cluster\_8  cluster\_9  cluster\_10  cluster\_11  cluster\_12  cluster\_13  cluster\_14  cluster\_15  cluster\_16  cluster\_17 |
|  | |  |  |  |  |  |  |  |  |  |  | | --- | --- | --- | --- | --- | --- | --- | --- | --- | --- | | **Clusters Summary**  Clusters Summary Table  | Root Motif | Root Motif (Reverse) | Cluster ID | # Motifs | Motif\_Name::Collection | Number of Motifs by Collection | Root Motif (transfac Format) | | --- | --- | --- | --- | --- | --- | --- | |  | | --- | |  | | cluster\_1 | 13 | cluster2\_2\_motif14\_r::coexpression\_cluster2\_2:: cluster2\_2\_motif1\_r::coexpression\_cluster2\_2:: cluster2\_2\_motif6\_r::coexpression\_cluster2\_2:: cluster2\_2\_motif10\_r::coexpression\_cluster2\_2:: cluster2\_2\_motif9\_r::coexpression\_cluster2\_2:: cluster2\_2\_motif8\_r::coexpression\_cluster2\_2:: cluster2\_2\_motif13\_r::coexpression\_cluster2\_2:: cluster2\_2\_motif5\_r::coexpression\_cluster2\_2:: cluster2\_2\_motif12\_r::coexpression\_cluster2\_2:: cluster2\_2\_motif16\_r::coexpression\_cluster2\_2:: cluster2\_2\_motif7\_r::coexpression\_cluster2\_2:: cluster2\_2\_motif15\_r::coexpression\_cluster2\_2:: cluster2\_2\_motif2\_r::coexpression\_cluster2\_2:: | coexpression\_cluster2\_2::13\_motifs | Show matrix |
| cluster\_2 | 5 | cluster2\_2\_motif18\_r::coexpression\_cluster2\_2:: cluster2\_2\_motif19\_r::coexpression\_cluster2\_2:: cluster2\_2\_motif4\_r::coexpression\_cluster2\_2:: cluster2\_2\_motif11\_r::coexpression\_cluster2\_2:: cluster2\_2\_motif3\_r::coexpression\_cluster2\_2:: | coexpression\_cluster2\_2::5\_motifs | Show matrix | | |
| cluster\_3 | 2 | cluster2\_2\_motif22\_m::coexpression\_cluster2\_2:: cluster2\_2\_motif23\_m::coexpression\_cluster2\_2:: | coexpression\_cluster2\_2::2\_motifs | Show matrix | | |
| cluster\_4 | 3 | cluster2\_2\_motif37\_m::coexpression\_cluster2\_2:: cluster2\_2\_motif21\_m::coexpression\_cluster2\_2:: cluster2\_2\_motif32\_m::coexpression\_cluster2\_2:: | coexpression\_cluster2\_2::3\_motifs | Show matrix | | |
| cluster\_5 | 2 | cluster2\_2\_motif17\_r::coexpression\_cluster2\_2:: cluster2\_2\_motif36\_m::coexpression\_cluster2\_2:: | coexpression\_cluster2\_2::2\_motifs | Show matrix | | |
| cluster\_6 | 1 | cluster2\_2\_motif25\_m::coexpression\_cluster2\_2 | coexpression\_cluster2\_2 : 1 motif | Show matrix | | |
| cluster\_7 | 1 | cluster2\_2\_motif33\_m::coexpression\_cluster2\_2 | coexpression\_cluster2\_2 : 1 motif | Show matrix | | |
| cluster\_8 | 1 | cluster2\_2\_motif28\_m::coexpression\_cluster2\_2 | coexpression\_cluster2\_2 : 1 motif | Show matrix | | |
| cluster\_9 | 1 | cluster2\_2\_motif31\_m::coexpression\_cluster2\_2 | coexpression\_cluster2\_2 : 1 motif | Show matrix | | |
| cluster\_10 | 2 | cluster2\_2\_motif20\_m::coexpression\_cluster2\_2:: cluster2\_2\_motif29\_m::coexpression\_cluster2\_2:: | coexpression\_cluster2\_2::2\_motifs | Show matrix | | |
| cluster\_11 | 1 | cluster2\_2\_motif30\_m::coexpression\_cluster2\_2 | coexpression\_cluster2\_2 : 1 motif | Show matrix | | |
| cluster\_12 | 1 | cluster2\_2\_motif26\_m::coexpression\_cluster2\_2 | coexpression\_cluster2\_2 : 1 motif | Show matrix | | |
| cluster\_13 | 1 | cluster2\_2\_motif24\_m::coexpression\_cluster2\_2 | coexpression\_cluster2\_2 : 1 motif | Show matrix | | |
| cluster\_14 | 1 | cluster2\_2\_motif38\_m::coexpression\_cluster2\_2 | coexpression\_cluster2\_2 : 1 motif | Show matrix | | |
| cluster\_15 | 1 | cluster2\_2\_motif34\_m::coexpression\_cluster2\_2 | coexpression\_cluster2\_2 : 1 motif | Show matrix | | |
| cluster\_16 | 1 | cluster2\_2\_motif35\_m::coexpression\_cluster2\_2 | coexpression\_cluster2\_2 : 1 motif | Show matrix | | |
| cluster\_17 | 1 | cluster2\_2\_motif27\_m::coexpression\_cluster2\_2 | coexpression\_cluster2\_2 : 1 motif | Show matrix | | |

- Note: by default the table displays the first 50 clusters. Click on the *Show X entries* button to display more (or less) clusters.
- Click on the column names to change the order of the data.
- Use the *Search* window to find your motif (s) of interest.

| **Individual Cluster View**  Individual Cluster View **Show All**  **Hide All**  **cluster\_1**  **cluster\_2**  **cluster\_3**  **cluster\_4**  **cluster\_5**  **cluster\_6**  **cluster\_7**  **cluster\_8**  **cluster\_9**  **cluster\_10**  **cluster\_11**  **cluster\_12**  **cluster\_13**  **cluster\_14**  **cluster\_15**  **cluster\_16**  **cluster\_17**  **Display Logo Trees**   **cluster\_1**    **cluster\_2**    **cluster\_3**    **cluster\_4**    **cluster\_5**    **cluster\_6**    **cluster\_7**    **cluster\_8**    **cluster\_9**    **cluster\_10**    **cluster\_11**    **cluster\_12**    **cluster\_13**    **cluster\_14**    **cluster\_15**    **cluster\_16**    **cluster\_17**  **Display Branch-Motifs** **cluster\_1**   | Branch Motifs | | | | | | --- | --- | --- | --- | --- | | Node | Consensus | Logo | Logo (Reverse) | Collections | Matrix (Transfac format) | IC | Nb sites | | Node 1 | **-----wwwwwwTAATAATATAawTATAwAwaww-----------** |  |  | coexpression\_cluster2\_2::2\_motifs | PSSM | 14.33 | 3701 | | --- | --- | --- | --- | --- | --- | --- | --- | | Node 2 | **--wwwwwwwwwwwAtwATAATAAWwwAATATawAwwww------** |  |  | coexpression\_cluster2\_2::2\_motifs | PSSM | 17.21 | 2265 | | Node 3 | **--wwwwTwTwATAAwTATwATAATAwwAwwawwwww--------** |  |  | coexpression\_cluster2\_2::2\_motifs | PSSM | 16.53 | 2270 | | Node 4 | **--wwwwTwTwATAATTATwATAATAwwwwwawwwww--------** |  |  | coexpression\_cluster2\_2::3\_motifs | PSSM | 16.65 | 3200 | | Node 5 | **wwtwwwwwwwwTwwTwwTwwTAwwwwtwTwwTAATATAwwwwww** |  |  | coexpression\_cluster2\_2::2\_motifs | PSSM | 19.90 | 1537 | | Node 6 | **-----wwwwwwTAATwATAwwAwTwTAwAwwww-----------** |  |  | coexpression\_cluster2\_2::3\_motifs | PSSM | 13.42 | 5074 | | Node 7 | **wwtwwwwwtwwwwwTwwTwwTAwwwwTwTwwwwwwwTAwwwwww** |  |  | coexpression\_cluster2\_2::3\_motifs | PSSM | 19.83 | 2755 | | Node 8 | **--wwwwwwTwwTAATTATwATAATwwwawwwwwawwww------** |  |  | coexpression\_cluster2\_2::5\_motifs | PSSM | 16.57 | 5465 | | Node 9 | **----wwwwTwwwTAwTATwATAwwww------------------** |  |  | coexpression\_cluster2\_2::2\_motifs | PSSM | 11.09 | 4955 | | Node 10 | **--wwwwwwwwwTAATwATwwwAwTwwwwwwwwwawwww------** |  |  | coexpression\_cluster2\_2::8\_motifs | PSSM | 15.58 | 10539 | | Node 11 | **wwwwwwwwwwwTwATwWTwwwAwTwwwwwwwwwawwwwwwwwww** |  |  | coexpression\_cluster2\_2::11\_motifs | PSSM | 18.47 | 13294 | | Node 12 | **wwwwwwwwwwwwwAwwATwwwAwtwwwwwwwwwawwwwwwwwww** |  |  | coexpression\_cluster2\_2::13\_motifs | PSSM | 18.26 | 18249 |  **cluster\_2**   | Branch Motifs | | | | | | --- | --- | --- | --- | --- | | Node | Consensus | Logo | Logo (Reverse) | Collections | Matrix (Transfac format) | IC | Nb sites | | Node 1 | **wwTATmTAAAATww** |  |  | coexpression\_cluster2\_2::2\_motifs | PSSM | 9.42 | 2202 | | --- | --- | --- | --- | --- | --- | --- | --- | | Node 2 | **wwwTThTAAAATww** |  |  | coexpression\_cluster2\_2::2\_motifs | PSSM | 8.88 | 3492 | | Node 3 | **wwwwThTAAAATww** |  |  | coexpression\_cluster2\_2::4\_motifs | PSSM | 8.68 | 5694 | | Node 4 | **wwTwTwTAAwATww** |  |  | coexpression\_cluster2\_2::5\_motifs | PSSM | 7.92 | 8299 |  **cluster\_3**   | Branch Motifs | | | | | | --- | --- | --- | --- | --- | | Node | Consensus | Logo | Logo (Reverse) | Collections | Matrix (Transfac format) | IC | Nb sites | | Node 1 | **GSAKAGAwAAgGrAA** |  |  | coexpression\_cluster2\_2::2\_motifs | PSSM | 14.33 | 68 | | --- | --- | --- | --- | --- | --- | --- | --- |  **cluster\_4**   | Branch Motifs | | | | | | --- | --- | --- | --- | --- | | Node | Consensus | Logo | Logo (Reverse) | Collections | Matrix (Transfac format) | IC | Nb sites | | Node 1 | **--mCCCAATA** |  |  | coexpression\_cluster2\_2::2\_motifs | PSSM | 7.62 | 143 | | --- | --- | --- | --- | --- | --- | --- | --- | | Node 2 | **CrmCCCAATA** |  |  | coexpression\_cluster2\_2::3\_motifs | PSSM | 8.61 | 181 |  **cluster\_5**   | Branch Motifs | | | | | | --- | --- | --- | --- | --- | | Node | Consensus | Logo | Logo (Reverse) | Collections | Matrix (Transfac format) | IC | Nb sites | | Node 1 | **AwwAAACTAwwCGTA** |  |  | coexpression\_cluster2\_2::2\_motifs | PSSM | 15.24 | 217 | | --- | --- | --- | --- | --- | --- | --- | --- |  **cluster\_6**   | Branch Motifs | | | | | | --- | --- | --- | --- | --- | | Node | Consensus | Logo | Logo (Reverse) | Collections | Matrix (Transfac format) | IC | Nb sites | | Singleton | **tryrtrYryrYrtaY** |  |  | coexpression\_cluster2\_2 : 1 motif | PSSM | 12.51 | 179 | | --- | --- | --- | --- | --- | --- | --- | --- |  **cluster\_7**   | Branch Motifs | | | | | | --- | --- | --- | --- | --- | | Node | Consensus | Logo | Logo (Reverse) | Collections | Matrix (Transfac format) | IC | Nb sites | | Singleton | **taYataSGGYyaaaK** |  |  | coexpression\_cluster2\_2 : 1 motif | PSSM | 16.40 | 22 | | --- | --- | --- | --- | --- | --- | --- | --- |  **cluster\_8**   | Branch Motifs | | | | | | --- | --- | --- | --- | --- | | Node | Consensus | Logo | Logo (Reverse) | Collections | Matrix (Transfac format) | IC | Nb sites | | Singleton | **RaaaGatGyGCamaa** |  |  | coexpression\_cluster2\_2 : 1 motif | PSSM | 15.93 | 18 | | --- | --- | --- | --- | --- | --- | --- | --- |  **cluster\_9**   | Branch Motifs | | | | | | --- | --- | --- | --- | --- | | Node | Consensus | Logo | Logo (Reverse) | Collections | Matrix (Transfac format) | IC | Nb sites | | Singleton | **GCRCRCVtBSS** |  |  | coexpression\_cluster2\_2 : 1 motif | PSSM | 8.24 | 20 | | --- | --- | --- | --- | --- | --- | --- | --- |  **cluster\_10**   | Branch Motifs | | | | | | --- | --- | --- | --- | --- | | Node | Consensus | Logo | Logo (Reverse) | Collections | Matrix (Transfac format) | IC | Nb sites | | Node 1 | **GcATyAwTACTAATT** |  |  | coexpression\_cluster2\_2::2\_motifs | PSSM | 15.26 | 78 | | --- | --- | --- | --- | --- | --- | --- | --- |  **cluster\_11**   | Branch Motifs | | | | | | --- | --- | --- | --- | --- | | Node | Consensus | Logo | Logo (Reverse) | Collections | Matrix (Transfac format) | IC | Nb sites | | Singleton | **aGRCaaSRatRYaGC** |  |  | coexpression\_cluster2\_2 : 1 motif | PSSM | 14.32 | 12 | | --- | --- | --- | --- | --- | --- | --- | --- |  **cluster\_12**   | Branch Motifs | | | | | | --- | --- | --- | --- | --- | | Node | Consensus | Logo | Logo (Reverse) | Collections | Matrix (Transfac format) | IC | Nb sites | | Singleton | **tGCatyCaaaGRCtt** |  |  | coexpression\_cluster2\_2 : 1 motif | PSSM | 16.73 | 18 | | --- | --- | --- | --- | --- | --- | --- | --- |  **cluster\_13**   | Branch Motifs | | | | | | --- | --- | --- | --- | --- | | Node | Consensus | Logo | Logo (Reverse) | Collections | Matrix (Transfac format) | IC | Nb sites | | Singleton | **aatCaCCCaataaGG** |  |  | coexpression\_cluster2\_2 : 1 motif | PSSM | 17.50 | 13 | | --- | --- | --- | --- | --- | --- | --- | --- |  **cluster\_14**   | Branch Motifs | | | | | | --- | --- | --- | --- | --- | | Node | Consensus | Logo | Logo (Reverse) | Collections | Matrix (Transfac format) | IC | Nb sites | | Singleton | **CtattaaaG** |  |  | coexpression\_cluster2\_2 : 1 motif | PSSM | 10.22 | 34 | | --- | --- | --- | --- | --- | --- | --- | --- |  **cluster\_15**   | Branch Motifs | | | | | | --- | --- | --- | --- | --- | | Node | Consensus | Logo | Logo (Reverse) | Collections | Matrix (Transfac format) | IC | Nb sites | | Singleton | **tGCtCatatGaGtGt** |  |  | coexpression\_cluster2\_2 : 1 motif | PSSM | 20.79 | 5 | | --- | --- | --- | --- | --- | --- | --- | --- |  **cluster\_16**   | Branch Motifs | | | | | | --- | --- | --- | --- | --- | | Node | Consensus | Logo | Logo (Reverse) | Collections | Matrix (Transfac format) | IC | Nb sites | | Singleton | **CCCCttaaCCtaGaR** |  |  | coexpression\_cluster2\_2 : 1 motif | PSSM | 18.95 | 5 | | --- | --- | --- | --- | --- | --- | --- | --- |  **cluster\_17**   | Branch Motifs | | | | | | --- | --- | --- | --- | --- | | Node | Consensus | Logo | Logo (Reverse) | Collections | Matrix (Transfac format) | IC | Nb sites | | Singleton | **SGktkyaarCCSyGG** |  |  | coexpression\_cluster2\_2 : 1 motif | PSSM | 13.56 | 25 | | --- | --- | --- | --- | --- | --- | --- | --- |   - **Each cluster has a different color.** - **Click on the upper buttons to display separately the information of each cluster.** - **Click on the circles at each tree to display its corresponding merged motifs.**   Definitions  - **Logo tree.**  This results shows the hierarchical tree with its logo alignment of one cluster. The logos are shown in Forward (left logo) and Reverse (right logo) orientation. - **Branch-motifs.**  On the trees are displayed the branch number that is the number in which the motifs were incorporated in the tree. The Branch-Motifs table shows the logo in both orientations and the link to the file in TRANSFAC format with the branch-motif. |
|  | |  |  |  |  |  |  |  |  |  |  |  |  |  |  |  |  |  |  |  |  |  |  |  |  |  |  |  |  |  |  |  |  |  |  |  |  |  |  |  |  |  |  |  |  |  |  |  |  |  |  |  |  |  |  |  |  |  |  |  |  |  |  |  |  |  |  |  |  |  |  |  |  |  |  |  |  |  |  |  |  |  |  |  |  |  |  |  |  |  |  |  |  |  |  |  |  |  |  |  |  |  |  |  |  |  |  |  |  |  |  |  |  |  |  |  |  |  |  |  |  |  |  |  |  |  |  |  |  |  |  |  |  |  |  |  |  |  |  |  |  |  |  |  |  |  |  |  |  |  |  |  |  |  |  |  |  |  |  |  |  |  |  |  |  |  |  |  |  |  |  |  |  |  |  |  |  |  |  |  |  |  |  |  |  |  |  |  |  |  |  |  |  |  |  |  |  |  |  |  |  |  |  |  |  |  |  |  |  |  |  |  |  |  |  |  |  |  |  |  |  |  |  |  |  |  |  |  |  |  |  |  |  |  |  |  |  |  |  |  |  |  |  |  |  |  |  |  |  |  |  |  |  |  |  |  |  |  |  |  |  |  |  |  |  |  |  |  |  |  |  |  |  |  |  |  |  |  |  |  |  |  |  |  |  |  |  |  |  |  |  |  |  |  |  |  |  |  |  |  |  |  |  |  |  |  |  |  |  |  |  |  |  |  |  |  |  |  |  |  |  |  |  |  |  |  |  |  |  |  |  |  |  |  |  |  |  |  |  |  |  |  |  |  |  |  |  |  |  |  |  |  |  |  |  |  |  |  |  |  |  |  |  |  |  |  |  |  |  |  |  |  |  |  |  |  |  |  |  |  |  |  |  |  |  |  |  |  |  |  |  |  |  |  |  |  |  |  |  |  |  |  |  |  |  |  |  |  |  |  |  |  |  |  |  |  |  |  |  |  |  |  |  |  |  |  |  |  |  |  |  | | --- | --- | --- | --- | --- | --- | --- | --- | --- | --- | --- | --- | --- | --- | --- | --- | --- | --- | --- | --- | --- | --- | --- | --- | --- | --- | --- | --- | --- | --- | --- | --- | --- | --- | --- | --- | --- | --- | --- | --- | --- | --- | --- | --- | --- | --- | --- | --- | --- | --- | --- | --- | --- | --- | --- | --- | --- | --- | --- | --- | --- | --- | --- | --- | --- | --- | --- | --- | --- | --- | --- | --- | --- | --- | --- | --- | --- | --- | --- | --- | --- | --- | --- | --- | --- | --- | --- | --- | --- | --- | --- | --- | --- | --- | --- | --- | --- | --- | --- | --- | --- | --- | --- | --- | --- | --- | --- | --- | --- | --- | --- | --- | --- | --- | --- | --- | --- | --- | --- | --- | --- | --- | --- | --- | --- | --- | --- | --- | --- | --- | --- | --- | --- | --- | --- | --- | --- | --- | --- | --- | --- | --- | --- | --- | --- | --- | --- | --- | --- | --- | --- | --- | --- | --- | --- | --- | --- | --- | --- | --- | --- | --- | --- | --- | --- | --- | --- | --- | --- | --- | --- | --- | --- | --- | --- | --- | --- | --- | --- | --- | --- | --- | --- | --- | --- | --- | --- | --- | --- | --- | --- | --- | --- | --- | --- | --- | --- | --- | --- | --- | --- | --- | --- | --- | --- | --- | --- | --- | --- | --- | --- | --- | --- | --- | --- | --- | --- | --- | --- | --- | --- | --- | --- | --- | --- | --- | --- | --- | --- | --- | --- | --- | --- | --- | --- | --- | --- | --- | --- | --- | --- | --- | --- | --- | --- | --- | --- | --- | --- | --- | --- | --- | --- | --- | --- | --- | --- | --- | --- | --- | --- | --- | --- | --- | --- | --- | --- | --- | --- | --- | --- | --- | --- | --- | --- | --- | --- | --- | --- | --- | --- | --- | --- | --- | --- | --- | --- | --- | --- | --- | --- | --- | --- | --- | --- | --- | --- | --- | --- | --- | --- | --- | --- | --- | --- | --- | --- | --- | --- | --- | --- | --- | --- | --- | --- | --- | --- | --- | --- | --- | --- | --- | --- | --- | --- | --- | --- | --- | --- | --- | --- | --- | --- | --- | --- | --- | --- | --- | --- | --- | --- | --- | --- | --- | --- | --- | --- | --- | --- | --- | --- | --- | --- | --- | --- | --- | --- | --- | --- | --- | --- | --- | --- | --- | --- | --- | --- | --- | --- | --- | --- | --- | --- | --- | --- | --- | --- | --- | --- | --- | --- | --- | --- | --- | --- | --- | --- | --- | --- | --- | --- | --- | --- | --- | --- | --- | --- | --- | --- | --- | --- | --- | --- | --- | --- | --- | --- | --- | --- | --- | --- | --- | --- | --- | --- | --- | --- | --- | --- | --- | --- | --- | --- | --- | --- | --- | --- | --- | --- | --- | | **Individual Motif View**  Individual Motif View  | Motif id | Motif name | Cluster | Collection | Width | IC | Number of sites | Consensus | Consensus (Rev) | Logo | Logo (Rev) | | --- | --- | --- | --- | --- | --- | --- | --- | --- | --- | --- | | coexpression\_cluster2\_2\_m13\_cluster2\_2\_motif13\_r | cluster2\_2\_motif13\_r | cluster\_1 | coexpression\_cluster2\_2 | 44 | 14.30 | 2135 | **+++++WWWWWWTAATAATATAWWTATAWAWAWW+++++++++++** | **+++++++++++wwtwTwTATAwwTATATTATTAwwwwww+++++** |  |  | | coexpression\_cluster2\_2\_m8\_cluster2\_2\_motif8\_r | cluster2\_2\_motif8\_r | cluster\_1 | coexpression\_cluster2\_2 | 44 | 14.22 | 1373 | **+++++WWTWTWTWATTATAATAATWTAWAAWWW+++++++++++** | **+++++++++++wwwttwtAwATTATTATAATwAwAwAww+++++** |  |  | | coexpression\_cluster2\_2\_m36\_cluster2\_2\_motif36\_m | cluster2\_2\_motif36\_m | cluster\_5 | coexpression\_cluster2\_2 | 15 | 19.14 | 11 | **ARAAAACTATGCGTA** | **TACGCATAGTTTTYT** |  |  | | coexpression\_cluster2\_2\_m29\_cluster2\_2\_motif29\_m | cluster2\_2\_motif29\_m | cluster\_10 | coexpression\_cluster2\_2 | 15 | 15.86 | 29 | **GcwTCArTrCTwATT** | **AATWAGYAYTGAWGC** |  |  | | coexpression\_cluster2\_2\_m22\_cluster2\_2\_motif22\_m | cluster2\_2\_motif22\_m | cluster\_3 | coexpression\_cluster2\_2 | 15 | 10.46 | 46 | **++++AGAAAAgGrAA** | **TTYCCTTTTCT++++** |  |  | | coexpression\_cluster2\_2\_m21\_cluster2\_2\_motif21\_m | cluster2\_2\_motif21\_m | cluster\_4 | coexpression\_cluster2\_2 | 10 | 8.39 | 99 | **++ACCCAATA** | **TATTGGGT++** |  |  | | coexpression\_cluster2\_2\_m14\_cluster2\_2\_motif14\_r | cluster2\_2\_motif14\_r | cluster\_1 | coexpression\_cluster2\_2 | 44 | 11.87 | 3027 | **++++wwtwTwTATAATATTATAwwww++++++++++++++++++** | **++++++++++++++++++WWWWTATAATATTATAWAWAWW++++** |  |  | | coexpression\_cluster2\_2\_m16\_cluster2\_2\_motif16\_r | cluster2\_2\_motif16\_r | cluster\_1 | coexpression\_cluster2\_2 | 44 | 17.45 | 1165 | **++WWWWWWTWWWWWTWATAATAAWWWAATATWWAWWWW++++++** | **++++++wwwwTwwaTATTwwwTTATTATwawwwwwawwwwww++** |  |  | | coexpression\_cluster2\_2\_m37\_cluster2\_2\_motif37\_m | cluster2\_2\_motif37\_m | cluster\_4 | coexpression\_cluster2\_2 | 10 | 6.63 | 38 | **KRGGGKYG++** | **++CrmCCCym** |  |  | | coexpression\_cluster2\_2\_m27\_cluster2\_2\_motif27\_m | cluster2\_2\_motif27\_m | cluster\_17 | coexpression\_cluster2\_2 | 15 | 13.56 | 25 | **CRTTTTAAACCGTGG** | **CCACGGTTTAAAAYG** |  |  | | coexpression\_cluster2\_2\_m9\_cluster2\_2\_motif9\_r | cluster2\_2\_motif9\_r | cluster\_1 | coexpression\_cluster2\_2 | 44 | 19.62 | 864 | **+++wwwwtwTATwwwTATTATAwwwwtwTAATaATATAwwwwww** | **WWWWWWTATATTATTAWAWWWWTATAATAWWWATAWAWWWW+++** |  |  | | coexpression\_cluster2\_2\_m34\_cluster2\_2\_motif34\_m | cluster2\_2\_motif34\_m | cluster\_15 | coexpression\_cluster2\_2 | 15 | 20.79 | 5 | **TGCTCATATGAGTGT** | **ACACTCATATGAGCA** |  |  | | coexpression\_cluster2\_2\_m1\_cluster2\_2\_motif1\_r | cluster2\_2\_motif1\_r | cluster\_1 | coexpression\_cluster2\_2 | 44 | 12.13 | 1928 | **+++++wwwTwATTATTATAATAATww++++++++++++++++++** | **++++++++++++++++++WWATTATTATAATAATWAWWW+++++** |  |  | | coexpression\_cluster2\_2\_m18\_cluster2\_2\_motif18\_r | cluster2\_2\_motif18\_r | cluster\_2 | coexpression\_cluster2\_2 | 14 | 8.81 | 2605 | **WWTATTTAATWWW+** | **+wwwATTAAATAww** |  |  | | coexpression\_cluster2\_2\_m26\_cluster2\_2\_motif26\_m | cluster2\_2\_motif26\_m | cluster\_12 | coexpression\_cluster2\_2 | 15 | 16.73 | 18 | **TRCATTCAAAGrCTT** | **AAGYCTTTGAATGYA** |  |  | | coexpression\_cluster2\_2\_m11\_cluster2\_2\_motif11\_r | cluster2\_2\_motif11\_r | cluster\_2 | coexpression\_cluster2\_2 | 14 | 9.15 | 1801 | **WWTTTHTAAAATWW** | **wwATTTTAdAAAww** |  |  | | coexpression\_cluster2\_2\_m3\_cluster2\_2\_motif3\_r | cluster2\_2\_motif3\_r | cluster\_2 | coexpression\_cluster2\_2 | 14 | 9.33 | 1691 | **wwATThTAAAATww** | **WWATTTTADAATWW** |  |  | | coexpression\_cluster2\_2\_m5\_cluster2\_2\_motif5\_r | cluster2\_2\_motif5\_r | cluster\_1 | coexpression\_cluster2\_2 | 44 | 14.50 | 1566 | **+++++WWWWWWTAATAATATAAWTATAWAWAWW+++++++++++** | **+++++++++++wwtwTwTATAwTTATATTATTAwwwwww+++++** |  |  | | coexpression\_cluster2\_2\_m19\_cluster2\_2\_motif19\_r | cluster2\_2\_motif19\_r | cluster\_2 | coexpression\_cluster2\_2 | 14 | 10.55 | 403 | **wwwATmTAAAATww** | **WWATTTTAKATWWW** |  |  | | coexpression\_cluster2\_2\_m4\_cluster2\_2\_motif4\_r | cluster2\_2\_motif4\_r | cluster\_2 | coexpression\_cluster2\_2 | 14 | 9.36 | 1799 | **wwTAThTAAAATww** | **WWATTTTADATAWW** |  |  | | coexpression\_cluster2\_2\_m32\_cluster2\_2\_motif32\_m | cluster2\_2\_motif32\_m | cluster\_4 | coexpression\_cluster2\_2 | 10 | 6.48 | 44 | **++CCCCAA++** | **++TTGGGG++** |  |  | | coexpression\_cluster2\_2\_m20\_cluster2\_2\_motif20\_m | cluster2\_2\_motif20\_m | cluster\_10 | coexpression\_cluster2\_2 | 15 | 12.02 | 49 | **++AtTATTACTA+++** | **+++TAGTAATAAT++** |  |  | | coexpression\_cluster2\_2\_m35\_cluster2\_2\_motif35\_m | cluster2\_2\_motif35\_m | cluster\_16 | coexpression\_cluster2\_2 | 15 | 18.95 | 5 | **CCCCTTAACyTAGAr** | **YTCTARGTTAAGGGG** |  |  | | coexpression\_cluster2\_2\_m25\_cluster2\_2\_motif25\_m | cluster2\_2\_motif25\_m | cluster\_6 | coexpression\_cluster2\_2 | 15 | 12.51 | 179 | **TATATAyATRyATAy** | **RTATRYATRTATATA** |  |  | | coexpression\_cluster2\_2\_m2\_cluster2\_2\_motif2\_r | cluster2\_2\_motif2\_r | cluster\_1 | coexpression\_cluster2\_2 | 44 | 15.85 | 1249 | **++WWWWTWTWATAAWTATWATAATAWAWAWAWW+++++++++++** | **+++++++++++wwtwTwTwTATTATwATAwTTATwAwAwwww++** |  |  | | coexpression\_cluster2\_2\_m38\_cluster2\_2\_motif38\_m | cluster2\_2\_motif38\_m | cluster\_14 | coexpression\_cluster2\_2 | 9 | 10.22 | 34 | **CTATTAAAG** | **CTTTAATAG** |  |  | | coexpression\_cluster2\_2\_m23\_cluster2\_2\_motif23\_m | cluster2\_2\_motif23\_m | cluster\_3 | coexpression\_cluster2\_2 | 15 | 16.13 | 22 | **GSAKAGAWAAKGGAA** | **TTCCmTTwTCTMTSC** |  |  | | coexpression\_cluster2\_2\_m6\_cluster2\_2\_motif6\_r | cluster2\_2\_motif6\_r | cluster\_1 | coexpression\_cluster2\_2 | 44 | 18.24 | 1218 | **WWWWWWTATATWWWTAWWAATATWWWTATAWAWAWW++++++++** | **++++++++wwtwtwTATAwwwATATTWwTAWwWATATawwwwww** |  |  | | coexpression\_cluster2\_2\_m17\_cluster2\_2\_motif17\_r | cluster2\_2\_motif17\_r | cluster\_5 | coexpression\_cluster2\_2 | 15 | 9.38 | 206 | **+wwAAACTAww++++** | **++++WWTAGTTTWW+** |  |  | | coexpression\_cluster2\_2\_m33\_cluster2\_2\_motif33\_m | cluster2\_2\_motif33\_m | cluster\_7 | coexpression\_cluster2\_2 | 15 | 16.40 | 22 | **TAyATAGGGyTAAAk** | **MTTTARCCCTATRTA** |  |  | | coexpression\_cluster2\_2\_m12\_cluster2\_2\_motif12\_r | cluster2\_2\_motif12\_r | cluster\_1 | coexpression\_cluster2\_2 | 44 | 16.42 | 1100 | **+++++WWWWTWTWAWTATAATAATWWAATATAWAWAWW++++++** | **++++++wwtwTwtATATtwwATTATTATAwTwawawwww+++++** |  |  | | coexpression\_cluster2\_2\_m30\_cluster2\_2\_motif30\_m | cluster2\_2\_motif30\_m | cluster\_11 | coexpression\_cluster2\_2 | 15 | 14.32 | 12 | **AGryAAsrATryAGC** | **GCTRYATYSTTRYCT** |  |  | | coexpression\_cluster2\_2\_m28\_cluster2\_2\_motif28\_m | cluster2\_2\_motif28\_m | cluster\_8 | coexpression\_cluster2\_2 | 15 | 15.93 | 18 | **rAAAKATGTGCAMAA** | **TTKTGCACATMTTTY** |  |  | | coexpression\_cluster2\_2\_m31\_cluster2\_2\_motif31\_m | cluster2\_2\_motif31\_m | cluster\_9 | coexpression\_cluster2\_2 | 11 | 8.24 | 20 | **GCrYrCrWkgm** | **KCMWYGYRYGC** |  |  | | coexpression\_cluster2\_2\_m24\_cluster2\_2\_motif24\_m | cluster2\_2\_motif24\_m | cluster\_13 | coexpression\_cluster2\_2 | 15 | 17.50 | 13 | **AATCACCCAATAAGG** | **CCTTATTGGGTGATT** |  |  | | coexpression\_cluster2\_2\_m15\_cluster2\_2\_motif15\_r | cluster2\_2\_motif15\_r | cluster\_1 | coexpression\_cluster2\_2 | 44 | 17.13 | 1021 | **++WWWWWWTWATAATTATWATAATAWTAWAWAWWWW++++++++** | **++++++++wwwwtwtwTawTATTATwATAATTATwAwwwwww++** |  |  | | coexpression\_cluster2\_2\_m7\_cluster2\_2\_motif7\_r | cluster2\_2\_motif7\_r | cluster\_1 | coexpression\_cluster2\_2 | 44 | 14.38 | 930 | **++WWWWTTTWATAATTATWATAATAWWW++++++++++++++++** | **++++++++++++++++wwwTATTATWATAATTATwAAAwwww++** |  |  | | coexpression\_cluster2\_2\_m10\_cluster2\_2\_motif10\_r | cluster2\_2\_motif10\_r | cluster\_1 | coexpression\_cluster2\_2 | 44 | 21.48 | 673 | **WWTWWWTATAWWTATATWWTTAWWWWTWTWWTWATAWAWWWWWW** | **wwwwwwtWTATWAwwAwawwwwTAawWATATawwTATAwwwaww** |  |  |  - Click on the column names to change the order of the data. - Write the name of one cluster, collection or a pattern in the *Search* window. | | **Heatmap View**  Heatmap View Distance table | PDF | |

**Additional Files**

## Exported files

| File | Description |
| --- | --- |
| **Pairwise comparison** | This table shows the pairwise comparison between all the input motifs using different metrics. This is the *compare-matrices* result. |
| **Matrix description** | This table shows information of each input motif. |
| **Cluster-wise Motif Files [zip]** | One TRANSFAC file is generated for each cluster including all the input motifs grouped in the corresponding cluster. |
| **Root motifs** | A file with the root matrix of each cluster. |
| **Motif collections per cluster** | A tab separated file containing the counts of motif of each collection on each cluster. |
| **Clusters** | A tab separated file containing the clusters and their correspondng motifs. |
| **Central motifs IDs** | A tab separated file containing the central motif, that one who is the most similar to all the others motif in the same cluster. |
| **Central motifs (in TRANSFAC format)** | A transfac file containing the central motif, that one who is the most similar to all the others motif within the same cluster. |
| **Input motifs** | The input motifs analyzed with matrix-clustering. |

- **References**
  1. Castro-Mondragon JA et al.  RSAT matrix-clustering: dynamic exploration and redundancy reduction of transcription factor binding motif collections. doi: 10.1093/nar/gkx314; Nucleic Acids Research (2017)
  2. **Last RSAT release:** Nguyen NTT et al. RSAT 2018: regulatory sequence analysis tools 20th anniversary. Nucleic Acids Research (2018).
- **Additional Information**
  - Jacques van Helden course
- **Contact**
  - Jaime Castro-Mondragon - **j.a.c.mondragon@ncmm.uio.no**
  - Morgane Thomas-Chollier - **mthomas@biologie.ens.fr**
  - Jacques van Helden - **Jacques.van-Helden@univ-amu.fr**
