# Supplementary material for: Transcriptome analysis reveals a de novo DNA element that may interact with chromatin-associated proteins in Plasmodium berghei during erythrocytic development
Source: Sci Rep. 2025 May 28;15:18621. doi: 10.1038/s41598-025-03586-4 (PMC12120095; doi:10.1038/s41598-025-03586-4)
Supplement: Supplementary file 2 — Supplementary Information 2. [file 41598_2025_3586_MOESM2_ESM.zip › Supplementary File S2/Clustering/matrix-clustering_SUMMARY_cluster2_4.html]

matrix-clustering coexpression\_cluster2\_4


# RSAT - matrix-clustering result

### Analysis: coexpression\_cluster2\_4 (08/09/2023 15:33)

### Command

```
matrix-clustering  -v 1 -max_matrices 300 -matrix coexpression_cluster2_4 $RSAT/public_html/tmp/www-data/2023/09/08/matrix-clustering_2023-09-08.153321_0k34kN/matrix-clustering_query_matrices.transfac transfac -hclust_method average -calc sum -title coexpression_cluster2_4 -metric_build_tree Ncor -lth w 5 -lth cor 0.6 -lth Ncor 0.4 -quick -label_in_tree name -return json,heatmap -o $RSAT/public_html/tmp/www-data/2023/09/08/matrix-clustering_2023-09-08.153321_0k34kN/matrix-clustering
```


## Results Summary

|  |  |  |  |  |  |  |  |  |  |  |
| --- | --- | --- | --- | --- | --- | --- | --- | --- | --- | --- |
| Nb Input motifs | Nb Input collections | Nb Clusters Found | Download   Root motifs | Complete results [zip] | Parameters used  |  |  |  | | --- | --- | --- | | Linkage method | Similarity metric | Thresholds to partition the tree | | | |
| 15 | 1 | 11 | **Download** | Download | |  |  |  | | --- | --- | --- | | average | Ncor | **Ncor** = 0.4 **cor** = 0.6 | |

|  |  |
| --- | --- |
|  | **Logo Forest (dynamic browsing)**  **Logo Forest (rapid overview - low image quality)**  cluster\_1  cluster\_2  cluster\_3  cluster\_4  cluster\_5  cluster\_6  cluster\_7  cluster\_8  cluster\_9  cluster\_10  cluster\_11 |
|  | |  |  |  |  |  |  |  |  |  |  | | --- | --- | --- | --- | --- | --- | --- | --- | --- | --- | | **Clusters Summary**  Clusters Summary Table  | Root Motif | Root Motif (Reverse) | Cluster ID | # Motifs | Motif\_Name::Collection | Number of Motifs by Collection | Root Motif (transfac Format) | | --- | --- | --- | --- | --- | --- | --- | |  | | --- | |  | | cluster\_1 | 2 | cluster2\_4\_motif8\_m::coexpression\_cluster2\_4:: cluster2\_4\_motif9\_m::coexpression\_cluster2\_4:: | coexpression\_cluster2\_4::2\_motifs | Show matrix |
| cluster\_2 | 3 | cluster2\_4\_motif15\_m::coexpression\_cluster2\_4:: cluster2\_4\_motif10\_m::coexpression\_cluster2\_4:: cluster2\_4\_motif7\_m::coexpression\_cluster2\_4:: | coexpression\_cluster2\_4::3\_motifs | Show matrix | | |
| cluster\_3 | 2 | cluster2\_4\_motif11\_m::coexpression\_cluster2\_4:: cluster2\_4\_motif12\_m::coexpression\_cluster2\_4:: | coexpression\_cluster2\_4::2\_motifs | Show matrix | | |
| cluster\_4 | 1 | cluster2\_4\_motif2\_r::coexpression\_cluster2\_4 | coexpression\_cluster2\_4 : 1 motif | Show matrix | | |
| cluster\_5 | 1 | cluster2\_4\_motif5\_r::coexpression\_cluster2\_4 | coexpression\_cluster2\_4 : 1 motif | Show matrix | | |
| cluster\_6 | 1 | cluster2\_4\_motif3\_r::coexpression\_cluster2\_4 | coexpression\_cluster2\_4 : 1 motif | Show matrix | | |
| cluster\_7 | 1 | cluster2\_4\_motif4\_r::coexpression\_cluster2\_4 | coexpression\_cluster2\_4 : 1 motif | Show matrix | | |
| cluster\_8 | 1 | cluster2\_4\_motif6\_r::coexpression\_cluster2\_4 | coexpression\_cluster2\_4 : 1 motif | Show matrix | | |
| cluster\_9 | 1 | cluster2\_4\_motif1\_r::coexpression\_cluster2\_4 | coexpression\_cluster2\_4 : 1 motif | Show matrix | | |
| cluster\_10 | 1 | cluster2\_4\_motif13\_m::coexpression\_cluster2\_4 | coexpression\_cluster2\_4 : 1 motif | Show matrix | | |
| cluster\_11 | 1 | cluster2\_4\_motif14\_m::coexpression\_cluster2\_4 | coexpression\_cluster2\_4 : 1 motif | Show matrix | | |

- Note: by default the table displays the first 50 clusters. Click on the *Show X entries* button to display more (or less) clusters.
- Click on the column names to change the order of the data.
- Use the *Search* window to find your motif (s) of interest.

| **Individual Cluster View**  Individual Cluster View **Show All**  **Hide All**  **cluster\_1**  **cluster\_2**  **cluster\_3**  **cluster\_4**  **cluster\_5**  **cluster\_6**  **cluster\_7**  **cluster\_8**  **cluster\_9**  **cluster\_10**  **cluster\_11**  **Display Logo Trees**   **cluster\_1**    **cluster\_2**    **cluster\_3**    **cluster\_4**    **cluster\_5**    **cluster\_6**    **cluster\_7**    **cluster\_8**    **cluster\_9**    **cluster\_10**    **cluster\_11**  **Display Branch-Motifs** **cluster\_1**   | Branch Motifs | | | | | | --- | --- | --- | --- | --- | | Node | Consensus | Logo | Logo (Reverse) | Collections | Matrix (Transfac format) | IC | Nb sites | | Node 1 | **ATATATAyATryATR** |  |  | coexpression\_cluster2\_4::2\_motifs | PSSM | 12.83 | 373 | | --- | --- | --- | --- | --- | --- | --- | --- |  **cluster\_2**   | Branch Motifs | | | | | | --- | --- | --- | --- | --- | | Node | Consensus | Logo | Logo (Reverse) | Collections | Matrix (Transfac format) | IC | Nb sites | | Node 1 | **AAAAAAwaAdAaAAAA** |  |  | coexpression\_cluster2\_4::2\_motifs | PSSM | 12.77 | 326 | | --- | --- | --- | --- | --- | --- | --- | --- | | Node 2 | **AAAAAAwAAdAaAAAA** |  |  | coexpression\_cluster2\_4::3\_motifs | PSSM | 12.48 | 369 |  **cluster\_3**   | Branch Motifs | | | | | | --- | --- | --- | --- | --- | | Node | Consensus | Logo | Logo (Reverse) | Collections | Matrix (Transfac format) | IC | Nb sites | | Node 1 | **yTcrTCAyTT** |  |  | coexpression\_cluster2\_4::2\_motifs | PSSM | 8.02 | 145 | | --- | --- | --- | --- | --- | --- | --- | --- |  **cluster\_4**   | Branch Motifs | | | | | | --- | --- | --- | --- | --- | | Node | Consensus | Logo | Logo (Reverse) | Collections | Matrix (Transfac format) | IC | Nb sites | | Singleton | **wwCAAATGww** |  |  | coexpression\_cluster2\_4 : 1 motif | PSSM | 8.83 | 138 | | --- | --- | --- | --- | --- | --- | --- | --- |  **cluster\_5**   | Branch Motifs | | | | | | --- | --- | --- | --- | --- | | Node | Consensus | Logo | Logo (Reverse) | Collections | Matrix (Transfac format) | IC | Nb sites | | Singleton | **twCAATCGtt** |  |  | coexpression\_cluster2\_4 : 1 motif | PSSM | 8.83 | 22 | | --- | --- | --- | --- | --- | --- | --- | --- |  **cluster\_6**   | Branch Motifs | | | | | | --- | --- | --- | --- | --- | | Node | Consensus | Logo | Logo (Reverse) | Collections | Matrix (Transfac format) | IC | Nb sites | | Singleton | **wwGATGAArAww** |  |  | coexpression\_cluster2\_4 : 1 motif | PSSM | 9.16 | 105 | | --- | --- | --- | --- | --- | --- | --- | --- |  **cluster\_7**   | Branch Motifs | | | | | | --- | --- | --- | --- | --- | | Node | Consensus | Logo | Logo (Reverse) | Collections | Matrix (Transfac format) | IC | Nb sites | | Singleton | **wwgAAGCAAww** |  |  | coexpression\_cluster2\_4 : 1 motif | PSSM | 7.19 | 323 | | --- | --- | --- | --- | --- | --- | --- | --- |  **cluster\_8**   | Branch Motifs | | | | | | --- | --- | --- | --- | --- | | Node | Consensus | Logo | Logo (Reverse) | Collections | Matrix (Transfac format) | IC | Nb sites | | Singleton | **wwCCTTATAww** |  |  | coexpression\_cluster2\_4 : 1 motif | PSSM | 7.05 | 701 | | --- | --- | --- | --- | --- | --- | --- | --- |  **cluster\_9**   | Branch Motifs | | | | | | --- | --- | --- | --- | --- | | Node | Consensus | Logo | Logo (Reverse) | Collections | Matrix (Transfac format) | IC | Nb sites | | Singleton | **awAGGATCrw** |  |  | coexpression\_cluster2\_4 : 1 motif | PSSM | 8.88 | 21 | | --- | --- | --- | --- | --- | --- | --- | --- |  **cluster\_10**   | Branch Motifs | | | | | | --- | --- | --- | --- | --- | | Node | Consensus | Logo | Logo (Reverse) | Collections | Matrix (Transfac format) | IC | Nb sites | | Singleton | **SSSCCMCm** |  |  | coexpression\_cluster2\_4 : 1 motif | PSSM | 7.17 | 33 | | --- | --- | --- | --- | --- | --- | --- | --- |  **cluster\_11**   | Branch Motifs | | | | | | --- | --- | --- | --- | --- | | Node | Consensus | Logo | Logo (Reverse) | Collections | Matrix (Transfac format) | IC | Nb sites | | Singleton | **SaYCCCCyCcSamkY** |  |  | coexpression\_cluster2\_4 : 1 motif | PSSM | 11.20 | 18 | | --- | --- | --- | --- | --- | --- | --- | --- |   - **Each cluster has a different color.** - **Click on the upper buttons to display separately the information of each cluster.** - **Click on the circles at each tree to display its corresponding merged motifs.**   Definitions  - **Logo tree.**  This results shows the hierarchical tree with its logo alignment of one cluster. The logos are shown in Forward (left logo) and Reverse (right logo) orientation. - **Branch-motifs.**  On the trees are displayed the branch number that is the number in which the motifs were incorporated in the tree. The Branch-Motifs table shows the logo in both orientations and the link to the file in TRANSFAC format with the branch-motif. |
|  | |  |  |  |  |  |  |  |  |  |  |  |  |  |  |  |  |  |  |  |  |  |  |  |  |  |  |  |  |  |  |  |  |  |  |  |  |  |  |  |  |  |  |  |  |  |  |  |  |  |  |  |  |  |  |  |  |  |  |  |  |  |  |  |  |  |  |  |  |  |  |  |  |  |  |  |  |  |  |  |  |  |  |  |  |  |  |  |  |  |  |  |  |  |  |  |  |  |  |  |  |  |  |  |  |  |  |  |  |  |  |  |  |  |  |  |  |  |  |  |  |  |  |  |  |  |  |  |  |  |  |  |  |  |  |  |  |  |  |  |  |  |  |  |  |  |  |  |  |  |  |  |  |  |  |  |  |  |  |  |  |  |  |  |  |  |  |  |  |  |  |  |  |  |  |  |  |  | | --- | --- | --- | --- | --- | --- | --- | --- | --- | --- | --- | --- | --- | --- | --- | --- | --- | --- | --- | --- | --- | --- | --- | --- | --- | --- | --- | --- | --- | --- | --- | --- | --- | --- | --- | --- | --- | --- | --- | --- | --- | --- | --- | --- | --- | --- | --- | --- | --- | --- | --- | --- | --- | --- | --- | --- | --- | --- | --- | --- | --- | --- | --- | --- | --- | --- | --- | --- | --- | --- | --- | --- | --- | --- | --- | --- | --- | --- | --- | --- | --- | --- | --- | --- | --- | --- | --- | --- | --- | --- | --- | --- | --- | --- | --- | --- | --- | --- | --- | --- | --- | --- | --- | --- | --- | --- | --- | --- | --- | --- | --- | --- | --- | --- | --- | --- | --- | --- | --- | --- | --- | --- | --- | --- | --- | --- | --- | --- | --- | --- | --- | --- | --- | --- | --- | --- | --- | --- | --- | --- | --- | --- | --- | --- | --- | --- | --- | --- | --- | --- | --- | --- | --- | --- | --- | --- | --- | --- | --- | --- | --- | --- | --- | --- | --- | --- | --- | --- | --- | --- | --- | --- | --- | --- | --- | --- | --- | | **Individual Motif View**  Individual Motif View  | Motif id | Motif name | Cluster | Collection | Width | IC | Number of sites | Consensus | Consensus (Rev) | Logo | Logo (Rev) | | --- | --- | --- | --- | --- | --- | --- | --- | --- | --- | --- | | coexpression\_cluster2\_4\_m13\_cluster2\_4\_motif13\_m | cluster2\_4\_motif13\_m | cluster\_10 | coexpression\_cluster2\_4 | 8 | 7.17 | 33 | **ssCCCmMA** | **TKKGGGSS** |  |  | | coexpression\_cluster2\_4\_m9\_cluster2\_4\_motif9\_m | cluster2\_4\_motif9\_m | cluster\_1 | coexpression\_cluster2\_4 | 15 | 12.37 | 253 | **ATATATAyATryATr** | **YATRYATRTATATAT** |  |  | | coexpression\_cluster2\_4\_m14\_cluster2\_4\_motif14\_m | cluster2\_4\_motif14\_m | cluster\_11 | coexpression\_cluster2\_4 | 15 | 11.20 | 18 | **CAymCyCTChsAwTy** | **RAWTSDGAGRGKRTG** |  |  | | coexpression\_cluster2\_4\_m6\_cluster2\_4\_motif6\_r | cluster2\_4\_motif6\_r | cluster\_8 | coexpression\_cluster2\_4 | 11 | 7.05 | 701 | **wwCCTTATAww** | **WWTATAAGGWW** |  |  | | coexpression\_cluster2\_4\_m11\_cluster2\_4\_motif11\_m | cluster2\_4\_motif11\_m | cluster\_3 | coexpression\_cluster2\_4 | 10 | 8.42 | 102 | **+TCATCAcTT** | **AAGTGATGA+** |  |  | | coexpression\_cluster2\_4\_m15\_cluster2\_4\_motif15\_m | cluster2\_4\_motif15\_m | cluster\_2 | coexpression\_cluster2\_4 | 16 | 15.78 | 43 | **+TTTTTATTTTATTW+** | **+WAATAAAATAAAAA+** |  |  | | coexpression\_cluster2\_4\_m10\_cluster2\_4\_motif10\_m | cluster2\_4\_motif10\_m | cluster\_2 | coexpression\_cluster2\_4 | 16 | 12.79 | 223 | **AAAAAAwrArAwAAA+** | **+TTTWTYTYWTTTTTT** |  |  | | coexpression\_cluster2\_4\_m8\_cluster2\_4\_motif8\_m | cluster2\_4\_motif8\_m | cluster\_1 | coexpression\_cluster2\_4 | 15 | 14.72 | 120 | **+TATrTATATAyATA** | **TATRTATATAYATA+** |  |  | | coexpression\_cluster2\_4\_m5\_cluster2\_4\_motif5\_r | cluster2\_4\_motif5\_r | cluster\_5 | coexpression\_cluster2\_4 | 10 | 8.83 | 22 | **twCAATCGtt** | **AACGATTGWA** |  |  | | coexpression\_cluster2\_4\_m1\_cluster2\_4\_motif1\_r | cluster2\_4\_motif1\_r | cluster\_9 | coexpression\_cluster2\_4 | 10 | 8.88 | 21 | **awAGGATCrw** | **WYGATCCTWT** |  |  | | coexpression\_cluster2\_4\_m2\_cluster2\_4\_motif2\_r | cluster2\_4\_motif2\_r | cluster\_4 | coexpression\_cluster2\_4 | 10 | 8.83 | 138 | **wwCAAATGww** | **WWCATTTGWW** |  |  | | coexpression\_cluster2\_4\_m4\_cluster2\_4\_motif4\_r | cluster2\_4\_motif4\_r | cluster\_7 | coexpression\_cluster2\_4 | 11 | 7.19 | 323 | **wwgAAGCAAww** | **WWTTGCTTCWW** |  |  | | coexpression\_cluster2\_4\_m7\_cluster2\_4\_motif7\_m | cluster2\_4\_motif7\_m | cluster\_2 | coexpression\_cluster2\_4 | 16 | 14.86 | 103 | **+AAAAATAATAAAahA** | **TDTTTTATTATTTTT+** |  |  | | coexpression\_cluster2\_4\_m3\_cluster2\_4\_motif3\_r | cluster2\_4\_motif3\_r | cluster\_6 | coexpression\_cluster2\_4 | 12 | 9.16 | 105 | **wwGATGAArAww** | **WWTYTTCATCWW** |  |  | | coexpression\_cluster2\_4\_m12\_cluster2\_4\_motif12\_m | cluster2\_4\_motif12\_m | cluster\_3 | coexpression\_cluster2\_4 | 10 | 9.89 | 43 | **YTKGTCATTT** | **AAaTGACmAr** |  |  |  - Click on the column names to change the order of the data. - Write the name of one cluster, collection or a pattern in the *Search* window. | | **Heatmap View**  Heatmap View Distance table | PDF | |

**Additional Files**

## Exported files

| File | Description |
| --- | --- |
| **Pairwise comparison** | This table shows the pairwise comparison between all the input motifs using different metrics. This is the *compare-matrices* result. |
| **Matrix description** | This table shows information of each input motif. |
| **Cluster-wise Motif Files [zip]** | One TRANSFAC file is generated for each cluster including all the input motifs grouped in the corresponding cluster. |
| **Root motifs** | A file with the root matrix of each cluster. |
| **Motif collections per cluster** | A tab separated file containing the counts of motif of each collection on each cluster. |
| **Clusters** | A tab separated file containing the clusters and their correspondng motifs. |
| **Central motifs IDs** | A tab separated file containing the central motif, that one who is the most similar to all the others motif in the same cluster. |
| **Central motifs (in TRANSFAC format)** | A transfac file containing the central motif, that one who is the most similar to all the others motif within the same cluster. |
| **Input motifs** | The input motifs analyzed with matrix-clustering. |

- **References**
  1. Castro-Mondragon JA et al.  RSAT matrix-clustering: dynamic exploration and redundancy reduction of transcription factor binding motif collections. doi: 10.1093/nar/gkx314; Nucleic Acids Research (2017)
  2. **Last RSAT release:** Nguyen NTT et al. RSAT 2018: regulatory sequence analysis tools 20th anniversary. Nucleic Acids Research (2018).
- **Additional Information**
  - Jacques van Helden course
- **Contact**
  - Jaime Castro-Mondragon - **j.a.c.mondragon@ncmm.uio.no**
  - Morgane Thomas-Chollier - **mthomas@biologie.ens.fr**
  - Jacques van Helden - **Jacques.van-Helden@univ-amu.fr**
