# Supplementary material for: Transcriptome analysis reveals a de novo DNA element that may interact with chromatin-associated proteins in Plasmodium berghei during erythrocytic development
Source: Sci Rep. 2025 May 28;15:18621. doi: 10.1038/s41598-025-03586-4 (PMC12120095; doi:10.1038/s41598-025-03586-4)
Supplement: Supplementary file 2 — Supplementary Information 2. [file 41598_2025_3586_MOESM2_ESM.zip › Supplementary File S2/Clustering/matrix-clustering_SUMMARY_cluster3_5.html]

matrix-clustering coexpression\_cluster3\_5


# RSAT - matrix-clustering result

### Analysis: coexpression\_cluster3\_5 (08/09/2023 15:54)

### Command

```
matrix-clustering  -v 1 -max_matrices 300 -matrix coexpression_cluster3_5 $RSAT/public_html/tmp/www-data/2023/09/08/matrix-clustering_2023-09-08.155430_2GIcCG/matrix-clustering_query_matrices.transfac transfac -hclust_method average -calc sum -title coexpression_cluster3_5 -metric_build_tree Ncor -lth w 5 -lth cor 0.6 -lth Ncor 0.4 -quick -label_in_tree name -return json,heatmap -o $RSAT/public_html/tmp/www-data/2023/09/08/matrix-clustering_2023-09-08.155430_2GIcCG/matrix-clustering
```


## Results Summary

|  |  |  |  |  |  |  |  |  |  |  |
| --- | --- | --- | --- | --- | --- | --- | --- | --- | --- | --- |
| Nb Input motifs | Nb Input collections | Nb Clusters Found | Download   Root motifs | Complete results [zip] | Parameters used  |  |  |  | | --- | --- | --- | | Linkage method | Similarity metric | Thresholds to partition the tree | | | |
| 34 | 1 | 9 | **Download** | Download | |  |  |  | | --- | --- | --- | | average | Ncor | **Ncor** = 0.4 **cor** = 0.6 | |

|  |  |
| --- | --- |
|  | **Logo Forest (dynamic browsing)**  **Logo Forest (rapid overview - low image quality)**  cluster\_1  cluster\_2  cluster\_3  cluster\_4  cluster\_5  cluster\_6  cluster\_7  cluster\_8  cluster\_9 |
|  | |  |  |  |  |  |  |  |  |  |  | | --- | --- | --- | --- | --- | --- | --- | --- | --- | --- | | **Clusters Summary**  Clusters Summary Table  | Root Motif | Root Motif (Reverse) | Cluster ID | # Motifs | Motif\_Name::Collection | Number of Motifs by Collection | Root Motif (transfac Format) | | --- | --- | --- | --- | --- | --- | --- | |  | | --- | |  | | cluster\_1 | 15 | cluster3\_5\_motif32\_m::coexpression\_cluster3\_5:: cluster3\_5\_motif11\_r::coexpression\_cluster3\_5:: cluster3\_5\_motif12\_r::coexpression\_cluster3\_5:: cluster3\_5\_motif2\_r::coexpression\_cluster3\_5:: cluster3\_5\_motif6\_r::coexpression\_cluster3\_5:: cluster3\_5\_motif7\_r::coexpression\_cluster3\_5:: cluster3\_5\_motif3\_r::coexpression\_cluster3\_5:: cluster3\_5\_motif5\_r::coexpression\_cluster3\_5:: cluster3\_5\_motif10\_r::coexpression\_cluster3\_5:: cluster3\_5\_motif1\_r::coexpression\_cluster3\_5:: cluster3\_5\_motif16\_r::coexpression\_cluster3\_5:: cluster3\_5\_motif8\_r::coexpression\_cluster3\_5:: cluster3\_5\_motif23\_m::coexpression\_cluster3\_5:: cluster3\_5\_motif26\_m::coexpression\_cluster3\_5:: cluster3\_5\_motif28\_m::coexpression\_cluster3\_5:: | coexpression\_cluster3\_5::15\_motifs | Show matrix |
| cluster\_2 | 10 | cluster3\_5\_motif31\_m::coexpression\_cluster3\_5:: cluster3\_5\_motif9\_r::coexpression\_cluster3\_5:: cluster3\_5\_motif24\_m::coexpression\_cluster3\_5:: cluster3\_5\_motif25\_m::coexpression\_cluster3\_5:: cluster3\_5\_motif29\_m::coexpression\_cluster3\_5:: cluster3\_5\_motif13\_r::coexpression\_cluster3\_5:: cluster3\_5\_motif19\_r::coexpression\_cluster3\_5:: cluster3\_5\_motif4\_r::coexpression\_cluster3\_5:: cluster3\_5\_motif14\_r::coexpression\_cluster3\_5:: cluster3\_5\_motif34\_m::coexpression\_cluster3\_5:: | coexpression\_cluster3\_5::10\_motifs | Show matrix | | |
| cluster\_3 | 2 | cluster3\_5\_motif15\_r::coexpression\_cluster3\_5:: cluster3\_5\_motif21\_r::coexpression\_cluster3\_5:: | coexpression\_cluster3\_5::2\_motifs | Show matrix | | |
| cluster\_4 | 2 | cluster3\_5\_motif20\_r::coexpression\_cluster3\_5:: cluster3\_5\_motif22\_r::coexpression\_cluster3\_5:: | coexpression\_cluster3\_5::2\_motifs | Show matrix | | |
| cluster\_5 | 1 | cluster3\_5\_motif30\_m::coexpression\_cluster3\_5 | coexpression\_cluster3\_5 : 1 motif | Show matrix | | |
| cluster\_6 | 1 | cluster3\_5\_motif18\_r::coexpression\_cluster3\_5 | coexpression\_cluster3\_5 : 1 motif | Show matrix | | |
| cluster\_7 | 1 | cluster3\_5\_motif33\_m::coexpression\_cluster3\_5 | coexpression\_cluster3\_5 : 1 motif | Show matrix | | |
| cluster\_8 | 1 | cluster3\_5\_motif27\_m::coexpression\_cluster3\_5 | coexpression\_cluster3\_5 : 1 motif | Show matrix | | |
| cluster\_9 | 1 | cluster3\_5\_motif17\_r::coexpression\_cluster3\_5 | coexpression\_cluster3\_5 : 1 motif | Show matrix | | |

- Note: by default the table displays the first 50 clusters. Click on the *Show X entries* button to display more (or less) clusters.
- Click on the column names to change the order of the data.
- Use the *Search* window to find your motif (s) of interest.

| **Individual Cluster View**  Individual Cluster View **Show All**  **Hide All**  **cluster\_1**  **cluster\_2**  **cluster\_3**  **cluster\_4**  **cluster\_5**  **cluster\_6**  **cluster\_7**  **cluster\_8**  **cluster\_9**  **Display Logo Trees**   **cluster\_1**    **cluster\_2**    **cluster\_3**    **cluster\_4**    **cluster\_5**    **cluster\_6**    **cluster\_7**    **cluster\_8**    **cluster\_9**  **Display Branch-Motifs** **cluster\_1**   | Branch Motifs | | | | | | --- | --- | --- | --- | --- | | Node | Consensus | Logo | Logo (Reverse) | Collections | Matrix (Transfac format) | IC | Nb sites | | Node 1 | **-waAAAAgACAawww** |  |  | coexpression\_cluster3\_5::2\_motifs | PSSM | 9.16 | 1941 | | --- | --- | --- | --- | --- | --- | --- | --- | | Node 2 | **-waAAAArAmAAAww** |  |  | coexpression\_cluster3\_5::3\_motifs | PSSM | 9.26 | 5455 | | Node 3 | **-wwTATAkAcAAAww** |  |  | coexpression\_cluster3\_5::2\_motifs | PSSM | 8.70 | 3205 | | Node 4 | **-waAAAArAmAAwww** |  |  | coexpression\_cluster3\_5::4\_motifs | PSSM | 9.07 | 7669 | | Node 5 | **-wwTATAgAcAAAww** |  |  | coexpression\_cluster3\_5::3\_motifs | PSSM | 8.72 | 4017 | | Node 6 | **-waAAAArAmAAwww** |  |  | coexpression\_cluster3\_5::5\_motifs | PSSM | 8.97 | 8906 | | Node 7 | **-wwwATAgAcAAAww** |  |  | coexpression\_cluster3\_5::4\_motifs | PSSM | 8.58 | 6360 | | Node 8 | **-waAAAArAmAwAww** |  |  | coexpression\_cluster3\_5::6\_motifs | PSSM | 8.82 | 11727 | | Node 9 | **-waAAAArAmAwAww** |  |  | coexpression\_cluster3\_5::7\_motifs | PSSM | 8.73 | 12788 | | Node 10 | **AAAAAAAaraAAwAA** |  |  | coexpression\_cluster3\_5::2\_motifs | PSSM | 11.83 | 550 | | Node 11 | **-wwAAwArAmAAAww** |  |  | coexpression\_cluster3\_5::11\_motifs | PSSM | 8.11 | 19148 | | Node 12 | **AAAwAAAAwaAAwAA** |  |  | coexpression\_cluster3\_5::3\_motifs | PSSM | 11.18 | 856 | | Node 13 | **AwwAAwArAmAAAww** |  |  | coexpression\_cluster3\_5::14\_motifs | PSSM | 8.92 | 20004 | | Node 14 | **AwwAAwArAmAAAww** |  |  | coexpression\_cluster3\_5::15\_motifs | PSSM | 8.90 | 20117 |  **cluster\_2**   | Branch Motifs | | | | | | --- | --- | --- | --- | --- | | Node | Consensus | Logo | Logo (Reverse) | Collections | Matrix (Transfac format) | IC | Nb sites | | Node 1 | **--wwwtgTGTGTGCATww----** |  |  | coexpression\_cluster3\_5::2\_motifs | PSSM | 9.46 | 867 | | --- | --- | --- | --- | --- | --- | --- | --- | | Node 2 | **---TATaTrYaTrTaTRYA---** |  |  | coexpression\_cluster3\_5::2\_motifs | PSSM | 12.39 | 1013 | | Node 3 | **---taTATrTaTATAtaYA---** |  |  | coexpression\_cluster3\_5::3\_motifs | PSSM | 11.69 | 4256 | | Node 4 | **---wwTGTGTGTAya-------** |  |  | coexpression\_cluster3\_5::2\_motifs | PSSM | 8.37 | 1037 | | Node 5 | **--wwwTGTGTGTryATww----** |  |  | coexpression\_cluster3\_5::4\_motifs | PSSM | 9.00 | 1904 | | Node 6 | **--wwwTrTrTGTryATww----** |  |  | coexpression\_cluster3\_5::5\_motifs | PSSM | 8.59 | 2847 | | Node 7 | **--wwwTrTrTGTryATww----** |  |  | coexpression\_cluster3\_5::6\_motifs | PSSM | 8.57 | 3055 | | Node 8 | **--wtaTaTrTrTryATatA---** |  |  | coexpression\_cluster3\_5::9\_motifs | PSSM | 9.97 | 7311 | | Node 9 | **cywtaTrTrTrTryATatasaw** |  |  | coexpression\_cluster3\_5::10\_motifs | PSSM | 9.68 | 7442 |  **cluster\_3**   | Branch Motifs | | | | | | --- | --- | --- | --- | --- | | Node | Consensus | Logo | Logo (Reverse) | Collections | Matrix (Transfac format) | IC | Nb sites | | Node 1 | **wwAGAAwTww** |  |  | coexpression\_cluster3\_5::2\_motifs | PSSM | 8.48 | 749 | | --- | --- | --- | --- | --- | --- | --- | --- |  **cluster\_4**   | Branch Motifs | | | | | | --- | --- | --- | --- | --- | | Node | Consensus | Logo | Logo (Reverse) | Collections | Matrix (Transfac format) | IC | Nb sites | | Node 1 | **wwwTCTATAww** |  |  | coexpression\_cluster3\_5::2\_motifs | PSSM | 8.70 | 625 | | --- | --- | --- | --- | --- | --- | --- | --- |  **cluster\_5**   | Branch Motifs | | | | | | --- | --- | --- | --- | --- | | Node | Consensus | Logo | Logo (Reverse) | Collections | Matrix (Transfac format) | IC | Nb sites | | Singleton | **aaataataataat** |  |  | coexpression\_cluster3\_5 : 1 motif | PSSM | 13.83 | 147 | | --- | --- | --- | --- | --- | --- | --- | --- |  **cluster\_6**   | Branch Motifs | | | | | | --- | --- | --- | --- | --- | | Node | Consensus | Logo | Logo (Reverse) | Collections | Matrix (Transfac format) | IC | Nb sites | | Singleton | **wwAAGTGTdw** |  |  | coexpression\_cluster3\_5 : 1 motif | PSSM | 9.15 | 186 | | --- | --- | --- | --- | --- | --- | --- | --- |  **cluster\_7**   | Branch Motifs | | | | | | --- | --- | --- | --- | --- | | Node | Consensus | Logo | Logo (Reverse) | Collections | Matrix (Transfac format) | IC | Nb sites | | Singleton | **aaaCCGwGGraaa** |  |  | coexpression\_cluster3\_5 : 1 motif | PSSM | 10.63 | 92 | | --- | --- | --- | --- | --- | --- | --- | --- |  **cluster\_8**   | Branch Motifs | | | | | | --- | --- | --- | --- | --- | | Node | Consensus | Logo | Logo (Reverse) | Collections | Matrix (Transfac format) | IC | Nb sites | | Singleton | **yaaGaCa** |  |  | coexpression\_cluster3\_5 : 1 motif | PSSM | 7.71 | 360 | | --- | --- | --- | --- | --- | --- | --- | --- |  **cluster\_9**   | Branch Motifs | | | | | | --- | --- | --- | --- | --- | | Node | Consensus | Logo | Logo (Reverse) | Collections | Matrix (Transfac format) | IC | Nb sites | | Singleton | **raAACGCCww** |  |  | coexpression\_cluster3\_5 : 1 motif | PSSM | 9.08 | 36 | | --- | --- | --- | --- | --- | --- | --- | --- |   - **Each cluster has a different color.** - **Click on the upper buttons to display separately the information of each cluster.** - **Click on the circles at each tree to display its corresponding merged motifs.**   Definitions  - **Logo tree.**  This results shows the hierarchical tree with its logo alignment of one cluster. The logos are shown in Forward (left logo) and Reverse (right logo) orientation. - **Branch-motifs.**  On the trees are displayed the branch number that is the number in which the motifs were incorporated in the tree. The Branch-Motifs table shows the logo in both orientations and the link to the file in TRANSFAC format with the branch-motif. |
|  | |  |  |  |  |  |  |  |  |  |  |  |  |  |  |  |  |  |  |  |  |  |  |  |  |  |  |  |  |  |  |  |  |  |  |  |  |  |  |  |  |  |  |  |  |  |  |  |  |  |  |  |  |  |  |  |  |  |  |  |  |  |  |  |  |  |  |  |  |  |  |  |  |  |  |  |  |  |  |  |  |  |  |  |  |  |  |  |  |  |  |  |  |  |  |  |  |  |  |  |  |  |  |  |  |  |  |  |  |  |  |  |  |  |  |  |  |  |  |  |  |  |  |  |  |  |  |  |  |  |  |  |  |  |  |  |  |  |  |  |  |  |  |  |  |  |  |  |  |  |  |  |  |  |  |  |  |  |  |  |  |  |  |  |  |  |  |  |  |  |  |  |  |  |  |  |  |  |  |  |  |  |  |  |  |  |  |  |  |  |  |  |  |  |  |  |  |  |  |  |  |  |  |  |  |  |  |  |  |  |  |  |  |  |  |  |  |  |  |  |  |  |  |  |  |  |  |  |  |  |  |  |  |  |  |  |  |  |  |  |  |  |  |  |  |  |  |  |  |  |  |  |  |  |  |  |  |  |  |  |  |  |  |  |  |  |  |  |  |  |  |  |  |  |  |  |  |  |  |  |  |  |  |  |  |  |  |  |  |  |  |  |  |  |  |  |  |  |  |  |  |  |  |  |  |  |  |  |  |  |  |  |  |  |  |  |  |  |  |  |  |  |  |  |  |  |  |  |  |  |  |  |  |  |  |  |  |  |  |  |  |  |  |  |  |  |  |  |  |  |  |  |  |  |  |  |  |  |  |  |  |  |  |  |  |  |  |  |  |  |  |  |  |  |  |  |  |  |  |  |  |  |  |  |  |  |  | | --- | --- | --- | --- | --- | --- | --- | --- | --- | --- | --- | --- | --- | --- | --- | --- | --- | --- | --- | --- | --- | --- | --- | --- | --- | --- | --- | --- | --- | --- | --- | --- | --- | --- | --- | --- | --- | --- | --- | --- | --- | --- | --- | --- | --- | --- | --- | --- | --- | --- | --- | --- | --- | --- | --- | --- | --- | --- | --- | --- | --- | --- | --- | --- | --- | --- | --- | --- | --- | --- | --- | --- | --- | --- | --- | --- | --- | --- | --- | --- | --- | --- | --- | --- | --- | --- | --- | --- | --- | --- | --- | --- | --- | --- | --- | --- | --- | --- | --- | --- | --- | --- | --- | --- | --- | --- | --- | --- | --- | --- | --- | --- | --- | --- | --- | --- | --- | --- | --- | --- | --- | --- | --- | --- | --- | --- | --- | --- | --- | --- | --- | --- | --- | --- | --- | --- | --- | --- | --- | --- | --- | --- | --- | --- | --- | --- | --- | --- | --- | --- | --- | --- | --- | --- | --- | --- | --- | --- | --- | --- | --- | --- | --- | --- | --- | --- | --- | --- | --- | --- | --- | --- | --- | --- | --- | --- | --- | --- | --- | --- | --- | --- | --- | --- | --- | --- | --- | --- | --- | --- | --- | --- | --- | --- | --- | --- | --- | --- | --- | --- | --- | --- | --- | --- | --- | --- | --- | --- | --- | --- | --- | --- | --- | --- | --- | --- | --- | --- | --- | --- | --- | --- | --- | --- | --- | --- | --- | --- | --- | --- | --- | --- | --- | --- | --- | --- | --- | --- | --- | --- | --- | --- | --- | --- | --- | --- | --- | --- | --- | --- | --- | --- | --- | --- | --- | --- | --- | --- | --- | --- | --- | --- | --- | --- | --- | --- | --- | --- | --- | --- | --- | --- | --- | --- | --- | --- | --- | --- | --- | --- | --- | --- | --- | --- | --- | --- | --- | --- | --- | --- | --- | --- | --- | --- | --- | --- | --- | --- | --- | --- | --- | --- | --- | --- | --- | --- | --- | --- | --- | --- | --- | --- | --- | --- | --- | --- | --- | --- | --- | --- | --- | --- | --- | --- | --- | --- | --- | --- | --- | --- | --- | --- | --- | --- | --- | --- | --- | --- | --- | --- | --- | --- | --- | --- | --- | --- | --- | --- | --- | --- | --- | --- | --- | --- | --- | --- | --- | --- | --- | --- | --- | --- | --- | --- | --- | --- | --- | --- | --- | --- | --- | --- | --- | --- | --- | --- | --- | --- | --- | --- | --- | --- | --- | --- | --- | --- | | **Individual Motif View**  Individual Motif View  | Motif id | Motif name | Cluster | Collection | Width | IC | Number of sites | Consensus | Consensus (Rev) | Logo | Logo (Rev) | | --- | --- | --- | --- | --- | --- | --- | --- | --- | --- | --- | | coexpression\_cluster3\_5\_m27\_cluster3\_5\_motif27\_m | cluster3\_5\_motif27\_m | cluster\_8 | coexpression\_cluster3\_5 | 7 | 7.71 | 360 | **TAAGACA** | **TGTCTTA** |  |  | | coexpression\_cluster3\_5\_m7\_cluster3\_5\_motif7\_r | cluster3\_5\_motif7\_r | cluster\_1 | coexpression\_cluster3\_5 | 15 | 9.10 | 1061 | **++WWTTTGTCTTWWW** | **wwwAAgACAAAww++** |  |  | | coexpression\_cluster3\_5\_m29\_cluster3\_5\_motif29\_m | cluster3\_5\_motif29\_m | cluster\_2 | coexpression\_cluster3\_5 | 22 | 7.91 | 208 | **+++++++TGTGTGYR+++++++** | **+++++++YrCACACa+++++++** |  |  | | coexpression\_cluster3\_5\_m13\_cluster3\_5\_motif13\_r | cluster3\_5\_motif13\_r | cluster\_2 | coexpression\_cluster3\_5 | 22 | 8.70 | 943 | **++++++wwATGTGCATww++++** | **++++WWATGCACATWW++++++** |  |  | | coexpression\_cluster3\_5\_m23\_cluster3\_5\_motif23\_m | cluster3\_5\_motif23\_m | cluster\_1 | coexpression\_cluster3\_5 | 15 | 12.47 | 307 | **+TTATTTATTTTATT** | **AATAAAATAAATAA+** |  |  | | coexpression\_cluster3\_5\_m19\_cluster3\_5\_motif19\_r | cluster3\_5\_motif19\_r | cluster\_2 | coexpression\_cluster3\_5 | 22 | 9.73 | 291 | **++wwwtrTGTGTGCAwww++++** | **++++WWWTGCACACAYAWWW++** |  |  | | coexpression\_cluster3\_5\_m10\_cluster3\_5\_motif10\_r | cluster3\_5\_motif10\_r | cluster\_1 | coexpression\_cluster3\_5 | 15 | 9.46 | 2214 | **+WWAAAARAMAATWW** | **wwATTkTyTTTTww+** |  |  | | coexpression\_cluster3\_5\_m12\_cluster3\_5\_motif12\_r | cluster3\_5\_motif12\_r | cluster\_1 | coexpression\_cluster3\_5 | 15 | 9.08 | 812 | **++wwwTAgACAAAww** | **WWTTTGTCTAWWW++** |  |  | | coexpression\_cluster3\_5\_m8\_cluster3\_5\_motif8\_r | cluster3\_5\_motif8\_r | cluster\_1 | coexpression\_cluster3\_5 | 15 | 9.23 | 1154 | **+waAAAAgACAwwww** | **WWWWTGTCTTTTTW+** |  |  | | coexpression\_cluster3\_5\_m33\_cluster3\_5\_motif33\_m | cluster3\_5\_motif33\_m | cluster\_7 | coexpression\_cluster3\_5 | 13 | 10.63 | 92 | **AAAyCGwkrRAAA** | **TTTYYMWCGRTTT** |  |  | | coexpression\_cluster3\_5\_m22\_cluster3\_5\_motif22\_r | cluster3\_5\_motif22\_r | cluster\_4 | coexpression\_cluster3\_5 | 11 | 9.44 | 512 | **WWWTCTATAWW** | **wwTATAGAwww** |  |  | | coexpression\_cluster3\_5\_m16\_cluster3\_5\_motif16\_r | cluster3\_5\_motif16\_r | cluster\_1 | coexpression\_cluster3\_5 | 15 | 9.35 | 787 | **+WAAAAAGACAARWW** | **wwyTTGTcTTTTtw+** |  |  | | coexpression\_cluster3\_5\_m25\_cluster3\_5\_motif25\_m | cluster3\_5\_motif25\_m | cluster\_2 | coexpression\_cluster3\_5 | 22 | 10.53 | 863 | **+++TATWTRYATRTATRY++++** | **++++ryAtAyAtRyAwATA+++** |  |  | | coexpression\_cluster3\_5\_m18\_cluster3\_5\_motif18\_r | cluster3\_5\_motif18\_r | cluster\_6 | coexpression\_cluster3\_5 | 10 | 9.15 | 186 | **wwAAGTGTdw** | **WHACACTTWW** |  |  | | coexpression\_cluster3\_5\_m20\_cluster3\_5\_motif20\_r | cluster3\_5\_motif20\_r | cluster\_4 | coexpression\_cluster3\_5 | 11 | 9.34 | 113 | **wtATCTACAw+** | **+WTGTAGATAW** |  |  | | coexpression\_cluster3\_5\_m2\_cluster3\_5\_motif2\_r | cluster3\_5\_motif2\_r | cluster\_1 | coexpression\_cluster3\_5 | 15 | 9.09 | 2162 | **+wwTATAkAcAAAww** | **WWTTTGTMTATAWW+** |  |  | | coexpression\_cluster3\_5\_m15\_cluster3\_5\_motif15\_r | cluster3\_5\_motif15\_r | cluster\_3 | coexpression\_cluster3\_5 | 10 | 9.20 | 490 | **wwAGAAATaw** | **WTATTTCTWW** |  |  | | coexpression\_cluster3\_5\_m28\_cluster3\_5\_motif28\_m | cluster3\_5\_motif28\_m | cluster\_1 | coexpression\_cluster3\_5 | 15 | 12.50 | 412 | **AAAAAAAmrrAAAAA** | **TTTTTYYKTTTTTTT** |  |  | | coexpression\_cluster3\_5\_m26\_cluster3\_5\_motif26\_m | cluster3\_5\_motif26\_m | cluster\_1 | coexpression\_cluster3\_5 | 15 | 15.26 | 138 | **AAAAAAAAATAATAA** | **TTATTATTTTTTTTT** |  |  | | coexpression\_cluster3\_5\_m5\_cluster3\_5\_motif5\_r | cluster3\_5\_motif5\_r | cluster\_1 | coexpression\_cluster3\_5 | 15 | 8.72 | 1237 | **+WWWTGTCTTTTTW+** | **+waAAAAgACAwww+** |  |  | | coexpression\_cluster3\_5\_m11\_cluster3\_5\_motif11\_r | cluster3\_5\_motif11\_r | cluster\_1 | coexpression\_cluster3\_5 | 15 | 9.22 | 2343 | **+wwAATArAmAAAww** | **WWTTTKTYTATTWW+** |  |  | | coexpression\_cluster3\_5\_m24\_cluster3\_5\_motif24\_m | cluster3\_5\_motif24\_m | cluster\_2 | coexpression\_cluster3\_5 | 22 | 18.43 | 150 | **+++TATATATATATATATA+++** | **+++TATATATATATATATA+++** |  |  | | coexpression\_cluster3\_5\_m32\_cluster3\_5\_motif32\_m | cluster3\_5\_motif32\_m | cluster\_1 | coexpression\_cluster3\_5 | 15 | 11.29 | 114 | **+TTTYCCTTWTCT++** | **++AGAwAAGGRAAA+** |  |  | | coexpression\_cluster3\_5\_m31\_cluster3\_5\_motif31\_m | cluster3\_5\_motif31\_m | cluster\_2 | coexpression\_cluster3\_5 | 22 | 10.83 | 132 | **CYBKDYGTGTGTGCAAAWWSAW** | **wtswwttTGCACACACrhmvrg** |  |  | | coexpression\_cluster3\_5\_m9\_cluster3\_5\_motif9\_r | cluster3\_5\_motif9\_r | cluster\_2 | coexpression\_cluster3\_5 | 22 | 10.35 | 3243 | **+++waTATrTaTATAtw+++++** | **+++++WATATATAYATATW+++** |  |  | | coexpression\_cluster3\_5\_m6\_cluster3\_5\_motif6\_r | cluster3\_5\_motif6\_r | cluster\_1 | coexpression\_cluster3\_5 | 15 | 8.74 | 1043 | **+WWWWTAGACAAAWW** | **wwTTTGTcTAwwww+** |  |  | | coexpression\_cluster3\_5\_m30\_cluster3\_5\_motif30\_m | cluster3\_5\_motif30\_m | cluster\_5 | coexpression\_cluster3\_5 | 13 | 13.83 | 147 | **AAATAATAATAAT** | **ATTATTATTATTT** |  |  | | coexpression\_cluster3\_5\_m17\_cluster3\_5\_motif17\_r | cluster3\_5\_motif17\_r | cluster\_9 | coexpression\_cluster3\_5 | 10 | 9.08 | 36 | **raAACGCCww** | **WWGGCGTTTY** |  |  | | coexpression\_cluster3\_5\_m34\_cluster3\_5\_motif34\_m | cluster3\_5\_motif34\_m | cluster\_2 | coexpression\_cluster3\_5 | 22 | 9.42 | 183 | **++++ATGTGTGTGCA+++++++** | **+++++++TGCACACACAT++++** |  |  | | coexpression\_cluster3\_5\_m4\_cluster3\_5\_motif4\_r | cluster3\_5\_motif4\_r | cluster\_2 | coexpression\_cluster3\_5 | 22 | 9.64 | 576 | **++WWWTGTGTGTGCATWW++++** | **++++wwATGCACACAcawww++** |  |  | | coexpression\_cluster3\_5\_m14\_cluster3\_5\_motif14\_r | cluster3\_5\_motif14\_r | cluster\_2 | coexpression\_cluster3\_5 | 22 | 8.65 | 854 | **+++WWTGTGTGTATW+++++++** | **+++++++waTACACACAww+++** |  |  | | coexpression\_cluster3\_5\_m3\_cluster3\_5\_motif3\_r | cluster3\_5\_motif3\_r | cluster\_1 | coexpression\_cluster3\_5 | 15 | 9.60 | 2821 | **+WWTATDTYTTTTWW** | **wwAAAArAhATAww+** |  |  | | coexpression\_cluster3\_5\_m1\_cluster3\_5\_motif1\_r | cluster3\_5\_motif1\_r | cluster\_1 | coexpression\_cluster3\_5 | 15 | 9.75 | 3514 | **+waAAAArAmAAAww** | **WWTTTKTYTTTTTW+** |  |  | | coexpression\_cluster3\_5\_m21\_cluster3\_5\_motif21\_r | cluster3\_5\_motif21\_r | cluster\_3 | coexpression\_cluster3\_5 | 10 | 9.07 | 259 | **AWAGAATTWW** | **wwAATTCTwt** |  |  |  - Click on the column names to change the order of the data. - Write the name of one cluster, collection or a pattern in the *Search* window. | | **Heatmap View**  Heatmap View Distance table | PDF | |

**Additional Files**

## Exported files

| File | Description |
| --- | --- |
| **Pairwise comparison** | This table shows the pairwise comparison between all the input motifs using different metrics. This is the *compare-matrices* result. |
| **Matrix description** | This table shows information of each input motif. |
| **Cluster-wise Motif Files [zip]** | One TRANSFAC file is generated for each cluster including all the input motifs grouped in the corresponding cluster. |
| **Root motifs** | A file with the root matrix of each cluster. |
| **Motif collections per cluster** | A tab separated file containing the counts of motif of each collection on each cluster. |
| **Clusters** | A tab separated file containing the clusters and their correspondng motifs. |
| **Central motifs IDs** | A tab separated file containing the central motif, that one who is the most similar to all the others motif in the same cluster. |
| **Central motifs (in TRANSFAC format)** | A transfac file containing the central motif, that one who is the most similar to all the others motif within the same cluster. |
| **Input motifs** | The input motifs analyzed with matrix-clustering. |

- **References**
  1. Castro-Mondragon JA et al.  RSAT matrix-clustering: dynamic exploration and redundancy reduction of transcription factor binding motif collections. doi: 10.1093/nar/gkx314; Nucleic Acids Research (2017)
  2. **Last RSAT release:** Nguyen NTT et al. RSAT 2018: regulatory sequence analysis tools 20th anniversary. Nucleic Acids Research (2018).
- **Additional Information**
  - Jacques van Helden course
- **Contact**
  - Jaime Castro-Mondragon - **j.a.c.mondragon@ncmm.uio.no**
  - Morgane Thomas-Chollier - **mthomas@biologie.ens.fr**
  - Jacques van Helden - **Jacques.van-Helden@univ-amu.fr**
