# Supplementary material for: Transcriptome analysis reveals a de novo DNA element that may interact with chromatin-associated proteins in Plasmodium berghei during erythrocytic development
Source: Sci Rep. 2025 May 28;15:18621. doi: 10.1038/s41598-025-03586-4 (PMC12120095; doi:10.1038/s41598-025-03586-4)
Supplement: Supplementary file 2 — Supplementary Information 2. [file 41598_2025_3586_MOESM2_ESM.zip › Supplementary File S2/Clustering/matrix-clustering_SUMMARY_cluster3_7.html]

matrix-clustering coexpression\_cluster3\_7


# RSAT - matrix-clustering result

### Analysis: coexpression\_cluster3\_7 (08/09/2023 16:02)

### Command

```
matrix-clustering  -v 1 -max_matrices 300 -matrix coexpression_cluster3_7 $RSAT/public_html/tmp/www-data/2023/09/08/matrix-clustering_2023-09-08.160225_1025zP/matrix-clustering_query_matrices.transfac transfac -hclust_method average -calc sum -title coexpression_cluster3_7 -metric_build_tree Ncor -lth w 5 -lth cor 0.6 -lth Ncor 0.4 -quick -label_in_tree name -return json,heatmap -o $RSAT/public_html/tmp/www-data/2023/09/08/matrix-clustering_2023-09-08.160225_1025zP/matrix-clustering
```


## Results Summary

|  |  |  |  |  |  |  |  |  |  |  |
| --- | --- | --- | --- | --- | --- | --- | --- | --- | --- | --- |
| Nb Input motifs | Nb Input collections | Nb Clusters Found | Download   Root motifs | Complete results [zip] | Parameters used  |  |  |  | | --- | --- | --- | | Linkage method | Similarity metric | Thresholds to partition the tree | | | |
| 30 | 1 | 9 | **Download** | Download | |  |  |  | | --- | --- | --- | | average | Ncor | **Ncor** = 0.4 **cor** = 0.6 | |

|  |  |
| --- | --- |
|  | **Logo Forest (dynamic browsing)**  **Logo Forest (rapid overview - low image quality)**  cluster\_1  cluster\_2  cluster\_3  cluster\_4  cluster\_5  cluster\_6  cluster\_7  cluster\_8  cluster\_9 |
|  | |  |  |  |  |  |  |  |  |  |  | | --- | --- | --- | --- | --- | --- | --- | --- | --- | --- | | **Clusters Summary**  Clusters Summary Table  | Root Motif | Root Motif (Reverse) | Cluster ID | # Motifs | Motif\_Name::Collection | Number of Motifs by Collection | Root Motif (transfac Format) | | --- | --- | --- | --- | --- | --- | --- | |  | | --- | |  | | cluster\_1 | 9 | cluster3\_7\_motif19\_m::coexpression\_cluster3\_7:: cluster3\_7\_motif20\_m::coexpression\_cluster3\_7:: cluster3\_7\_motif5\_r::coexpression\_cluster3\_7:: cluster3\_7\_motif6\_r::coexpression\_cluster3\_7:: cluster3\_7\_motif1\_r::coexpression\_cluster3\_7:: cluster3\_7\_motif2\_r::coexpression\_cluster3\_7:: cluster3\_7\_motif7\_r::coexpression\_cluster3\_7:: cluster3\_7\_motif12\_r::coexpression\_cluster3\_7:: cluster3\_7\_motif3\_r::coexpression\_cluster3\_7:: | coexpression\_cluster3\_7::9\_motifs | Show matrix |
| cluster\_2 | 3 | cluster3\_7\_motif11\_r::coexpression\_cluster3\_7:: cluster3\_7\_motif23\_m::coexpression\_cluster3\_7:: cluster3\_7\_motif27\_m::coexpression\_cluster3\_7:: | coexpression\_cluster3\_7::3\_motifs | Show matrix | | |
| cluster\_3 | 4 | cluster3\_7\_motif18\_m::coexpression\_cluster3\_7:: cluster3\_7\_motif21\_m::coexpression\_cluster3\_7:: cluster3\_7\_motif4\_r::coexpression\_cluster3\_7:: cluster3\_7\_motif9\_r::coexpression\_cluster3\_7:: | coexpression\_cluster3\_7::4\_motifs | Show matrix | | |
| cluster\_4 | 5 | cluster3\_7\_motif26\_m::coexpression\_cluster3\_7:: cluster3\_7\_motif17\_m::coexpression\_cluster3\_7:: cluster3\_7\_motif25\_m::coexpression\_cluster3\_7:: cluster3\_7\_motif15\_r::coexpression\_cluster3\_7:: cluster3\_7\_motif8\_r::coexpression\_cluster3\_7:: | coexpression\_cluster3\_7::5\_motifs | Show matrix | | |
| cluster\_5 | 5 | cluster3\_7\_motif28\_m::coexpression\_cluster3\_7:: cluster3\_7\_motif29\_m::coexpression\_cluster3\_7:: cluster3\_7\_motif24\_m::coexpression\_cluster3\_7:: cluster3\_7\_motif10\_r::coexpression\_cluster3\_7:: cluster3\_7\_motif22\_m::coexpression\_cluster3\_7:: | coexpression\_cluster3\_7::5\_motifs | Show matrix | | |
| cluster\_6 | 1 | cluster3\_7\_motif14\_r::coexpression\_cluster3\_7 | coexpression\_cluster3\_7 : 1 motif | Show matrix | | |
| cluster\_7 | 1 | cluster3\_7\_motif16\_r::coexpression\_cluster3\_7 | coexpression\_cluster3\_7 : 1 motif | Show matrix | | |
| cluster\_8 | 1 | cluster3\_7\_motif13\_r::coexpression\_cluster3\_7 | coexpression\_cluster3\_7 : 1 motif | Show matrix | | |
| cluster\_9 | 1 | cluster3\_7\_motif30\_m::coexpression\_cluster3\_7 | coexpression\_cluster3\_7 : 1 motif | Show matrix | | |

- Note: by default the table displays the first 50 clusters. Click on the *Show X entries* button to display more (or less) clusters.
- Click on the column names to change the order of the data.
- Use the *Search* window to find your motif (s) of interest.

| **Individual Cluster View**  Individual Cluster View **Show All**  **Hide All**  **cluster\_1**  **cluster\_2**  **cluster\_3**  **cluster\_4**  **cluster\_5**  **cluster\_6**  **cluster\_7**  **cluster\_8**  **cluster\_9**  **Display Logo Trees**   **cluster\_1**    **cluster\_2**    **cluster\_3**    **cluster\_4**    **cluster\_5**    **cluster\_6**    **cluster\_7**    **cluster\_8**    **cluster\_9**  **Display Branch-Motifs** **cluster\_1**   | Branch Motifs | | | | | | --- | --- | --- | --- | --- | | Node | Consensus | Logo | Logo (Reverse) | Collections | Matrix (Transfac format) | IC | Nb sites | | Node 1 | **wwAamTAGCTAtTTww-** |  |  | coexpression\_cluster3\_7::2\_motifs | PSSM | 9.27 | 579 | | --- | --- | --- | --- | --- | --- | --- | --- | | Node 2 | **-wwttTAGCTAktTww-** |  |  | coexpression\_cluster3\_7::2\_motifs | PSSM | 9.22 | 503 | | Node 3 | **wwAamTAGCTAktTwww** |  |  | coexpression\_cluster3\_7::3\_motifs | PSSM | 9.41 | 772 | | Node 4 | **-wwwtTAGCTAktTww-** |  |  | coexpression\_cluster3\_7::3\_motifs | PSSM | 9.26 | 732 | | Node 5 | **----wtArCTAGCyAw-** |  |  | coexpression\_cluster3\_7::2\_motifs | PSSM | 8.74 | 177 | | Node 6 | **wwwwhTAGCTAktTwww** |  |  | coexpression\_cluster3\_7::6\_motifs | PSSM | 9.26 | 1504 | | Node 7 | **wwwwhTAGCTAktTwww** |  |  | coexpression\_cluster3\_7::7\_motifs | PSSM | 9.13 | 1628 | | Node 8 | **wwwwhTAGCTAkyTwww** |  |  | coexpression\_cluster3\_7::9\_motifs | PSSM | 8.86 | 1805 |  **cluster\_2**   | Branch Motifs | | | | | | --- | --- | --- | --- | --- | | Node | Consensus | Logo | Logo (Reverse) | Collections | Matrix (Transfac format) | IC | Nb sites | | Node 1 | **--TTATkAACAaktmAT** |  |  | coexpression\_cluster3\_7::2\_motifs | PSSM | 14.41 | 91 | | --- | --- | --- | --- | --- | --- | --- | --- | | Node 2 | **wwwTATGAACAwwtmAT** |  |  | coexpression\_cluster3\_7::3\_motifs | PSSM | 12.09 | 584 |  **cluster\_3**   | Branch Motifs | | | | | | --- | --- | --- | --- | --- | | Node | Consensus | Logo | Logo (Reverse) | Collections | Matrix (Transfac format) | IC | Nb sites | | Node 1 | **TAYAYAYAyATATATA** |  |  | coexpression\_cluster3\_7::2\_motifs | PSSM | 13.58 | 392 | | --- | --- | --- | --- | --- | --- | --- | --- | | Node 2 | **-wwATAgACAwwww--** |  |  | coexpression\_cluster3\_7::2\_motifs | PSSM | 8.50 | 846 | | Node 3 | **TawATAkACAwawaTA** |  |  | coexpression\_cluster3\_7::4\_motifs | PSSM | 11.03 | 1238 |  **cluster\_4**   | Branch Motifs | | | | | | --- | --- | --- | --- | --- | | Node | Consensus | Logo | Logo (Reverse) | Collections | Matrix (Transfac format) | IC | Nb sites | | Node 1 | **--AAAAAAAArAwAAAA** |  |  | coexpression\_cluster3\_7::2\_motifs | PSSM | 12.73 | 332 | | --- | --- | --- | --- | --- | --- | --- | --- | | Node 2 | **-wwaAAAAAArww----** |  |  | coexpression\_cluster3\_7::2\_motifs | PSSM | 8.14 | 5987 | | Node 3 | **-wwaAAAAAArwwAAAA** |  |  | coexpression\_cluster3\_7::4\_motifs | PSSM | 11.87 | 6318 | | Node 4 | **AawaAAAAAArwwAAAA** |  |  | coexpression\_cluster3\_7::5\_motifs | PSSM | 12.47 | 6402 |  **cluster\_5**   | Branch Motifs | | | | | | --- | --- | --- | --- | --- | | Node | Consensus | Logo | Logo (Reverse) | Collections | Matrix (Transfac format) | IC | Nb sites | | Node 1 | **----AtdTGTGTGyat------** |  |  | coexpression\_cluster3\_7::2\_motifs | PSSM | 8.69 | 501 | | --- | --- | --- | --- | --- | --- | --- | --- | | Node 2 | **cyykdyGTGTGTGYraawwgaw** |  |  | coexpression\_cluster3\_7::2\_motifs | PSSM | 10.47 | 108 | | Node 3 | **----AtdTGTGTGyat------** |  |  | coexpression\_cluster3\_7::3\_motifs | PSSM | 8.72 | 591 | | Node 4 | **cyykatdTGTGTGyawawwgaw** |  |  | coexpression\_cluster3\_7::5\_motifs | PSSM | 8.94 | 699 |  **cluster\_6**   | Branch Motifs | | | | | | --- | --- | --- | --- | --- | | Node | Consensus | Logo | Logo (Reverse) | Collections | Matrix (Transfac format) | IC | Nb sites | | Singleton | **wwTACAAcTww** |  |  | coexpression\_cluster3\_7 : 1 motif | PSSM | 7.40 | 626 | | --- | --- | --- | --- | --- | --- | --- | --- |  **cluster\_7**   | Branch Motifs | | | | | | --- | --- | --- | --- | --- | | Node | Consensus | Logo | Logo (Reverse) | Collections | Matrix (Transfac format) | IC | Nb sites | | Singleton | **wwACAAGAww** |  |  | coexpression\_cluster3\_7 : 1 motif | PSSM | 9.37 | 59 | | --- | --- | --- | --- | --- | --- | --- | --- |  **cluster\_8**   | Branch Motifs | | | | | | --- | --- | --- | --- | --- | | Node | Consensus | Logo | Logo (Reverse) | Collections | Matrix (Transfac format) | IC | Nb sites | | Singleton | **wwACATTGhw** |  |  | coexpression\_cluster3\_7 : 1 motif | PSSM | 8.95 | 103 | | --- | --- | --- | --- | --- | --- | --- | --- |  **cluster\_9**   | Branch Motifs | | | | | | --- | --- | --- | --- | --- | | Node | Consensus | Logo | Logo (Reverse) | Collections | Matrix (Transfac format) | IC | Nb sites | | Singleton | **CCCCSC** |  |  | coexpression\_cluster3\_7 : 1 motif | PSSM | 7.05 | 19 | | --- | --- | --- | --- | --- | --- | --- | --- |   - **Each cluster has a different color.** - **Click on the upper buttons to display separately the information of each cluster.** - **Click on the circles at each tree to display its corresponding merged motifs.**   Definitions  - **Logo tree.**  This results shows the hierarchical tree with its logo alignment of one cluster. The logos are shown in Forward (left logo) and Reverse (right logo) orientation. - **Branch-motifs.**  On the trees are displayed the branch number that is the number in which the motifs were incorporated in the tree. The Branch-Motifs table shows the logo in both orientations and the link to the file in TRANSFAC format with the branch-motif. |
|  | |  |  |  |  |  |  |  |  |  |  |  |  |  |  |  |  |  |  |  |  |  |  |  |  |  |  |  |  |  |  |  |  |  |  |  |  |  |  |  |  |  |  |  |  |  |  |  |  |  |  |  |  |  |  |  |  |  |  |  |  |  |  |  |  |  |  |  |  |  |  |  |  |  |  |  |  |  |  |  |  |  |  |  |  |  |  |  |  |  |  |  |  |  |  |  |  |  |  |  |  |  |  |  |  |  |  |  |  |  |  |  |  |  |  |  |  |  |  |  |  |  |  |  |  |  |  |  |  |  |  |  |  |  |  |  |  |  |  |  |  |  |  |  |  |  |  |  |  |  |  |  |  |  |  |  |  |  |  |  |  |  |  |  |  |  |  |  |  |  |  |  |  |  |  |  |  |  |  |  |  |  |  |  |  |  |  |  |  |  |  |  |  |  |  |  |  |  |  |  |  |  |  |  |  |  |  |  |  |  |  |  |  |  |  |  |  |  |  |  |  |  |  |  |  |  |  |  |  |  |  |  |  |  |  |  |  |  |  |  |  |  |  |  |  |  |  |  |  |  |  |  |  |  |  |  |  |  |  |  |  |  |  |  |  |  |  |  |  |  |  |  |  |  |  |  |  |  |  |  |  |  |  |  |  |  |  |  |  |  |  |  |  |  |  |  |  |  |  |  |  |  |  |  |  |  |  |  |  |  |  |  |  |  |  |  |  |  |  |  |  |  |  |  |  |  |  |  |  |  |  |  |  |  |  |  |  |  |  |  |  |  |  | | --- | --- | --- | --- | --- | --- | --- | --- | --- | --- | --- | --- | --- | --- | --- | --- | --- | --- | --- | --- | --- | --- | --- | --- | --- | --- | --- | --- | --- | --- | --- | --- | --- | --- | --- | --- | --- | --- | --- | --- | --- | --- | --- | --- | --- | --- | --- | --- | --- | --- | --- | --- | --- | --- | --- | --- | --- | --- | --- | --- | --- | --- | --- | --- | --- | --- | --- | --- | --- | --- | --- | --- | --- | --- | --- | --- | --- | --- | --- | --- | --- | --- | --- | --- | --- | --- | --- | --- | --- | --- | --- | --- | --- | --- | --- | --- | --- | --- | --- | --- | --- | --- | --- | --- | --- | --- | --- | --- | --- | --- | --- | --- | --- | --- | --- | --- | --- | --- | --- | --- | --- | --- | --- | --- | --- | --- | --- | --- | --- | --- | --- | --- | --- | --- | --- | --- | --- | --- | --- | --- | --- | --- | --- | --- | --- | --- | --- | --- | --- | --- | --- | --- | --- | --- | --- | --- | --- | --- | --- | --- | --- | --- | --- | --- | --- | --- | --- | --- | --- | --- | --- | --- | --- | --- | --- | --- | --- | --- | --- | --- | --- | --- | --- | --- | --- | --- | --- | --- | --- | --- | --- | --- | --- | --- | --- | --- | --- | --- | --- | --- | --- | --- | --- | --- | --- | --- | --- | --- | --- | --- | --- | --- | --- | --- | --- | --- | --- | --- | --- | --- | --- | --- | --- | --- | --- | --- | --- | --- | --- | --- | --- | --- | --- | --- | --- | --- | --- | --- | --- | --- | --- | --- | --- | --- | --- | --- | --- | --- | --- | --- | --- | --- | --- | --- | --- | --- | --- | --- | --- | --- | --- | --- | --- | --- | --- | --- | --- | --- | --- | --- | --- | --- | --- | --- | --- | --- | --- | --- | --- | --- | --- | --- | --- | --- | --- | --- | --- | --- | --- | --- | --- | --- | --- | --- | --- | --- | --- | --- | --- | --- | --- | --- | --- | --- | --- | --- | --- | --- | --- | --- | --- | --- | --- | --- | --- | --- | --- | --- | --- | --- | --- | --- | --- | --- | --- | --- | --- | --- | --- | --- | --- | --- | --- | --- | --- | --- | --- | --- | --- | --- | --- | --- | | **Individual Motif View**  Individual Motif View  | Motif id | Motif name | Cluster | Collection | Width | IC | Number of sites | Consensus | Consensus (Rev) | Logo | Logo (Rev) | | --- | --- | --- | --- | --- | --- | --- | --- | --- | --- | --- | | coexpression\_cluster3\_7\_m3\_cluster3\_7\_motif3\_r | cluster3\_7\_motif3\_r | cluster\_1 | coexpression\_cluster3\_7 | 17 | 9.21 | 299 | **+WWTTTAGCTAKTTWW+** | **+wwAamTAGCTAAAww+** |  |  | | coexpression\_cluster3\_7\_m12\_cluster3\_7\_motif12\_r | cluster3\_7\_motif12\_r | cluster\_1 | coexpression\_cluster3\_7 | 17 | 9.85 | 204 | **+WWWMTAGCTAKTTWW+** | **+wwAamTAGCTAkwww+** |  |  | | coexpression\_cluster3\_7\_m27\_cluster3\_7\_motif27\_m | cluster3\_7\_motif27\_m | cluster\_2 | coexpression\_cluster3\_7 | 17 | 13.59 | 47 | **++WTATKAACAaktmA+** | **+TKAMTTGTTMATAW++** |  |  | | coexpression\_cluster3\_7\_m30\_cluster3\_7\_motif30\_m | cluster3\_7\_motif30\_m | cluster\_9 | coexpression\_cluster3\_7 | 6 | 7.05 | 19 | **MCCCCC** | **GGGGGK** |  |  | | coexpression\_cluster3\_7\_m28\_cluster3\_7\_motif28\_m | cluster3\_7\_motif28\_m | cluster\_5 | coexpression\_cluster3\_7 | 22 | 10.97 | 88 | **CYBKDYGTGTGTGCAAAWWSAW** | **wtswwttTGCACACACrhmvrg** |  |  | | coexpression\_cluster3\_7\_m9\_cluster3\_7\_motif9\_r | cluster3\_7\_motif9\_r | cluster\_3 | coexpression\_cluster3\_7 | 16 | 8.92 | 417 | **+wwATAkACATww+++** | **+++WWATGTMTATWW+** |  |  | | coexpression\_cluster3\_7\_m21\_cluster3\_7\_motif21\_m | cluster3\_7\_motif21\_m | cluster\_3 | coexpression\_cluster3\_7 | 16 | 12.02 | 317 | **+RYAYAyAyATATATA** | **TATATATRTRTRTRY+** |  |  | | coexpression\_cluster3\_7\_m10\_cluster3\_7\_motif10\_r | cluster3\_7\_motif10\_r | cluster\_5 | coexpression\_cluster3\_7 | 22 | 7.77 | 403 | **+++++TWTGTGTGYAT++++++** | **++++++atRCACACAwa+++++** |  |  | | coexpression\_cluster3\_7\_m17\_cluster3\_7\_motif17\_m | cluster3\_7\_motif17\_m | cluster\_4 | coexpression\_cluster3\_7 | 17 | 15.84 | 132 | **++AAAAAAAWAATAAAA** | **TTTTATTWTTTTTTT++** |  |  | | coexpression\_cluster3\_7\_m2\_cluster3\_7\_motif2\_r | cluster3\_7\_motif2\_r | cluster\_1 | coexpression\_cluster3\_7 | 17 | 9.39 | 331 | **WWAWMTAGCTATTTWW+** | **+wwAAATAGCTAkwTww** |  |  | | coexpression\_cluster3\_7\_m14\_cluster3\_7\_motif14\_r | cluster3\_7\_motif14\_r | cluster\_6 | coexpression\_cluster3\_7 | 11 | 7.40 | 626 | **wwTACAAcTww** | **WWAGTTGTAWW** |  |  | | coexpression\_cluster3\_7\_m19\_cluster3\_7\_motif19\_m | cluster3\_7\_motif19\_m | cluster\_1 | coexpression\_cluster3\_7 | 17 | 8.46 | 91 | **++++wTAGCTAGCTAw+** | **+WTAGCTAGCTAW++++** |  |  | | coexpression\_cluster3\_7\_m18\_cluster3\_7\_motif18\_m | cluster3\_7\_motif18\_m | cluster\_3 | coexpression\_cluster3\_7 | 16 | 18.23 | 75 | **TATATATATATATATA** | **TATATATATATATATA** |  |  | | coexpression\_cluster3\_7\_m20\_cluster3\_7\_motif20\_m | cluster3\_7\_motif20\_m | cluster\_1 | coexpression\_cluster3\_7 | 17 | 9.57 | 86 | **+++++yArCTAGCyAw+** | **+WTRGCTAGYTR+++++** |  |  | | coexpression\_cluster3\_7\_m11\_cluster3\_7\_motif11\_r | cluster3\_7\_motif11\_r | cluster\_2 | coexpression\_cluster3\_7 | 17 | 9.03 | 493 | **WWTGTTCATAWWW++++** | **++++wwwTATGAACAww** |  |  | | coexpression\_cluster3\_7\_m26\_cluster3\_7\_motif26\_m | cluster3\_7\_motif26\_m | cluster\_4 | coexpression\_cluster3\_7 | 17 | 11.17 | 84 | **CTTTTTTTTCATT++++** | **++++AATGAAAAAAAAG** |  |  | | coexpression\_cluster3\_7\_m25\_cluster3\_7\_motif25\_m | cluster3\_7\_motif25\_m | cluster\_4 | coexpression\_cluster3\_7 | 17 | 13.15 | 200 | **++AAAAAAAArRRAAAA** | **TTTTYYYTTTTTTTT++** |  |  | | coexpression\_cluster3\_7\_m4\_cluster3\_7\_motif4\_r | cluster3\_7\_motif4\_r | cluster\_3 | coexpression\_cluster3\_7 | 16 | 8.99 | 429 | **+wwATAgACAAwww++** | **++WWWTTGTCTATWW+** |  |  | | coexpression\_cluster3\_7\_m16\_cluster3\_7\_motif16\_r | cluster3\_7\_motif16\_r | cluster\_7 | coexpression\_cluster3\_7 | 10 | 9.37 | 59 | **wwACAAGAww** | **WWTCTTGTWW** |  |  | | coexpression\_cluster3\_7\_m7\_cluster3\_7\_motif7\_r | cluster3\_7\_motif7\_r | cluster\_1 | coexpression\_cluster3\_7 | 17 | 9.45 | 229 | **++wwdTAGCTAkTTww+** | **+WWAAMTAGCTAHWW++** |  |  | | coexpression\_cluster3\_7\_m29\_cluster3\_7\_motif29\_m | cluster3\_7\_motif29\_m | cluster\_5 | coexpression\_cluster3\_7 | 22 | 12.48 | 21 | **WKMYDYGTGTGTGYGSAWWABA** | **TvtwwtsCRCACACACrhrkmw** |  |  | | coexpression\_cluster3\_7\_m8\_cluster3\_7\_motif8\_r | cluster3\_7\_motif8\_r | cluster\_4 | coexpression\_cluster3\_7 | 17 | 8.95 | 535 | **+wwAAgACAAAww++++** | **++++WWTTTGTCTTWW+** |  |  | | coexpression\_cluster3\_7\_m13\_cluster3\_7\_motif13\_r | cluster3\_7\_motif13\_r | cluster\_8 | coexpression\_cluster3\_7 | 10 | 8.95 | 103 | **wwACATTGhw** | **WDCAATGTWW** |  |  | | coexpression\_cluster3\_7\_m6\_cluster3\_7\_motif6\_r | cluster3\_7\_motif6\_r | cluster\_1 | coexpression\_cluster3\_7 | 17 | 10.00 | 193 | **wwAamTAGCTAgyTwww** | **WWWARCTAGCTAKTTWW** |  |  | | coexpression\_cluster3\_7\_m1\_cluster3\_7\_motif1\_r | cluster3\_7\_motif1\_r | cluster\_1 | coexpression\_cluster3\_7 | 17 | 9.70 | 248 | **WWAAMTAGCTAKTTWW+** | **+wwAamTAGCTAktTww** |  |  | | coexpression\_cluster3\_7\_m15\_cluster3\_7\_motif15\_r | cluster3\_7\_motif15\_r | cluster\_4 | coexpression\_cluster3\_7 | 17 | 8.24 | 5452 | **++waAAAAAArww++++** | **++++WWYTTTTTTTW++** |  |  | | coexpression\_cluster3\_7\_m5\_cluster3\_7\_motif5\_r | cluster3\_7\_motif5\_r | cluster\_1 | coexpression\_cluster3\_7 | 17 | 10.55 | 124 | **+WWTTTGGCTAKTTWW+** | **+wwAAmTAGCcAAAww+** |  |  | | coexpression\_cluster3\_7\_m23\_cluster3\_7\_motif23\_m | cluster3\_7\_motif23\_m | cluster\_2 | coexpression\_cluster3\_7 | 17 | 14.71 | 44 | **++TTATkAACAwGwmAT** | **ATKWCWTGTTMATAA++** |  |  | | coexpression\_cluster3\_7\_m24\_cluster3\_7\_motif24\_m | cluster3\_7\_motif24\_m | cluster\_5 | coexpression\_cluster3\_7 | 22 | 8.57 | 90 | **+++++++TGTGTGYA+++++++** | **+++++++TrCACACA+++++++** |  |  | | coexpression\_cluster3\_7\_m22\_cluster3\_7\_motif22\_m | cluster3\_7\_motif22\_m | cluster\_5 | coexpression\_cluster3\_7 | 22 | 10.11 | 98 | **++++ATGTGTGTGya+++++++** | **+++++++TRCACACACAT++++** |  |  |  - Click on the column names to change the order of the data. - Write the name of one cluster, collection or a pattern in the *Search* window. | | **Heatmap View**  Heatmap View Distance table | PDF | |

**Additional Files**

## Exported files

| File | Description |
| --- | --- |
| **Pairwise comparison** | This table shows the pairwise comparison between all the input motifs using different metrics. This is the *compare-matrices* result. |
| **Matrix description** | This table shows information of each input motif. |
| **Cluster-wise Motif Files [zip]** | One TRANSFAC file is generated for each cluster including all the input motifs grouped in the corresponding cluster. |
| **Root motifs** | A file with the root matrix of each cluster. |
| **Motif collections per cluster** | A tab separated file containing the counts of motif of each collection on each cluster. |
| **Clusters** | A tab separated file containing the clusters and their correspondng motifs. |
| **Central motifs IDs** | A tab separated file containing the central motif, that one who is the most similar to all the others motif in the same cluster. |
| **Central motifs (in TRANSFAC format)** | A transfac file containing the central motif, that one who is the most similar to all the others motif within the same cluster. |
| **Input motifs** | The input motifs analyzed with matrix-clustering. |

- **References**
  1. Castro-Mondragon JA et al.  RSAT matrix-clustering: dynamic exploration and redundancy reduction of transcription factor binding motif collections. doi: 10.1093/nar/gkx314; Nucleic Acids Research (2017)
  2. **Last RSAT release:** Nguyen NTT et al. RSAT 2018: regulatory sequence analysis tools 20th anniversary. Nucleic Acids Research (2018).
- **Additional Information**
  - Jacques van Helden course
- **Contact**
  - Jaime Castro-Mondragon - **j.a.c.mondragon@ncmm.uio.no**
  - Morgane Thomas-Chollier - **mthomas@biologie.ens.fr**
  - Jacques van Helden - **Jacques.van-Helden@univ-amu.fr**
