# Supplementary material for: Transcriptome analysis reveals a de novo DNA element that may interact with chromatin-associated proteins in Plasmodium berghei during erythrocytic development
Source: Sci Rep. 2025 May 28;15:18621. doi: 10.1038/s41598-025-03586-4 (PMC12120095; doi:10.1038/s41598-025-03586-4)
Supplement: Supplementary file 2 — Supplementary Information 2. [file 41598_2025_3586_MOESM2_ESM.zip › Supplementary File S2/Clustering/matrix-clustering_SUMMARY_cluster3_8.html]

matrix-clustering coexpression\_cluster3\_8


# RSAT - matrix-clustering result

### Analysis: coexpression\_cluster3\_8 (08/09/2023 16:09)

### Command

```
matrix-clustering  -v 1 -max_matrices 300 -matrix coexpression_cluster3_8 $RSAT/public_html/tmp/www-data/2023/09/08/matrix-clustering_2023-09-08.160939_KQzGMX/matrix-clustering_query_matrices.transfac transfac -hclust_method average -calc sum -title coexpression_cluster3_8 -metric_build_tree Ncor -lth w 5 -lth cor 0.6 -lth Ncor 0.4 -quick -label_in_tree name -return json,heatmap -o $RSAT/public_html/tmp/www-data/2023/09/08/matrix-clustering_2023-09-08.160939_KQzGMX/matrix-clustering
```


## Results Summary

|  |  |  |  |  |  |  |  |  |  |  |
| --- | --- | --- | --- | --- | --- | --- | --- | --- | --- | --- |
| Nb Input motifs | Nb Input collections | Nb Clusters Found | Download   Root motifs | Complete results [zip] | Parameters used  |  |  |  | | --- | --- | --- | | Linkage method | Similarity metric | Thresholds to partition the tree | | | |
| 30 | 1 | 8 | **Download** | Download | |  |  |  | | --- | --- | --- | | average | Ncor | **Ncor** = 0.4 **cor** = 0.6 | |

|  |  |
| --- | --- |
|  | **Logo Forest (dynamic browsing)**  **Logo Forest (rapid overview - low image quality)**  cluster\_1  cluster\_2  cluster\_3  cluster\_4  cluster\_5  cluster\_6  cluster\_7  cluster\_8 |
|  | |  |  |  |  |  |  |  |  |  |  | | --- | --- | --- | --- | --- | --- | --- | --- | --- | --- | | **Clusters Summary**  Clusters Summary Table  | Root Motif | Root Motif (Reverse) | Cluster ID | # Motifs | Motif\_Name::Collection | Number of Motifs by Collection | Root Motif (transfac Format) | | --- | --- | --- | --- | --- | --- | --- | |  | | --- | |  | | cluster\_1 | 13 | cluster3\_8\_motif7\_r::coexpression\_cluster3\_8:: cluster3\_8\_motif12\_r::coexpression\_cluster3\_8:: cluster3\_8\_motif13\_r::coexpression\_cluster3\_8:: cluster3\_8\_motif11\_r::coexpression\_cluster3\_8:: cluster3\_8\_motif1\_r::coexpression\_cluster3\_8:: cluster3\_8\_motif10\_r::coexpression\_cluster3\_8:: cluster3\_8\_motif19\_m::coexpression\_cluster3\_8:: cluster3\_8\_motif5\_r::coexpression\_cluster3\_8:: cluster3\_8\_motif20\_m::coexpression\_cluster3\_8:: cluster3\_8\_motif16\_r::coexpression\_cluster3\_8:: cluster3\_8\_motif4\_r::coexpression\_cluster3\_8:: cluster3\_8\_motif3\_r::coexpression\_cluster3\_8:: cluster3\_8\_motif9\_r::coexpression\_cluster3\_8:: | coexpression\_cluster3\_8::13\_motifs | Show matrix |
| cluster\_2 | 7 | cluster3\_8\_motif22\_m::coexpression\_cluster3\_8:: cluster3\_8\_motif25\_m::coexpression\_cluster3\_8:: cluster3\_8\_motif2\_r::coexpression\_cluster3\_8:: cluster3\_8\_motif6\_r::coexpression\_cluster3\_8:: cluster3\_8\_motif24\_m::coexpression\_cluster3\_8:: cluster3\_8\_motif14\_r::coexpression\_cluster3\_8:: cluster3\_8\_motif8\_r::coexpression\_cluster3\_8:: | coexpression\_cluster3\_8::7\_motifs | Show matrix | | |
| cluster\_3 | 4 | cluster3\_8\_motif18\_r::coexpression\_cluster3\_8:: cluster3\_8\_motif21\_m::coexpression\_cluster3\_8:: cluster3\_8\_motif27\_m::coexpression\_cluster3\_8:: cluster3\_8\_motif28\_m::coexpression\_cluster3\_8:: | coexpression\_cluster3\_8::4\_motifs | Show matrix | | |
| cluster\_4 | 2 | cluster3\_8\_motif26\_m::coexpression\_cluster3\_8:: cluster3\_8\_motif29\_m::coexpression\_cluster3\_8:: | coexpression\_cluster3\_8::2\_motifs | Show matrix | | |
| cluster\_5 | 1 | cluster3\_8\_motif23\_m::coexpression\_cluster3\_8 | coexpression\_cluster3\_8 : 1 motif | Show matrix | | |
| cluster\_6 | 1 | cluster3\_8\_motif15\_r::coexpression\_cluster3\_8 | coexpression\_cluster3\_8 : 1 motif | Show matrix | | |
| cluster\_7 | 1 | cluster3\_8\_motif17\_r::coexpression\_cluster3\_8 | coexpression\_cluster3\_8 : 1 motif | Show matrix | | |
| cluster\_8 | 1 | cluster3\_8\_motif30\_m::coexpression\_cluster3\_8 | coexpression\_cluster3\_8 : 1 motif | Show matrix | | |

- Note: by default the table displays the first 50 clusters. Click on the *Show X entries* button to display more (or less) clusters.
- Click on the column names to change the order of the data.
- Use the *Search* window to find your motif (s) of interest.

| **Individual Cluster View**  Individual Cluster View **Show All**  **Hide All**  **cluster\_1**  **cluster\_2**  **cluster\_3**  **cluster\_4**  **cluster\_5**  **cluster\_6**  **cluster\_7**  **cluster\_8**  **Display Logo Trees**   **cluster\_1**    **cluster\_2**    **cluster\_3**    **cluster\_4**    **cluster\_5**    **cluster\_6**    **cluster\_7**    **cluster\_8**  **Display Branch-Motifs** **cluster\_1**   | Branch Motifs | | | | | | --- | --- | --- | --- | --- | | Node | Consensus | Logo | Logo (Reverse) | Collections | Matrix (Transfac format) | IC | Nb sites | | Node 1 | **----wwTATAgAcAAAwww** |  |  | coexpression\_cluster3\_8::2\_motifs | PSSM | 9.40 | 1719 | | --- | --- | --- | --- | --- | --- | --- | --- | | Node 2 | **---awATACACACAtaw--** |  |  | coexpression\_cluster3\_8::2\_motifs | PSSM | 8.82 | 2139 | | Node 3 | **----wwTATAgAcAAAwww** |  |  | coexpression\_cluster3\_8::3\_motifs | PSSM | 9.19 | 2293 | | Node 4 | **----wwTATAgACAAAwww** |  |  | coexpression\_cluster3\_8::4\_motifs | PSSM | 8.90 | 2875 | | Node 5 | **---AtaTATAtAyATAta-** |  |  | coexpression\_cluster3\_8::2\_motifs | PSSM | 10.62 | 2458 | | Node 6 | **--wawATACACAcAtaw--** |  |  | coexpression\_cluster3\_8::3\_motifs | PSSM | 8.97 | 2496 | | Node 7 | **---AwaTATAkAyATAta-** |  |  | coexpression\_cluster3\_8::3\_motifs | PSSM | 10.23 | 3204 | | Node 8 | **waTatAtACACAcATaw--** |  |  | coexpression\_cluster3\_8::4\_motifs | PSSM | 9.91 | 3063 | | Node 9 | **----wwwwTAgAcAAAwww** |  |  | coexpression\_cluster3\_8::5\_motifs | PSSM | 8.60 | 4053 | | Node 10 | **waTatAtACACAcATaw--** |  |  | coexpression\_cluster3\_8::5\_motifs | PSSM | 9.94 | 3176 | | Node 11 | **waTatATAyAyAcATAwa-** |  |  | coexpression\_cluster3\_8::8\_motifs | PSSM | 9.93 | 6380 | | Node 12 | **waTawaTAtAbAcAwAwww** |  |  | coexpression\_cluster3\_8::13\_motifs | PSSM | 9.30 | 10433 |  **cluster\_2**   | Branch Motifs | | | | | | --- | --- | --- | --- | --- | | Node | Consensus | Logo | Logo (Reverse) | Collections | Matrix (Transfac format) | IC | Nb sites | | Node 1 | **---wwAAAmAAAAww-** |  |  | coexpression\_cluster3\_8::2\_motifs | PSSM | 9.18 | 4121 | | --- | --- | --- | --- | --- | --- | --- | --- | | Node 2 | **wwaAAArAmAAAAww-** |  |  | coexpression\_cluster3\_8::2\_motifs | PSSM | 9.71 | 3612 | | Node 3 | **waaAAArAmAAAAww-** |  |  | coexpression\_cluster3\_8::3\_motifs | PSSM | 9.52 | 3954 | | Node 4 | **---wwAAAmAAAAwwA** |  |  | coexpression\_cluster3\_8::3\_motifs | PSSM | 10.59 | 4246 | | Node 5 | **waawAAAAmAAAAwwA** |  |  | coexpression\_cluster3\_8::6\_motifs | PSSM | 10.86 | 8200 | | Node 6 | **waawAAAAmAAAAwwA** |  |  | coexpression\_cluster3\_8::7\_motifs | PSSM | 10.77 | 8367 |  **cluster\_3**   | Branch Motifs | | | | | | --- | --- | --- | --- | --- | | Node | Consensus | Logo | Logo (Reverse) | Collections | Matrix (Transfac format) | IC | Nb sites | | Node 1 | **GtaTGTACAta** |  |  | coexpression\_cluster3\_8::2\_motifs | PSSM | 10.33 | 327 | | --- | --- | --- | --- | --- | --- | --- | --- | | Node 2 | **-TATGCAyATA** |  |  | coexpression\_cluster3\_8::2\_motifs | PSSM | 10.75 | 179 | | Node 3 | **GTATGyAYAta** |  |  | coexpression\_cluster3\_8::4\_motifs | PSSM | 9.40 | 506 |  **cluster\_4**   | Branch Motifs | | | | | | --- | --- | --- | --- | --- | | Node | Consensus | Logo | Logo (Reverse) | Collections | Matrix (Transfac format) | IC | Nb sites | | Node 1 | **wtywwttyrCACACACrhvrrk** |  |  | coexpression\_cluster3\_8::2\_motifs | PSSM | 10.26 | 180 | | --- | --- | --- | --- | --- | --- | --- | --- |  **cluster\_5**   | Branch Motifs | | | | | | --- | --- | --- | --- | --- | | Node | Consensus | Logo | Logo (Reverse) | Collections | Matrix (Transfac format) | IC | Nb sites | | Singleton | **aStaGmCarR** |  |  | coexpression\_cluster3\_8 : 1 motif | PSSM | 7.06 | 190 | | --- | --- | --- | --- | --- | --- | --- | --- |  **cluster\_6**   | Branch Motifs | | | | | | --- | --- | --- | --- | --- | | Node | Consensus | Logo | Logo (Reverse) | Collections | Matrix (Transfac format) | IC | Nb sites | | Singleton | **awACATGTwt** |  |  | coexpression\_cluster3\_8 : 1 motif | PSSM | 9.24 | 222 | | --- | --- | --- | --- | --- | --- | --- | --- |  **cluster\_7**   | Branch Motifs | | | | | | --- | --- | --- | --- | --- | | Node | Consensus | Logo | Logo (Reverse) | Collections | Matrix (Transfac format) | IC | Nb sites | | Singleton | **wwAGAATGtw** |  |  | coexpression\_cluster3\_8 : 1 motif | PSSM | 9.15 | 104 | | --- | --- | --- | --- | --- | --- | --- | --- |  **cluster\_8**   | Branch Motifs | | | | | | --- | --- | --- | --- | --- | | Node | Consensus | Logo | Logo (Reverse) | Collections | Matrix (Transfac format) | IC | Nb sites | | Singleton | **KasSmttkBCCSSCC** |  |  | coexpression\_cluster3\_8 : 1 motif | PSSM | 10.82 | 18 | | --- | --- | --- | --- | --- | --- | --- | --- |   - **Each cluster has a different color.** - **Click on the upper buttons to display separately the information of each cluster.** - **Click on the circles at each tree to display its corresponding merged motifs.**   Definitions  - **Logo tree.**  This results shows the hierarchical tree with its logo alignment of one cluster. The logos are shown in Forward (left logo) and Reverse (right logo) orientation. - **Branch-motifs.**  On the trees are displayed the branch number that is the number in which the motifs were incorporated in the tree. The Branch-Motifs table shows the logo in both orientations and the link to the file in TRANSFAC format with the branch-motif. |
|  | |  |  |  |  |  |  |  |  |  |  |  |  |  |  |  |  |  |  |  |  |  |  |  |  |  |  |  |  |  |  |  |  |  |  |  |  |  |  |  |  |  |  |  |  |  |  |  |  |  |  |  |  |  |  |  |  |  |  |  |  |  |  |  |  |  |  |  |  |  |  |  |  |  |  |  |  |  |  |  |  |  |  |  |  |  |  |  |  |  |  |  |  |  |  |  |  |  |  |  |  |  |  |  |  |  |  |  |  |  |  |  |  |  |  |  |  |  |  |  |  |  |  |  |  |  |  |  |  |  |  |  |  |  |  |  |  |  |  |  |  |  |  |  |  |  |  |  |  |  |  |  |  |  |  |  |  |  |  |  |  |  |  |  |  |  |  |  |  |  |  |  |  |  |  |  |  |  |  |  |  |  |  |  |  |  |  |  |  |  |  |  |  |  |  |  |  |  |  |  |  |  |  |  |  |  |  |  |  |  |  |  |  |  |  |  |  |  |  |  |  |  |  |  |  |  |  |  |  |  |  |  |  |  |  |  |  |  |  |  |  |  |  |  |  |  |  |  |  |  |  |  |  |  |  |  |  |  |  |  |  |  |  |  |  |  |  |  |  |  |  |  |  |  |  |  |  |  |  |  |  |  |  |  |  |  |  |  |  |  |  |  |  |  |  |  |  |  |  |  |  |  |  |  |  |  |  |  |  |  |  |  |  |  |  |  |  |  |  |  |  |  |  |  |  |  |  |  |  |  |  |  |  |  |  |  |  |  |  |  |  |  |  | | --- | --- | --- | --- | --- | --- | --- | --- | --- | --- | --- | --- | --- | --- | --- | --- | --- | --- | --- | --- | --- | --- | --- | --- | --- | --- | --- | --- | --- | --- | --- | --- | --- | --- | --- | --- | --- | --- | --- | --- | --- | --- | --- | --- | --- | --- | --- | --- | --- | --- | --- | --- | --- | --- | --- | --- | --- | --- | --- | --- | --- | --- | --- | --- | --- | --- | --- | --- | --- | --- | --- | --- | --- | --- | --- | --- | --- | --- | --- | --- | --- | --- | --- | --- | --- | --- | --- | --- | --- | --- | --- | --- | --- | --- | --- | --- | --- | --- | --- | --- | --- | --- | --- | --- | --- | --- | --- | --- | --- | --- | --- | --- | --- | --- | --- | --- | --- | --- | --- | --- | --- | --- | --- | --- | --- | --- | --- | --- | --- | --- | --- | --- | --- | --- | --- | --- | --- | --- | --- | --- | --- | --- | --- | --- | --- | --- | --- | --- | --- | --- | --- | --- | --- | --- | --- | --- | --- | --- | --- | --- | --- | --- | --- | --- | --- | --- | --- | --- | --- | --- | --- | --- | --- | --- | --- | --- | --- | --- | --- | --- | --- | --- | --- | --- | --- | --- | --- | --- | --- | --- | --- | --- | --- | --- | --- | --- | --- | --- | --- | --- | --- | --- | --- | --- | --- | --- | --- | --- | --- | --- | --- | --- | --- | --- | --- | --- | --- | --- | --- | --- | --- | --- | --- | --- | --- | --- | --- | --- | --- | --- | --- | --- | --- | --- | --- | --- | --- | --- | --- | --- | --- | --- | --- | --- | --- | --- | --- | --- | --- | --- | --- | --- | --- | --- | --- | --- | --- | --- | --- | --- | --- | --- | --- | --- | --- | --- | --- | --- | --- | --- | --- | --- | --- | --- | --- | --- | --- | --- | --- | --- | --- | --- | --- | --- | --- | --- | --- | --- | --- | --- | --- | --- | --- | --- | --- | --- | --- | --- | --- | --- | --- | --- | --- | --- | --- | --- | --- | --- | --- | --- | --- | --- | --- | --- | --- | --- | --- | --- | --- | --- | --- | --- | --- | --- | --- | --- | --- | --- | --- | --- | --- | --- | --- | --- | --- | --- | --- | --- | --- | --- | --- | --- | | **Individual Motif View**  Individual Motif View  | Motif id | Motif name | Cluster | Collection | Width | IC | Number of sites | Consensus | Consensus (Rev) | Logo | Logo (Rev) | | --- | --- | --- | --- | --- | --- | --- | --- | --- | --- | --- | | coexpression\_cluster3\_8\_m20\_cluster3\_8\_motif20\_m | cluster3\_8\_motif20\_m | cluster\_1 | coexpression\_cluster3\_8 | 19 | 11.04 | 113 | **+++++RTATGTGTGTGT++** | **++ACACACACATAy+++++** |  |  | | coexpression\_cluster3\_8\_m16\_cluster3\_8\_motif16\_r | cluster3\_8\_motif16\_r | cluster\_1 | coexpression\_cluster3\_8 | 19 | 11.27 | 567 | **AWATRTGTGTATATATW++** | **++waTATAtACAcAyATwt** |  |  | | coexpression\_cluster3\_8\_m24\_cluster3\_8\_motif24\_m | cluster3\_8\_motif24\_m | cluster\_2 | coexpression\_cluster3\_8 | 16 | 15.21 | 125 | **+++TATTTTTTTTTTT** | **AAAAAAAAAAATA+++** |  |  | | coexpression\_cluster3\_8\_m17\_cluster3\_8\_motif17\_r | cluster3\_8\_motif17\_r | cluster\_7 | coexpression\_cluster3\_8 | 10 | 9.15 | 104 | **wwAGAATGtw** | **WACATTCTWW** |  |  | | coexpression\_cluster3\_8\_m23\_cluster3\_8\_motif23\_m | cluster3\_8\_motif23\_m | cluster\_5 | coexpression\_cluster3\_8 | 10 | 7.06 | 190 | **AgTAGACAAr** | **YTTGTCTACT** |  |  | | coexpression\_cluster3\_8\_m27\_cluster3\_8\_motif27\_m | cluster3\_8\_motif27\_m | cluster\_3 | coexpression\_cluster3\_8 | 11 | 10.75 | 118 | **+TATGCATAT+** | **+ATATGCATA+** |  |  | | coexpression\_cluster3\_8\_m21\_cluster3\_8\_motif21\_m | cluster3\_8\_motif21\_m | cluster\_3 | coexpression\_cluster3\_8 | 11 | 10.59 | 101 | **GTATGTACAT+** | **+ATGTACATAC** |  |  | | coexpression\_cluster3\_8\_m29\_cluster3\_8\_motif29\_m | cluster3\_8\_motif29\_m | cluster\_4 | coexpression\_cluster3\_8 | 22 | 10.90 | 120 | **wtswwttTGCACACACrhmvrg** | **CYBKDYGTGTGTGCAAAWWSAW** |  |  | | coexpression\_cluster3\_8\_m18\_cluster3\_8\_motif18\_r | cluster3\_8\_motif18\_r | cluster\_3 | coexpression\_cluster3\_8 | 11 | 9.43 | 226 | **+taTGTACAta** | **TATGTACATA+** |  |  | | coexpression\_cluster3\_8\_m5\_cluster3\_8\_motif5\_r | cluster3\_8\_motif5\_r | cluster\_1 | coexpression\_cluster3\_8 | 19 | 10.03 | 2042 | **++++waTATAtAyATAwa+** | **+TWTATRTATATATW++++** |  |  | | coexpression\_cluster3\_8\_m25\_cluster3\_8\_motif25\_m | cluster3\_8\_motif25\_m | cluster\_2 | coexpression\_cluster3\_8 | 16 | 12.17 | 342 | **TTTTTTYTTTTTTTT+** | **+AAAAaAaarAAaAAA** |  |  | | coexpression\_cluster3\_8\_m14\_cluster3\_8\_motif14\_r | cluster3\_8\_motif14\_r | cluster\_2 | coexpression\_cluster3\_8 | 16 | 9.11 | 837 | **+++wwcAAmAAAAww+** | **+WWTTTTKTTGWW+++** |  |  | | coexpression\_cluster3\_8\_m9\_cluster3\_8\_motif9\_r | cluster3\_8\_motif9\_r | cluster\_1 | coexpression\_cluster3\_8 | 19 | 8.90 | 821 | **+++wwATACACACAwww++** | **++WWWTGTGTGTATWW+++** |  |  | | coexpression\_cluster3\_8\_m1\_cluster3\_8\_motif1\_r | cluster3\_8\_motif1\_r | cluster\_1 | coexpression\_cluster3\_8 | 19 | 9.63 | 851 | **++++wwTATAgAcAAAAww** | **WWTTTTGTCTATAWW++++** |  |  | | coexpression\_cluster3\_8\_m3\_cluster3\_8\_motif3\_r | cluster3\_8\_motif3\_r | cluster\_1 | coexpression\_cluster3\_8 | 19 | 9.10 | 1318 | **+++awATAyACAyATaw++** | **++WTATRTGTRTATWT+++** |  |  | | coexpression\_cluster3\_8\_m8\_cluster3\_8\_motif8\_r | cluster3\_8\_motif8\_r | cluster\_2 | coexpression\_cluster3\_8 | 16 | 9.56 | 3284 | **+++wwAAAmAAAAww+** | **+WWTTTTKTTTWW+++** |  |  | | coexpression\_cluster3\_8\_m28\_cluster3\_8\_motif28\_m | cluster3\_8\_motif28\_m | cluster\_3 | coexpression\_cluster3\_8 | 11 | 11.33 | 61 | **+TATGCACAyA** | **TRTGTGCATA+** |  |  | | coexpression\_cluster3\_8\_m12\_cluster3\_8\_motif12\_r | cluster3\_8\_motif12\_r | cluster\_1 | coexpression\_cluster3\_8 | 19 | 8.95 | 582 | **++++WWTATAGACAATWWW** | **wwwaTTGTcTATAww++++** |  |  | | coexpression\_cluster3\_8\_m6\_cluster3\_8\_motif6\_r | cluster3\_8\_motif6\_r | cluster\_2 | coexpression\_cluster3\_8 | 16 | 9.77 | 1127 | **WWAWAARAMAAAAWW+** | **+wwTTTTkTyTTwTww** |  |  | | coexpression\_cluster3\_8\_m4\_cluster3\_8\_motif4\_r | cluster3\_8\_motif4\_r | cluster\_1 | coexpression\_cluster3\_8 | 19 | 10.17 | 357 | **++ATATGTGTGTGYATW++** | **++watrCACACAcATat++** |  |  | | coexpression\_cluster3\_8\_m11\_cluster3\_8\_motif11\_r | cluster3\_8\_motif11\_r | cluster\_1 | coexpression\_cluster3\_8 | 19 | 9.47 | 868 | **++++wwTATAkACAAAwww** | **WWWTTTGTMTATAWW++++** |  |  | | coexpression\_cluster3\_8\_m30\_cluster3\_8\_motif30\_m | cluster3\_8\_motif30\_m | cluster\_8 | coexpression\_cluster3\_8 | 15 | 10.82 | 18 | **kwtCATTTyCCCCyy** | **RRGGGGRAAATGAWM** |  |  | | coexpression\_cluster3\_8\_m26\_cluster3\_8\_motif26\_m | cluster3\_8\_motif26\_m | cluster\_4 | coexpression\_cluster3\_8 | 22 | 12.34 | 61 | **TvtwdtgCRCACACACrwrkmw** | **WKMYWYGTGTGTGYGCAHWABA** |  |  | | coexpression\_cluster3\_8\_m2\_cluster3\_8\_motif2\_r | cluster3\_8\_motif2\_r | cluster\_2 | coexpression\_cluster3\_8 | 16 | 9.91 | 2485 | **+wwAAArAmAAAAww+** | **+WWTTTTKTYTTTWW+** |  |  | | coexpression\_cluster3\_8\_m10\_cluster3\_8\_motif10\_r | cluster3\_8\_motif10\_r | cluster\_1 | coexpression\_cluster3\_8 | 19 | 9.07 | 746 | **++++WWWTGTMTATATW++** | **++waTATAkACAwww++++** |  |  | | coexpression\_cluster3\_8\_m13\_cluster3\_8\_motif13\_r | cluster3\_8\_motif13\_r | cluster\_1 | coexpression\_cluster3\_8 | 19 | 9.29 | 574 | **++++wwwrTAgACAAAAww** | **WWTTTTGTCTAYWWW++++** |  |  | | coexpression\_cluster3\_8\_m22\_cluster3\_8\_motif22\_m | cluster3\_8\_motif22\_m | cluster\_2 | coexpression\_cluster3\_8 | 16 | 11.10 | 168 | **+++TTATTATTWTT++** | **++AAwAATAATAA+++** |  |  | | coexpression\_cluster3\_8\_m19\_cluster3\_8\_motif19\_m | cluster3\_8\_motif19\_m | cluster\_1 | coexpression\_cluster3\_8 | 19 | 11.71 | 416 | **+++ATRYAYATrCAYAya+** | **+TRTRTGYATRTRYAT+++** |  |  | | coexpression\_cluster3\_8\_m7\_cluster3\_8\_motif7\_r | cluster3\_8\_motif7\_r | cluster\_1 | coexpression\_cluster3\_8 | 19 | 9.24 | 1178 | **+++++WWTTARAMAAAAWW** | **wwTTTTkTyTAAww+++++** |  |  | | coexpression\_cluster3\_8\_m15\_cluster3\_8\_motif15\_r | cluster3\_8\_motif15\_r | cluster\_6 | coexpression\_cluster3\_8 | 10 | 9.24 | 222 | **awACATGTwt** | **AWACATGTWT** |  |  |  - Click on the column names to change the order of the data. - Write the name of one cluster, collection or a pattern in the *Search* window. | | **Heatmap View**  Heatmap View Distance table | PDF | |

**Additional Files**

## Exported files

| File | Description |
| --- | --- |
| **Pairwise comparison** | This table shows the pairwise comparison between all the input motifs using different metrics. This is the *compare-matrices* result. |
| **Matrix description** | This table shows information of each input motif. |
| **Cluster-wise Motif Files [zip]** | One TRANSFAC file is generated for each cluster including all the input motifs grouped in the corresponding cluster. |
| **Root motifs** | A file with the root matrix of each cluster. |
| **Motif collections per cluster** | A tab separated file containing the counts of motif of each collection on each cluster. |
| **Clusters** | A tab separated file containing the clusters and their correspondng motifs. |
| **Central motifs IDs** | A tab separated file containing the central motif, that one who is the most similar to all the others motif in the same cluster. |
| **Central motifs (in TRANSFAC format)** | A transfac file containing the central motif, that one who is the most similar to all the others motif within the same cluster. |
| **Input motifs** | The input motifs analyzed with matrix-clustering. |

- **References**
  1. Castro-Mondragon JA et al.  RSAT matrix-clustering: dynamic exploration and redundancy reduction of transcription factor binding motif collections. doi: 10.1093/nar/gkx314; Nucleic Acids Research (2017)
  2. **Last RSAT release:** Nguyen NTT et al. RSAT 2018: regulatory sequence analysis tools 20th anniversary. Nucleic Acids Research (2018).
- **Additional Information**
  - Jacques van Helden course
- **Contact**
  - Jaime Castro-Mondragon - **j.a.c.mondragon@ncmm.uio.no**
  - Morgane Thomas-Chollier - **mthomas@biologie.ens.fr**
  - Jacques van Helden - **Jacques.van-Helden@univ-amu.fr**
