# Supplementary material for: Transcriptome analysis reveals a de novo DNA element that may interact with chromatin-associated proteins in Plasmodium berghei during erythrocytic development
Source: Sci Rep. 2025 May 28;15:18621. doi: 10.1038/s41598-025-03586-4 (PMC12120095; doi:10.1038/s41598-025-03586-4)
Supplement: Supplementary file 2 — Supplementary Information 2. [file 41598_2025_3586_MOESM2_ESM.zip › Supplementary File S2/Clustering/matrix-clustering_SUMMARY_cluster3_9.html]

matrix-clustering coexpression\_cluster3\_9


# RSAT - matrix-clustering result

### Analysis: coexpression\_cluster3\_9 (08/09/2023 16:16)

### Command

```
matrix-clustering  -v 1 -max_matrices 300 -matrix coexpression_cluster3_9 $RSAT/public_html/tmp/www-data/2023/09/08/matrix-clustering_2023-09-08.161614_08Wx62/matrix-clustering_query_matrices.transfac transfac -hclust_method average -calc sum -title coexpression_cluster3_9 -metric_build_tree Ncor -lth w 5 -lth cor 0.6 -lth Ncor 0.4 -quick -label_in_tree name -return json,heatmap -o $RSAT/public_html/tmp/www-data/2023/09/08/matrix-clustering_2023-09-08.161614_08Wx62/matrix-clustering
```


## Results Summary

|  |  |  |  |  |  |  |  |  |  |  |
| --- | --- | --- | --- | --- | --- | --- | --- | --- | --- | --- |
| Nb Input motifs | Nb Input collections | Nb Clusters Found | Download   Root motifs | Complete results [zip] | Parameters used  |  |  |  | | --- | --- | --- | | Linkage method | Similarity metric | Thresholds to partition the tree | | | |
| 25 | 1 | 9 | **Download** | Download | |  |  |  | | --- | --- | --- | | average | Ncor | **Ncor** = 0.4 **cor** = 0.6 | |

|  |  |
| --- | --- |
|  | **Logo Forest (dynamic browsing)**  **Logo Forest (rapid overview - low image quality)**  cluster\_1  cluster\_2  cluster\_3  cluster\_4  cluster\_5  cluster\_6  cluster\_7  cluster\_8  cluster\_9 |
|  | |  |  |  |  |  |  |  |  |  |  | | --- | --- | --- | --- | --- | --- | --- | --- | --- | --- | | **Clusters Summary**  Clusters Summary Table  | Root Motif | Root Motif (Reverse) | Cluster ID | # Motifs | Motif\_Name::Collection | Number of Motifs by Collection | Root Motif (transfac Format) | | --- | --- | --- | --- | --- | --- | --- | |  | | --- | |  | | cluster\_1 | 8 | cluster3\_9\_motif16\_m::coexpression\_cluster3\_9:: cluster3\_9\_motif1\_r::coexpression\_cluster3\_9:: cluster3\_9\_motif2\_r::coexpression\_cluster3\_9:: cluster3\_9\_motif12\_r::coexpression\_cluster3\_9:: cluster3\_9\_motif5\_r::coexpression\_cluster3\_9:: cluster3\_9\_motif3\_r::coexpression\_cluster3\_9:: cluster3\_9\_motif6\_r::coexpression\_cluster3\_9:: cluster3\_9\_motif9\_r::coexpression\_cluster3\_9:: | coexpression\_cluster3\_9::8\_motifs | Show matrix |
| cluster\_2 | 3 | cluster3\_9\_motif24\_m::coexpression\_cluster3\_9:: cluster3\_9\_motif23\_m::coexpression\_cluster3\_9:: cluster3\_9\_motif4\_r::coexpression\_cluster3\_9:: | coexpression\_cluster3\_9::3\_motifs | Show matrix | | |
| cluster\_3 | 6 | cluster3\_9\_motif11\_r::coexpression\_cluster3\_9:: cluster3\_9\_motif7\_r::coexpression\_cluster3\_9:: cluster3\_9\_motif19\_m::coexpression\_cluster3\_9:: cluster3\_9\_motif20\_m::coexpression\_cluster3\_9:: cluster3\_9\_motif17\_m::coexpression\_cluster3\_9:: cluster3\_9\_motif21\_m::coexpression\_cluster3\_9:: | coexpression\_cluster3\_9::6\_motifs | Show matrix | | |
| cluster\_4 | 3 | cluster3\_9\_motif14\_m::coexpression\_cluster3\_9:: cluster3\_9\_motif15\_m::coexpression\_cluster3\_9:: cluster3\_9\_motif18\_m::coexpression\_cluster3\_9:: | coexpression\_cluster3\_9::3\_motifs | Show matrix | | |
| cluster\_5 | 1 | cluster3\_9\_motif13\_r::coexpression\_cluster3\_9 | coexpression\_cluster3\_9 : 1 motif | Show matrix | | |
| cluster\_6 | 1 | cluster3\_9\_motif8\_r::coexpression\_cluster3\_9 | coexpression\_cluster3\_9 : 1 motif | Show matrix | | |
| cluster\_7 | 1 | cluster3\_9\_motif10\_r::coexpression\_cluster3\_9 | coexpression\_cluster3\_9 : 1 motif | Show matrix | | |
| cluster\_8 | 1 | cluster3\_9\_motif22\_m::coexpression\_cluster3\_9 | coexpression\_cluster3\_9 : 1 motif | Show matrix | | |
| cluster\_9 | 1 | cluster3\_9\_motif25\_m::coexpression\_cluster3\_9 | coexpression\_cluster3\_9 : 1 motif | Show matrix | | |

- Note: by default the table displays the first 50 clusters. Click on the *Show X entries* button to display more (or less) clusters.
- Click on the column names to change the order of the data.
- Use the *Search* window to find your motif (s) of interest.

| **Individual Cluster View**  Individual Cluster View **Show All**  **Hide All**  **cluster\_1**  **cluster\_2**  **cluster\_3**  **cluster\_4**  **cluster\_5**  **cluster\_6**  **cluster\_7**  **cluster\_8**  **cluster\_9**  **Display Logo Trees**   **cluster\_1**    **cluster\_2**    **cluster\_3**    **cluster\_4**    **cluster\_5**    **cluster\_6**    **cluster\_7**    **cluster\_8**    **cluster\_9**  **Display Branch-Motifs** **cluster\_1**   | Branch Motifs | | | | | | --- | --- | --- | --- | --- | | Node | Consensus | Logo | Logo (Reverse) | Collections | Matrix (Transfac format) | IC | Nb sites | | Node 1 | **waAAAtAgCTAGCTAktwwww--** |  |  | coexpression\_cluster3\_9::2\_motifs | PSSM | 11.92 | 144 | | --- | --- | --- | --- | --- | --- | --- | --- | | Node 2 | **--wwwtAgCTAGCTATtTtw---** |  |  | coexpression\_cluster3\_9::2\_motifs | PSSM | 10.66 | 210 | | Node 3 | **wwAAAtAgCTAGCTAtttwww--** |  |  | coexpression\_cluster3\_9::3\_motifs | PSSM | 11.42 | 272 | | Node 4 | **-wwwwTAgCTAGCTATtTtw---** |  |  | coexpression\_cluster3\_9::3\_motifs | PSSM | 10.71 | 337 | | Node 5 | **wwwwwTAgCTAGCTATtTwww--** |  |  | coexpression\_cluster3\_9::6\_motifs | PSSM | 10.84 | 609 | | Node 6 | **wwwwwTAgCTAGCTAttTwwwww** |  |  | coexpression\_cluster3\_9::7\_motifs | PSSM | 11.19 | 635 | | Node 7 | **wwwwwtAgCTAGCTAttTwwwww** |  |  | coexpression\_cluster3\_9::8\_motifs | PSSM | 11.23 | 683 |  **cluster\_2**   | Branch Motifs | | | | | | --- | --- | --- | --- | --- | | Node | Consensus | Logo | Logo (Reverse) | Collections | Matrix (Transfac format) | IC | Nb sites | | Node 1 | **-----wwtGTTCATAAww** |  |  | coexpression\_cluster3\_9::2\_motifs | PSSM | 9.21 | 289 | | --- | --- | --- | --- | --- | --- | --- | --- | | Node 2 | **kymTGwwtGTTCATAAww** |  |  | coexpression\_cluster3\_9::3\_motifs | PSSM | 13.60 | 306 |  **cluster\_3**   | Branch Motifs | | | | | | --- | --- | --- | --- | --- | | Node | Consensus | Logo | Logo (Reverse) | Collections | Matrix (Transfac format) | IC | Nb sites | | Node 1 | **-wwAAAAAAAAww--** |  |  | coexpression\_cluster3\_9::2\_motifs | PSSM | 8.66 | 6119 | | --- | --- | --- | --- | --- | --- | --- | --- | | Node 2 | **rAAAAAAAArAAAAA** |  |  | coexpression\_cluster3\_9::2\_motifs | PSSM | 12.97 | 375 | | Node 3 | **-AAwAAAAATAAWAw** |  |  | coexpression\_cluster3\_9::2\_motifs | PSSM | 13.36 | 100 | | Node 4 | **rAAAAAAAArAAAAA** |  |  | coexpression\_cluster3\_9::4\_motifs | PSSM | 12.12 | 475 | | Node 5 | **rwaAAAAAAAAwwAA** |  |  | coexpression\_cluster3\_9::6\_motifs | PSSM | 10.75 | 6594 |  **cluster\_4**   | Branch Motifs | | | | | | --- | --- | --- | --- | --- | | Node | Consensus | Logo | Logo (Reverse) | Collections | Matrix (Transfac format) | IC | Nb sites | | Node 1 | **ATATATRTATryATRT** |  |  | coexpression\_cluster3\_9::2\_motifs | PSSM | 12.77 | 271 | | --- | --- | --- | --- | --- | --- | --- | --- | | Node 2 | **ATATATaTATryATRT** |  |  | coexpression\_cluster3\_9::3\_motifs | PSSM | 12.58 | 375 |  **cluster\_5**   | Branch Motifs | | | | | | --- | --- | --- | --- | --- | | Node | Consensus | Logo | Logo (Reverse) | Collections | Matrix (Transfac format) | IC | Nb sites | | Singleton | **wayAGCCAAAww** |  |  | coexpression\_cluster3\_9 : 1 motif | PSSM | 9.30 | 58 | | --- | --- | --- | --- | --- | --- | --- | --- |  **cluster\_6**   | Branch Motifs | | | | | | --- | --- | --- | --- | --- | | Node | Consensus | Logo | Logo (Reverse) | Collections | Matrix (Transfac format) | IC | Nb sites | | Singleton | **wwAAGCTACww** |  |  | coexpression\_cluster3\_9 : 1 motif | PSSM | 7.24 | 156 | | --- | --- | --- | --- | --- | --- | --- | --- |  **cluster\_7**   | Branch Motifs | | | | | | --- | --- | --- | --- | --- | | Node | Consensus | Logo | Logo (Reverse) | Collections | Matrix (Transfac format) | IC | Nb sites | | Singleton | **wkGATATCmw** |  |  | coexpression\_cluster3\_9 : 1 motif | PSSM | 8.96 | 50 | | --- | --- | --- | --- | --- | --- | --- | --- |  **cluster\_8**   | Branch Motifs | | | | | | --- | --- | --- | --- | --- | | Node | Consensus | Logo | Logo (Reverse) | Collections | Matrix (Transfac format) | IC | Nb sites | | Singleton | **aGttGC** |  |  | coexpression\_cluster3\_9 : 1 motif | PSSM | 7.64 | 51 | | --- | --- | --- | --- | --- | --- | --- | --- |  **cluster\_9**   | Branch Motifs | | | | | | --- | --- | --- | --- | --- | | Node | Consensus | Logo | Logo (Reverse) | Collections | Matrix (Transfac format) | IC | Nb sites | | Singleton | **SSCSCCCC** |  |  | coexpression\_cluster3\_9 : 1 motif | PSSM | 6.83 | 17 | | --- | --- | --- | --- | --- | --- | --- | --- |   - **Each cluster has a different color.** - **Click on the upper buttons to display separately the information of each cluster.** - **Click on the circles at each tree to display its corresponding merged motifs.**   Definitions  - **Logo tree.**  This results shows the hierarchical tree with its logo alignment of one cluster. The logos are shown in Forward (left logo) and Reverse (right logo) orientation. - **Branch-motifs.**  On the trees are displayed the branch number that is the number in which the motifs were incorporated in the tree. The Branch-Motifs table shows the logo in both orientations and the link to the file in TRANSFAC format with the branch-motif. |
|  | |  |  |  |  |  |  |  |  |  |  |  |  |  |  |  |  |  |  |  |  |  |  |  |  |  |  |  |  |  |  |  |  |  |  |  |  |  |  |  |  |  |  |  |  |  |  |  |  |  |  |  |  |  |  |  |  |  |  |  |  |  |  |  |  |  |  |  |  |  |  |  |  |  |  |  |  |  |  |  |  |  |  |  |  |  |  |  |  |  |  |  |  |  |  |  |  |  |  |  |  |  |  |  |  |  |  |  |  |  |  |  |  |  |  |  |  |  |  |  |  |  |  |  |  |  |  |  |  |  |  |  |  |  |  |  |  |  |  |  |  |  |  |  |  |  |  |  |  |  |  |  |  |  |  |  |  |  |  |  |  |  |  |  |  |  |  |  |  |  |  |  |  |  |  |  |  |  |  |  |  |  |  |  |  |  |  |  |  |  |  |  |  |  |  |  |  |  |  |  |  |  |  |  |  |  |  |  |  |  |  |  |  |  |  |  |  |  |  |  |  |  |  |  |  |  |  |  |  |  |  |  |  |  |  |  |  |  |  |  |  |  |  |  |  |  |  |  |  |  |  |  |  |  |  |  |  |  |  |  |  |  |  |  |  |  |  |  |  |  |  |  |  |  |  |  |  |  |  |  |  |  |  |  |  |  |  |  | | --- | --- | --- | --- | --- | --- | --- | --- | --- | --- | --- | --- | --- | --- | --- | --- | --- | --- | --- | --- | --- | --- | --- | --- | --- | --- | --- | --- | --- | --- | --- | --- | --- | --- | --- | --- | --- | --- | --- | --- | --- | --- | --- | --- | --- | --- | --- | --- | --- | --- | --- | --- | --- | --- | --- | --- | --- | --- | --- | --- | --- | --- | --- | --- | --- | --- | --- | --- | --- | --- | --- | --- | --- | --- | --- | --- | --- | --- | --- | --- | --- | --- | --- | --- | --- | --- | --- | --- | --- | --- | --- | --- | --- | --- | --- | --- | --- | --- | --- | --- | --- | --- | --- | --- | --- | --- | --- | --- | --- | --- | --- | --- | --- | --- | --- | --- | --- | --- | --- | --- | --- | --- | --- | --- | --- | --- | --- | --- | --- | --- | --- | --- | --- | --- | --- | --- | --- | --- | --- | --- | --- | --- | --- | --- | --- | --- | --- | --- | --- | --- | --- | --- | --- | --- | --- | --- | --- | --- | --- | --- | --- | --- | --- | --- | --- | --- | --- | --- | --- | --- | --- | --- | --- | --- | --- | --- | --- | --- | --- | --- | --- | --- | --- | --- | --- | --- | --- | --- | --- | --- | --- | --- | --- | --- | --- | --- | --- | --- | --- | --- | --- | --- | --- | --- | --- | --- | --- | --- | --- | --- | --- | --- | --- | --- | --- | --- | --- | --- | --- | --- | --- | --- | --- | --- | --- | --- | --- | --- | --- | --- | --- | --- | --- | --- | --- | --- | --- | --- | --- | --- | --- | --- | --- | --- | --- | --- | --- | --- | --- | --- | --- | --- | --- | --- | --- | --- | --- | --- | --- | --- | --- | --- | --- | --- | --- | --- | --- | --- | --- | --- | --- | --- | --- | --- | --- | --- | --- | --- | --- | --- | --- | --- | --- | --- | --- | --- | --- | | **Individual Motif View**  Individual Motif View  | Motif id | Motif name | Cluster | Collection | Width | IC | Number of sites | Consensus | Consensus (Rev) | Logo | Logo (Rev) | | --- | --- | --- | --- | --- | --- | --- | --- | --- | --- | --- | | coexpression\_cluster3\_9\_m17\_cluster3\_9\_motif17\_m | cluster3\_9\_motif17\_m | cluster\_3 | coexpression\_cluster3\_9 | 15 | 15.20 | 65 | **+AAAAAAAATAATAA** | **TTATTATTTTTTTT+** |  |  | | coexpression\_cluster3\_9\_m15\_cluster3\_9\_motif15\_m | cluster3\_9\_motif15\_m | cluster\_4 | coexpression\_cluster3\_9 | 16 | 13.74 | 63 | **+TATRTATATGCATAT** | **ATATGCATATAyATA+** |  |  | | coexpression\_cluster3\_9\_m6\_cluster3\_9\_motif6\_r | cluster3\_9\_motif6\_r | cluster\_1 | coexpression\_cluster3\_9 | 23 | 11.03 | 100 | **++WWWTAGCTAGCTATTTTW+++** | **+++waAaAtAGCTAGCTAwww++** |  |  | | coexpression\_cluster3\_9\_m22\_cluster3\_9\_motif22\_m | cluster3\_9\_motif22\_m | cluster\_8 | coexpression\_cluster3\_9 | 6 | 7.64 | 51 | **AGTTGC** | **GCAACT** |  |  | | coexpression\_cluster3\_9\_m4\_cluster3\_9\_motif4\_r | cluster3\_9\_motif4\_r | cluster\_2 | coexpression\_cluster3\_9 | 18 | 9.07 | 245 | **+++++wwtGTTCATAAww** | **WWTTATGAACAWW+++++** |  |  | | coexpression\_cluster3\_9\_m10\_cluster3\_9\_motif10\_r | cluster3\_9\_motif10\_r | cluster\_7 | coexpression\_cluster3\_9 | 10 | 8.96 | 50 | **wkGATATCmw** | **WKGATATCMW** |  |  | | coexpression\_cluster3\_9\_m12\_cluster3\_9\_motif12\_r | cluster3\_9\_motif12\_r | cluster\_1 | coexpression\_cluster3\_9 | 23 | 11.78 | 78 | **WAAAATAGCTAGCTAKTWWWW++** | **++wwwwamTAGCTAGcTaTtTtw** |  |  | | coexpression\_cluster3\_9\_m21\_cluster3\_9\_motif21\_m | cluster3\_9\_motif21\_m | cluster\_3 | coexpression\_cluster3\_9 | 15 | 14.71 | 35 | **+AATAAAAATAAAAT** | **ATTTTATTTTTATT+** |  |  | | coexpression\_cluster3\_9\_m11\_cluster3\_9\_motif11\_r | cluster3\_9\_motif11\_r | cluster\_3 | coexpression\_cluster3\_9 | 15 | 9.06 | 579 | **+wwGArAAAAAww++** | **++WWTTTTTYTCWW+** |  |  | | coexpression\_cluster3\_9\_m23\_cluster3\_9\_motif23\_m | cluster3\_9\_motif23\_m | cluster\_2 | coexpression\_cluster3\_9 | 18 | 11.16 | 44 | **+++++HTTGTTCATAAT+** | **+ATTATGAACAad+++++** |  |  | | coexpression\_cluster3\_9\_m5\_cluster3\_9\_motif5\_r | cluster3\_9\_motif5\_r | cluster\_1 | coexpression\_cluster3\_9 | 23 | 12.24 | 66 | **AAAAATARCTAGCTAKTTWWW++** | **++wwwAamTAGCTAGyTaTTTtt** |  |  | | coexpression\_cluster3\_9\_m24\_cluster3\_9\_motif24\_m | cluster3\_9\_motif24\_m | cluster\_2 | coexpression\_cluster3\_9 | 18 | 14.67 | 17 | **KYMTGAAYGTTCATA+++** | **+++TATGaACrTTCAkrm** |  |  | | coexpression\_cluster3\_9\_m8\_cluster3\_9\_motif8\_r | cluster3\_9\_motif8\_r | cluster\_6 | coexpression\_cluster3\_9 | 11 | 7.24 | 156 | **wwAAGCTACww** | **WWGTAGCTTWW** |  |  | | coexpression\_cluster3\_9\_m1\_cluster3\_9\_motif1\_r | cluster3\_9\_motif1\_r | cluster\_1 | coexpression\_cluster3\_9 | 23 | 12.63 | 26 | **+WWTTTRGMTAGCTAKCYAAAWW** | **wwTTTrgmTAGCTAkcyAAAww+** |  |  | | coexpression\_cluster3\_9\_m16\_cluster3\_9\_motif16\_m | cluster3\_9\_motif16\_m | cluster\_1 | coexpression\_cluster3\_9 | 23 | 9.79 | 48 | **+++++TRGCTAGYTR++++++++** | **++++++++yArCTAGCyA+++++** |  |  | | coexpression\_cluster3\_9\_m7\_cluster3\_9\_motif7\_r | cluster3\_9\_motif7\_r | cluster\_3 | coexpression\_cluster3\_9 | 15 | 8.95 | 5540 | **+wwAAAAAAAAww++** | **++WWTTTTTTTTWW+** |  |  | | coexpression\_cluster3\_9\_m9\_cluster3\_9\_motif9\_r | cluster3\_9\_motif9\_r | cluster\_1 | coexpression\_cluster3\_9 | 23 | 10.81 | 110 | **++wwawAgCTAGCTATwTtw+++** | **+++WAAWATAGCTAGCTWTWW++** |  |  | | coexpression\_cluster3\_9\_m3\_cluster3\_9\_motif3\_r | cluster3\_9\_motif3\_r | cluster\_1 | coexpression\_cluster3\_9 | 23 | 11.04 | 127 | **+WWTTTAGCTAGCTATWTWW+++** | **+++wwAwAtAGcTAGCTAAAww+** |  |  | | coexpression\_cluster3\_9\_m25\_cluster3\_9\_motif25\_m | cluster3\_9\_motif25\_m | cluster\_9 | coexpression\_cluster3\_9 | 8 | 6.83 | 17 | **scCsyCCC** | **GGGRSGGS** |  |  | | coexpression\_cluster3\_9\_m14\_cluster3\_9\_motif14\_m | cluster3\_9\_motif14\_m | cluster\_4 | coexpression\_cluster3\_9 | 16 | 11.82 | 105 | **+ATATAKATATA++++** | **++++TATATmTATAT+** |  |  | | coexpression\_cluster3\_9\_m19\_cluster3\_9\_motif19\_m | cluster3\_9\_motif19\_m | cluster\_3 | coexpression\_cluster3\_9 | 15 | 13.04 | 236 | **rAAAAAAaarRAAAA** | **TTTTYYTTTTTTTTY** |  |  | | coexpression\_cluster3\_9\_m13\_cluster3\_9\_motif13\_r | cluster3\_9\_motif13\_r | cluster\_5 | coexpression\_cluster3\_9 | 12 | 9.30 | 58 | **wayAGCCAAAww** | **WWTTTGGCTRTW** |  |  | | coexpression\_cluster3\_9\_m2\_cluster3\_9\_motif2\_r | cluster3\_9\_motif2\_r | cluster\_1 | coexpression\_cluster3\_9 | 23 | 11.26 | 128 | **WWAAATAGCTAGCTATTTWW+++** | **+++wwAaATAgcTAgcTATtTww** |  |  | | coexpression\_cluster3\_9\_m18\_cluster3\_9\_motif18\_m | cluster3\_9\_motif18\_m | cluster\_4 | coexpression\_cluster3\_9 | 16 | 12.34 | 208 | **ATATATRTATRYATR+** | **+yATryATAyATATAT** |  |  | | coexpression\_cluster3\_9\_m20\_cluster3\_9\_motif20\_m | cluster3\_9\_motif20\_m | cluster\_3 | coexpression\_cluster3\_9 | 15 | 12.93 | 139 | **+AAAAAAAAGAAAA+** | **+TTTTCTTTTTTTT+** |  |  |  - Click on the column names to change the order of the data. - Write the name of one cluster, collection or a pattern in the *Search* window. | | **Heatmap View**  Heatmap View Distance table | PDF | |

**Additional Files**

## Exported files

| File | Description |
| --- | --- |
| **Pairwise comparison** | This table shows the pairwise comparison between all the input motifs using different metrics. This is the *compare-matrices* result. |
| **Matrix description** | This table shows information of each input motif. |
| **Cluster-wise Motif Files [zip]** | One TRANSFAC file is generated for each cluster including all the input motifs grouped in the corresponding cluster. |
| **Root motifs** | A file with the root matrix of each cluster. |
| **Motif collections per cluster** | A tab separated file containing the counts of motif of each collection on each cluster. |
| **Clusters** | A tab separated file containing the clusters and their correspondng motifs. |
| **Central motifs IDs** | A tab separated file containing the central motif, that one who is the most similar to all the others motif in the same cluster. |
| **Central motifs (in TRANSFAC format)** | A transfac file containing the central motif, that one who is the most similar to all the others motif within the same cluster. |
| **Input motifs** | The input motifs analyzed with matrix-clustering. |

- **References**
  1. Castro-Mondragon JA et al.  RSAT matrix-clustering: dynamic exploration and redundancy reduction of transcription factor binding motif collections. doi: 10.1093/nar/gkx314; Nucleic Acids Research (2017)
  2. **Last RSAT release:** Nguyen NTT et al. RSAT 2018: regulatory sequence analysis tools 20th anniversary. Nucleic Acids Research (2018).
- **Additional Information**
  - Jacques van Helden course
- **Contact**
  - Jaime Castro-Mondragon - **j.a.c.mondragon@ncmm.uio.no**
  - Morgane Thomas-Chollier - **mthomas@biologie.ens.fr**
  - Jacques van Helden - **Jacques.van-Helden@univ-amu.fr**
