# Supplementary figures and images for: Transcriptome analysis reveals a de novo DNA element that may interact with chromatin-associated proteins in Plasmodium berghei during erythrocytic development (part 2 of 2)
Source: Sci Rep. 2025 May 28;15:18621. doi: 10.1038/s41598-025-03586-4 (PMC12120095; doi:10.1038/s41598-025-03586-4)

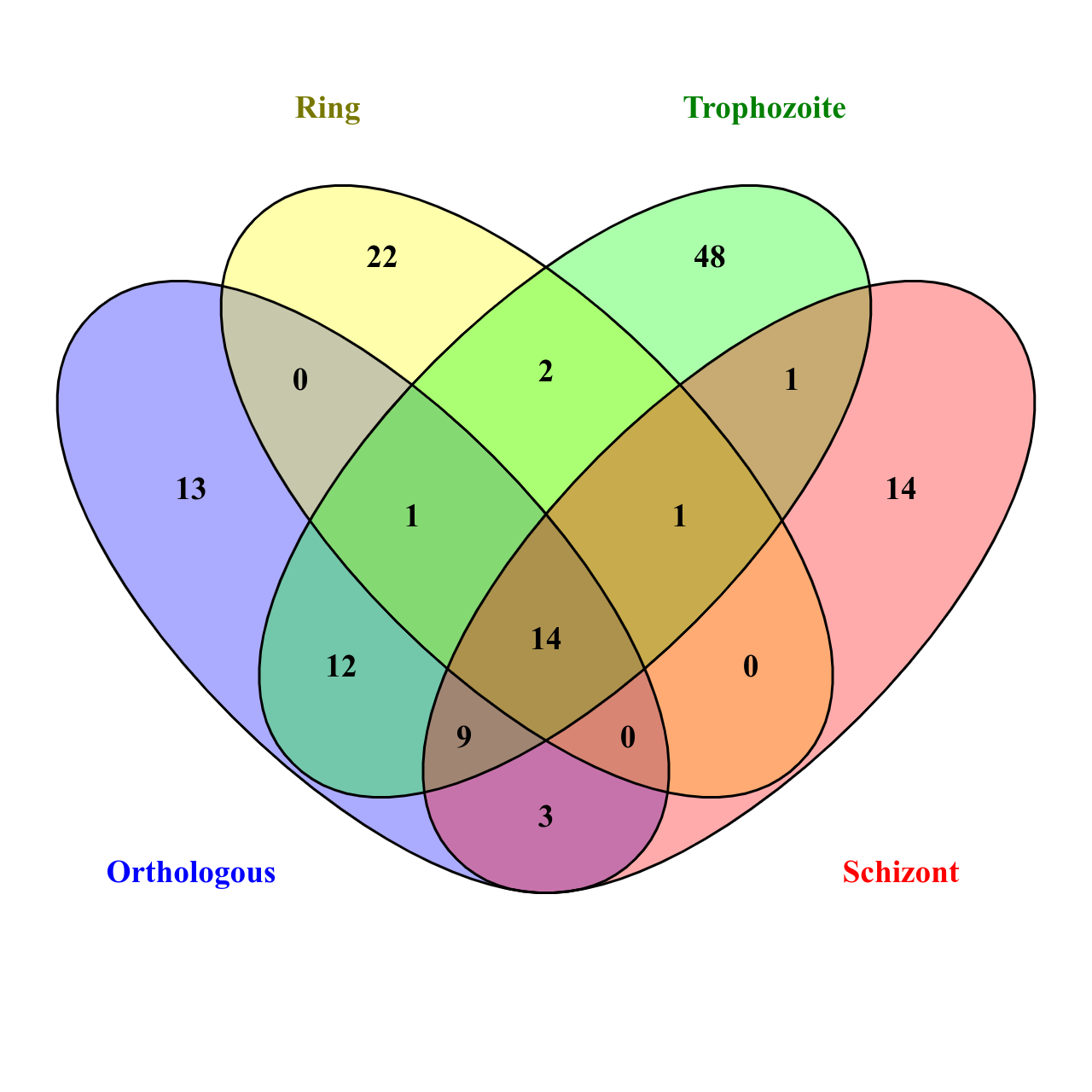

Supplement: Supplementary file 5 — Supplementary Information 5. [file 41598_2025_3586_MOESM5_ESM.zip › Supplementary File S6/Venn_common52_orthologous.png]

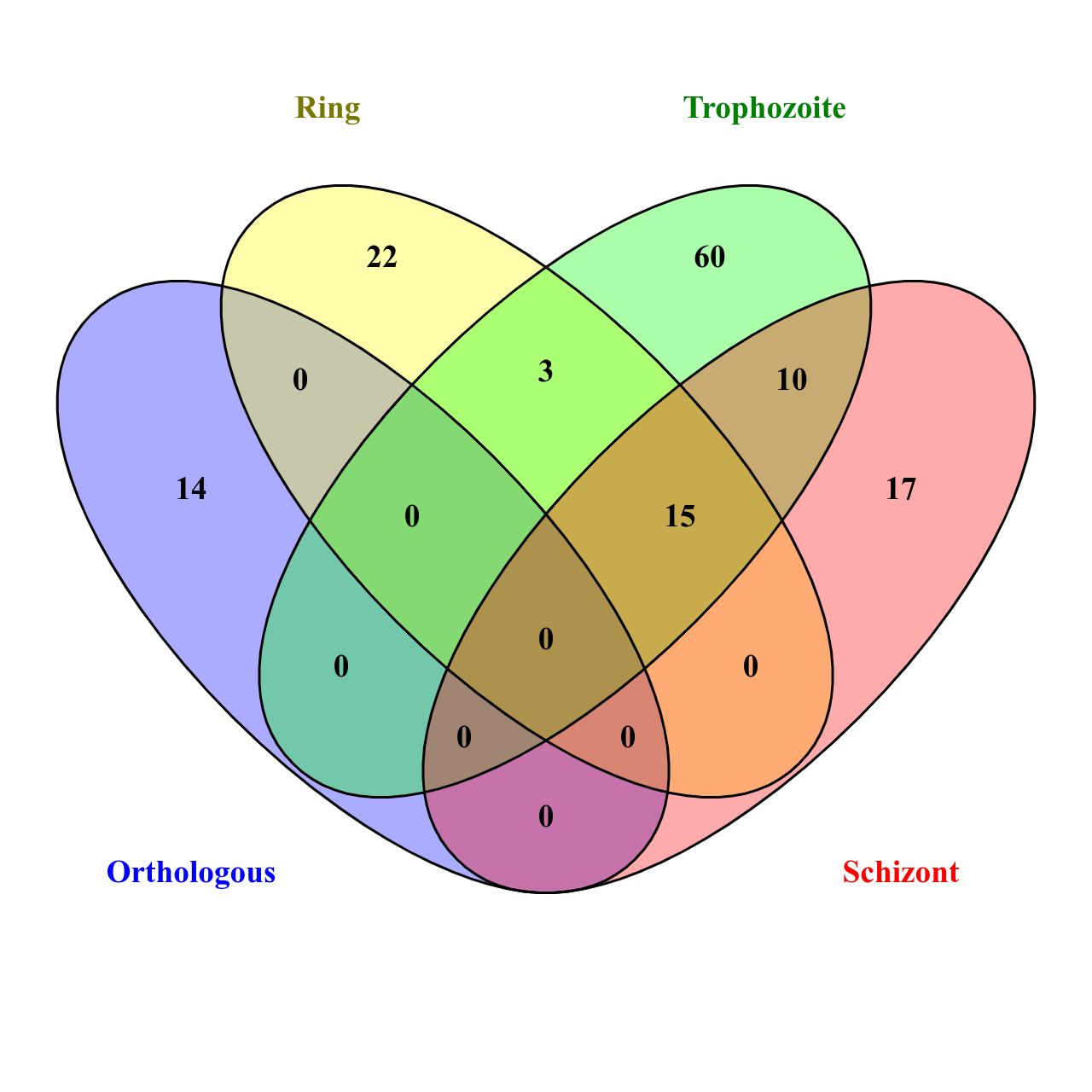

Supplement: Supplementary file 5 — Supplementary Information 5. [file 41598_2025_3586_MOESM5_ESM.zip › Supplementary File S6/Venn_unique14_orthologous.png]
