# Supplementary figures and images for: Transcriptome analysis reveals a de novo DNA element that may interact with chromatin-associated proteins in Plasmodium berghei during erythrocytic development (part 1 of 2)
Source: Sci Rep. 2025 May 28;15:18621. doi: 10.1038/s41598-025-03586-4 (PMC12120095; doi:10.1038/s41598-025-03586-4)

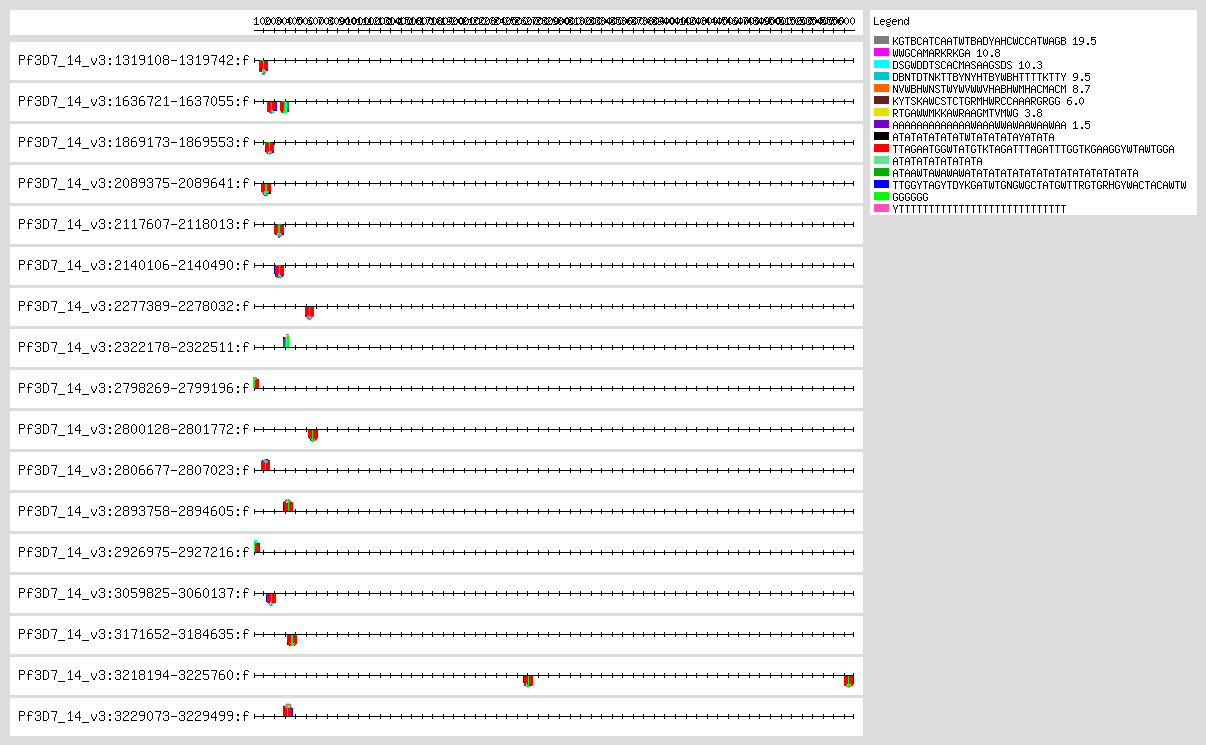

Supplement: Supplementary file 5 — Supplementary Information 5. [file 41598_2025_3586_MOESM5_ESM.zip › Supplementary File S6/rsat_chip_seq/DynSpansBySourceId_trophozoite_GCACTA.png]

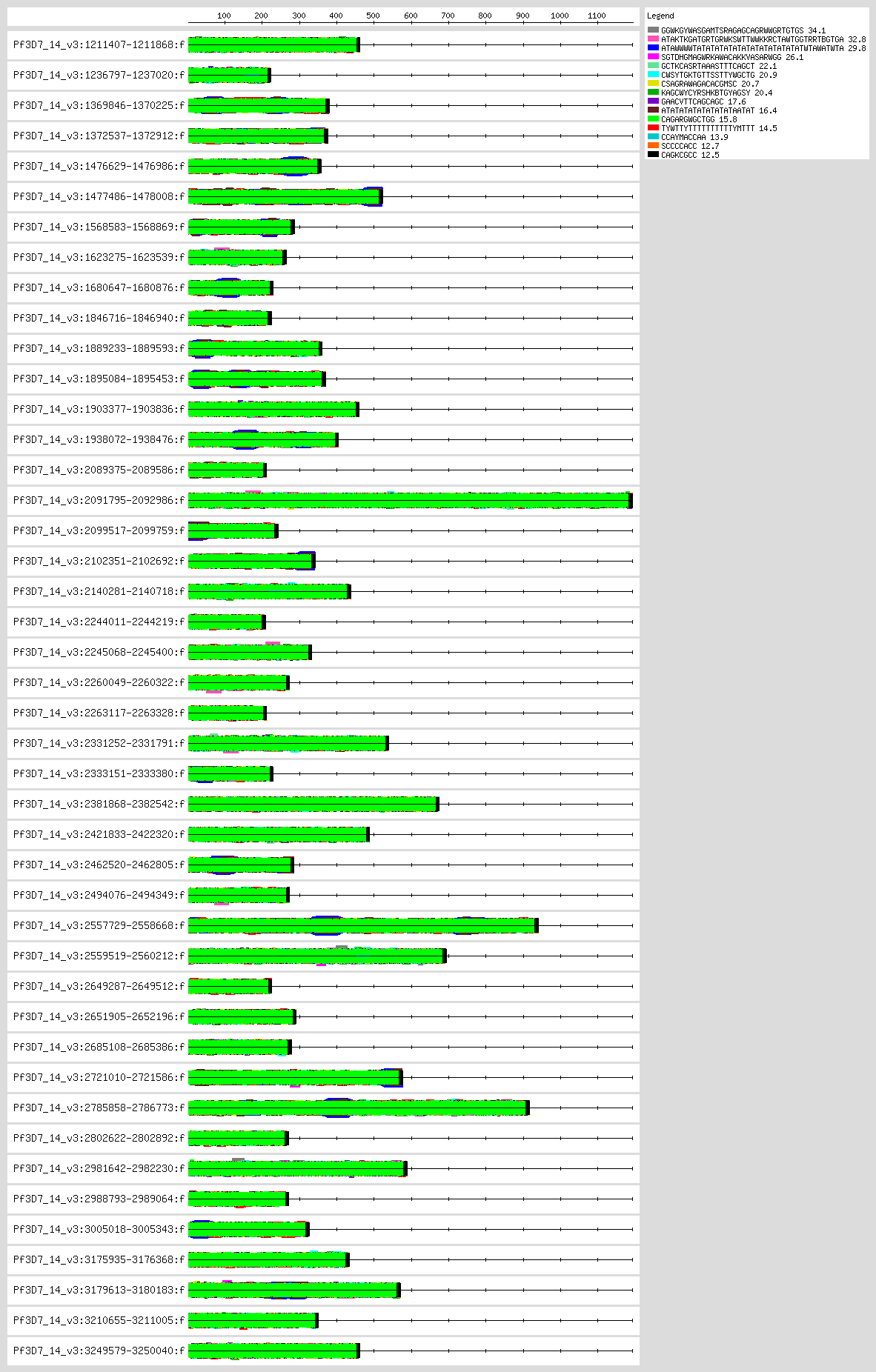

Supplement: Supplementary file 5 — Supplementary Information 5. [file 41598_2025_3586_MOESM5_ESM.zip › Supplementary File S6/rsat_chip_seq/DynSpansBySourceId_ring.png]

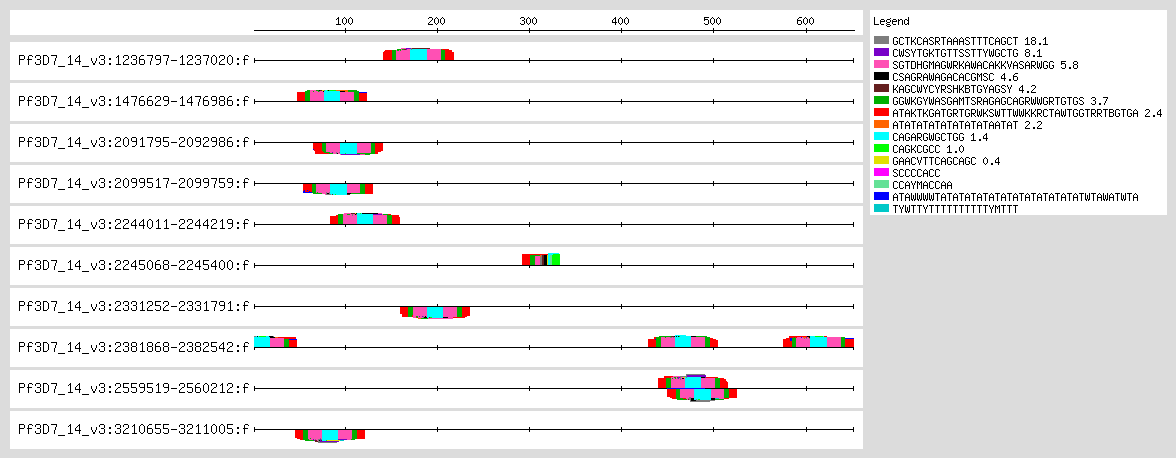

Supplement: Supplementary file 5 — Supplementary Information 5. [file 41598_2025_3586_MOESM5_ESM.zip › Supplementary File S6/rsat_chip_seq/DynSpansBySourceId_ring_AGGTAA.png]

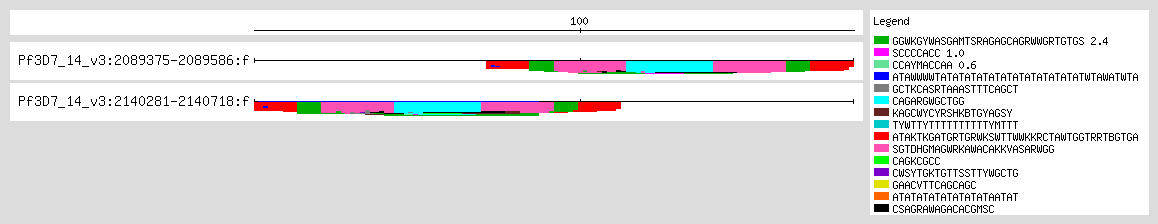

Supplement: Supplementary file 5 — Supplementary Information 5. [file 41598_2025_3586_MOESM5_ESM.zip › Supplementary File S6/rsat_chip_seq/DynSpansBySourceId_ring_GCACTA.png]

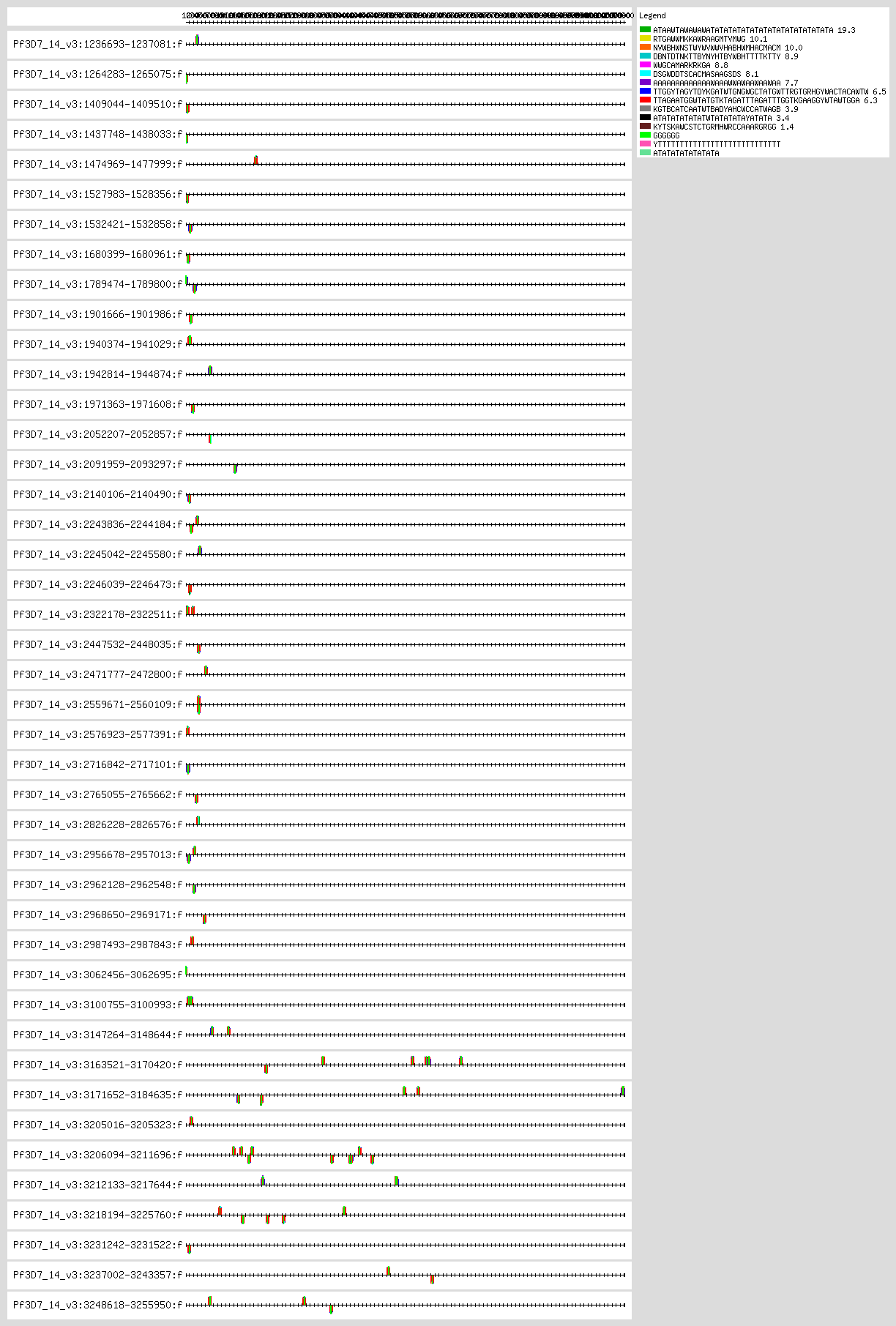

Supplement: Supplementary file 5 — Supplementary Information 5. [file 41598_2025_3586_MOESM5_ESM.zip › Supplementary File S6/rsat_chip_seq/DynSpansBySourceId_trophozoite_AGGTAA.png]

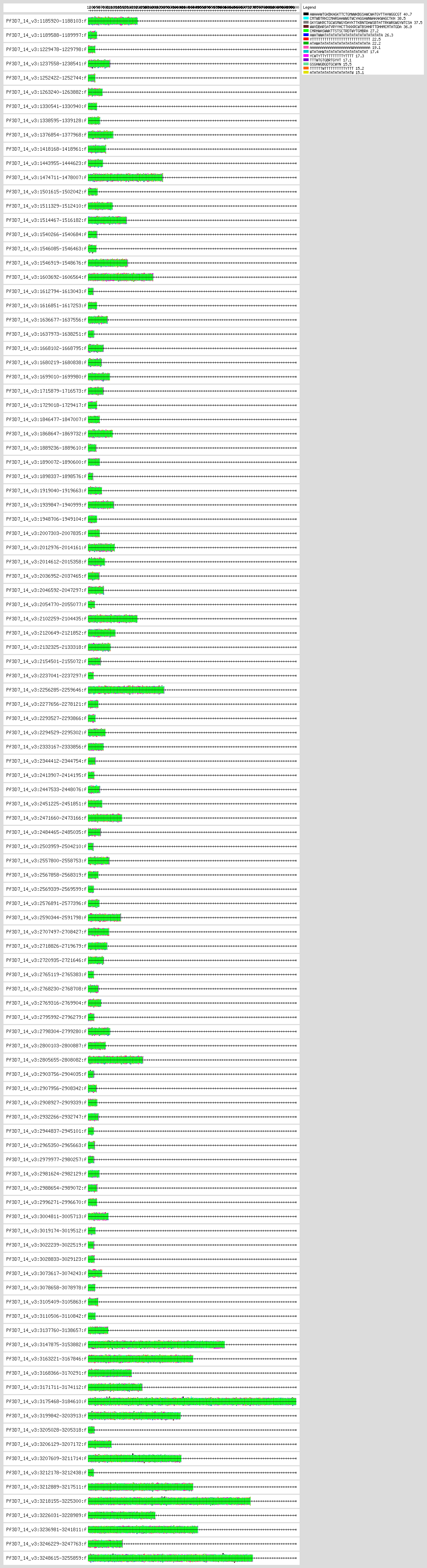

Supplement: Supplementary file 5 — Supplementary Information 5. [file 41598_2025_3586_MOESM5_ESM.zip › Supplementary File S6/rsat_chip_seq/DynSpansBySourceId_schizont.png]

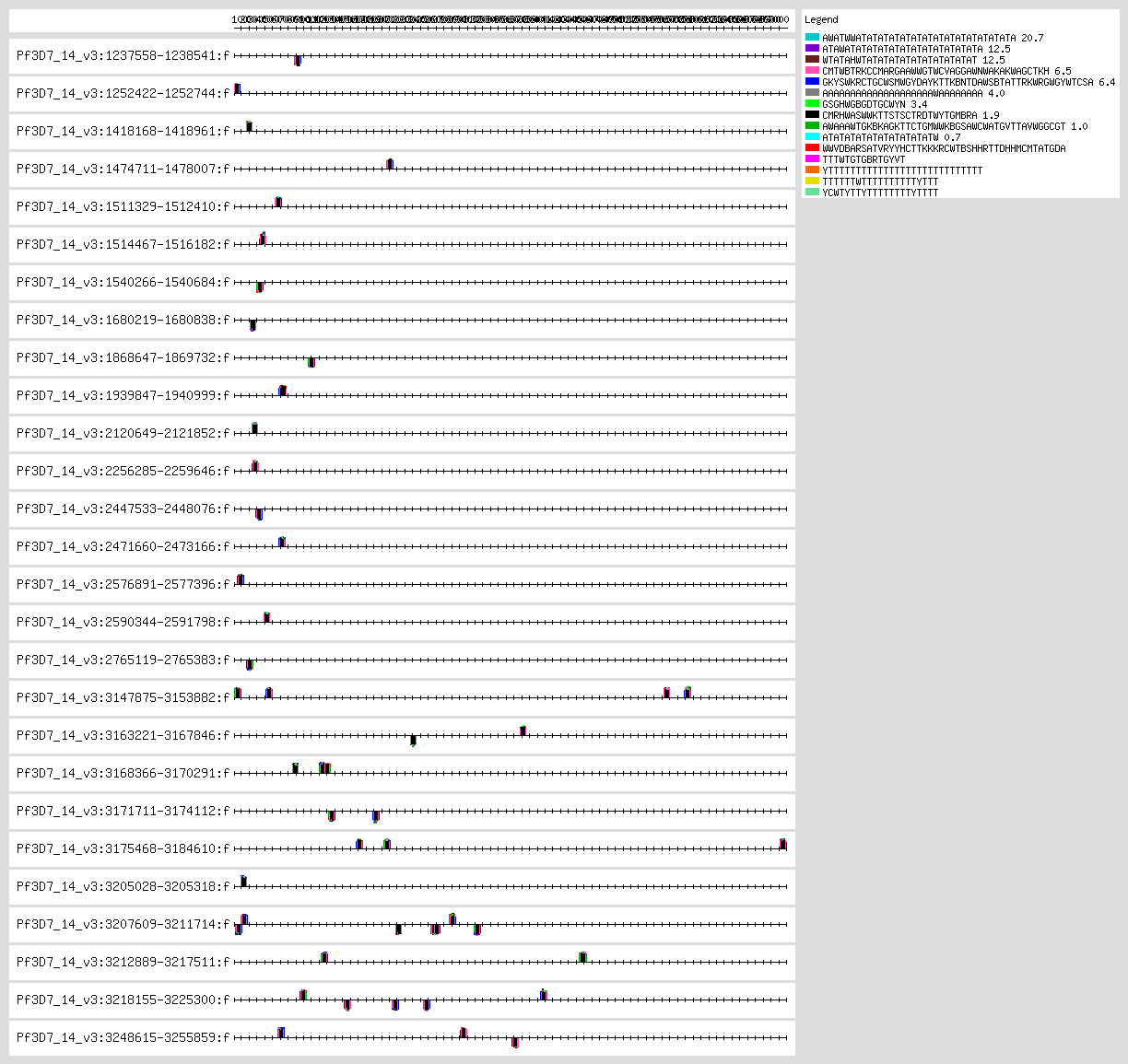

Supplement: Supplementary file 5 — Supplementary Information 5. [file 41598_2025_3586_MOESM5_ESM.zip › Supplementary File S6/rsat_chip_seq/DynSpansBySourceId_schizont_AGGTAA.png]

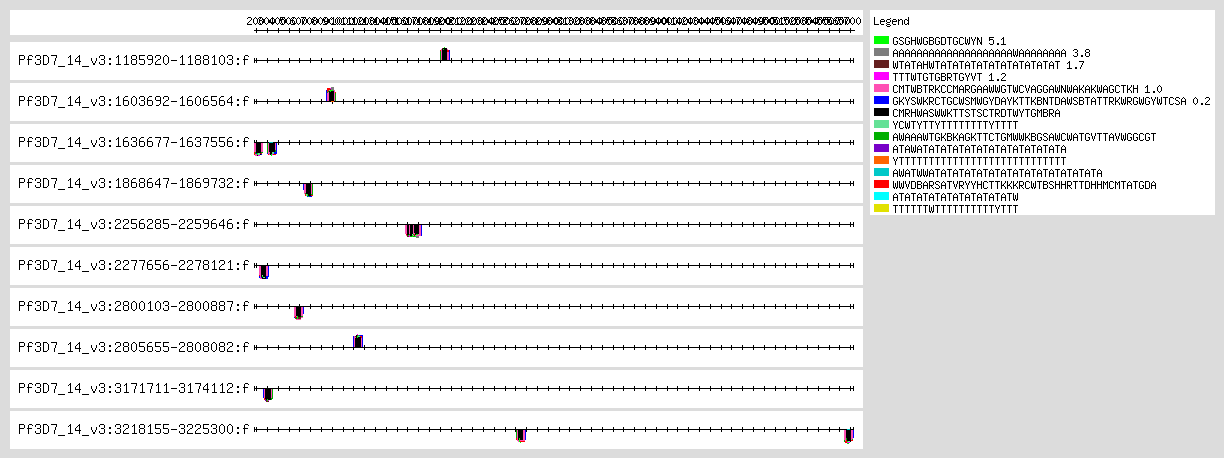

Supplement: Supplementary file 5 — Supplementary Information 5. [file 41598_2025_3586_MOESM5_ESM.zip › Supplementary File S6/rsat_chip_seq/DynSpansBySourceId_schizont_GCACTA.png]

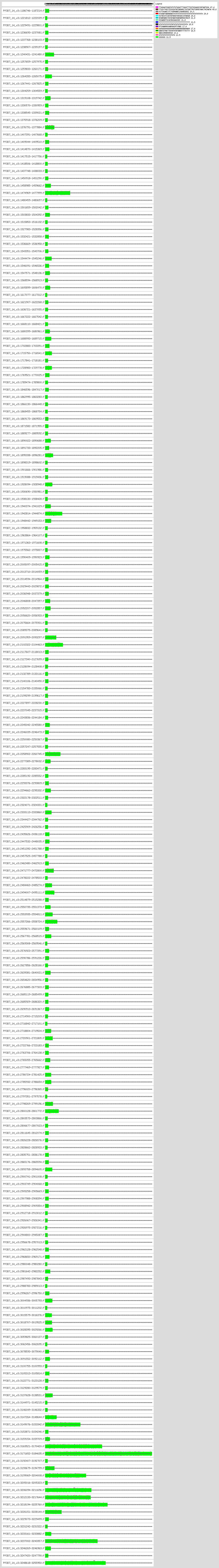

Supplement: Supplementary file 5 — Supplementary Information 5. [file 41598_2025_3586_MOESM5_ESM.zip › Supplementary File S6/rsat_chip_seq/DynSpansBySourceId_trophozoite.png]

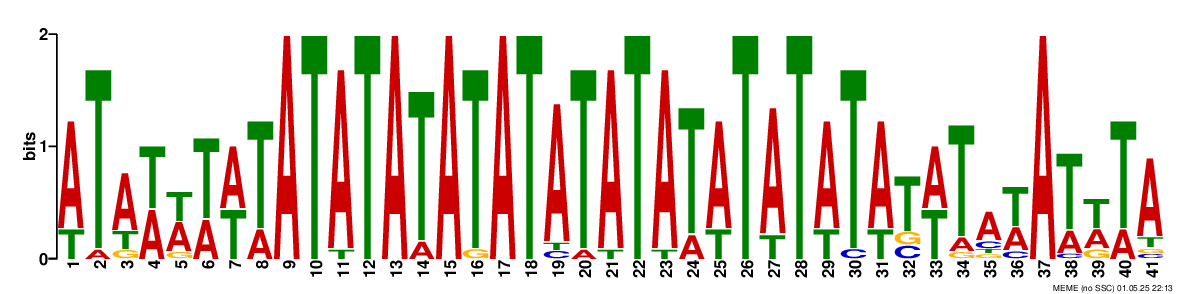

Supplement: Supplementary file 5 — Supplementary Information 5. [file 41598_2025_3586_MOESM5_ESM.zip › Supplementary File S6/meme_out_chip_seq/DynSpansBySourceId_ring/logo1.png]

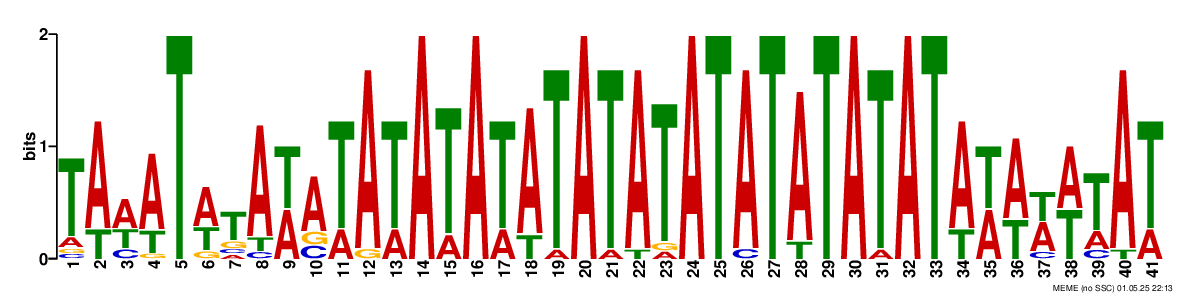

Supplement: Supplementary file 5 — Supplementary Information 5. [file 41598_2025_3586_MOESM5_ESM.zip › Supplementary File S6/meme_out_chip_seq/DynSpansBySourceId_ring/logo_rc1.png]

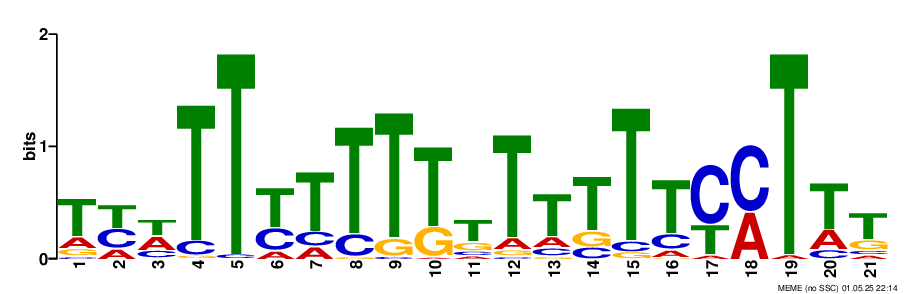

Supplement: Supplementary file 5 — Supplementary Information 5. [file 41598_2025_3586_MOESM5_ESM.zip › Supplementary File S6/meme_out_chip_seq/DynSpansBySourceId_ring/logo2.png]

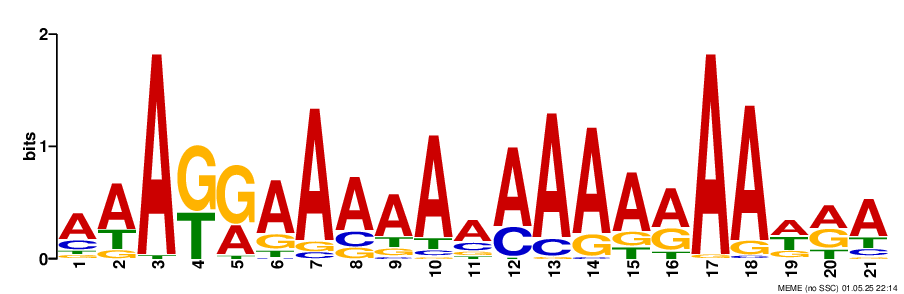

Supplement: Supplementary file 5 — Supplementary Information 5. [file 41598_2025_3586_MOESM5_ESM.zip › Supplementary File S6/meme_out_chip_seq/DynSpansBySourceId_ring/logo_rc2.png]

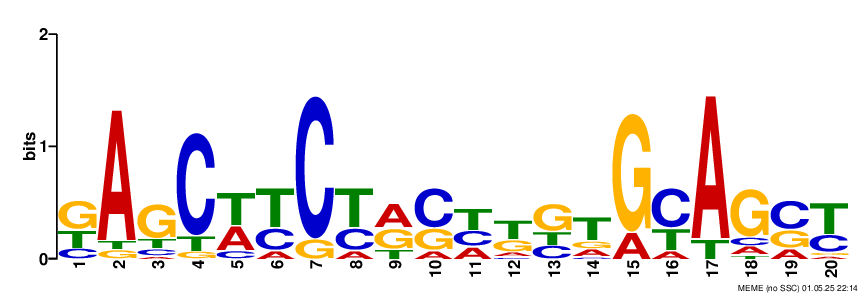

Supplement: Supplementary file 5 — Supplementary Information 5. [file 41598_2025_3586_MOESM5_ESM.zip › Supplementary File S6/meme_out_chip_seq/DynSpansBySourceId_ring/logo3.png]

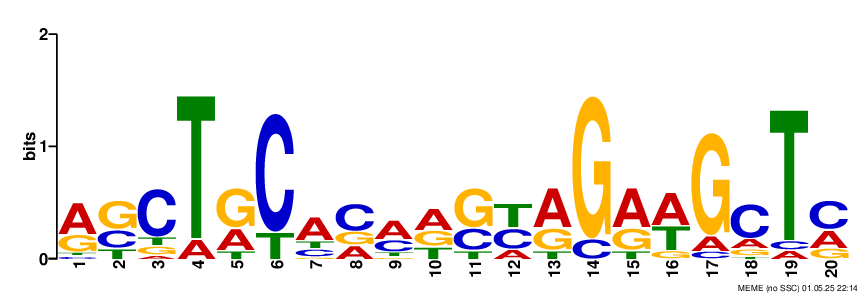

Supplement: Supplementary file 5 — Supplementary Information 5. [file 41598_2025_3586_MOESM5_ESM.zip › Supplementary File S6/meme_out_chip_seq/DynSpansBySourceId_ring/logo_rc3.png]

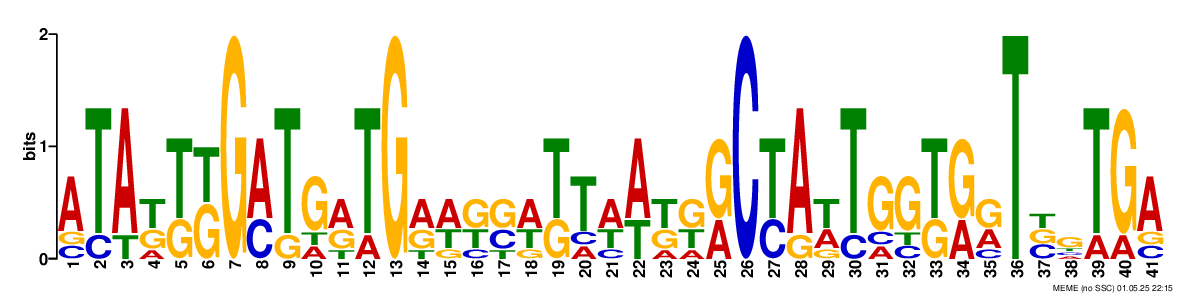

Supplement: Supplementary file 5 — Supplementary Information 5. [file 41598_2025_3586_MOESM5_ESM.zip › Supplementary File S6/meme_out_chip_seq/DynSpansBySourceId_ring/logo4.png]

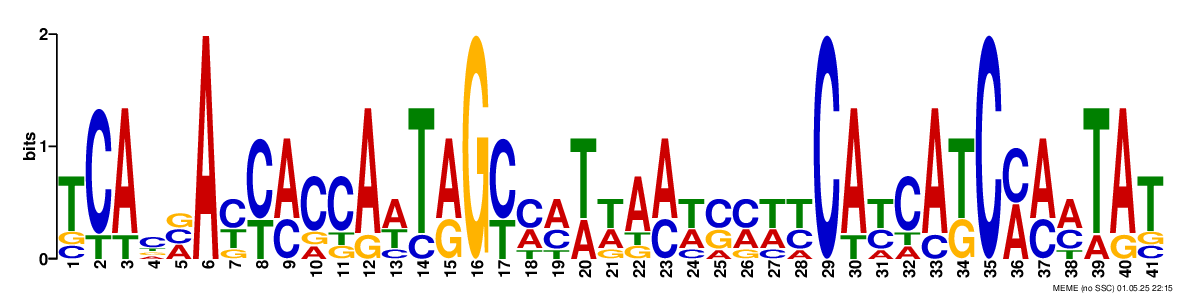

Supplement: Supplementary file 5 — Supplementary Information 5. [file 41598_2025_3586_MOESM5_ESM.zip › Supplementary File S6/meme_out_chip_seq/DynSpansBySourceId_ring/logo_rc4.png]

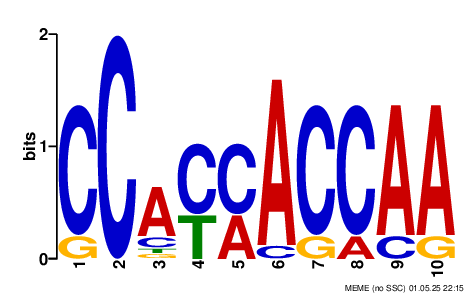

Supplement: Supplementary file 5 — Supplementary Information 5. [file 41598_2025_3586_MOESM5_ESM.zip › Supplementary File S6/meme_out_chip_seq/DynSpansBySourceId_ring/logo5.png]

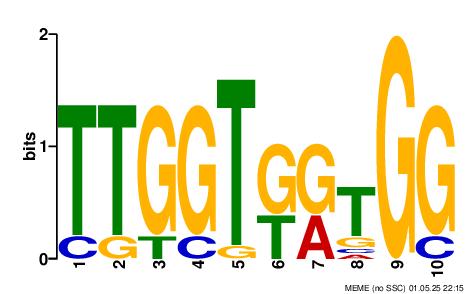

Supplement: Supplementary file 5 — Supplementary Information 5. [file 41598_2025_3586_MOESM5_ESM.zip › Supplementary File S6/meme_out_chip_seq/DynSpansBySourceId_ring/logo_rc5.png]

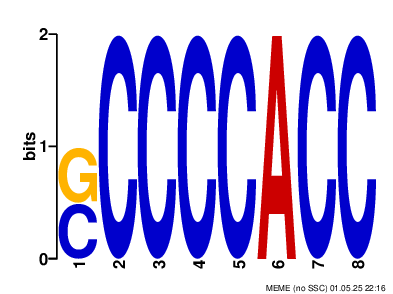

Supplement: Supplementary file 5 — Supplementary Information 5. [file 41598_2025_3586_MOESM5_ESM.zip › Supplementary File S6/meme_out_chip_seq/DynSpansBySourceId_ring/logo6.png]

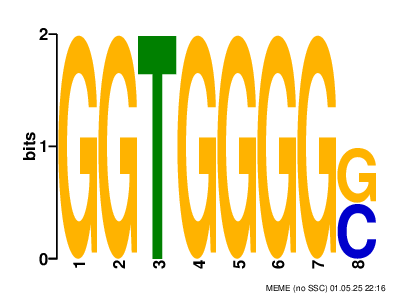

Supplement: Supplementary file 5 — Supplementary Information 5. [file 41598_2025_3586_MOESM5_ESM.zip › Supplementary File S6/meme_out_chip_seq/DynSpansBySourceId_ring/logo_rc6.png]

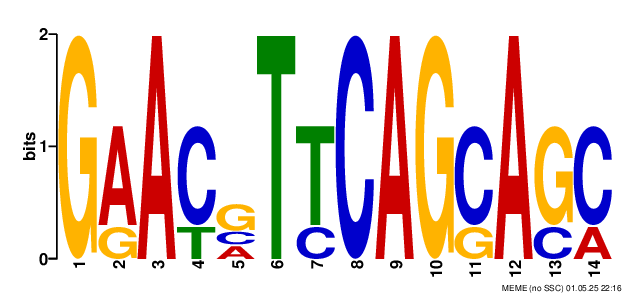

Supplement: Supplementary file 5 — Supplementary Information 5. [file 41598_2025_3586_MOESM5_ESM.zip › Supplementary File S6/meme_out_chip_seq/DynSpansBySourceId_ring/logo7.png]

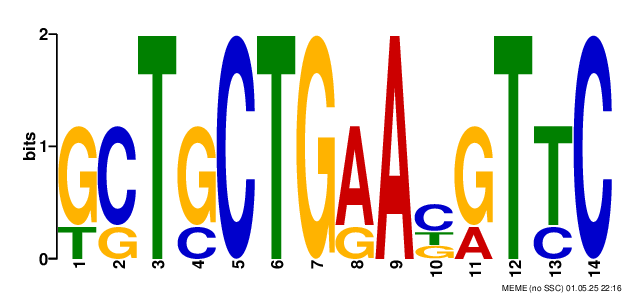

Supplement: Supplementary file 5 — Supplementary Information 5. [file 41598_2025_3586_MOESM5_ESM.zip › Supplementary File S6/meme_out_chip_seq/DynSpansBySourceId_ring/logo_rc7.png]

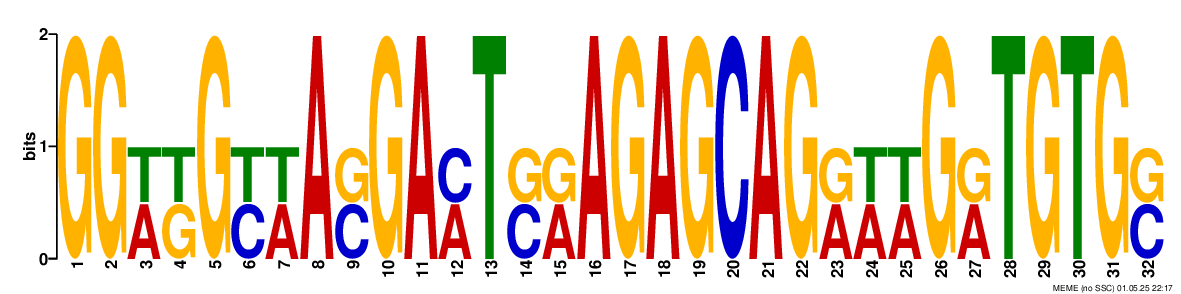

Supplement: Supplementary file 5 — Supplementary Information 5. [file 41598_2025_3586_MOESM5_ESM.zip › Supplementary File S6/meme_out_chip_seq/DynSpansBySourceId_ring/logo8.png]

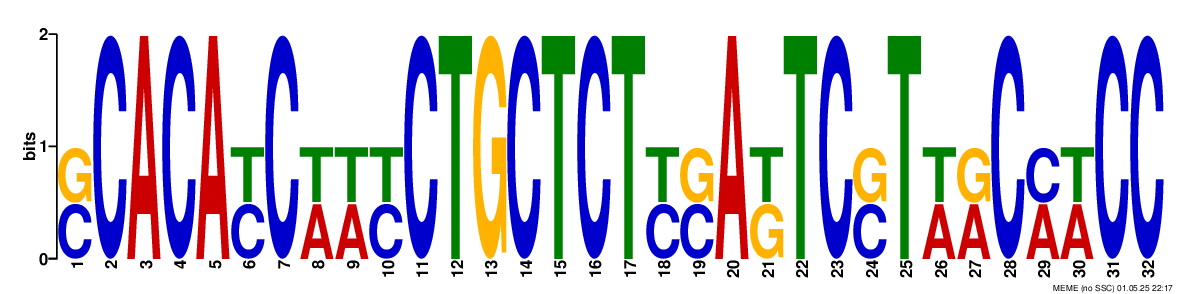

Supplement: Supplementary file 5 — Supplementary Information 5. [file 41598_2025_3586_MOESM5_ESM.zip › Supplementary File S6/meme_out_chip_seq/DynSpansBySourceId_ring/logo_rc8.png]

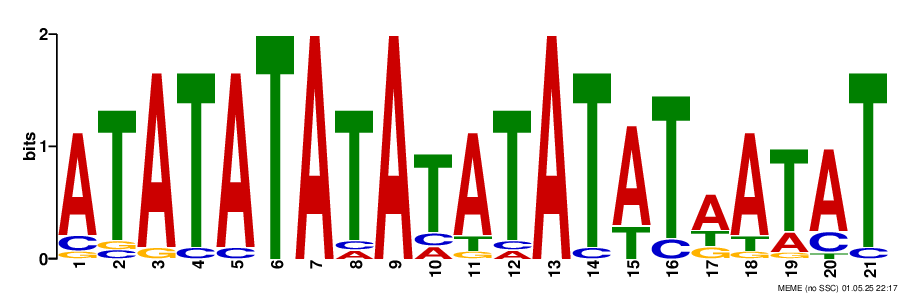

Supplement: Supplementary file 5 — Supplementary Information 5. [file 41598_2025_3586_MOESM5_ESM.zip › Supplementary File S6/meme_out_chip_seq/DynSpansBySourceId_ring/logo9.png]

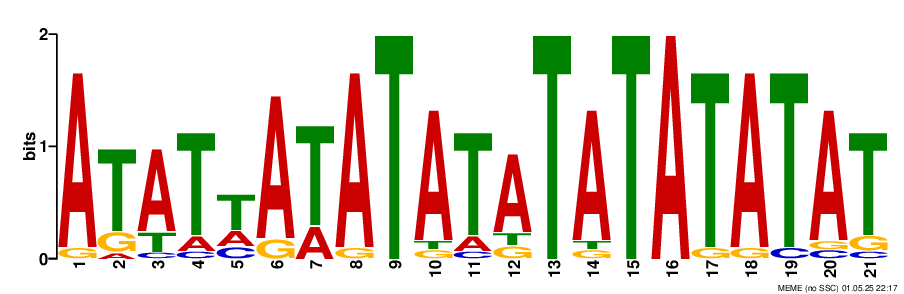

Supplement: Supplementary file 5 — Supplementary Information 5. [file 41598_2025_3586_MOESM5_ESM.zip › Supplementary File S6/meme_out_chip_seq/DynSpansBySourceId_ring/logo_rc9.png]

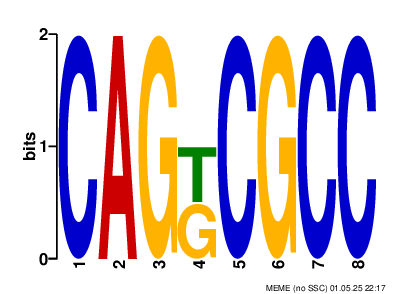

Supplement: Supplementary file 5 — Supplementary Information 5. [file 41598_2025_3586_MOESM5_ESM.zip › Supplementary File S6/meme_out_chip_seq/DynSpansBySourceId_ring/logo10.png]

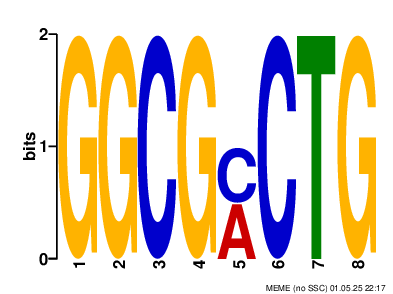

Supplement: Supplementary file 5 — Supplementary Information 5. [file 41598_2025_3586_MOESM5_ESM.zip › Supplementary File S6/meme_out_chip_seq/DynSpansBySourceId_ring/logo_rc10.png]

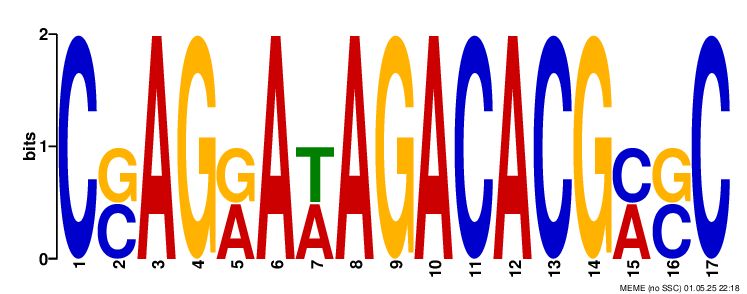

Supplement: Supplementary file 5 — Supplementary Information 5. [file 41598_2025_3586_MOESM5_ESM.zip › Supplementary File S6/meme_out_chip_seq/DynSpansBySourceId_ring/logo11.png]

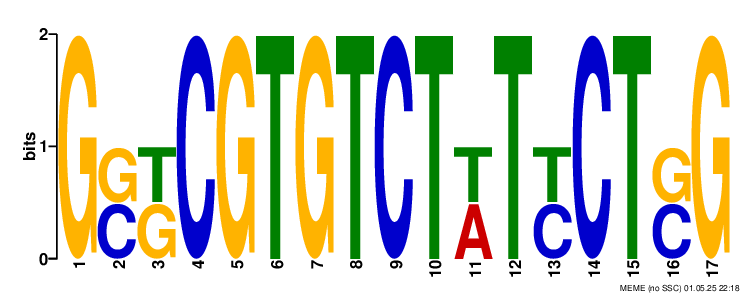

Supplement: Supplementary file 5 — Supplementary Information 5. [file 41598_2025_3586_MOESM5_ESM.zip › Supplementary File S6/meme_out_chip_seq/DynSpansBySourceId_ring/logo_rc11.png]

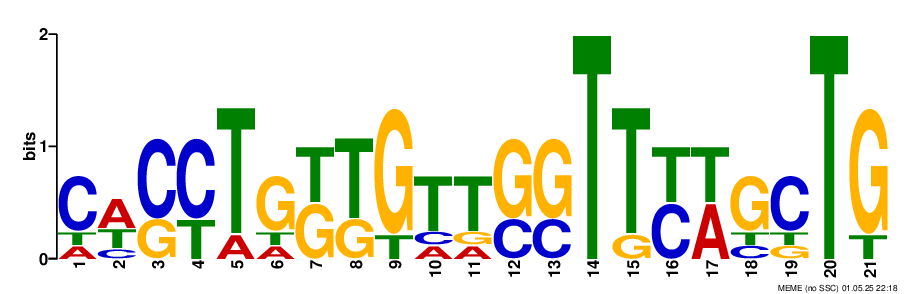

Supplement: Supplementary file 5 — Supplementary Information 5. [file 41598_2025_3586_MOESM5_ESM.zip › Supplementary File S6/meme_out_chip_seq/DynSpansBySourceId_ring/logo12.png]

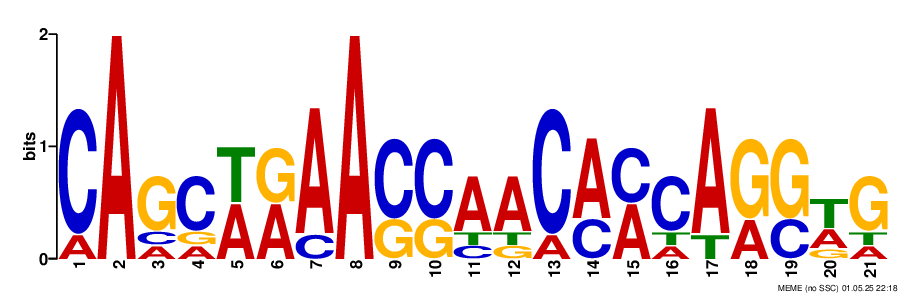

Supplement: Supplementary file 5 — Supplementary Information 5. [file 41598_2025_3586_MOESM5_ESM.zip › Supplementary File S6/meme_out_chip_seq/DynSpansBySourceId_ring/logo_rc12.png]

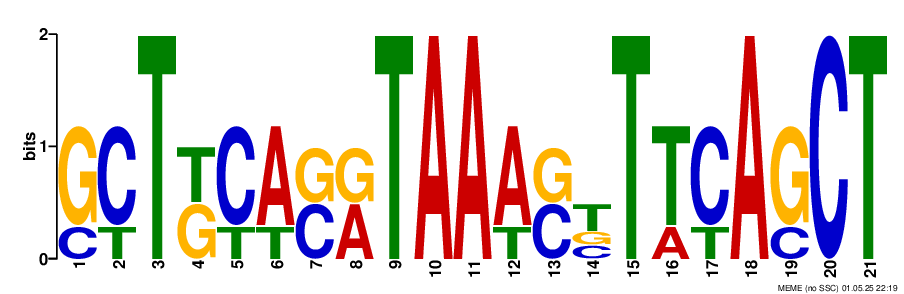

Supplement: Supplementary file 5 — Supplementary Information 5. [file 41598_2025_3586_MOESM5_ESM.zip › Supplementary File S6/meme_out_chip_seq/DynSpansBySourceId_ring/logo13.png]

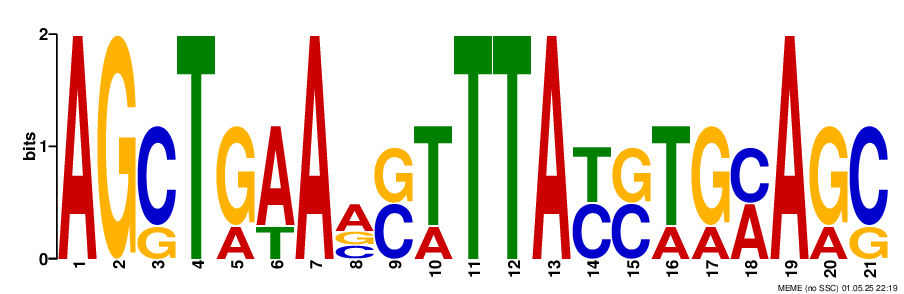

Supplement: Supplementary file 5 — Supplementary Information 5. [file 41598_2025_3586_MOESM5_ESM.zip › Supplementary File S6/meme_out_chip_seq/DynSpansBySourceId_ring/logo_rc13.png]

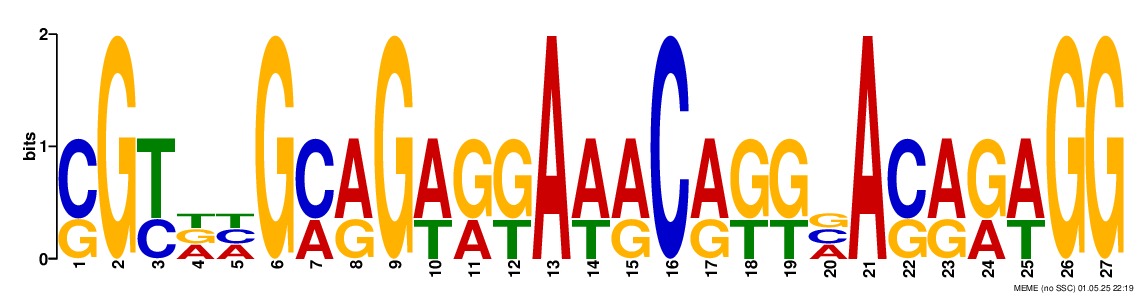

Supplement: Supplementary file 5 — Supplementary Information 5. [file 41598_2025_3586_MOESM5_ESM.zip › Supplementary File S6/meme_out_chip_seq/DynSpansBySourceId_ring/logo14.png]

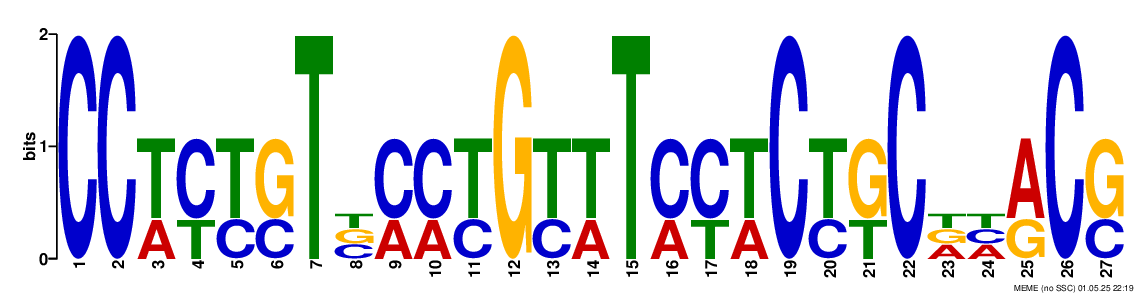

Supplement: Supplementary file 5 — Supplementary Information 5. [file 41598_2025_3586_MOESM5_ESM.zip › Supplementary File S6/meme_out_chip_seq/DynSpansBySourceId_ring/logo_rc14.png]

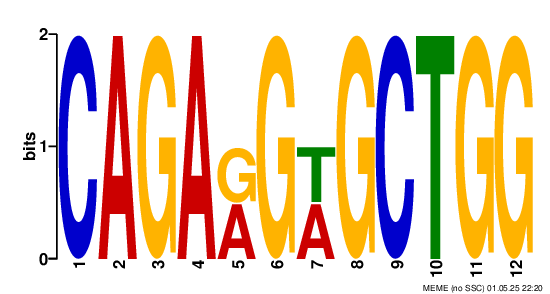

Supplement: Supplementary file 5 — Supplementary Information 5. [file 41598_2025_3586_MOESM5_ESM.zip › Supplementary File S6/meme_out_chip_seq/DynSpansBySourceId_ring/logo15.png]

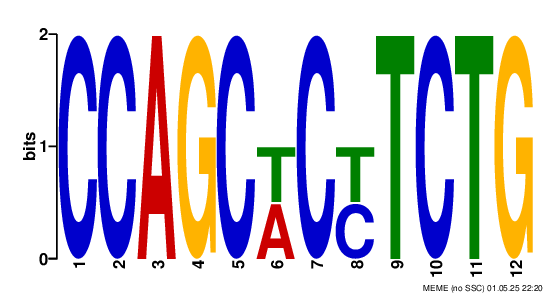

Supplement: Supplementary file 5 — Supplementary Information 5. [file 41598_2025_3586_MOESM5_ESM.zip › Supplementary File S6/meme_out_chip_seq/DynSpansBySourceId_ring/logo_rc15.png]

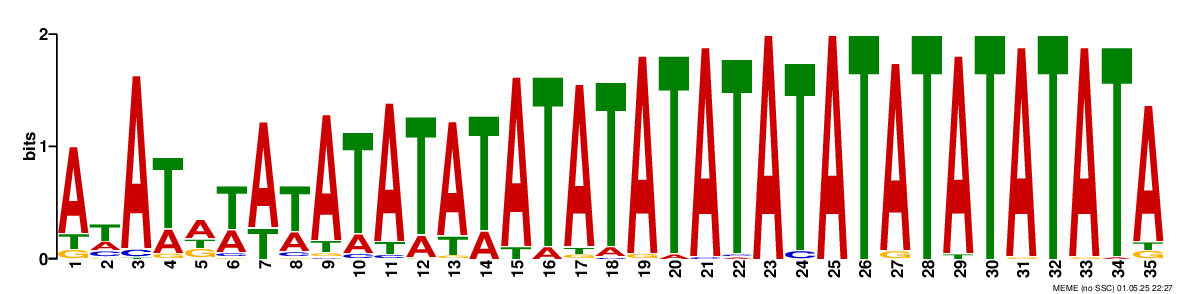

Supplement: Supplementary file 5 — Supplementary Information 5. [file 41598_2025_3586_MOESM5_ESM.zip › Supplementary File S6/meme_out_chip_seq/DynSpansBySourceId_schizont/logo1.png]

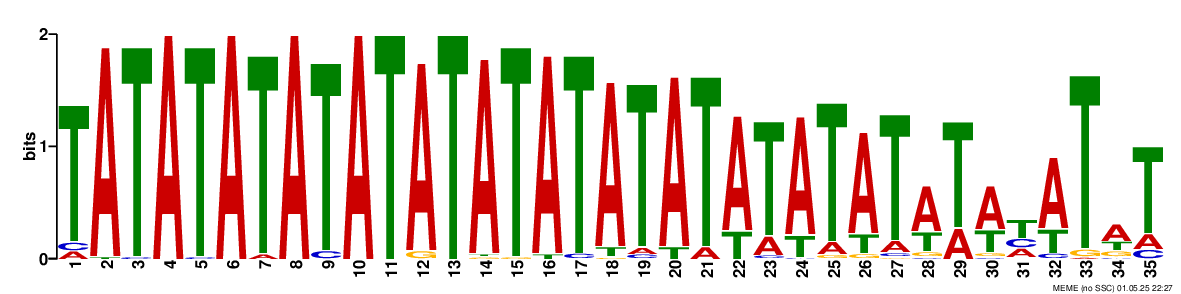

Supplement: Supplementary file 5 — Supplementary Information 5. [file 41598_2025_3586_MOESM5_ESM.zip › Supplementary File S6/meme_out_chip_seq/DynSpansBySourceId_schizont/logo_rc1.png]

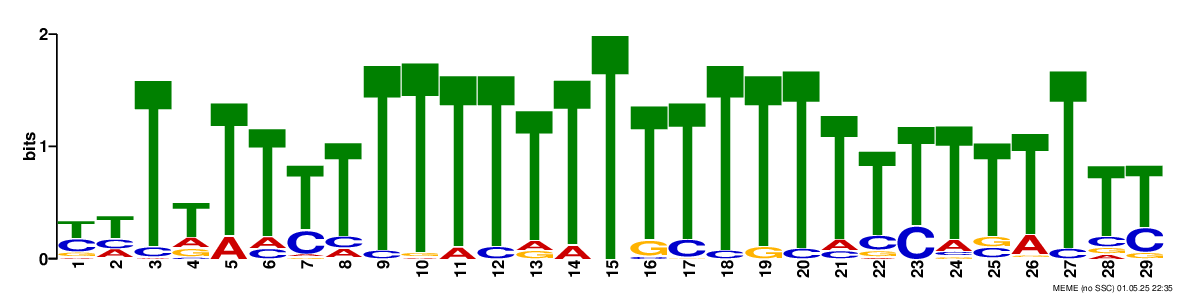

Supplement: Supplementary file 5 — Supplementary Information 5. [file 41598_2025_3586_MOESM5_ESM.zip › Supplementary File S6/meme_out_chip_seq/DynSpansBySourceId_schizont/logo2.png]

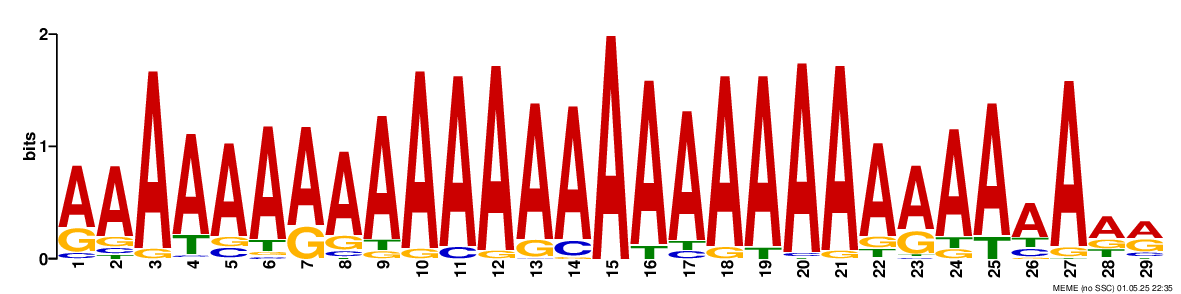

Supplement: Supplementary file 5 — Supplementary Information 5. [file 41598_2025_3586_MOESM5_ESM.zip › Supplementary File S6/meme_out_chip_seq/DynSpansBySourceId_schizont/logo_rc2.png]

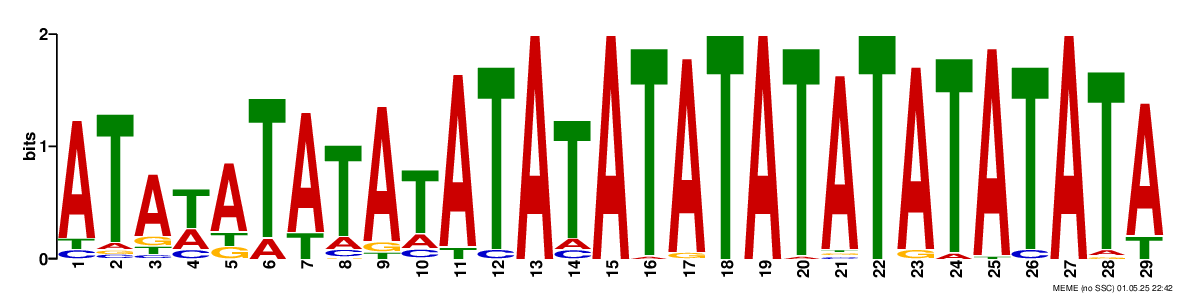

Supplement: Supplementary file 5 — Supplementary Information 5. [file 41598_2025_3586_MOESM5_ESM.zip › Supplementary File S6/meme_out_chip_seq/DynSpansBySourceId_schizont/logo3.png]

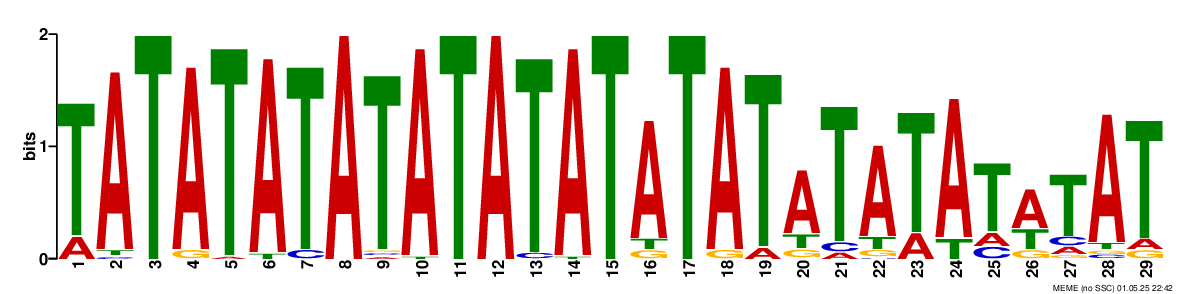

Supplement: Supplementary file 5 — Supplementary Information 5. [file 41598_2025_3586_MOESM5_ESM.zip › Supplementary File S6/meme_out_chip_seq/DynSpansBySourceId_schizont/logo_rc3.png]

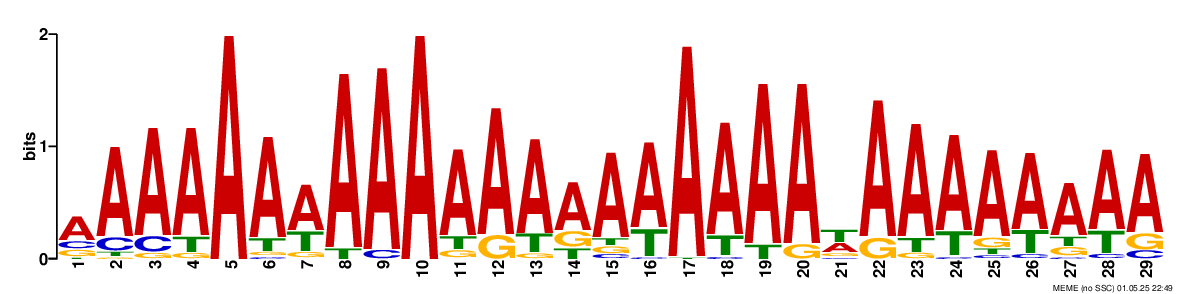

Supplement: Supplementary file 5 — Supplementary Information 5. [file 41598_2025_3586_MOESM5_ESM.zip › Supplementary File S6/meme_out_chip_seq/DynSpansBySourceId_schizont/logo4.png]

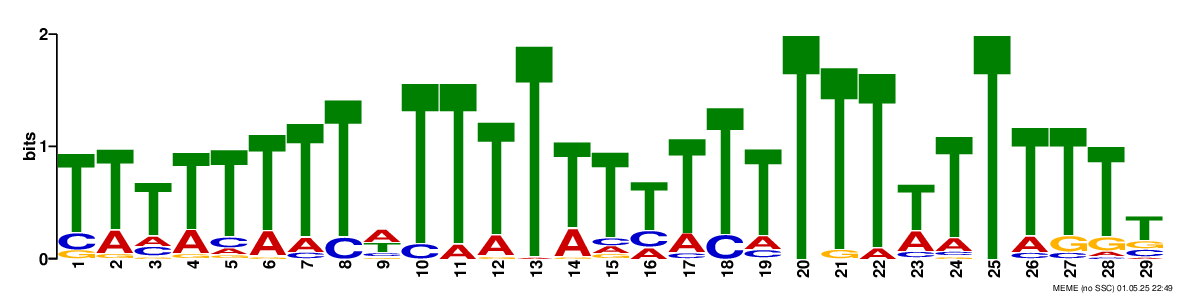

Supplement: Supplementary file 5 — Supplementary Information 5. [file 41598_2025_3586_MOESM5_ESM.zip › Supplementary File S6/meme_out_chip_seq/DynSpansBySourceId_schizont/logo_rc4.png]

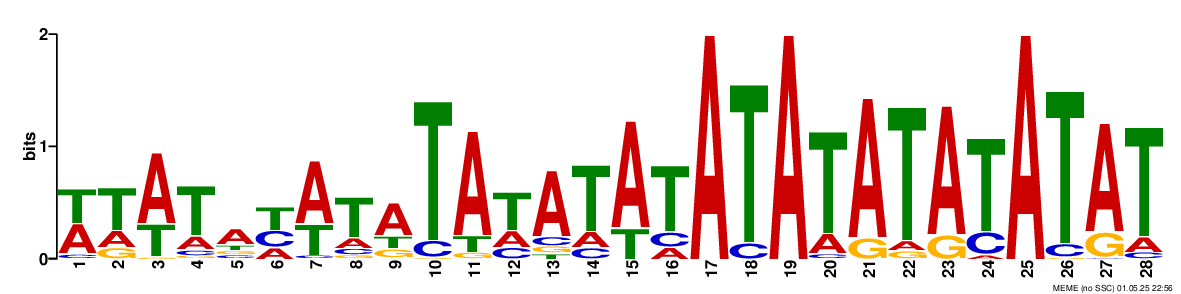

Supplement: Supplementary file 5 — Supplementary Information 5. [file 41598_2025_3586_MOESM5_ESM.zip › Supplementary File S6/meme_out_chip_seq/DynSpansBySourceId_schizont/logo5.png]

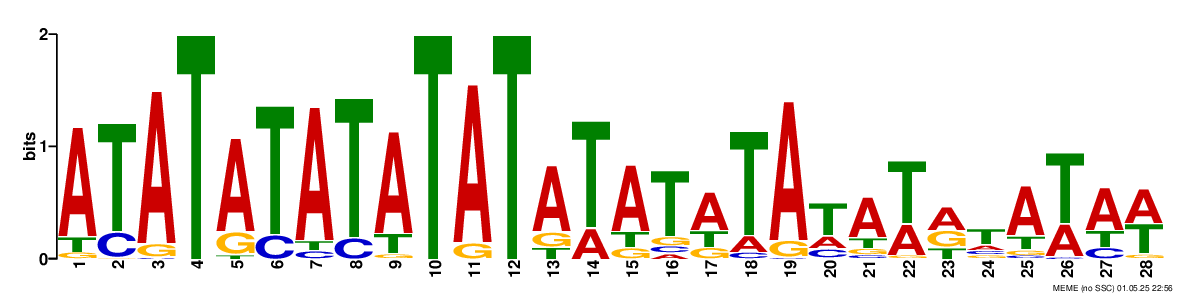

Supplement: Supplementary file 5 — Supplementary Information 5. [file 41598_2025_3586_MOESM5_ESM.zip › Supplementary File S6/meme_out_chip_seq/DynSpansBySourceId_schizont/logo_rc5.png]

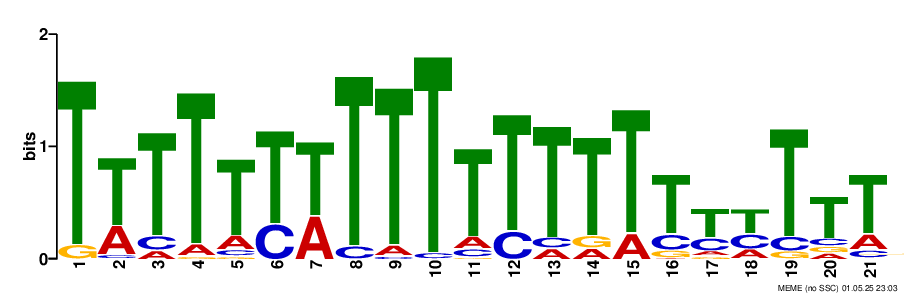

Supplement: Supplementary file 5 — Supplementary Information 5. [file 41598_2025_3586_MOESM5_ESM.zip › Supplementary File S6/meme_out_chip_seq/DynSpansBySourceId_schizont/logo6.png]

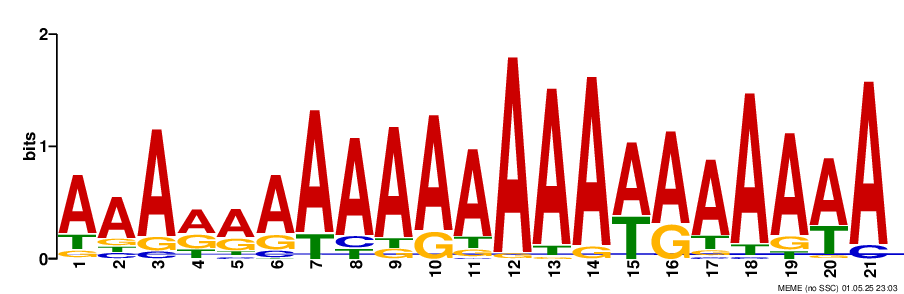

Supplement: Supplementary file 5 — Supplementary Information 5. [file 41598_2025_3586_MOESM5_ESM.zip › Supplementary File S6/meme_out_chip_seq/DynSpansBySourceId_schizont/logo_rc6.png]

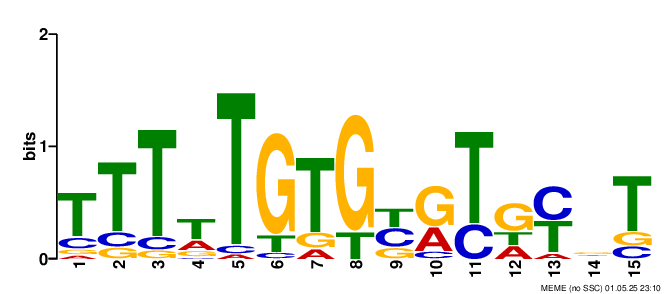

Supplement: Supplementary file 5 — Supplementary Information 5. [file 41598_2025_3586_MOESM5_ESM.zip › Supplementary File S6/meme_out_chip_seq/DynSpansBySourceId_schizont/logo7.png]

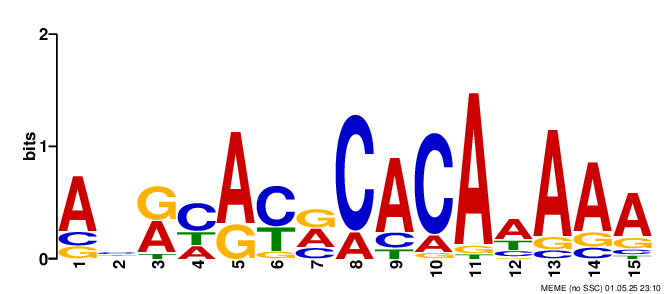

Supplement: Supplementary file 5 — Supplementary Information 5. [file 41598_2025_3586_MOESM5_ESM.zip › Supplementary File S6/meme_out_chip_seq/DynSpansBySourceId_schizont/logo_rc7.png]

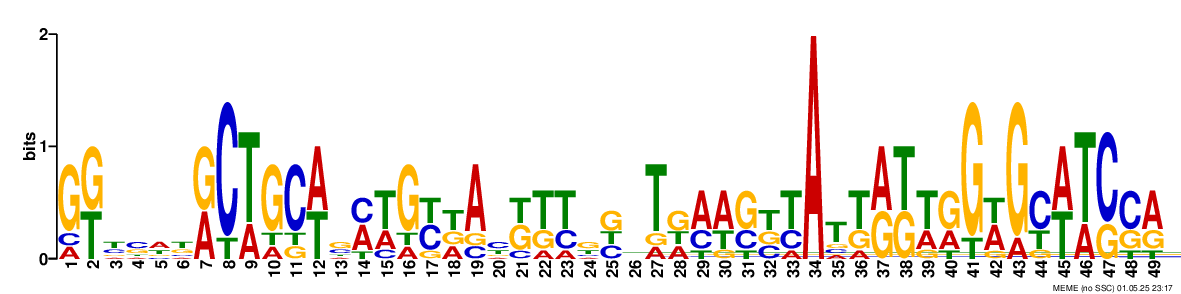

Supplement: Supplementary file 5 — Supplementary Information 5. [file 41598_2025_3586_MOESM5_ESM.zip › Supplementary File S6/meme_out_chip_seq/DynSpansBySourceId_schizont/logo8.png]

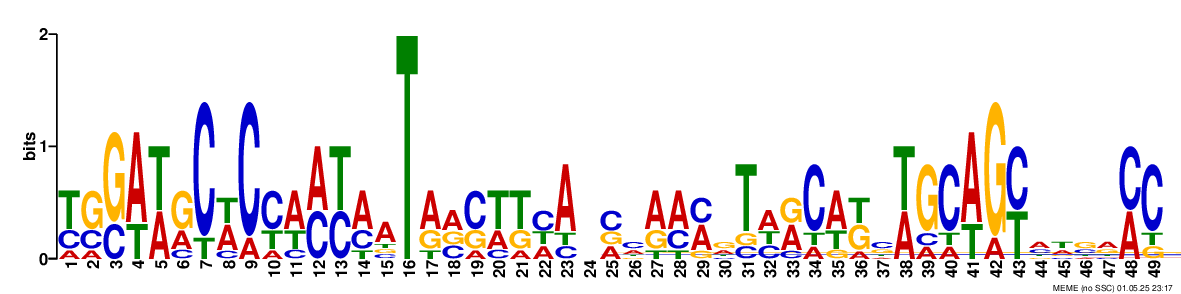

Supplement: Supplementary file 5 — Supplementary Information 5. [file 41598_2025_3586_MOESM5_ESM.zip › Supplementary File S6/meme_out_chip_seq/DynSpansBySourceId_schizont/logo_rc8.png]

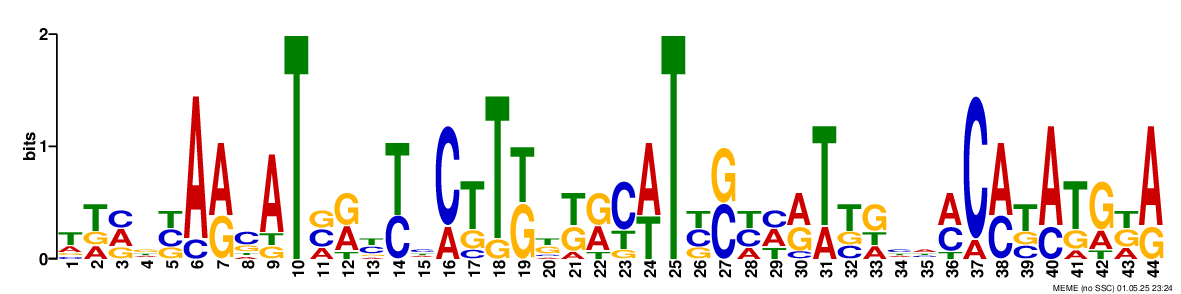

Supplement: Supplementary file 5 — Supplementary Information 5. [file 41598_2025_3586_MOESM5_ESM.zip › Supplementary File S6/meme_out_chip_seq/DynSpansBySourceId_schizont/logo9.png]

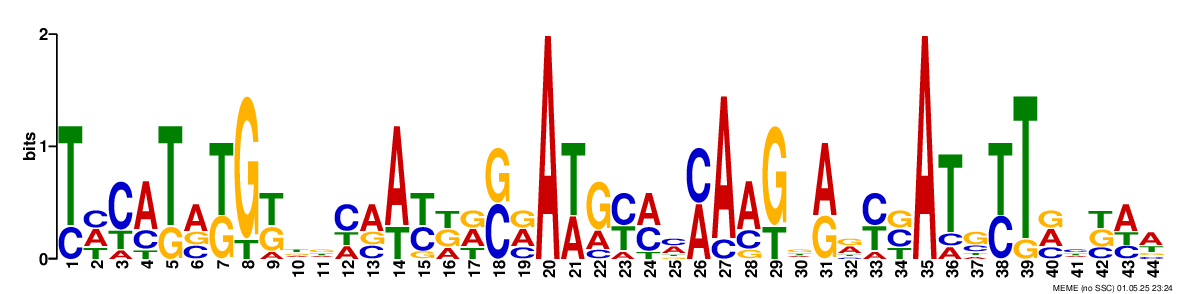

Supplement: Supplementary file 5 — Supplementary Information 5. [file 41598_2025_3586_MOESM5_ESM.zip › Supplementary File S6/meme_out_chip_seq/DynSpansBySourceId_schizont/logo_rc9.png]

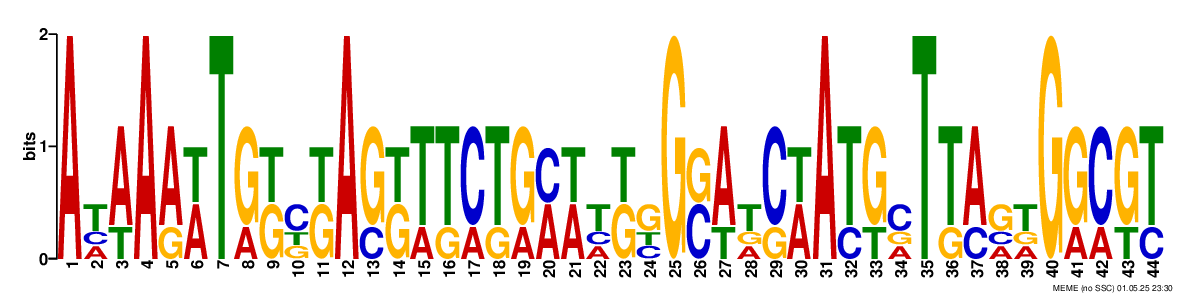

Supplement: Supplementary file 5 — Supplementary Information 5. [file 41598_2025_3586_MOESM5_ESM.zip › Supplementary File S6/meme_out_chip_seq/DynSpansBySourceId_schizont/logo10.png]

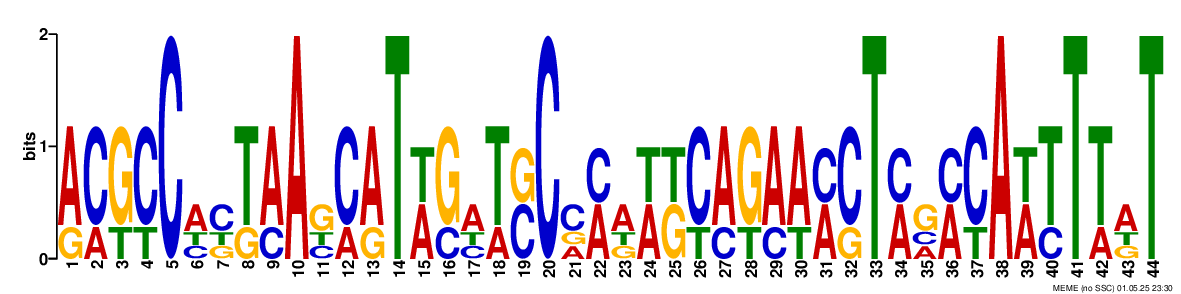

Supplement: Supplementary file 5 — Supplementary Information 5. [file 41598_2025_3586_MOESM5_ESM.zip › Supplementary File S6/meme_out_chip_seq/DynSpansBySourceId_schizont/logo_rc10.png]

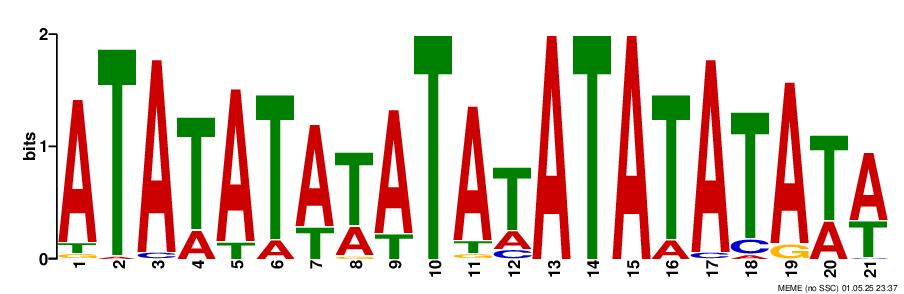

Supplement: Supplementary file 5 — Supplementary Information 5. [file 41598_2025_3586_MOESM5_ESM.zip › Supplementary File S6/meme_out_chip_seq/DynSpansBySourceId_schizont/logo11.png]

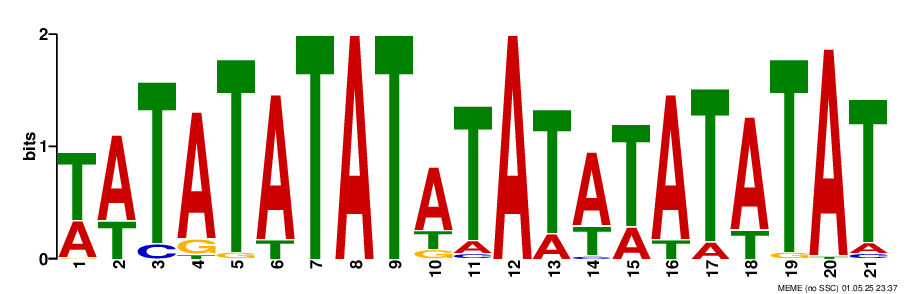

Supplement: Supplementary file 5 — Supplementary Information 5. [file 41598_2025_3586_MOESM5_ESM.zip › Supplementary File S6/meme_out_chip_seq/DynSpansBySourceId_schizont/logo_rc11.png]

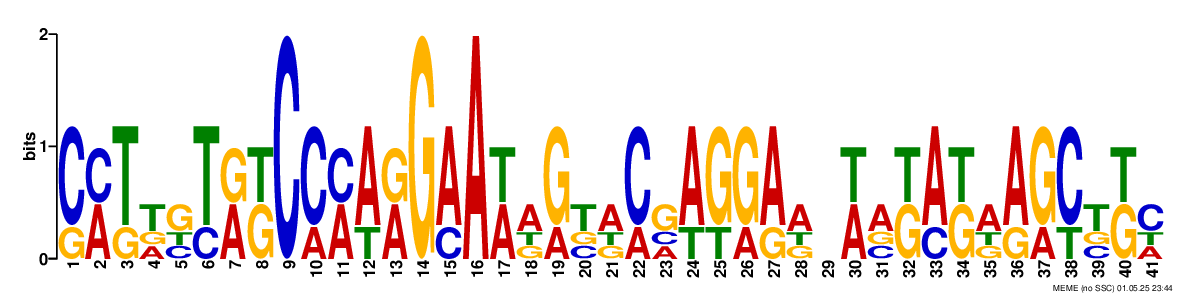

Supplement: Supplementary file 5 — Supplementary Information 5. [file 41598_2025_3586_MOESM5_ESM.zip › Supplementary File S6/meme_out_chip_seq/DynSpansBySourceId_schizont/logo12.png]

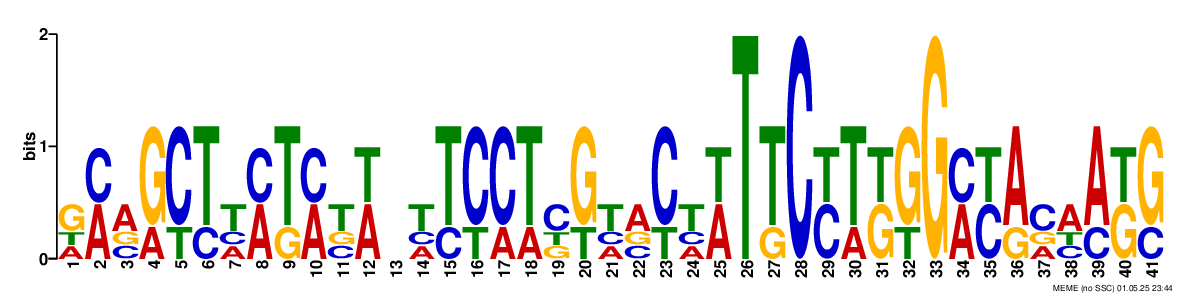

Supplement: Supplementary file 5 — Supplementary Information 5. [file 41598_2025_3586_MOESM5_ESM.zip › Supplementary File S6/meme_out_chip_seq/DynSpansBySourceId_schizont/logo_rc12.png]

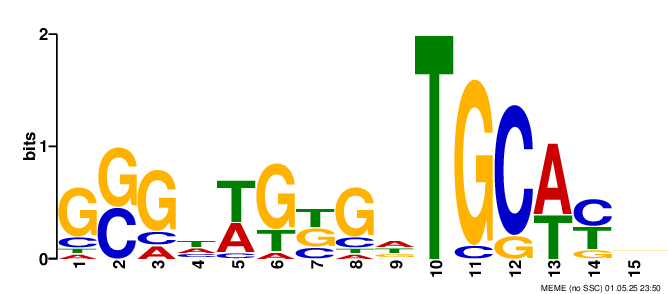

Supplement: Supplementary file 5 — Supplementary Information 5. [file 41598_2025_3586_MOESM5_ESM.zip › Supplementary File S6/meme_out_chip_seq/DynSpansBySourceId_schizont/logo13.png]

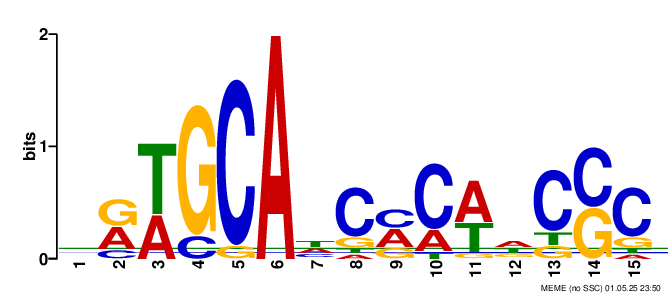

Supplement: Supplementary file 5 — Supplementary Information 5. [file 41598_2025_3586_MOESM5_ESM.zip › Supplementary File S6/meme_out_chip_seq/DynSpansBySourceId_schizont/logo_rc13.png]

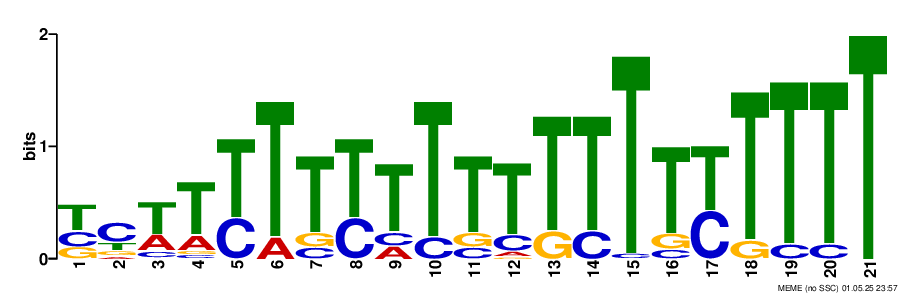

Supplement: Supplementary file 5 — Supplementary Information 5. [file 41598_2025_3586_MOESM5_ESM.zip › Supplementary File S6/meme_out_chip_seq/DynSpansBySourceId_schizont/logo14.png]

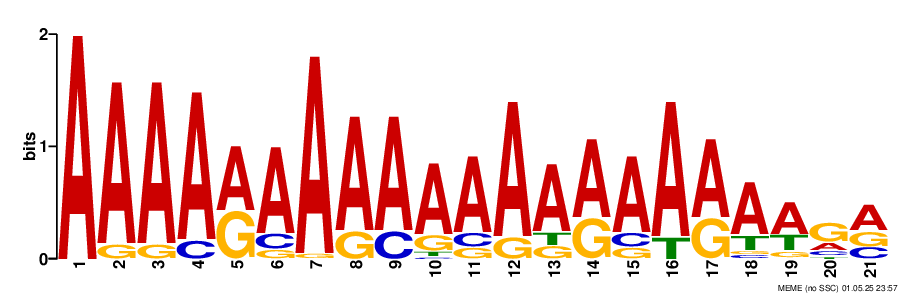

Supplement: Supplementary file 5 — Supplementary Information 5. [file 41598_2025_3586_MOESM5_ESM.zip › Supplementary File S6/meme_out_chip_seq/DynSpansBySourceId_schizont/logo_rc14.png]

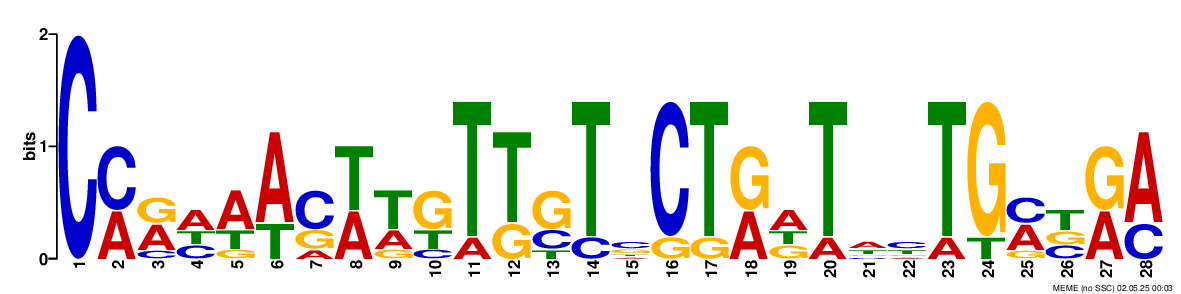

Supplement: Supplementary file 5 — Supplementary Information 5. [file 41598_2025_3586_MOESM5_ESM.zip › Supplementary File S6/meme_out_chip_seq/DynSpansBySourceId_schizont/logo15.png]

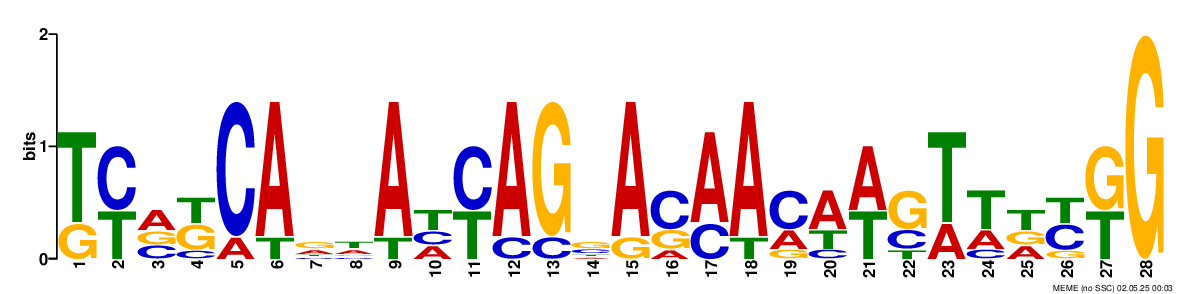

Supplement: Supplementary file 5 — Supplementary Information 5. [file 41598_2025_3586_MOESM5_ESM.zip › Supplementary File S6/meme_out_chip_seq/DynSpansBySourceId_schizont/logo_rc15.png]

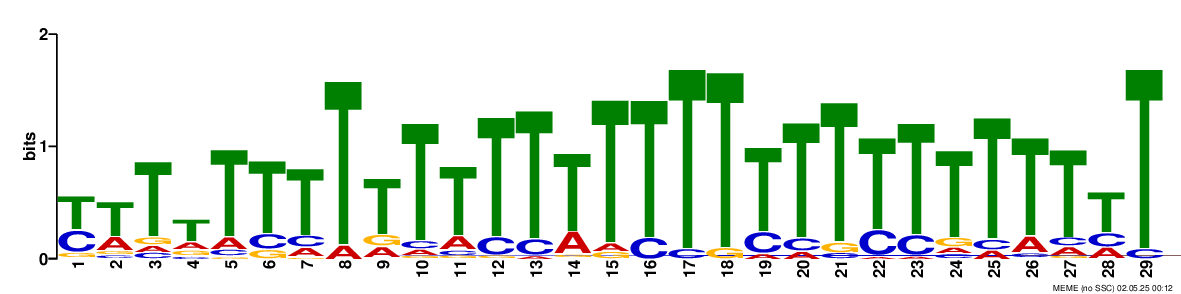

Supplement: Supplementary file 5 — Supplementary Information 5. [file 41598_2025_3586_MOESM5_ESM.zip › Supplementary File S6/meme_out_chip_seq/DynSpansBySourceId_trophozoite/logo1.png]

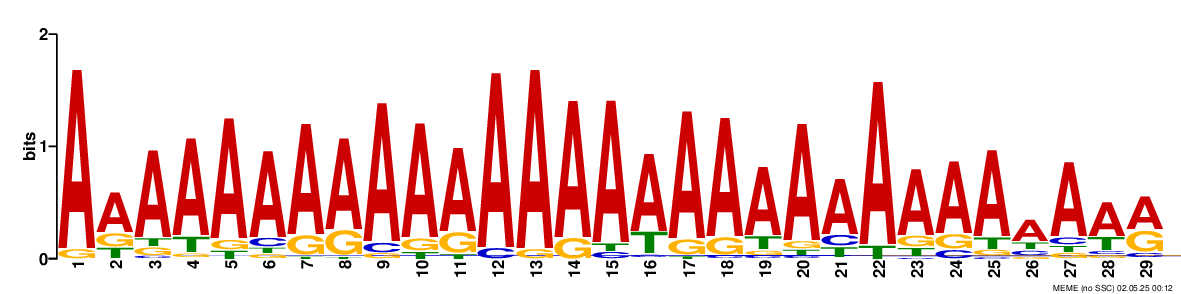

Supplement: Supplementary file 5 — Supplementary Information 5. [file 41598_2025_3586_MOESM5_ESM.zip › Supplementary File S6/meme_out_chip_seq/DynSpansBySourceId_trophozoite/logo_rc1.png]

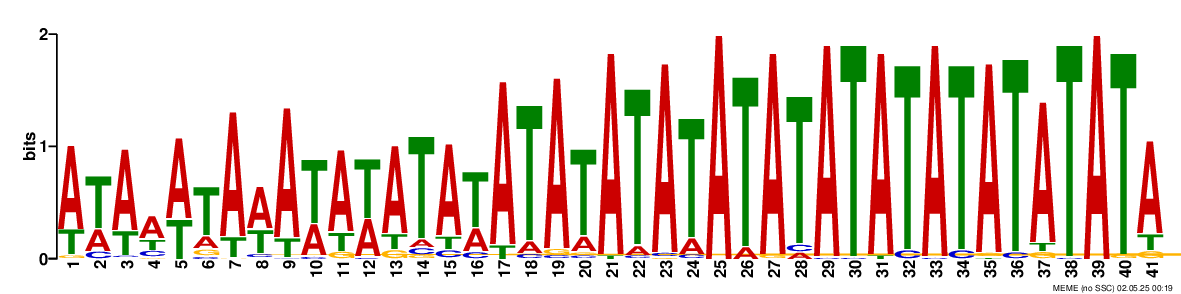

Supplement: Supplementary file 5 — Supplementary Information 5. [file 41598_2025_3586_MOESM5_ESM.zip › Supplementary File S6/meme_out_chip_seq/DynSpansBySourceId_trophozoite/logo2.png]

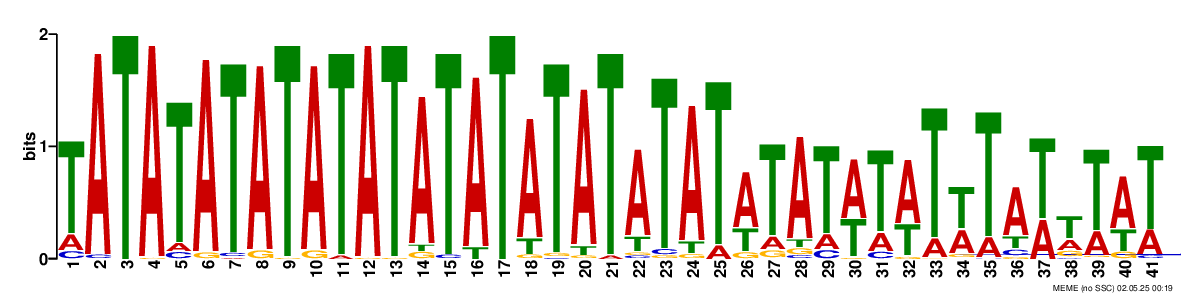

Supplement: Supplementary file 5 — Supplementary Information 5. [file 41598_2025_3586_MOESM5_ESM.zip › Supplementary File S6/meme_out_chip_seq/DynSpansBySourceId_trophozoite/logo_rc2.png]

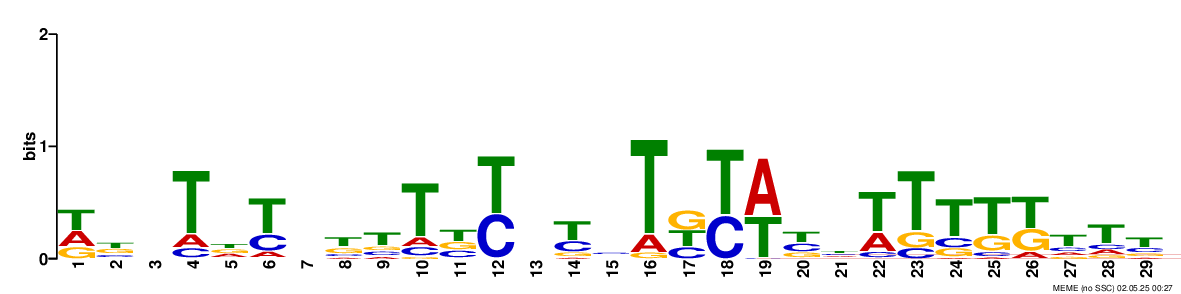

Supplement: Supplementary file 5 — Supplementary Information 5. [file 41598_2025_3586_MOESM5_ESM.zip › Supplementary File S6/meme_out_chip_seq/DynSpansBySourceId_trophozoite/logo3.png]

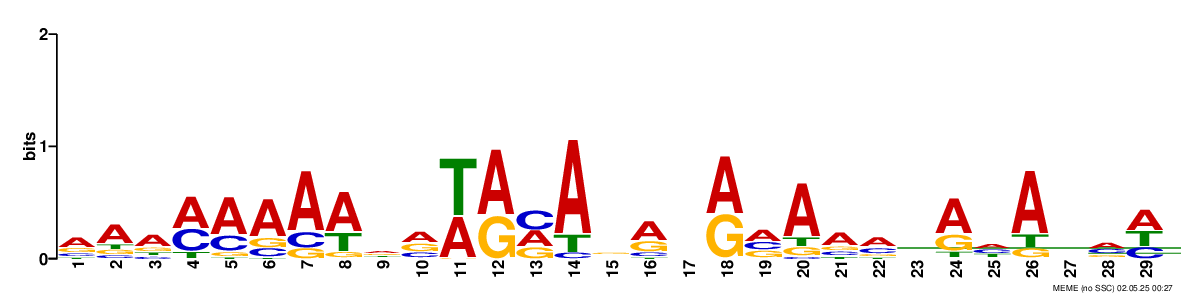

Supplement: Supplementary file 5 — Supplementary Information 5. [file 41598_2025_3586_MOESM5_ESM.zip › Supplementary File S6/meme_out_chip_seq/DynSpansBySourceId_trophozoite/logo_rc3.png]

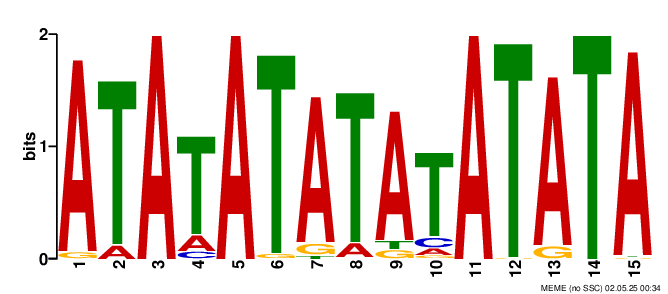

Supplement: Supplementary file 5 — Supplementary Information 5. [file 41598_2025_3586_MOESM5_ESM.zip › Supplementary File S6/meme_out_chip_seq/DynSpansBySourceId_trophozoite/logo4.png]

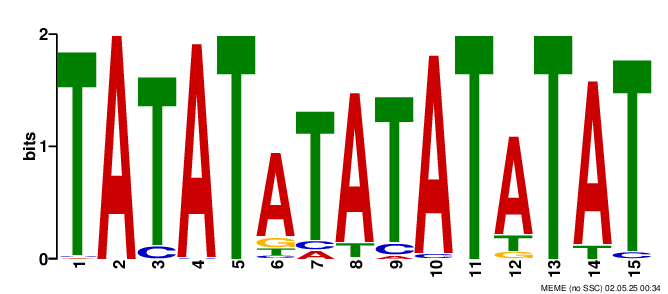

Supplement: Supplementary file 5 — Supplementary Information 5. [file 41598_2025_3586_MOESM5_ESM.zip › Supplementary File S6/meme_out_chip_seq/DynSpansBySourceId_trophozoite/logo_rc4.png]

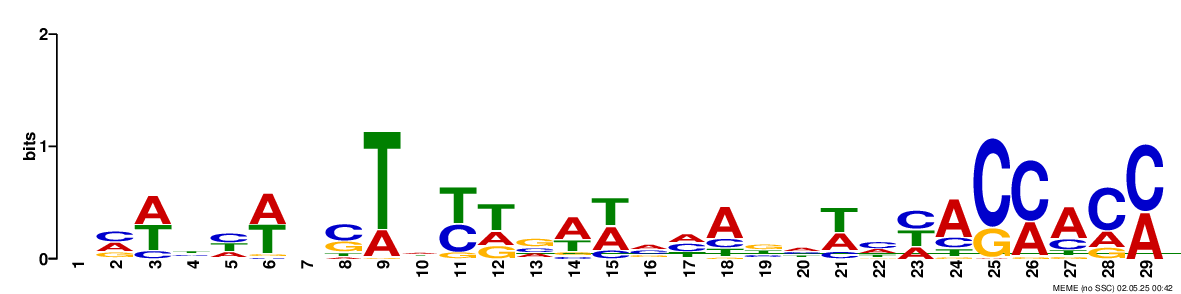

Supplement: Supplementary file 5 — Supplementary Information 5. [file 41598_2025_3586_MOESM5_ESM.zip › Supplementary File S6/meme_out_chip_seq/DynSpansBySourceId_trophozoite/logo5.png]

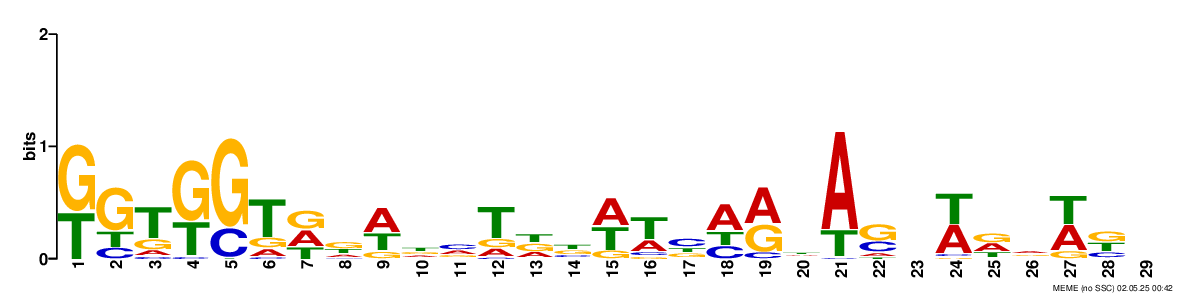

Supplement: Supplementary file 5 — Supplementary Information 5. [file 41598_2025_3586_MOESM5_ESM.zip › Supplementary File S6/meme_out_chip_seq/DynSpansBySourceId_trophozoite/logo_rc5.png]

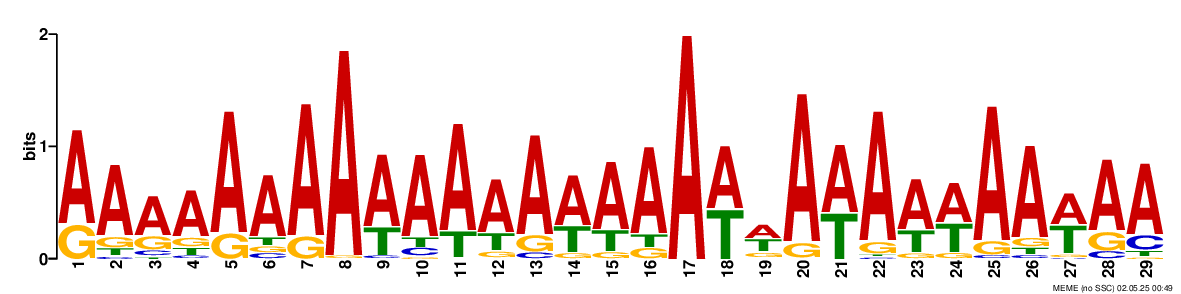

Supplement: Supplementary file 5 — Supplementary Information 5. [file 41598_2025_3586_MOESM5_ESM.zip › Supplementary File S6/meme_out_chip_seq/DynSpansBySourceId_trophozoite/logo6.png]

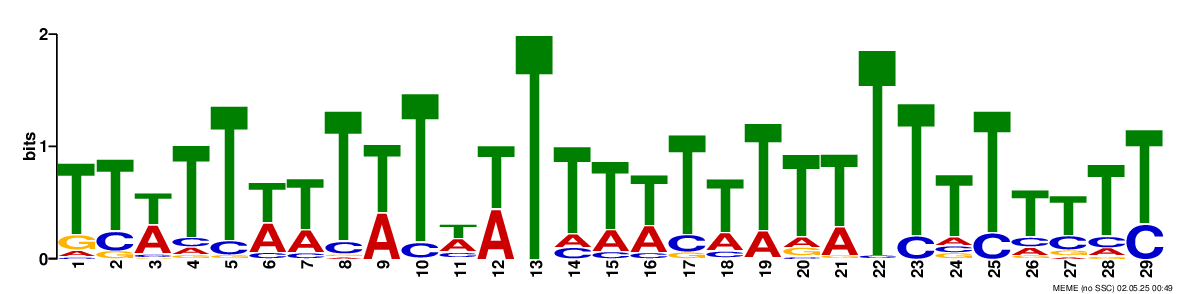

Supplement: Supplementary file 5 — Supplementary Information 5. [file 41598_2025_3586_MOESM5_ESM.zip › Supplementary File S6/meme_out_chip_seq/DynSpansBySourceId_trophozoite/logo_rc6.png]

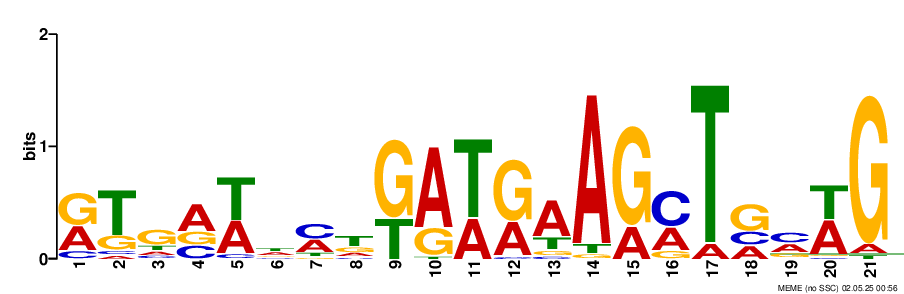

Supplement: Supplementary file 5 — Supplementary Information 5. [file 41598_2025_3586_MOESM5_ESM.zip › Supplementary File S6/meme_out_chip_seq/DynSpansBySourceId_trophozoite/logo7.png]

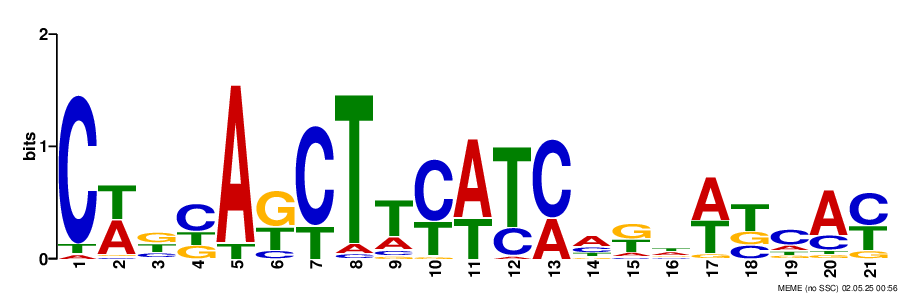

Supplement: Supplementary file 5 — Supplementary Information 5. [file 41598_2025_3586_MOESM5_ESM.zip › Supplementary File S6/meme_out_chip_seq/DynSpansBySourceId_trophozoite/logo_rc7.png]

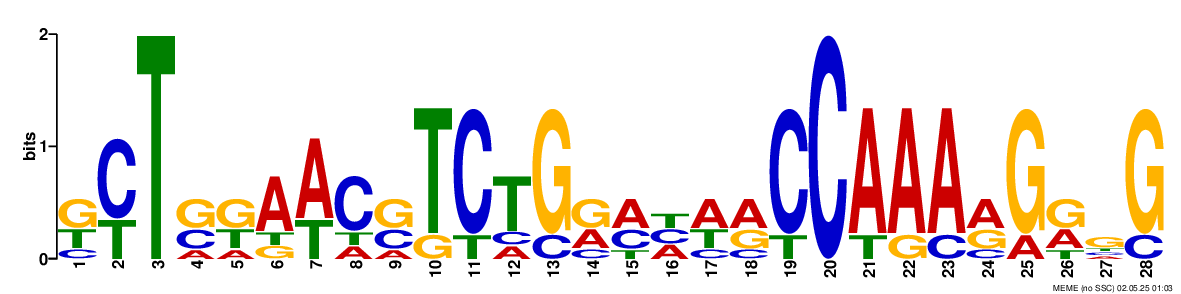

Supplement: Supplementary file 5 — Supplementary Information 5. [file 41598_2025_3586_MOESM5_ESM.zip › Supplementary File S6/meme_out_chip_seq/DynSpansBySourceId_trophozoite/logo8.png]

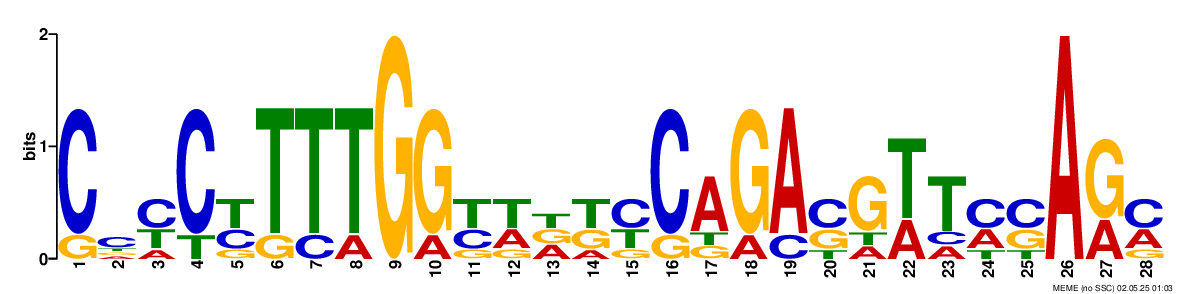

Supplement: Supplementary file 5 — Supplementary Information 5. [file 41598_2025_3586_MOESM5_ESM.zip › Supplementary File S6/meme_out_chip_seq/DynSpansBySourceId_trophozoite/logo_rc8.png]

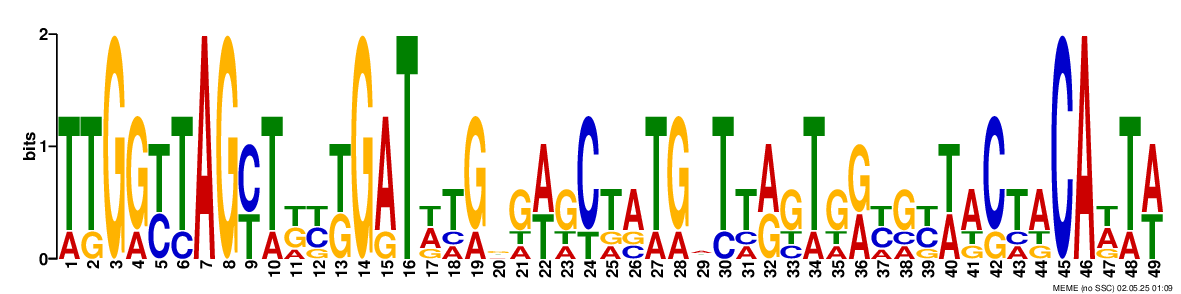

Supplement: Supplementary file 5 — Supplementary Information 5. [file 41598_2025_3586_MOESM5_ESM.zip › Supplementary File S6/meme_out_chip_seq/DynSpansBySourceId_trophozoite/logo9.png]

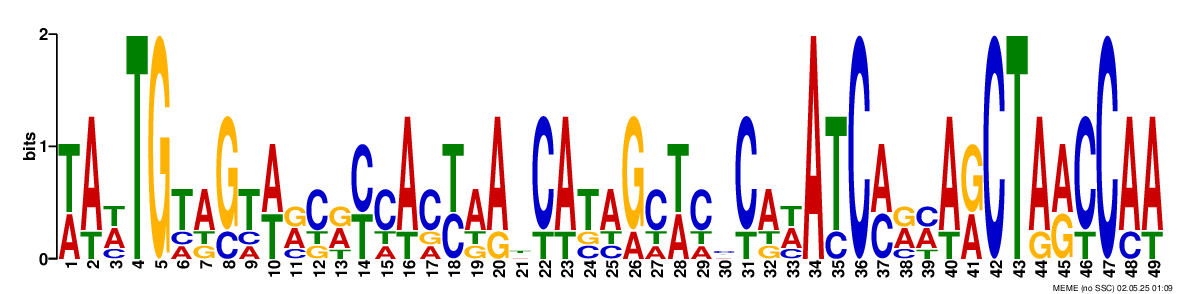

Supplement: Supplementary file 5 — Supplementary Information 5. [file 41598_2025_3586_MOESM5_ESM.zip › Supplementary File S6/meme_out_chip_seq/DynSpansBySourceId_trophozoite/logo_rc9.png]

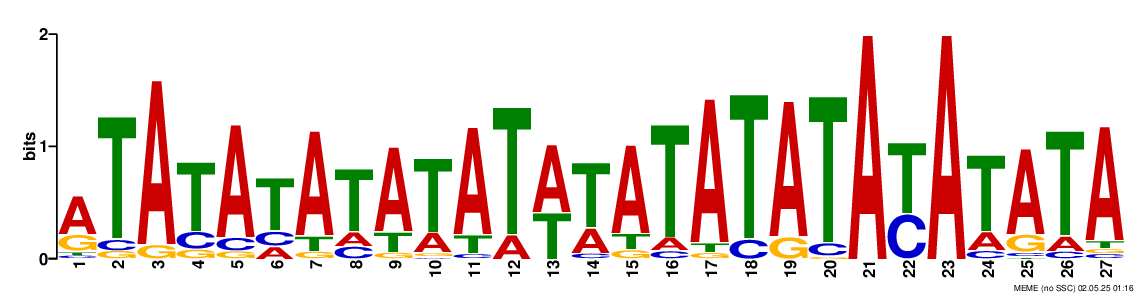

Supplement: Supplementary file 5 — Supplementary Information 5. [file 41598_2025_3586_MOESM5_ESM.zip › Supplementary File S6/meme_out_chip_seq/DynSpansBySourceId_trophozoite/logo10.png]

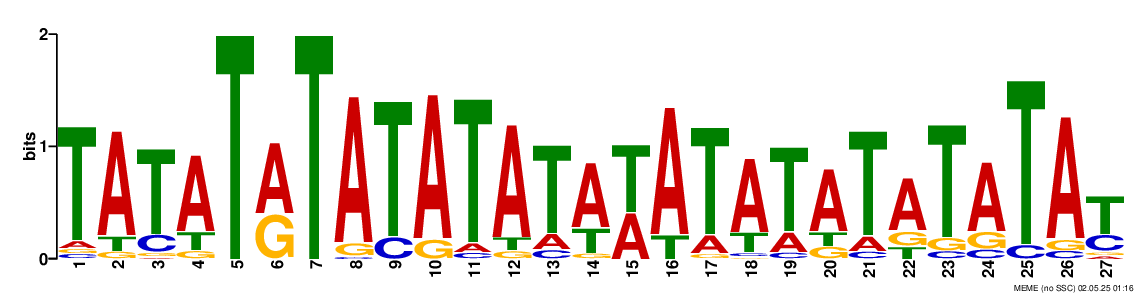

Supplement: Supplementary file 5 — Supplementary Information 5. [file 41598_2025_3586_MOESM5_ESM.zip › Supplementary File S6/meme_out_chip_seq/DynSpansBySourceId_trophozoite/logo_rc10.png]

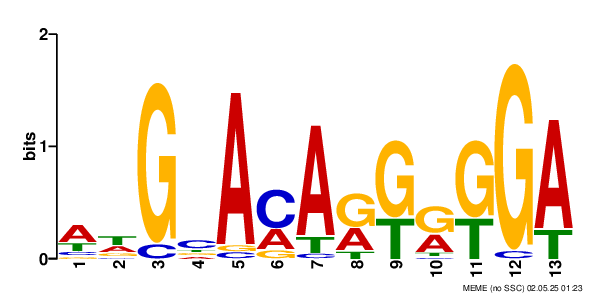

Supplement: Supplementary file 5 — Supplementary Information 5. [file 41598_2025_3586_MOESM5_ESM.zip › Supplementary File S6/meme_out_chip_seq/DynSpansBySourceId_trophozoite/logo11.png]

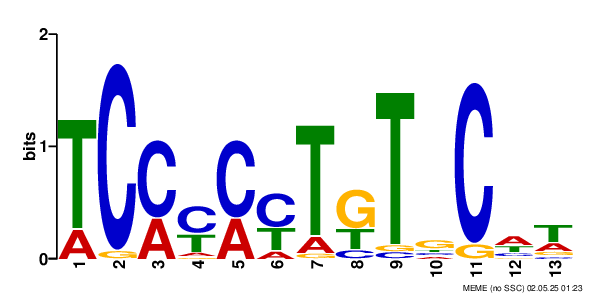

Supplement: Supplementary file 5 — Supplementary Information 5. [file 41598_2025_3586_MOESM5_ESM.zip › Supplementary File S6/meme_out_chip_seq/DynSpansBySourceId_trophozoite/logo_rc11.png]

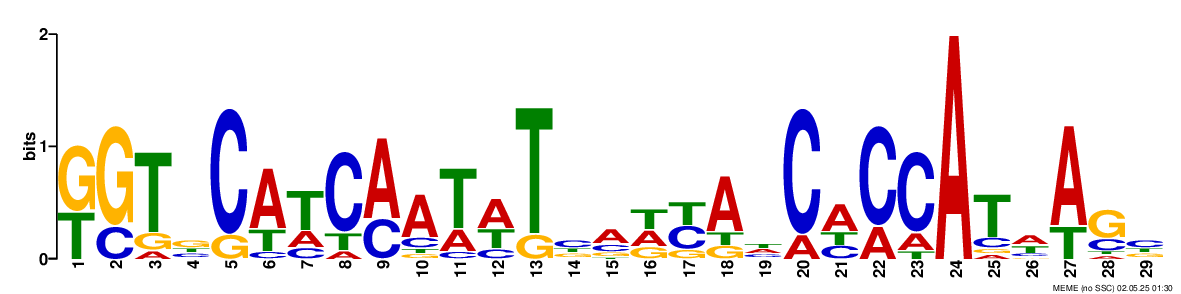

Supplement: Supplementary file 5 — Supplementary Information 5. [file 41598_2025_3586_MOESM5_ESM.zip › Supplementary File S6/meme_out_chip_seq/DynSpansBySourceId_trophozoite/logo12.png]

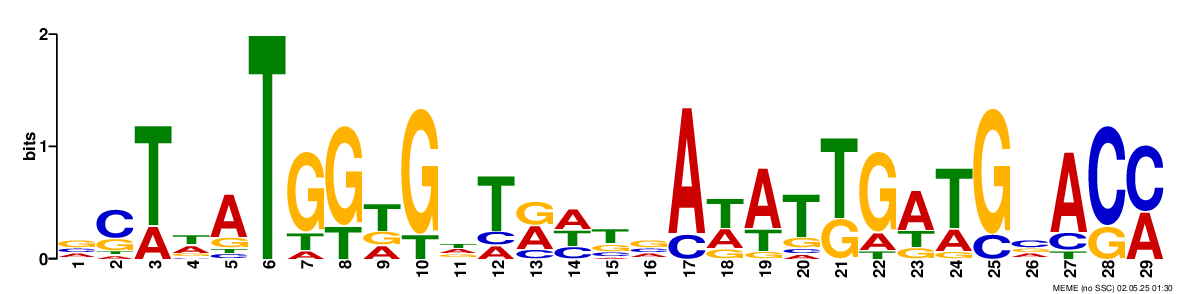

Supplement: Supplementary file 5 — Supplementary Information 5. [file 41598_2025_3586_MOESM5_ESM.zip › Supplementary File S6/meme_out_chip_seq/DynSpansBySourceId_trophozoite/logo_rc12.png]

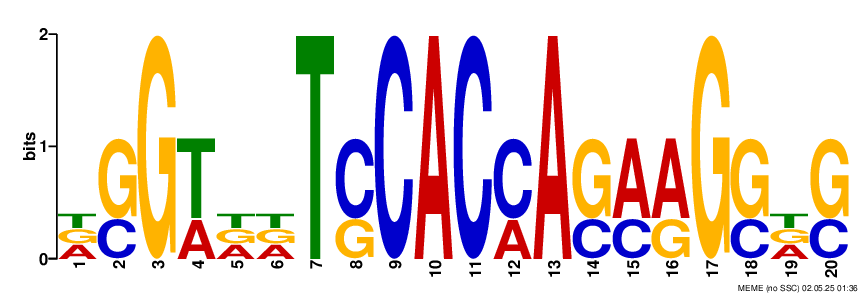

Supplement: Supplementary file 5 — Supplementary Information 5. [file 41598_2025_3586_MOESM5_ESM.zip › Supplementary File S6/meme_out_chip_seq/DynSpansBySourceId_trophozoite/logo13.png]

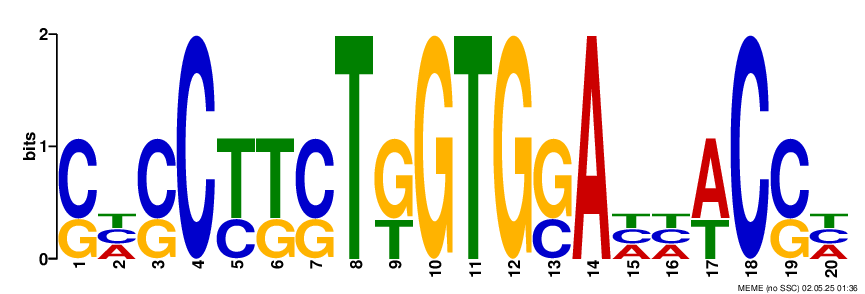

Supplement: Supplementary file 5 — Supplementary Information 5. [file 41598_2025_3586_MOESM5_ESM.zip › Supplementary File S6/meme_out_chip_seq/DynSpansBySourceId_trophozoite/logo_rc13.png]

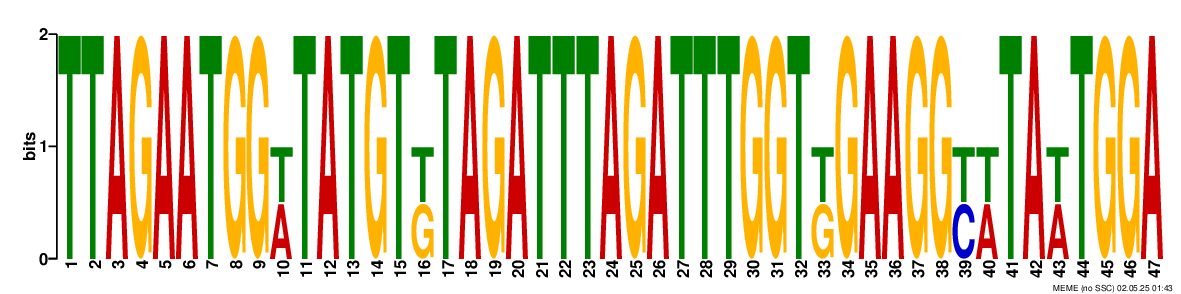

Supplement: Supplementary file 5 — Supplementary Information 5. [file 41598_2025_3586_MOESM5_ESM.zip › Supplementary File S6/meme_out_chip_seq/DynSpansBySourceId_trophozoite/logo14.png]

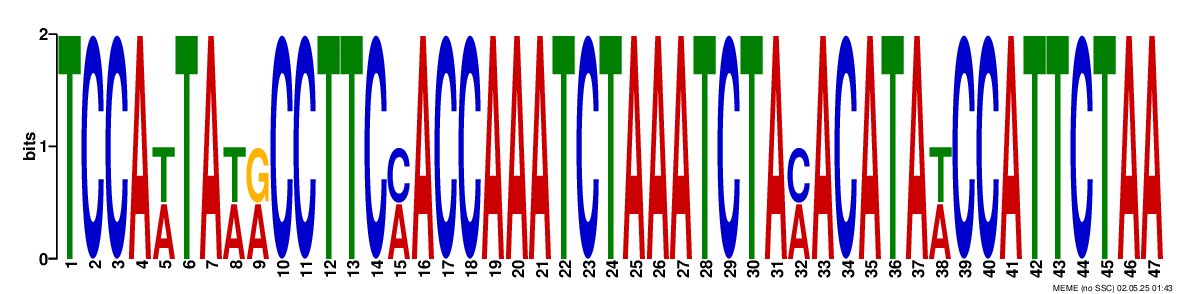

Supplement: Supplementary file 5 — Supplementary Information 5. [file 41598_2025_3586_MOESM5_ESM.zip › Supplementary File S6/meme_out_chip_seq/DynSpansBySourceId_trophozoite/logo_rc14.png]

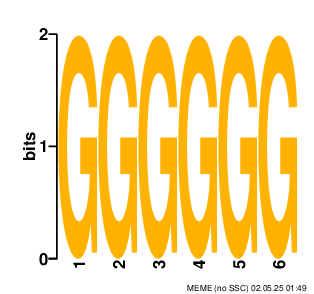

Supplement: Supplementary file 5 — Supplementary Information 5. [file 41598_2025_3586_MOESM5_ESM.zip › Supplementary File S6/meme_out_chip_seq/DynSpansBySourceId_trophozoite/logo15.png]

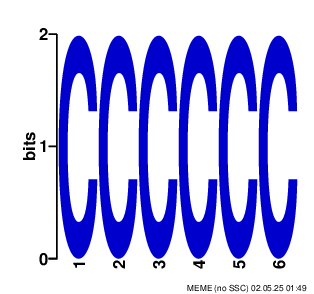

Supplement: Supplementary file 5 — Supplementary Information 5. [file 41598_2025_3586_MOESM5_ESM.zip › Supplementary File S6/meme_out_chip_seq/DynSpansBySourceId_trophozoite/logo_rc15.png]

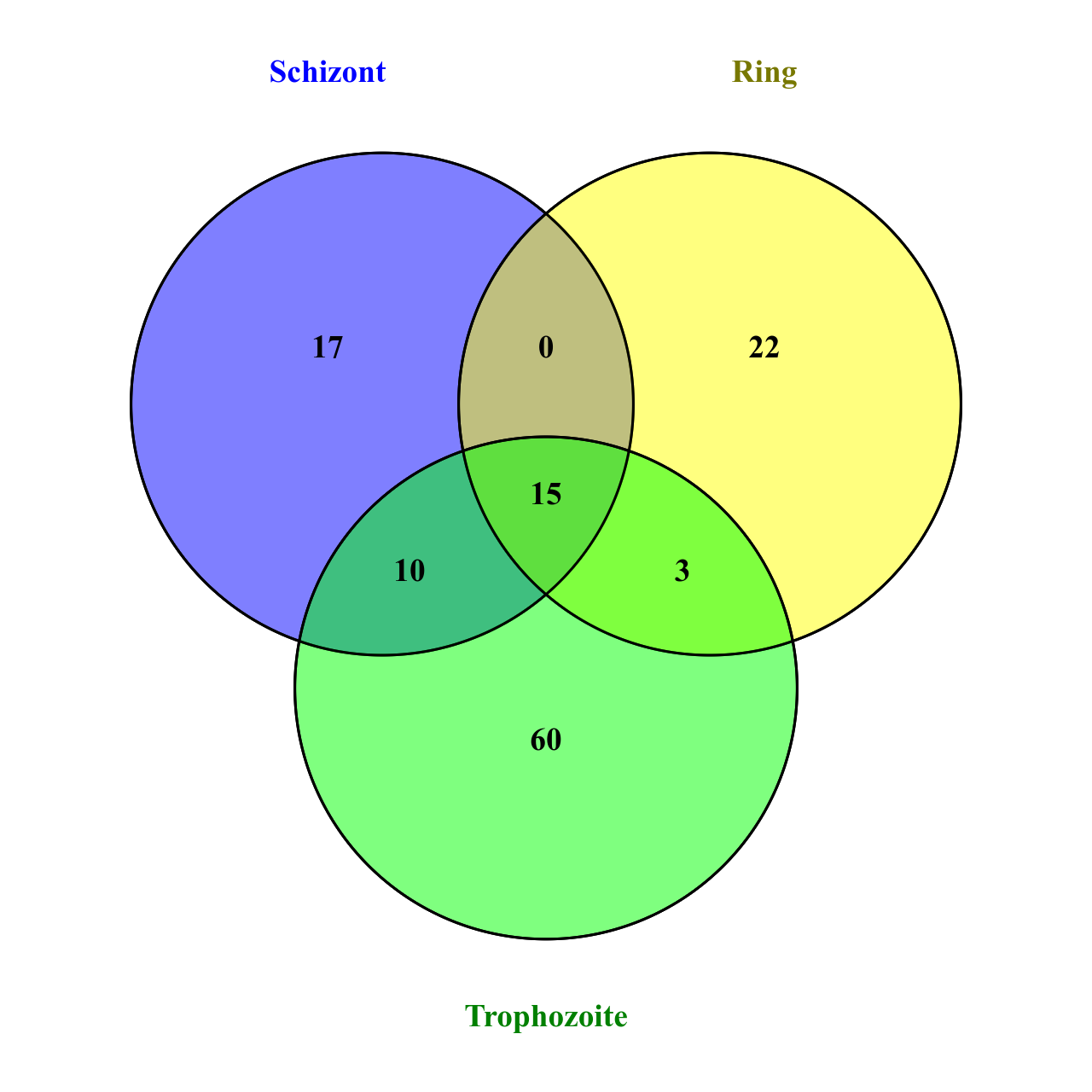

Supplement: Supplementary file 5 — Supplementary Information 5. [file 41598_2025_3586_MOESM5_ESM.zip › Supplementary File S6/venn_hip-seq.png]
