# Supplementary material for: Transcriptome analysis reveals a de novo DNA element that may interact with chromatin-associated proteins in Plasmodium berghei during erythrocytic development
Source: Sci Rep. 2025 May 28;15:18621. doi: 10.1038/s41598-025-03586-4 (PMC12120095; doi:10.1038/s41598-025-03586-4)
Supplement: Supplementary file 6 — Supplementary Information 6. [file 41598_2025_3586_MOESM6_ESM.pdf]

Supplementary figure

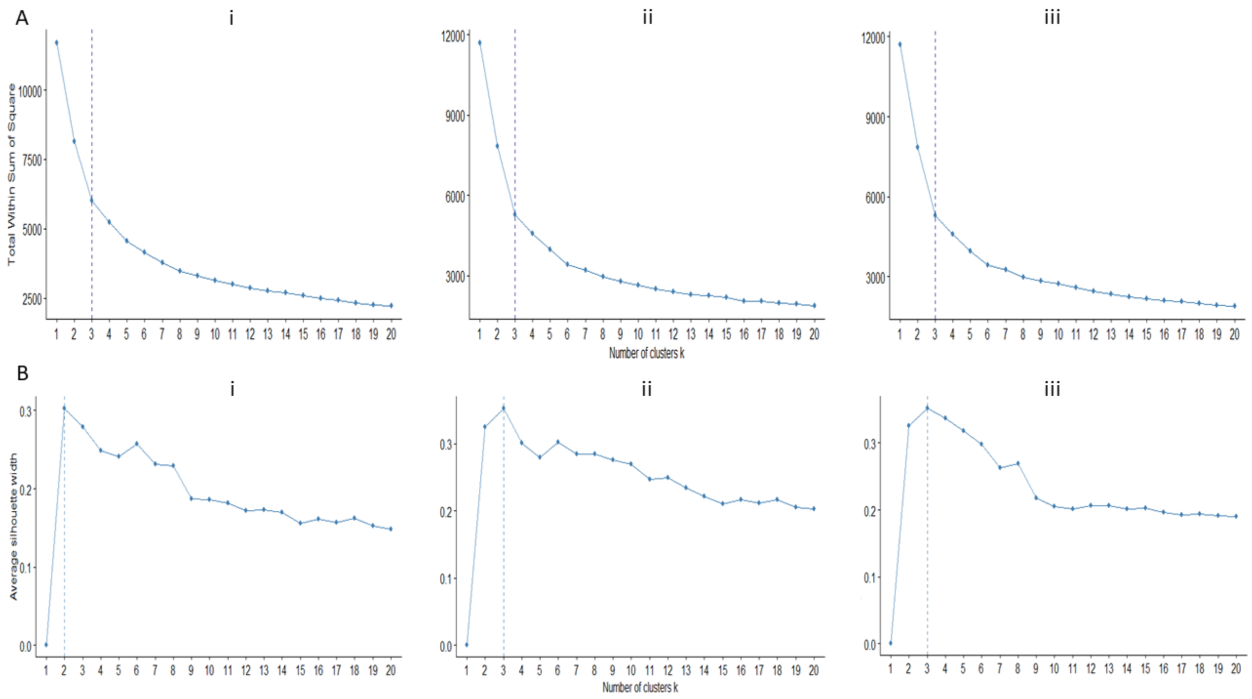

Figure S1: Determining the optimal number of co-expression clusters by A. the elbow method and B. the average silhouette width method. The number of clusters is determined for three algorithms: i. hierarchical ii. K-means and iii. PAM clustering. The vertical dashed line indicates the optimal number of clusters for each algorithm in each method.
