# Supplementary material for: Transcriptome analysis reveals a de novo DNA element that may interact with chromatin-associated proteins in Plasmodium berghei during erythrocytic development
Source: Sci Rep. 2025 May 28;15:18621. doi: 10.1038/s41598-025-03586-4 (PMC12120095; doi:10.1038/s41598-025-03586-4)
Supplement: Supplementary file 8 — Supplementary Information 8. [file 41598_2025_3586_MOESM8_ESM.pdf]

Table S2: Overview of datasets analysed

**Accession number**	**Species/ Strain**	**Library type**	**RNA Selection**	**Instrument**	**Read length**	**Layout**	**Adapter**	**Stages analyzed**	**Study**
ERP105548	*Pb*ANKA	U	Oligo-dT	Illumina HiSeq 2500	100	PE	Nextera	Sporozoite, liver stages	[1]
SRP250329	*Pb*ANKA	U	cDNA	Illumina HiSeq 4000	50	SE	SMART-Seq	Sporozoite, liver stages	[2]
SRP027529/ ERP004740	*Pb*	U		Illumina Genome Analyzer IIx	76	SE/PE	Truseq	Ring, Trophozoite, Schizont, Gametocyte, Ookinete	[3]
SRP099925	*Pb*ANKA	S (BS2-3) / SR (BS1)	Poly(A)	Illumina HiSeq 2500	100	PE		Asexual blood stages (mixed)	[4]
SRP197607	*Pb*ANKA	SR	PCR	Illumina NextSeq 500	75	PE	KAPA	Gametocyte	[5]
SRP073801	*Pb*ANKA	SR	cDNA	Illumina HiSeq 2500	100	PE	NEBNext	Ookinete	[6]
SRP090611	*Pf*NF54 and 3D7	SR	Random	Illumina NextSeq 550	150	SE	Truseq	Gametocyte, Sporozoite	[7]
SRP142460	*Pf*3D7	SR	cDNA	Illumina HiSeq 2500	100	SE	Truseq	Sporozoite	[8]
SRP048710	*Pf*K1	SR	cDNA	Illumina MiSeq		PE	Truseq	Ring, Trophozoite, Schizont	[9]
SRP211863	*Pf*NF54	SR	cDNA	Illumina HiSeq X Ten	150	PE	KAPA AGATCGGAAGAGC AAGATCGGAAGAGC	Ring, Trophozoite, Schizont	[10]
SRP069075	*Pf*3D7	SR	cDNA	Illumina HiSeq 2500		SE	TruSeq	Male and female gametocyte	unpublished
SRP100893	*Pv*	U	Random	Illumina HiSeq 2000 (NextSeq)		PE	TruSeq	Sporozoite	[11]
SRP046739	*Pv*	U	cDNA	Illumina HiSeq 2000	100	PE	TruSeq	Blood stages	[12]

S: stranded, SR: stranded reverse, U: unstranded, SE: single end, PE: paired end, oligo-dT: oligo-deoxythymidine

References

1. Caldelari R, Dogga S, Schmid MW, Franke-Fayard B, Janse CJ, Soldati-Favre D, et al. Transcriptome analysis of Plasmodium berghei during exo-erythrocytic development. Malar J. 2019;18:330.
2. Toro-Moreno M, Sylvester K, Srivastava T, Posfai D, Derbyshire ER. RNA-seq analysis illuminates the early stages of plasmodium liver infection. MBio. 2020;11.
3. Otto TD, Böhme U, Jackson AP, Hunt M, Franke-Fayard B, Hoeijmakers WAM, et al. A comprehensive evaluation of rodent malaria parasite genomes and gene expression. BMC Biol. 2014.
4. Yeoh LM, Goodman CD, Mollard V, McFadden GI, Ralph SA. Comparative transcriptomics of female and male gametocytes in Plasmodium berghei and the evolution of sex in alveolates. BMC Genomics. 2017;18:1–16.
5. Pandey R, Abel S, Boucher M, Holder AA, Le Roch KG, Tewari R. Plasmodium Condensin Core Subunits SMC2/SMC4 Mediate Atypical Mitosis and Are Essential for Parasite Proliferation and Transmission. Cell Rep. 2020;30.
6. Modrzynska K, Pfander C, Chappell L, Yu L, Suarez C, Dundas K, et al. A Knockout Screen of ApiAP2 Genes Reveals Networks of Interacting Transcriptional Regulators Controlling the Plasmodium Life Cycle. Cell Host Microbe. 2017;21:11–22.
7. Zanghì G, Vembar SS, Baumgarten S, Ding S, Guizetti J, Bryant JM, et al. A Specific PfEMP1 Is Expressed in P. falciparum Sporozoites and Plays a Role in Hepatocyte Infection. Cell Rep. 2018;22:2951–63.
8. Lindner SE, Swearingen KE, Shears MJ, Walker MP, Vrana EN, Hart KJ, et al. Transcriptomics and proteomics reveal two waves of translational repression during the maturation of malaria parasite sporozoites. Nat Commun 2019 101. 2019;10:1–13.
9. Shaw PJ, Chaotheing S, Kaewprommal P, Piriyapongsa J, Wongsombat C, Suwannakitti N, et al. Plasmodium parasites mount an arrest response to dihydroartemisinin, as revealed by whole transcriptome shotgun sequencing (RNA-seq) and microarray study. BMC Genomics. 2015;16:1–14.
10. Fan Y, Shen S, Wei G, Tang J, Zhao Y, Wang F, et al. Rrp6 regulates heterochromatic gene silencing via ncrna ruf6 decay in malaria parasites. MBio. 2020;11.
11. Muller I, Jex AR, Kappe SHI, Mikolajczak SA, Sattabongkot J, Patrapuvich R, et al. Transcriptome and histone epigenome of Plasmodium vivax salivary-gland sporozoites point to tight regulatory control and mechanisms for liver-stage differentiation in relapsing malaria. Int J Parasitol. 2019;49:501–13.
12. Zhu L, Mok S, Imwong M, Jaidee A, Russell B, Nosten F, et al. New insights into the Plasmodium vivax transcriptome using RNA-Seq. Sci Reports 2016 61. 2016;6:1–13.
